# Sociodemographic variations of belief in life after death across 22 Countries

**DOI:** 10.1038/s41598-024-83541-x

**Published:** 2025-04-30

**Authors:** Zhuo Job Chen, Richard G. Cowden, Alexander Moreira-Almeida, Thomas Breedlove, Blake Victor Kent, R. Noah Padgett, Byron R. Johnson, Tyler J. VanderWeele

**Affiliations:** 1 School of Nursing, University of North Carolina at Charlotte, Charlotte, NC USA; 2 Human Flourishing Program, Institute for Quantitative Social Science, Harvard University, Cambridge, MA USA; 3 Department of Epidemiology, Harvard T.H. Chan School of Public Health, Boston, MA USA; 4 Research Center in Spirituality and Health (NUPES), School of Medicine, Federal University of Juiz de Fora (UFJF), Juiz de Fora, MG Brazil; 5 Institute for Studies of Religion, Baylor University, Waco, TX USA; 6 Department of Sociology, Westmont College, Santa Barbara, CA USA; 7 Center on Genomics, Vulnerable Populations, and Health Disparities, Harvard Medical School, Massachusetts General Hospital, Boston, MA USA

**Keywords:** afterlife, spiritual belief, multinational, population wellbeing, Global Flourishing Study, Public health, Psychology and behaviour

## Abstract

Belief in life after death is among the oldest forms of spiritual belief, found in nearly every world civilization and religion. While several national surveys highlight differences in afterlife beliefs across countries, none have explored the sociodemographic factors associated with these beliefs. Using data from Wave 1 of the Global Flourishing Study (*N* = 202,898), weighted to be nationally representative, we estimated the proportion of people affirming belief in an afterlife in 22 countries. Primary analyses with demographic variables were conducted separately by country and then pooled using meta-analytic techniques. We examined variations in afterlife belief across nine sociodemographic characteristics: age, gender, marital status, employment status, education level, immigration status, frequency of religious service attendance, religious affiliation, and racial/ethnic identity. The overall proportion of the populations believing in life after death ranged from 95% in Indonesia to 21% in Japan. Meta-analytic results indicated cross-national heterogeneity across all sociodemographic categories, though the degree of variation differed. Random effects meta-analytic results highlighted religious service attendance as a key factor, with the highest belief in the afterlife observed among those attending services at least once per week. These findings provide a foundation for understanding population-level beliefs in the afterlife and continued exploration of their complexity across different contexts.

## Belief in life after death: Sociodemographic Variations across 22 Countries

Belief in life after death stands as one of the oldest manifestations of spiritual belief, finding expressions in almost every world civilization^1^ and established religion^2^. Closely connected with the general agreement among world religions on an immaterial aspect of human existence^3^, the notion that human life transcends corporeal demise is central to much religious belief and practice. Thus, the examination of afterlife beliefs is essential for a comprehensive understanding of religious beliefs.

Afterlife beliefs wield profound influence on values, worldviews, and priorities^4^. Afterlife beliefs can lead to a wide array of actions, from virtuous to hazardous. For example, fear of punishment and hope for a better afterlife may foster ethical conduct and benevolent deeds in the present realm^5^, or religious adherents may be swayed by the promise of posthumous rewards^6^. At the same time, afterlife beliefs may inform a distorted perception of gains from costly and immoral actions, such as those associated with suicide terrorism^7^. Afterlife beliefs may also be used as coping strategy^8^, and is associated with lower anxiety, depression, and cynicism about the world^9,10^.

## Cultural and sociodemographic variations

Several national surveys have estimated the prevalence of belief in life after death. According to a Gallup^11^ survey of 57,768 adults in 61 countries, 57% think that there is a life after death. Although not explicitly examined in the Gallup study, this belief appears closely intertwined with religious affiliation. Individuals in highly religious nations, such as Indonesia, Libya, Pakistan, Sierra Leone, and Senegal exhibit a higher propensity to endorse beliefs in life after death, whereas skepticism tends to prevail among residents of Japan, Vietnam, and several European Union countries where religious affiliation is less widespread.

Beyond religious affiliation, afterlife beliefs may vary across other sociodemographic factors. For instance, women consistently report higher levels of religious belief, including afterlife beliefs^12^. Age also emerges as a potentially influential factor, with spiritual concerns often intensifying with advancing age, a phenomenon referred to as gerotranscendence^13^. However, even younger individuals tend to report belief in life after death, even while many of their perspectives (particularly in secular contexts) are shaped by current societal trends emphasizing spirituality over traditional religiosity^14,15^. This is exemplified by data from the UK in the World Values Survey^16^, where despite a decline in belief in God over the past four decades, belief in life after death remains prevalent, with 46% of respondents expressing such convictions.

The influence of education on afterlife beliefs is multifaceted. While higher education may attenuate adherence to exclusivist religious doctrines, it often boosts a preference for institutionalized religion^17^. The relationship between cognitive complexity and religiosity is nuanced, as individuals operate within both reflective and non-reflective belief modes^18^. Moreover, empirical evidence from a comprehensive 14-study experimental research project demonstrates that analytical thinking does not inherently conflict with religious commitment^19^.

### The Present study

Variations in beliefs about life after death at the country level may be influenced by sociodemographic factors, although this aspect remains largely unexplored in existing literature. To help make strides toward addressing this gap, this study aims to investigate associations between demographic variables and afterlife beliefs within and across nations. Drawing on nationally representative data from 22 countries, our study pursues two main objectives: (1) to examine the distribution of afterlife beliefs within each cultural context and (2) to assess potential sociodemographic disparities in afterlife beliefs within and across these countries.

As an initial step, we provide a descriptive analysis of sociodemographic characteristics such as age, gender, marital status, employment status, educational attainment, frequency of religious service attendance, and immigration status. Although our primary focus lies in exploring the distribution of afterlife beliefs across countries, we anticipate encountering meaningful cross-national variation in these patterns. Building upon prior research^11^, we hypothesize that sociodemographic factors will play a significant role in shaping afterlife beliefs across all countries. In particular, it is expected that religious service attendance will demonstrate the strongest association with afterlife beliefs. While our primary emphasis rests on investigating sociodemographic disparities in afterlife beliefs across diverse cultural contexts, we also anticipate observing some degree of cross-national variation in these sociodemographic differences. This expectation stems from the unique sociocultural landscapes present in each country^20,21^, which may influence the manifestation and interpretation of afterlife beliefs among different demographic groups.

## Results

The sociodemographic characteristics of the total sample are detailed in Table 1. Countries with the largest representation were the United States (19%), Japan (10%), and Sweden (7.4%), while the smallest samples came from Turkey (0.7%), South Africa (1.3%), and Hong Kong (1.5%). The most represented age group was 30–39 years (20%), followed by 40–49 years (17%) and 50–59 years (16%). Gender distribution was nearly equal, with 49% male and 51% female. A majority of participants were married (53%), employed (57%), had 9–15 years of education (57%) and were born in the country where data were collected (94%). Regarding religious service attendance, 32% attended at least once a week, whereas 37% never attended.

Nationally representative sociodemographic characteristics of each country are reported in Supplemental Tables S1a to S22a, with some variability observed across the countries. For example, the percentage of the population who never attend any religious services annually is notably higher in Japan (77%), Australia (67%), and Sweden (66%) compared to Nigeria (1.1%), Kenya (4.1%), and Indonesia (4.8%).

Table 2 provides the ordered proportions of countries of people who believe in life after death. The countries with the highest proportions were Indonesia (0.95), Nigeria (0.73), and Kenya (0.72), while the lowest were Japan (0.21), Sweden (0.29), and Germany (0.34). These trends may be influenced by factors such as secularization, industrialization, and demographic shifts — countries with lower belief proportions tend to be more industrialized with aging populations, while those with higher proportions often exhibit a youth bulge and rapid urbanization.

The lower proportions of endorsement may be partly attributed to the survey offering an ‘Unsure’ response, which some participants selected as an alternative to ‘Yes.’ Supplement Table 24 provides the ordered proportions analogous to Table 2 but dichotomizes responses as Yes/Unsure versus No. Overall, the proportions of people endorsing either ‘Yes’ or ‘Unsure’ for belief in the afterlife were significantly higher, ranging from 0.98 to 0.57. The countries with the highest proportions were Indonesia (0.98) and the Philippines (0.92), while India (0.57) and Sweden (0.63) had the lowest. Supplement Table 25 shows the ordered proportions of those who responded ‘Unsure.’ Japan had the highest proportion of ‘Unsure’ responses (0.47), despite having the lowest proportion of ‘Yes’ responses among all countries. These variations and uncertainties underscore the complexity of endorsing belief in the afterlife across these countries.

## Sociodemographic differences in afterlife belief

Table 3 presents the results of random effects meta-analyses. The overall meta-analytic mean was 0.54. Across countries, 95% confidence intervals (CIs) for the proportions revealed significant differences between categories of religious service attendance. Specifically, belief in the afterlife was lower among those who never attend religious services (0.38, 95% CI: 0.30, 0.46) or attend a few times a year (0.53, 95% CI: 0.45, 0.61) compared to those who attend weekly (0.73, 95% CI: 0.66, 0.78) or more frequently (0.78, 95% CI: 0.71, 0.84). On average across countries, the meta-analytic mean proportion were similar across categories of gender, marital status, employment, education, or immigration status across countries. For each of these sociodemographic characteristics, the meta-analytic average proportion was commonly between 0.50 and 0.60 with a few exceptions such as those reporting to be in a domestic partnership (43%). The pattern with age was relatively constant, with a slight inverted-U-shaped pattern from 53% age 18–24, to 57% age 50–59, down to 51% age 70–79, but then those aged 80 + reportedly notably higher at 75%.

Tau values indicate cross-country variation in afterlife belief across sociodemographic categories, although variation was greater for some categories than others. For example, tau was higher for the 80 or older age category (τ = 0.55) compared to other age groups (τ = 0.20–0.22), suggesting greater cross-national variability in afterlife belief within this older age group; however, the observed sample size for this group was also quite small in several countries leading to large uncertainty in these estimates.

All global p-values in Table 3 passed the Bonferroni-corrected significance threshold, indicating differences for each sociodemographic variable in at least one country. Country-specific results for sociodemographic differences in afterlife belief are detailed in Supplemental Tables S1b–S22b, with accompanying forest plots ranking values by country for each sociodemographic category (see Supplemental Figures S1–S34), and the difference in proportions between each category and a refence category (see Supplemental Figures S35–S115).

We identified instances where country-specific differences between sociodemographic categories emerged that were not observed in the random effects meta-analyses. Pooled meta-analytic results suggested that afterlife belief were slightly increasing with age through age 50–59. However, in many countries young adults reported higher belief than middle aged and older adults (e.g., Japan, Germany). As noted above, in the oldest group (80+) there was a markedly higher belief (see Supplemental Figure S8). Although meta-analytic results did not suggest much difference in afterlife belief by gender, country-specific variations were evident. For example, women exhibited much higher belief in Australia, Poland, and Sweden, whereas men had significantly higher belief in Egypt and Tanzania. On average, educational level was not strongly associated with belief when results were pooled across countries; however, there were notable differences between education groups in some countries. More years of education were linked to higher belief in some countries such as Brazil, Egypt, Indonesia, Kenya, Mexico, the Philippines, Tanzania, and the United Kingdom. In contrast, an inverse correlation was observed in Australia, India, Israel, South Africa, Spain, Sweden, Turkey, and the United States.

Country-specific variation in afterlife belief based on religious affiliation and racial/ethnic identity (when available) was also estimated (see Supplemental Tables S1b–S22b). These variables were not included in the meta-analysis due to differing assessments across countries. However, the results do provide some evidence of meaningful differences in certain countries. For example, in a highly religious country like South Africa (predominantly Christian), the proportion of individuals endorsing afterlife belief among those with no religion was similar to that of Christians and other religious groups (see Supplemental Table S16b). In contrast, in a more secular country like Sweden (see Supplemental Table S18b), belief endorsement among the nonreligious was significantly lower than among the religious. Regarding racial differences belief was, for example, significantly lower among those who self-identified as White compared to other racial/ethnic minority groups in the United Kingdom (see Supplemental Table S21b). When we repeated the meta-analyses using a population-weighted approach, where each country’s results were weighted by its 2023 population size, the pattern of results was largely consistent with those from the random-effects meta-analysis (see Supplemental Table S23). Some slight differences emerged, which may be due in part to the substantial weight given to India.

## Discussion

In this study, we utilized multinational data from wave 1 of the Global Flourishing Study (GFS) to explore the distribution of afterlife belief across 22 geographically and culturally diverse countries. Three main findings emerged: (1) evidence of cross-national variation in afterlife belief, (2) sociodemographic differences across countries, and (3) cross-national variation in sociodemographic differences in afterlife belief.

Our results reveal substantial country-specific variation in afterlife belief. For example, Indonesia, Nigeria, and Kenya had the highest proportions of people believing in life after death, whereas Japan, Sweden, and Germany had the lowest. Perhaps unsurprisingly, religious involvement may be the most significant of several factors potentially contributing to this heterogeneity. The countries with the highest belief in afterlife are predominantly religious societies where active religious participation is common: Indonesia (92% affiliated with Islam, 4.8% never attend religious services), Nigeria (51% Christianity, 48% Islam, 1.1% never attend religious services), and Kenya (91% Christianity, 4.1% never attend religious services). Conversely, countries with lower belief rates are more secular, such as Japan (61% with no religion, 77% never attend religious services), or report greater identification with religion than religious attendance, such as Sweden (55% Christianity, 38% no religion, 66% never attend religious services) and Germany (53% Christianity, 40% no religion, 62% never attend religious services).

When isolating the “Unsure” responses, we found that India had the lowest percentage (57%) of combined Yes/Unsure responses. This figure aligns precisely with data from the World Values Survey 2017–2022 wave for the Yes/Don’t Know category. One potential contributor to India’s lower belief in the afterlife is its lower rate of religious attendance. Despite being one of the most religious countries, with 81% of the sample identifying as Hindu, 12% as Muslim, and only 0.1% reporting no religion, only 48% of the Indian sample attended religious services at least once a week. Hinduism emphasizes different aspects of religious observance, such as adherence to norms of purity, rather than compulsory attendance at temples.

The high rate of “Unsure” responses in Japan may reflect the country’s complex religious landscape and the unique ways religion is perceived and practiced there. Although Japan is a highly secular country, Shinto and Buddhist traditions remain deeply embedded in its society. Many Japanese individuals participate in religious rituals and ancestor worship, reflecting an integration of spiritual practices into daily life rather than organized religious observance^22^.

## Sociodemographic differences in afterlife belief

Using a random-effects meta-analysis, we examined the proportion of people believing in the afterlife across different demographic groups in 22 countries. We found that belief was highest among those who attend religious services at least once per week and lowest among those who never attend. This difference may be influenced by the degree of religiosity and the dominant religious affiliation in these countries. In countries with a dominant religion (where over 40% of the population adheres to one religion), such as Christianity in Argentina, Australia, Germany, Kenya, Mexico, the Philippines, Poland, Spain, and the US; Islam in Egypt, Indonesia, and Turkey; Judaism in Israel; and Hinduism in India, the dominant religion tends to drive belief in an afterlife. South Africa appears to be an exception to this pattern, as only about 52% of Christians (53% endorsed ‘Yes’ in the general population, and 18% endorsed ‘Unsure’) endorse afterlife belief, despite the country being over 80% Christian. The unique landscape of South African Christianity may be shaped by a complex historical, cultural, and social context, including the influence of African spirituality, Pentecostalism, and the legacy of apartheid.

Some countries, like Nigeria and Tanzania, have similar proportions of Christians and Muslims, with both religions showing strong endorsement of afterlife belief, although Muslims in Tanzania are more likely to believe in the afterlife than Christians. In more secular countries, where no single religion represents more than 40% of the population and the majority is non-religious, such as Hong Kong (50% no religion followed by 25% Christianity) and Japan (61% no religion followed by 33% Buddhism), being religious is still associated with higher belief in the afterlife. In countries with a large Christian population alongside a substantial non-religious group (over 30%), like Sweden and the UK, Christian belief in the afterlife is lower (below 50% of the Christian population).

These findings should be considered in light of the complex understandings of life and the afterlife, which vary significantly across different religious traditions. For instance, in Muslim contexts, heaven (Jannah) is envisioned as a paradise of joy and delights, with levels of divine pleasure based on one’s righteousness^23^. In Christian contexts, heaven is often viewed as God’s dwelling place, where people from all nations gather to worship him—a vision of communal worship (e.g. expressed famously in St. Augustine’s account of the beatific vision)—but there is significant variation among Christians concerning the role of individual righteousness determining one’s destiny after death. In Dharmic traditions (e.g., Buddhism and Hinduism), karma plays a central role in afterlife beliefs, with present actions influencing future cycles of death and rebirth. Adding to this complexity, even within traditions that emphasize eternal reward and punishment, there is variability. For example, among U.S. adults, 71% believe in heaven, but only 61% believe in hell^24^. These diverse motivations and expectations for the afterlife suggest that the prevalence of different afterlife beliefs may be influenced by complex relationships between these beliefs and other factors, and future research on the effects of afterlife beliefs can account for differences between specific beliefs.

Afterlife belief can also form experiences and perceptions of ultimate meaning, purpose, and transcendence that is not explicitly connected with organized religious belief and practice. These enhanced perceptions of meaning and purpose may have to do with aging and the life review process, by which older people reflect on their lives, resolve conflicts, and prepare for death^25^. In the aggregated analysis, we found that belief in the afterlife was highest among oldest age group (80+), although this varied significantly across countries. For example, countries like Indonesia, Egypt, and Nigeria show high proportions of afterlife belief among those over 60, while in Japan, Sweden, and Germany, the proportion of older individuals who believe in the afterlife tends to be less. This variation may, again, be influenced by the different religious landscapes in these countries, and/or the age-period-cohort effects^26^. We anticipate that data collected in the coming years of the Global Flourishing Study will further clarify the roles of age and religion in shaping afterlife beliefs. While anyone may hold beliefs about the afterlife, proximity to the end of life may generally heighten such considerations.

A major strength of this study is the use of large, nationally representative samples to document belief in afterlife across multiple countries. By estimating belief in afterlife at both national and subpopulation levels, this study lays the groundwork for a more focused study of this important human universal belief. However, there are some methodological limitations. First, afterlife belief was assessed with a single item, which may capture the essence of the construct but overlook its complexity and how people understand life after death. Second, while wave 1 of the GFS includes 22 geographically and culturally diverse countries, it does not represent all global contexts (e.g., mainland China), so caution is needed when generalizing findings. (Note: Data from Hong Kong (S.A.R. of China) is available in the first wave of data collection. Data from mainland China were not included in the first data release due to fieldwork delays. The first wave of fieldwork in mainland China was completed in February 2024, and a second wave is expected to be completed in November-December 2024. All wave 1 and 2 data from mainland China will be part of the second dataset release in March 2025.)

Third, caution is also needed in interpreting cross-national differences as these may be influenced by matters of translation, different modes of assessment, cultural norms, interpretation of items and also of response scales, and seasonal effects arising from data being collected in different countries at different times of the year. Additionally, it’s important to acknowledge the cross-cultural differences in respondents’ interpretations and responses to the GFS survey items^21,27^. Although extensive pretesting and cognitive interviewing took place in each of the 22 countries included in the GFS, the modifications and revisions to the survey may not have accounted for all possible differences between countries when aligning the survey^28^.

Finally, the cross-sectional nature of the data is purely descriptive, and the sociodemographic associations do not account for covariates or interrelations between sociodemographic indicators. For example, although religious participation was associated with stronger belief in an afterlife, it remains unclear whether gender plays a role, as women were more likely to attend religious services and also exhibited higher levels of religious beliefs. There may also be variables that moderate this association, such as attachment to God. A key objective for future studies would be to investigate the underlying causes of variation in afterlife belief across countries, such as national differences in sex, age and educational level. Upcoming waves of the GFS will provide opportunities to explore these possibilities further.

**Table 1:** Nationally representative sociodemographic characteristics of the observed sample (*N* = 202,898).

Characteristic	*n* (%)
Age group	
18–24	27,007 (13%)
25–29	20,700 (10%)
30–39	40,256 (20%)
40–49	34,464 (17%)
50–59	31,793 (16%)
60–69	27,763 (14%)
70–79	16,776 (8.3%)
80 or older	4,119 (2.0%)
Missing	20 (< 0.1%)
Gender	
Male	98,411 (49%)
Female	103,488 (51%)
Other	602 (0.3%)
Missing	397 (0.2%)
Marital status	
Married	107,354 (53%)
Separated	5,195 (2.6%)
Divorced	11,654 (5.7%)
Widowed	9,823 (4.8%)
Never	52,115 (26%)
Domestic Partner	14,931 (7.4%)
Missing	1,826 (0.9%)
Employment	
Employed for an employer	78,815 (39%)
Self-employed	36,362 (18%)
Retired	29,303 (14%)
Student	10,726 (5.3%)
Homemaker	21,677 (11%)
Unemployed and looking for a job	16,790 (8.3%)
None of these/other	8,431 (4.2%)
Missing	793 (0.4%)
Religious service attendance	
> 1/week	26,537 (13%)
1/week	39,157 (19%)
1–3/month	19,749 (9.7%)
A few times a year	41,436 (20%)
Never	75,297 (37%)
Missing	722 (0.4%)
Education	
Up to 8 years	45,078 (22%)
9–15 years	115,097 (57%)
16 + years	42,578 (21%)
Missing	146 (< 0.1%)
Immigration	
Born in this country	190,998 (94%)
Born in another country	9,791 (4.8%)
Missing	2,110 (1.0%)
Country	
Argentina	6,724 (3.3%)
Australia	3,844 (1.9%)
Brazil	13,204 (6.5%)
Egypt	4,729 (2.3%)
Germany	9,506 (4.7%)
Hong Kong (SAR)	3,012 (1.5%)
India	12,765 (6.3%)
Indonesia	6,992 (3.4%)
Israel	3,669 (1.8%)
Japan	20,543 (10%)
Kenya	11,389 (5.6%)
Mexico	5,776 (2.8%)
Nigeria	6,827 (3.4%)
Philippines	5,292 (2.6%)
Poland	10,389 (5.1%)
South Africa	2,651 (1.3%)
Spain	6,290 (3.1%)
Sweden	15,068 (7.4%)
Tanzania	9,075 (4.5%)
Turkey	1,473 (0.7%)
United Kingdom	5,368 (2.6%)
United States	38,312 (19%)

**Table 2:** Proportion endorsing belief in life after death by country.

Country	Proportion (95% CI)	Standard Deviation
Indonesia	0.95 (0.94, 0.96)	0.22
Nigeria	0.73 (0.71, 0.75)	0.44
Kenya	0.72 (0.71, 0.73)	0.45
Turkey	0.67 (0.64, 0.70)	0.47
Egypt	0.64 (0.62, 0.66)	0.48
Brazil	0.61 (0.60, 0.62)	0.49
Tanzania	0.61 (0.59, 0.63)	0.49
Mexico	0.60 (0.59, 0.62)	0.49
Argentina	0.58 (0.56, 0.60)	0.49
Poland	0.57 (0.55, 0.60)	0.49
United States	0.56 (0.54, 0.57)	0.50
Israel	0.55 (0.51, 0.58)	0.50
Philippines	0.54 (0.52, 0.56)	0.50
South Africa	0.53 (0.50, 0.56)	0.50
India	0.48 (0.47, 0.49)	0.50
Hong Kong (SAR)	0.45 (0.43, 0.48)	0.50
Spain	0.40 (0.38, 0.42)	0.49
United Kingdom	0.39 (0.37, 0.41)	0.49
Australia	0.36 (0.34, 0.38)	0.48
Germany	0.34 (0.33, 0.36)	0.47
Sweden	0.29 (0.28, 0.30)	0.46
Japan	0.21 (0.20, 0.22)	0.40

**Table 3:** Random effects meta-analysis for proportion endorsing belief in life after death by sociodemographic characteristic.

Variable	Proportion (95% CI)	Prediction interval		τ	I^2^	Global *p*-value
		Lower	Upper			
Age group						< 0.001
18–24 years	0.53 (0.45, 0.62)	0.25	0.96	0.21	96.7	
25–29 years	0.55 (0.46, 0.63)	0.23	0.96	0.20	96.6	
30–39 years	0.55 (0.46, 0.64)	0.22	0.94	0.21	96.7	
40–49 years	0.56 (0.47, 0.64)	0.22	0.95	0.20	96.6	
50–59 years	0.57 (0.48, 0.66)	0.22	0.93	0.21	96.8	
60–69 years	0.55 (0.46, 0.64)	0.21	0.92	0.22	97.0	
70–79 years	0.51 (0.42, 0.60)	0.16	0.88	0.22	96.9	
80 years or older	0.75 (0.47, 0.91)	0.16	1.00	0.55	99.7	
Gender						< 0.001
Male	0.53 (0.44, 0.63)	0.19	0.95	0.23	97.2	
Female	0.56 (0.48, 0.64)	0.23	0.94	0.19	96.1	
Other	0.70 (0.33, 0.92)	0.18	1.00	0.66	99.7	
Marital status						< 0.001
Married	0.56 (0.48, 0.65)	0.20	0.94	0.21	96.7	
Separated	0.53 (0.41, 0.65)	0.15	0.99	0.30	98.3	
Divorced	0.56 (0.45, 0.66)	0.23	0.99	0.26	97.9	
Widowed	0.52 (0.41, 0.63)	0.05	0.93	0.26	97.9	
Domestic partner	0.43 (0.36, 0.50)	0.27	0.82	0.16	94.8	
Single, never married	0.53 (0.44, 0.62)	0.22	0.96	0.22	97.0	
Employment status						< 0.001
Employed for an employer	0.55 (0.46, 0.64)	0.22	0.95	0.21	96.7	
Self-employed	0.56 (0.47, 0.64)	0.23	0.95	0.20	96.6	
Retired	0.54 (0.44, 0.64)	0.16	0.94	0.23	97.3	
Student	0.53 (0.43, 0.62)	0.24	0.96	0.22	97.1	
Homemaker	0.58 (0.50, 0.66)	0.23	0.94	0.19	96.0	
Unemployed & seeking jobs	0.54 (0.45, 0.62)	0.21	0.96	0.21	96.7	
None of these/other	0.55 (0.46, 0.64)	0.18	0.94	0.22	97.0	
Years of education						< 0.001
Up to 8 years	0.55 (0.47, 0.63)	0.23	0.93	0.19	96.2	
9–15 years	0.55 (0.46, 0.64)	0.21	0.96	0.22	97.0	
16 + years	0.56 (0.46, 0.66)	0.21	0.97	0.25	97.6	
Frequency of religious service attendance						< 0.001
> 1/week	0.78 (0.71, 0.84)	0.44	0.97	0.16	96.8	
1/week	0.73 (0.66, 0.78)	0.50	0.94	0.13	94.4	
1–3/month	0.63 (0.57, 0.70)	0.30	0.93	0.15	94.5	
A few times a year	0.53 (0.45, 0.61)	0.26	0.94	0.18	95.7	
Never	0.38 (0.30, 0.46)	0.18	0.86	0.19	96.2	
Immigration status						< 0.001
Born in this country	0.55 (0.46, 0.63)	0.21	0.95	0.21	96.7	
Born in another country	0.54 (0.45, 0.62)	0.15	0.88	0.20	96.5	
Overall	0.54 (0.47, 0.60)	0.21	0.95	0.17	99.8	

*CI * confidence interval.

τ is the standard deviation of the distribution of means across countries, an indicator of cross-national heterogeneity. *I*^2^ is an estimate of the variability in means due to heterogeneity across countries vs. sampling variability. Global *p*-value corresponds to a test of the null hypothesis that there are no differences between the groups for that sociodemographic characteristic in any of the 22 countries. All *p*-values passed the Bonferroni-corrected threshold of *p* < .007 (*p* = .05/7).

### Methods

The description of the methods below have been adapted from a team publication^29^. Further methodological detail is available elsewhere^28,30–35^.

## Data

The Global Flourishing Study (GFS) is a study of 202,898 participants from 22 geographically and culturally diverse countries, with nationally representative sampling within each country, concerning the distribution of determinants of well-being. Wave 1 of the data included the following countries and territories: Argentina, Australia, Brazil, Egypt, Germany, Hong Kong (S.A.R of China, with mainland China also included from 2024 onwards), India, Indonesia, Israel, Japan, Kenya, Mexico, Nigeria, the Philippines, Poland, South Africa, Spain, Sweden, Tanzania, Turkey, United Kingdom, and the United States. The countries were selected to (a) maximize coverage of the world’s population, (b) ensure geographic, cultural, and religious diversity, and (c) prioritize feasibility and existing data collection infrastructure. Data collection was carried out by Gallup Inc. Data for Wave 1 were collected principally during 2023, with some countries beginning data collection in 2022 and exact dates varying by country^28^. Plans are in place to collect four additional waves of annual panel data on the participants from 2024 to 2027. The precise sampling design to ensure nationally representative samples varied by country and further details are available^28^. Survey items included aspects of well-being such as happiness, health, meaning, character, relationships, and financial stability^36^, along with other demographic, social, economic, political, religious, personality, childhood, community, health, and well-being variables. The data are publicly available through the Center for Open Science (https://www.cos.io/gfs). During the translation process, Gallup adhered to TRAPD model (translation, review, adjudication, pretesting, and documentation) for cross-cultural survey research (ccsg.isr.umich.edu/chapters/translation/overview).

## Measures

*Demographics Variables*: Continuous age was classified as 18–24, 25–29, 30–39, 40–49, 50–59, 60–69, 70–79, and 80 or older. Gender was assessed as male, female, or other. Marital status was assessed as single/never married, married, separated, divorced, widowed, and domestic partner. Employment was assessed as employed, self-employed, retired, student, homemaker, unemployed and searching, and other. Education was assessed as up to 8 years, 9–15 years, and 16 + years. Service attendance was assessed as more than once/week, once/week, one-to-three times/month, a few times/year, or never. Immigration status was dichotomously assessed with: “Were you born in this country, or not?” Religious tradition/affiliation with categories of Christianity, Islam, Hinduism, Buddhism, Judaism, Sikhism, Baha’i, Jainism, Shinto, Taoism, Confucianism, Primal/Animist/Folk religion, Spiritism, African-Derived, some other religion, or no religion/atheist/agnostic; precise response categories varied by country (Johnson et al., 2023). Racial/ethnic identity was assessed in some, but not all, countries, with response categories varying by country. For additional details on the assessments see the COS GFS codebook^30,31^.

*Outcome Variable*: The afterlife belief measure comes from the following question: “I believe in life after death.” Respondents could answer Yes, No, or Unsure. This variable was dichotomized as Yes (1) vs. No/Unsure (0). As a post-hoc sensitivity analysis we will also report country means in the Online Supplement with the alternative dichotomization Yes/Unsure (1) vs. No (0), and furthermore report the absolute proportions in each country of “Unsure” responses.

### Analysis

Descriptive statistics for the full sample, weighted to be nationally representative within each country, were estimated for each of the demographic variables. Nationally representative proportions for afterlife belief were estimated separately for each country and ordered from highest to lowest along, with 95% confidence intervals and standard deviations for each. Variation in proportions in afterlife belief across demographic categories were estimated, with all analyses initially conducted by country. Primary results consisted of a random effects meta-analyses of country-specific proportion of afterlife belief in each specific demographic category^37,38^ along with 95% confidence intervals, standard errors, lower and upper limits of a prediction interval across countries, heterogeneity (τ), and I^2^ for evidence concerning variation within a particular demographic variable across countries^39^. All meta-analyses were conducted in **R**^40^ using the metafor package^41^. Within each country, a global test of variation of outcome across levels of each particular demographic variable was conducted, and a pooled p-value^42^ across countries reported concerning evidence for variation within any country. Bonferroni corrected p-value thresholds are provided based on the seven sociodemographic variables (i.e., age, gender, marital status, employment status, education status, frequency of religious service attendance, and immigration status) that were included in the meta-analyses: *p* = .05/7 = 0.007^43^. Religious affiliation/tradition and race/ethnicity were used, when available, as control variables within country, but were not included in the meta-analyses since the availability of these response categories varied by country. As a supplementary analysis, population weighted meta-analyses were also conducted. All analyses were pre-registered with COS prior to data access (https://osf.io/6qyzh); all code to reproduce analyses are openly available in an online repository.

### Missing data

Missing data on all variables was imputed using multivariate imputation by chained equations, and five imputed datasets were used^44,45^. To account for variation in the assessment of certain variables across countries (e.g., religious affiliation/tradition and race/ethnicity), the imputation process was conducted separately in each country. This within-country imputation approach ensured that the imputation models accurately reflected country-specific contexts and assessment methods. Sampling weights were included in the imputation models to account for specific-variable missingness that may have been related to probability of inclusion in the study.

### Accounting for complex sampling design

The GFS used different sampling scheme across countries based on availability of existing panels and recruitment needs^28^. All analyses accounted for the complex survey design components by including weights, primary sampling units, and strata. Additional methodological detail, including accounting for the complex sampling design, is provided elsewhere^32^.

## Electronic Supplementary Material

Below is the link to the electronic supplementary material.

Supplementary Material 1

## Data Availability

The data that support the findings of this article are openly available on the Open Science Framework^35^. The specific dataset used was: Wave 1 non-sensitive Global data https://osf.io/sm4cd/ available February 2024 - March 2026 via preregistration and publicly from then onwards. The research questions, variables, and analytic plan for the current study were preregistered with the Center for Open Science prior to accessing data (https://osf.io/6qyzh). All code to reproduce analyses are openly available in an online repository^33^.
