# Supplementary material for: Sociodemographic variations of belief in life after death across 22 Countries
Source: Sci Rep. 2025 Apr 30;15:14310. doi: 10.1038/s41598-024-83541-x (PMC12043894; doi:10.1038/s41598-024-83541-x)
Supplement: Supplementary file 1 — Supplementary Material 1 [file 41598_2024_83541_MOESM1_ESM.pdf]

Table S1a. Nationally representative descriptive statistics for Argentina

Characteristic	N = 6,724[1]
**Age group**	
18-24	1,108 (16%)
25-29	719 (11%)
30-39	1,432 (21%)
40-49	1,254 (19%)
50-59	1,014 (15%)
60-69	730 (11%)
70-79	356 (5.3%)
80 or older	112 (1.7%)
Missing	0 (0%)
**Gender**	
Male	3,143 (47%)
Female	3,542 (53%)
Other	21 (0.3%)
Missing	18 (0.3%)
**Marital status**	
Married	1,565 (23%)
Separated	455 (6.8%)
Divorced	321 (4.8%)
Widowed	401 (6.0%)
Never	2,381 (35%)
Domestic Partner	1,514 (23%)
Missing	88 (1.3%)
**Employment**	
Employed for an employer	2,440 (36%)
Self-employed	1,748 (26%)
Retired	773 (11%)
Student	354 (5.3%)
Homemaker	639 (9.5%)
Unemployed and looking for a job	569 (8.5%)
None of these/other	179 (2.7%)
Missing	22 (0.3%)
**Religious service attendance**	
>1/week	532 (7.9%)
1/week	773 (12%)
1-3/month	461 (6.8%)
A few times a year	1,949 (29%)
Never	2,982 (44%)
Missing	27 (0.4%)
**Education**	
up to 8 years	2,263 (34%)
9-15 years	3,823 (57%)
16+years	635 (9.4%)
Missing	3 (<0.1%)
**Immigration**	
Born in this country	6,346 (94%)
Born in another country	348 (5.2%)
Missing	29 (0.4%)
**Religious affiliation**	

Characteristic	N = 6,724[1]
Christianity	4,992 (74%)
Islam	9 (0.1%)
Hinduism	6 (<0.1%)
Buddhism	35 (0.5%)
Judaism	40 (0.6%)
Sikhism	0 (<0.1%)
Taoism	2 (<0.1%)
Confucianism	0 (<0.1%)
Primal, Animist, or Folk religion	19 (0.3%)
Some other religion	156 (2.3%)
No religion/Atheist/Agnostic	1,352 (20%)
Missing	111 (1.7%)
**Race and ethnicity**	
Asian	43 (0.6%)
Black	95 (1.4%)
Indigenous	129 (1.9%)
Mestizo(a)	1,801 (27%)
Mullato(a)	75 (1.1%)
Other	104 (1.5%)
White	3,406 (51%)
Missing	1,070 (16%)

[1]n (%)

Table S1b. Means by demographic category for Argentina

Variable	Category	Mean	SE	95% CI	Global p-value
Age group	18-24	0.56	0.02	(0.52, 0.60)	0.050
	25-29	0.60	0.03	(0.55, 0.65)	
	30-39	0.62	0.02	(0.58, 0.65)	
	40-49	0.58	0.02	(0.54, 0.61)	
	50-59	0.59	0.02	(0.54, 0.63)	
	60-69	0.57	0.03	(0.52, 0.62)	
	70-79	0.51	0.04	(0.43, 0.59)	
	80 or older	0.49	0.07	(0.34, 0.64)	
Gender	Male	0.54	0.01	(0.51, 0.56)	<0.001
	Female	0.62	0.01	(0.59, 0.64)	
	Other	0.52	0.15	(0.20, 0.83)	
Marital status	Married	0.63	0.02	(0.59, 0.66)	<0.001
	Separated	0.56	0.03	(0.50, 0.63)	
	Divorced	0.57	0.04	(0.50, 0.63)	
	Widowed	0.51	0.04	(0.43, 0.59)	
	Never	0.57	0.01	(0.54, 0.59)	
	Domestic Partner	0.58	0.02	(0.54, 0.61)	
Employment	Employed for an employer	0.59	0.01	(0.56, 0.61)	0.993
	Self-employed	0.57	0.02	(0.54, 0.61)	
	Retired	0.55	0.03	(0.50, 0.60)	
	Student	0.58	0.03	(0.51, 0.64)	
	Homemaker	0.60	0.03	(0.54, 0.66)	
	Unemployed and looking for a job	0.60	0.03	(0.54, 0.65)	
	None of these/other	0.58	0.05	(0.48, 0.68)	
Religious service attendance	>1/week	0.76	0.03	(0.70, 0.81)	<0.001
	1/week	0.76	0.02	(0.71, 0.80)	
	1-3/month	0.61	0.03	(0.54, 0.67)	
	A few times a year	0.60	0.02	(0.57, 0.63)	
	Never	0.48	0.01	(0.46, 0.51)	
Education	up to 8 years	0.56	0.02	(0.52, 0.60)	<0.001

Variable	Category	Mean	SE	95% CI	Global p-value
	9-15 years	0.60	0.01	(0.58, 0.62)	
	16+years	0.53	0.02	(0.49, 0.57)	
Immigration status	Born in this country	0.58	0.01	(0.56, 0.60)	0.534
	Born in another country	0.55	0.04	(0.47, 0.63)	
Religious affiliation	Christianity	0.63	0.01	(0.61, 0.65)	<0.001
	Islam	0.63	0.23	(0.08, 1.19)	
	Hinduism	0.08	0.09	(-0.16, 0.32)	
	Buddhism	0.67	0.11	(0.44, 0.90)	
	Judaism	0.42	0.12	(0.17, 0.67)	
	Taoism	0.31			
	Primal, Animist, or Folk religion	0.78	0.12	(0.53, 1.03)	
	Some other religion	0.59	0.06	(0.46, 0.71)	
	No religion/Atheist/Agnostic	0.40	0.02	(0.37, 0.44)	
Race and ethnicity	Asian	0.70	0.10	(0.50, 0.90)	0.444
	Black	0.59	0.08	(0.42, 0.75)	
	Indigenous	0.60	0.06	(0.47, 0.73)	
	Mestizo(a)	0.60	0.02	(0.57, 0.63)	
	Mullato(a)	0.63	0.08	(0.45, 0.80)	
	Other	0.62	0.08	(0.46, 0.78)	
	White	0.57	0.01	(0.54, 0.59)	

Table S2a. Nationally representative descriptive statistics for Australia

Characteristic	N = 3,844[1]
**Age group**	
18-24	345 (9.0%)
25-29	282 (7.3%)
30-39	641 (17%)
40-49	618 (16%)
50-59	691 (18%)
60-69	589 (15%)
70-79	498 (13%)
80 or older	178 (4.6%)
Missing	2 (<0.1%)
**Gender**	
Male	1,861 (48%)
Female	1,941 (50%)
Other	36 (0.9%)
Missing	6 (0.2%)
**Marital status**	
Married	1,797 (47%)
Separated	158 (4.1%)
Divorced	332 (8.6%)
Widowed	215 (5.6%)
Never	855 (22%)
Domestic Partner	450 (12%)
Missing	38 (1.0%)
**Employment**	
Employed for an employer	1,881 (49%)
Self-employed	380 (9.9%)
Retired	912 (24%)
Student	190 (5.0%)
Homemaker	137 (3.6%)
Unemployed and looking for a job	134 (3.5%)
None of these/other	206 (5.4%)
Missing	4 (0.1%)
**Religious service attendance**	
>1/week	162 (4.2%)
1/week	299 (7.8%)
1-3/month	135 (3.5%)
A few times a year	656 (17%)
Never	2,584 (67%)
Missing	7 (0.2%)
**Education**	
up to 8 years	70 (1.8%)
9-15 years	2,434 (63%)
16+years	1,330 (35%)
Missing	10 (0.3%)
**Immigration**	
Born in this country	2,953 (77%)
Born in another country	885 (23%)
Missing	6 (0.2%)
**Religious affiliation**	

Characteristic	N = 3,844[1]
Christianity	1,592 (41%)
Islam	45 (1.2%)
Hinduism	31 (0.8%)
Buddhism	36 (0.9%)
Judaism	26 (0.7%)
Sikhism	8 (0.2%)
Baha’i	7 (0.2%)
Taoism	5 (0.1%)
Primal, Animist, or Folk religion	23 (0.6%)
Some other religion	39 (1.0%)
No religion/Atheist/Agnostic	2,020 (53%)
Missing	15 (0.4%)
**Race and ethnicity**	
Aboriginal	53 (1.4%)
Australian	1,946 (51%)
Australian British/European	1,047 (27%)
Chinese	75 (1.9%)
Indian	58 (1.5%)
Japanese	1 (<0.1%)
Malay	11 (0.3%)
New Zealander	91 (2.4%)
Other	163 (4.2%)
Other European	357 (9.3%)
Russian	7 (0.2%)
Samoan	4 (0.1%)
Sinhalese	1 (<0.1%)
Spanish	2 (<0.1%)
Sri Lankan Moor	1 (<0.1%)
Sri Lankan Tamil	7 (0.2%)
Vietnamese	7 (0.2%)
Missing	14 (0.4%)

[1]n (%)

Table S2b. Means by demographic category for Australia

Variable	Category	Mean	SE	95% CI	Global p-value
Age group	18-24	0.34	0.04	(0.27, 0.42)	<0.001
	25-29	0.35	0.04	(0.27, 0.44)	
	30-39	0.30	0.03	(0.25, 0.35)	
	40-49	0.42	0.03	(0.37, 0.47)	
	50-59	0.37	0.02	(0.33, 0.41)	
	60-69	0.35	0.02	(0.31, 0.39)	
	70-79	0.36	0.03	(0.31, 0.41)	
	80 or older	0.44	0.04	(0.36, 0.53)	
Gender	Male	0.28	0.01	(0.25, 0.30)	<0.001
	Female	0.45	0.01	(0.42, 0.47)	
	Other	0.41	0.12	(0.17, 0.66)	
Marital status	Married	0.38	0.01	(0.36, 0.41)	<0.001
	Separated	0.38	0.05	(0.28, 0.48)	
	Divorced	0.39	0.03	(0.32, 0.45)	
	Widowed	0.44	0.04	(0.36, 0.52)	
	Never	0.34	0.02	(0.30, 0.39)	
	Domestic Partner	0.27	0.03	(0.21, 0.33)	
Employment	Employed for an employer	0.35	0.01	(0.32, 0.37)	<0.001
	Self-employed	0.36	0.03	(0.30, 0.42)	
	Retired	0.36	0.02	(0.33, 0.40)	
	Student	0.30	0.05	(0.20, 0.40)	
	Homemaker	0.50	0.06	(0.38, 0.62)	
	Unemployed and looking for a job	0.41	0.06	(0.30, 0.53)	
	None of these/other	0.47	0.05	(0.37, 0.56)	
Religious service attendance	>1/week	0.95	0.02	(0.91, 0.99)	<0.001
	1/week	0.83	0.03	(0.78, 0.88)	
	1-3/month	0.74	0.05	(0.64, 0.83)	
	A few times a year	0.48	0.02	(0.43, 0.53)	
	Never	0.22	0.01	(0.20, 0.25)	
Education	up to 8 years	0.40	0.09	(0.22, 0.57)	0.149

Variable	Category	Mean	SE	95% CI	Global p-value
	9-15 years	0.37	0.01	(0.35, 0.40)	
	16+years	0.34	0.01	(0.32, 0.37)	
Immigration status	Born in this country	0.36	0.01	(0.33, 0.38)	0.010
	Born in another country	0.39	0.02	(0.35, 0.43)	
Religious affiliation	Christianity	0.59	0.01	(0.56, 0.61)	<0.001
	Islam	0.83	0.08	(0.68, 0.99)	
	Hinduism	0.51	0.12	(0.27, 0.75)	
	Buddhism	0.69	0.08	(0.53, 0.85)	
	Judaism	0.22	0.09	(0.04, 0.40)	
	Sikhism	0.00			
	Baha'i	0.86	0.12	(0.55, 1.17)	
	Taoism	0.80	0.17	(0.17, 1.42)	
	Primal, Animist, or Folk religion	0.69	0.14	(0.39, 0.99)	
	Some other religion	0.55	0.11	(0.32, 0.77)	
	No religion/Atheist/Agnostic	0.16	0.01	(0.14, 0.19)	
Race and ethnicity	Other	0.49	0.05	(0.39, 0.59)	<0.001
	Aboriginal	0.49	0.10	(0.29, 0.69)	
	Australian	0.38	0.01	(0.35, 0.40)	
	Australian British/European	0.30	0.02	(0.26, 0.33)	
	Chinese	0.35	0.08	(0.20, 0.50)	
	Indian	0.49	0.08	(0.32, 0.66)	
	Japanese	0.54			
	Malay	0.31	0.24	(-0.32, 0.94)	
	New Zealander	0.46	0.07	(0.32, 0.60)	
	Other European	0.36	0.03	(0.30, 0.42)	
	Russian	0.33	0.20	(-0.19, 0.85)	
	Samoan	1.00			
	Sinhalese	0.39			
	Spanish	0.34			
	Sri Lankan Moor	0.72			
	Sri Lankan Tamil	0.64	0.26	(-0.04, 1.33)	

Variable	Category	Mean	SE	95% CI	Global p-value
	Vietnamese	0.26	0.22	(-0.31, 0.83)	

Table S3a. Nationally representative descriptive statistics for Brazil

Characteristic	N = 13,204[1]
**Age group**	
18-24	1,986 (15%)
25-29	1,468 (11%)
30-39	2,908 (22%)
40-49	2,638 (20%)
50-59	2,131 (16%)
60-69	1,435 (11%)
70-79	510 (3.9%)
80 or older	126 (1.0%)
Missing	0 (0%)
**Gender**	
Male	6,320 (48%)
Female	6,820 (52%)
Other	35 (0.3%)
Missing	30 (0.2%)
**Marital status**	
Married	4,646 (35%)
Separated	594 (4.5%)
Divorced	865 (6.5%)
Widowed	408 (3.1%)
Never	4,347 (33%)
Domestic Partner	2,081 (16%)
Missing	263 (2.0%)
**Employment**	
Employed for an employer	3,756 (28%)
Self-employed	2,918 (22%)
Retired	1,536 (12%)
Student	624 (4.7%)
Homemaker	1,305 (9.9%)
Unemployed and looking for a job	2,419 (18%)
None of these/other	448 (3.4%)
Missing	199 (1.5%)
**Religious service attendance**	
>1/week	2,386 (18%)
1/week	2,272 (17%)
1-3/month	1,398 (11%)
A few times a year	3,978 (30%)
Never	3,110 (24%)
Missing	61 (0.5%)
**Education**	
up to 8 years	3,139 (24%)
9-15 years	7,665 (58%)
16+years	2,390 (18%)
Missing	10 (<0.1%)
**Immigration**	
Born in this country	12,688 (96%)
Born in another country	153 (1.2%)
Missing	363 (2.7%)
**Religious affiliation**	

Characteristic	N = 13,204[1]
Christianity	9,911 (75%)
Islam	6 (<0.1%)
Hinduism	1 (<0.1%)
Buddhism	37 (0.3%)
Judaism	31 (0.2%)
Baha’i	2 (<0.1%)
Jainism	2 (<0.1%)
Shinto	1 (<0.1%)
Taoism	1 (<0.1%)
Confucianism	6 (<0.1%)
Primal, Animist, or Folk religion	15 (0.1%)
Spiritism	696 (5.3%)
Umbanda, Candomblé, and other African-derived religions	525 (4.0%)
Some other religion	144 (1.1%)
No religion/Atheist/Agnostic	1,712 (13%)
Missing	113 (0.9%)
**Race and ethnicity**	
Amarela	238 (1.8%)
Branca	5,169 (39%)
Indígena	131 (1.0%)
Other	61 (0.5%)
Parda	5,125 (39%)
Preta	1,615 (12%)
Missing	865 (6.6%)

[1]n (%)

Table S3b. Means by demographic category for Brazil

Variable	Category	Mean	SE	95% CI	Global p-value
Age group	18-24	0.57	0.01	(0.54, 0.59)	<0.001
	25-29	0.58	0.02	(0.55, 0.61)	
	30-39	0.60	0.01	(0.58, 0.62)	
	40-49	0.62	0.01	(0.60, 0.65)	
	50-59	0.66	0.01	(0.64, 0.69)	
	60-69	0.63	0.02	(0.58, 0.67)	
	70-79	0.56	0.04	(0.48, 0.64)	
	80 or older	0.60	0.07	(0.46, 0.74)	
Gender	Male	0.62	0.01	(0.60, 0.63)	0.032
	Female	0.60	0.01	(0.59, 0.62)	
	Other	0.47	0.10	(0.26, 0.67)	
Marital status	Married	0.60	0.01	(0.58, 0.62)	0.004
	Separated	0.65	0.03	(0.60, 0.71)	
	Divorced	0.64	0.02	(0.60, 0.69)	
	Widowed	0.58	0.04	(0.50, 0.65)	
	Never	0.60	0.01	(0.58, 0.62)	
	Domestic Partner	0.63	0.01	(0.60, 0.66)	
Employment	Employed for an employer	0.63	0.01	(0.61, 0.65)	<0.001
	Self-employed	0.62	0.01	(0.59, 0.64)	
	Retired	0.60	0.02	(0.56, 0.64)	
	Student	0.57	0.02	(0.52, 0.62)	
	Homemaker	0.56	0.02	(0.53, 0.60)	
	Unemployed and looking for a job	0.60	0.01	(0.58, 0.63)	
	None of these/other	0.62	0.03	(0.56, 0.68)	
Religious service attendance	>1/week	0.63	0.01	(0.60, 0.65)	<0.001
	1/week	0.67	0.01	(0.64, 0.69)	
	1-3/month	0.65	0.02	(0.62, 0.68)	
	A few times a year	0.62	0.01	(0.60, 0.64)	
	Never	0.52	0.01	(0.50, 0.54)	
Education	up to 8 years	0.59	0.01	(0.56, 0.61)	<0.001

Variable	Category	Mean	SE	95% CI	Global p-value
	9-15 years	0.60	0.01	(0.59, 0.62)	
	16+years	0.66	0.01	(0.63, 0.68)	
Immigration status	Born in this country	0.61	0.01	(0.60, 0.62)	0.693
	Born in another country	0.57	0.06	(0.46, 0.68)	
Religious affiliation	Christianity	0.60	0.01	(0.58, 0.61)	<0.001
	Islam	0.65	0.16	(0.21, 1.10)	
	Hinduism	0.73			
	Buddhism	0.79	0.10	(0.59, 1.00)	
	Judaism	0.62	0.13	(0.35, 0.89)	
	Baha'i	0.83			
	Jainism	0.19			
	Shinto	1.00			
	Taoism	1.00			
	Confucianism	0.13	0.11	(-0.19, 0.45)	
	Primal, Animist, or Folk religion	0.73	0.14	(0.42, 1.05)	
	Spiritism	0.93	0.01	(0.91, 0.95)	
	Umbanda, Candomblé, and other African-derived religions	0.87	0.02	(0.84, 0.91)	
	Some other religion	0.82	0.04	(0.74, 0.91)	
	No religion/Atheist/Agnostic	0.45	0.02	(0.42, 0.48)	
Race and ethnicity	Other	0.72	0.08	(0.56, 0.89)	0.004
	Amarela	0.59	0.04	(0.51, 0.66)	
	Branca	0.62	0.01	(0.61, 0.64)	
	Indígena	0.61	0.06	(0.50, 0.72)	
	Parda	0.60	0.01	(0.59, 0.62)	
	Preta	0.58	0.02	(0.55, 0.62)	

Table S4a. Nationally representative descriptive statistics for Egypt

Characteristic	N = 4,729[1]
**Age group**	
18-24	960 (20%)
25-29	607 (13%)
30-39	1,204 (25%)
40-49	897 (19%)
50-59	613 (13%)
60-69	387 (8.2%)
70-79	54 (1.1%)
80 or older	7 (0.2%)
Missing	0 (0%)
**Gender**	
Male	2,394 (51%)
Female	2,334 (49%)
Other	0 (0%)
Missing	0 (<0.1%)
**Marital status**	
Married	3,387 (72%)
Separated	39 (0.8%)
Divorced	101 (2.1%)
Widowed	238 (5.0%)
Never	947 (20%)
Domestic Partner	0 (0%)
Missing	17 (0.4%)
**Employment**	
Employed for an employer	1,267 (27%)
Self-employed	892 (19%)
Retired	253 (5.4%)
Student	297 (6.3%)
Homemaker	1,772 (37%)
Unemployed and looking for a job	224 (4.7%)
None of these/other	21 (0.4%)
Missing	3 (<0.1%)
**Religious service attendance**	
>1/week	839 (18%)
1/week	960 (20%)
1-3/month	368 (7.8%)
A few times a year	458 (9.7%)
Never	2,091 (44%)
Missing	12 (0.3%)
**Education**	
up to 8 years	2,486 (53%)
9-15 years	1,599 (34%)
16+years	643 (14%)
Missing	1 (<0.1%)
**Immigration**	
Born in this country	4,713 (100%)
Born in another country	16 (0.3%)
Missing	1 (<0.1%)
**Religious affiliation**	

**Characteristic**	**N = 4,729**[1]
Christianity	120 (2.5%)
Islam	4,607 (97%)
Taoism	0 (<0.1%)
Missing	1 (<0.1%)
**Race and ethnicity**	
Arab	4,585 (97%)
Bedouin Arab	4 (<0.1%)
Greek	1 (<0.1%)
Nubian	27 (0.6%)
Turkish	9 (0.2%)
Missing	102 (2.2%)

[1]n (%)

Table S4b. Means by demographic category for Egypt

Variable	Category	Mean	SE	95% CI	Global p-value
Age group	18-24	0.63	0.02	(0.58, 0.67)	<0.001
	25-29	0.60	0.03	(0.55, 0.65)	
	30-39	0.65	0.02	(0.62, 0.68)	
	40-49	0.65	0.02	(0.62, 0.69)	
	50-59	0.65	0.02	(0.61, 0.69)	
	60-69	0.66	0.03	(0.60, 0.73)	
	70-79	0.77	0.07	(0.63, 0.91)	
	80 or older	1.00			
Gender	Male	0.69	0.01	(0.67, 0.72)	<0.001
	Female	0.59	0.01	(0.57, 0.62)	
Marital status	Married	0.64	0.01	(0.62, 0.66)	1.000
	Separated	0.65	0.08	(0.49, 0.81)	
	Divorced	0.64	0.06	(0.53, 0.75)	
	Widowed	0.62	0.04	(0.54, 0.69)	
	Never	0.65	0.02	(0.61, 0.69)	
Employment	Employed for an employer	0.68	0.02	(0.65, 0.71)	<0.001
	Self-employed	0.69	0.02	(0.65, 0.73)	
	Retired	0.66	0.04	(0.58, 0.75)	
	Student	0.68	0.04	(0.61, 0.75)	
	Homemaker	0.58	0.01	(0.55, 0.61)	
	Unemployed and looking for a job	0.67	0.04	(0.60, 0.75)	
	None of these/other	0.78	0.12	(0.53, 1.04)	
Religious service attendance	>1/week	0.65	0.02	(0.61, 0.69)	0.031
	1/week	0.68	0.02	(0.64, 0.72)	
	1-3/month	0.63	0.03	(0.56, 0.70)	
	A few times a year	0.65	0.02	(0.60, 0.69)	
	Never	0.62	0.01	(0.60, 0.65)	
Education	up to 8 years	0.59	0.01	(0.57, 0.62)	<0.001
	9-15 years	0.66	0.01	(0.64, 0.69)	
	16+years	0.79	0.02	(0.74, 0.83)	

Variable	Category	Mean	SE	95% CI	Global p-value
Immigration status	Born in this country	0.64	0.01	(0.62, 0.66)	<0.001
	Born in another country	0.89	0.11	(0.65, 1.12)	
Religious affiliation	Christianity	0.72	0.04	(0.63, 0.80)	0.009
	Islam	0.64	0.01	(0.62, 0.66)	
Race and ethnicity	Arab	0.64	0.01	(0.62, 0.66)	<0.001
	Bedouin Arab	1.00			
	Nubian	0.55	0.05	(0.45, 0.65)	
	Turkish	0.82	0.11	(0.56, 1.08)	

Table S5a. Nationally representative descriptive statistics for Germany

Characteristic	N = 9,506[1]
**Age group**	
18-24	829 (8.7%)
25-29	774 (8.1%)
30-39	1,438 (15%)
40-49	1,494 (16%)
50-59	1,729 (18%)
60-69	1,915 (20%)
70-79	1,137 (12%)
80 or older	190 (2.0%)
Missing	0 (0%)
**Gender**	
Male	4,641 (49%)
Female	4,843 (51%)
Other	11 (0.1%)
Missing	11 (0.1%)
**Marital status**	
Married	4,784 (50%)
Separated	219 (2.3%)
Divorced	767 (8.1%)
Widowed	409 (4.3%)
Never	2,627 (28%)
Domestic Partner	619 (6.5%)
Missing	81 (0.9%)
**Employment**	
Employed for an employer	4,950 (52%)
Self-employed	712 (7.5%)
Retired	2,480 (26%)
Student	605 (6.4%)
Homemaker	251 (2.6%)
Unemployed and looking for a job	288 (3.0%)
None of these/other	204 (2.1%)
Missing	14 (0.2%)
**Religious service attendance**	
>1/week	285 (3.0%)
1/week	424 (4.5%)
1-3/month	550 (5.8%)
A few times a year	2,362 (25%)
Never	5,876 (62%)
Missing	9 (<0.1%)
**Education**	
up to 8 years	235 (2.5%)
9-15 years	6,094 (64%)
16+years	3,164 (33%)
Missing	13 (0.1%)
**Immigration**	
Born in this country	8,722 (92%)
Born in another country	744 (7.8%)
Missing	40 (0.4%)
**Religious affiliation**	

**Characteristic**	**N = 9,506**[1]
Christianity	5,052 (53%)
Islam	351 (3.7%)
Hinduism	12 (0.1%)
Buddhism	51 (0.5%)
Judaism	19 (0.2%)
Sikhism	5 (<0.1%)
Baha'i	3 (<0.1%)
Shinto	2 (<0.1%)
Taoism	0 (<0.1%)
Confucianism	4 (<0.1%)
Primal, Animist, or Folk religion	34 (0.4%)
Some other religion	60 (0.6%)
No religion/Atheist/Agnostic	3,815 (40%)
Missing	99 (1.0%)
**Race and ethnicity**	
Missing	9,506 (100%)

[1]n (%)

Table S5b. Means by demographic category for Germany

Variable	Category	Mean	SE	95% CI	Global p-value
Age group	18-24	0.38	0.02	(0.33, 0.42)	<0.001
	25-29	0.38	0.02	(0.33, 0.42)	
	30-39	0.37	0.02	(0.34, 0.39)	
	40-49	0.37	0.02	(0.34, 0.40)	
	50-59	0.34	0.01	(0.31, 0.37)	
	60-69	0.31	0.01	(0.28, 0.34)	
	70-79	0.29	0.02	(0.25, 0.33)	
	80 or older	0.32	0.04	(0.23, 0.41)	
Gender	Male	0.33	0.01	(0.31, 0.35)	0.052
	Female	0.36	0.01	(0.34, 0.37)	
	Other	0.40	0.19	(-0.04, 0.83)	
Marital status	Married	0.33	0.01	(0.32, 0.35)	0.774
	Separated	0.36	0.04	(0.28, 0.44)	
	Divorced	0.37	0.02	(0.33, 0.41)	
	Widowed	0.33	0.03	(0.27, 0.39)	
	Never	0.36	0.01	(0.33, 0.38)	
	Domestic Partner	0.34	0.02	(0.29, 0.39)	
Employment	Employed for an employer	0.35	0.01	(0.33, 0.37)	<0.001
	Self-employed	0.36	0.02	(0.32, 0.41)	
	Retired	0.31	0.01	(0.29, 0.34)	
	Student	0.34	0.03	(0.28, 0.39)	
	Homemaker	0.43	0.04	(0.35, 0.50)	
	Unemployed and looking for a job	0.37	0.03	(0.30, 0.43)	
	None of these/other	0.41	0.04	(0.33, 0.49)	
Religious service attendance	>1/week	0.64	0.04	(0.57, 0.71)	<0.001
	1/week	0.68	0.03	(0.62, 0.73)	
	1-3/month	0.57	0.03	(0.51, 0.63)	
	A few times a year	0.41	0.01	(0.39, 0.44)	
	Never	0.26	0.01	(0.24, 0.27)	
Education	up to 8 years	0.39	0.04	(0.31, 0.46)	0.671

Variable	Category	Mean	SE	95% CI	Global p-value
	9-15 years	0.34	0.01	(0.32, 0.36)	
	16+years	0.35	0.01	(0.33, 0.37)	
Immigration status	Born in this country	0.34	0.01	(0.33, 0.35)	0.035
	Born in another country	0.38	0.02	(0.33, 0.42)	
Religious affiliation	Christianity	0.42	0.01	(0.41, 0.44)	<0.001
	Islam	0.64	0.03	(0.57, 0.71)	
	Hinduism	0.41	0.19	(-0.02, 0.85)	
	Buddhism	0.68	0.08	(0.53, 0.84)	
	Judaism	0.24	0.12	(-0.02, 0.50)	
	Sikhism	0.59	0.26	(-0.24, 1.42)	
	Baha'i	0.71			
	Confucianism	0.00			
	Primal, Animist, or Folk religion	0.69	0.11	(0.46, 0.92)	
	Some other religion	0.70	0.09	(0.52, 0.88)	
	No religion/Atheist/Agnostic	0.20	0.01	(0.18, 0.22)	

Table S6a. Nationally representative descriptive statistics for Hong Kong

Characteristic	N = 3,012[1]
**Age group**	
18-24	217 (7.2%)
25-29	198 (6.6%)
30-39	507 (17%)
40-49	580 (19%)
50-59	711 (24%)
60-69	620 (21%)
70-79	164 (5.5%)
80 or older	15 (0.5%)
Missing	0 (0%)
**Gender**	
Male	1,390 (46%)
Female	1,620 (54%)
Other	2 (<0.1%)
Missing	0 (0%)
**Marital status**	
Married	2,080 (69%)
Separated	21 (0.7%)
Divorced	105 (3.5%)
Widowed	45 (1.5%)
Never	723 (24%)
Domestic Partner	37 (1.2%)
Missing	1 (<0.1%)
**Employment**	
Employed for an employer	2,056 (68%)
Self-employed	245 (8.1%)
Retired	423 (14%)
Student	55 (1.8%)
Homemaker	114 (3.8%)
Unemployed and looking for a job	62 (2.0%)
None of these/other	39 (1.3%)
Missing	18 (0.6%)
**Religious service attendance**	
>1/week	237 (7.9%)
1/week	567 (19%)
1-3/month	332 (11%)
A few times a year	543 (18%)
Never	1,332 (44%)
Missing	1 (<0.1%)
**Education**	
up to 8 years	433 (14%)
9-15 years	2,031 (67%)
16+years	547 (18%)
Missing	0 (0%)
**Immigration**	
Born in this country	2,637 (88%)
Born in another country	321 (11%)
Missing	53 (1.8%)
**Religious affiliation**	

**Characteristic**	**N = 3,012**[1]
Christianity	757 (25%)
Islam	86 (2.8%)
Hinduism	20 (0.7%)
Buddhism	349 (12%)
Judaism	10 (0.3%)
Sikhism	2 (<0.1%)
Baha'i	3 (<0.1%)
Jainism	1 (<0.1%)
Shinto	19 (0.6%)
Taoism	97 (3.2%)
Confucianism	11 (0.4%)
Primal, Animist, or Folk religion	27 (0.9%)
Chinese folk/traditional religion	106 (3.5%)
Some other religion	4 (0.1%)
No religion/Atheist/Agnostic	1,518 (50%)
Missing	5 (0.2%)
**Race and ethnicity**	
Chinese (Cantonese)	1,930 (64%)
Chinese (Chaoshan)	201 (6.7%)
Chinese (Fujianese)	117 (3.9%)
Chinese (Hakka)	121 (4.0%)
Chinese (Other ethnicity)	264 (8.8%)
Chinese (Shanghainese)	89 (2.9%)
East Asian (Korean, Japanese)	10 (0.3%)
Other	4 (0.1%)
South Asian (Indian, Nepalese, Pakistani)	17 (0.6%)
Southeast Asian (Filipino, Indonesian, Thailand)	46 (1.5%)
Taiwanese	14 (0.4%)
White	15 (0.5%)
Missing	184 (6.1%)

[1]n (%)

Table S6b. Means by demographic category for Hong Kong

Variable	Category	Mean	SE	95% CI	Global p-value
Age group	18-24	0.44	0.03	(0.38, 0.51)	<0.001
	25-29	0.50	0.04	(0.42, 0.59)	
	30-39	0.47	0.02	(0.42, 0.52)	
	40-49	0.55	0.02	(0.51, 0.60)	
	50-59	0.44	0.02	(0.39, 0.48)	
	60-69	0.39	0.04	(0.32, 0.46)	
	70-79	0.27	0.08	(0.12, 0.42)	
	80 or older	1.00			
Gender	Male	0.42	0.02	(0.39, 0.46)	0.002
	Female	0.48	0.02	(0.45, 0.52)	
	Other	0.55			
Marital status	Married	0.45	0.02	(0.42, 0.48)	<0.001
	Separated	0.39	0.22	(-0.06, 0.85)	
	Divorced	0.58	0.08	(0.43, 0.73)	
	Widowed	0.04	0.02	(-0.01, 0.09)	
	Never	0.49	0.02	(0.44, 0.53)	
	Domestic Partner	0.33	0.09	(0.15, 0.50)	
Employment	Employed for an employer	0.47	0.01	(0.44, 0.50)	<0.001
	Self-employed	0.60	0.04	(0.52, 0.67)	
	Retired	0.32	0.05	(0.23, 0.41)	
	Student	0.38	0.06	(0.25, 0.51)	
	Homemaker	0.47	0.07	(0.33, 0.62)	
	Unemployed and looking for a job	0.40	0.09	(0.22, 0.58)	
	None of these/other	0.26	0.12	(0.01, 0.51)	
Religious service attendance	>1/week	0.84	0.03	(0.77, 0.90)	<0.001
	1/week	0.63	0.03	(0.57, 0.69)	
	1-3/month	0.51	0.04	(0.43, 0.58)	
	A few times a year	0.47	0.03	(0.41, 0.53)	
	Never	0.29	0.02	(0.26, 0.32)	
Education	up to 8 years	0.48	0.05	(0.39, 0.57)	0.991

Variable	Category	Mean	SE	95% CI	Global p-value
	9-15 years	0.45	0.01	(0.42, 0.48)	
	16+years	0.45	0.03	(0.40, 0.50)	
Immigration status	Born in this country	0.45	0.01	(0.43, 0.48)	0.998
	Born in another country	0.46	0.05	(0.37, 0.56)	
Religious affiliation	Christianity	0.54	0.03	(0.49, 0.59)	<0.001
	Islam	0.84	0.04	(0.76, 0.93)	
	Hinduism	0.68	0.13	(0.40, 0.96)	
	Buddhism	0.73	0.03	(0.67, 0.80)	
	Judaism	0.37	0.14	(0.03, 0.70)	
	Sikhism	0.82			
	Baha'i	0.68			
	Shinto	0.93	0.05	(0.84, 1.03)	
	Taoism	0.55	0.08	(0.38, 0.71)	
	Confucianism	0.59	0.15	(0.24, 0.93)	
	Primal, Animist, or Folk religion	0.44	0.09	(0.25, 0.63)	
	Chinese folk/traditional religion	0.53	0.07	(0.39, 0.67)	
	Some other religion	0.41	0.32	(-1.31, 2.14)	
	No religion/Atheist/Agnostic	0.31	0.01	(0.28, 0.34)	
Race and ethnicity	Other	0.32	0.21	(-0.45, 1.09)	<0.001
	White	0.86	0.09	(0.68, 1.05)	
	Chinese (Cantonese)	0.42	0.01	(0.40, 0.45)	
	Chinese (Chaoshan)	0.50	0.04	(0.41, 0.58)	
	Chinese (Fujianese)	0.36	0.06	(0.25, 0.47)	
	Chinese (Hakka)	0.46	0.06	(0.34, 0.59)	
	Chinese (Other ethnicity)	0.57	0.04	(0.49, 0.66)	
	Chinese (Shanghainese)	0.46	0.09	(0.28, 0.65)	
	East Asian (Korean, Japanese)	0.68	0.21	(0.19, 1.16)	
	South Asian (Indian, Nepalese, Pakistani)	0.61	0.23	(0.12, 1.09)	
	Southeast Asian (Filipino, Indonesian, Thailand)	0.84	0.11	(0.63, 1.06)	
	Taiwanese	0.36	0.17	(-0.01, 0.72)	

Table S7a. Nationally representative descriptive statistics for India

Characteristic	N = 12,765[1]
**Age group**	
18-24	2,543 (20%)
25-29	1,640 (13%)
30-39	3,109 (24%)
40-49	2,275 (18%)
50-59	1,574 (12%)
60-69	1,188 (9.3%)
70-79	370 (2.9%)
80 or older	67 (0.5%)
Missing	0 (0%)
**Gender**	
Male	6,473 (51%)
Female	6,292 (49%)
Other	0 (0%)
Missing	0 (0%)
**Marital status**	
Married	9,848 (77%)
Separated	45 (0.4%)
Divorced	25 (0.2%)
Widowed	445 (3.5%)
Never	2,065 (16%)
Domestic Partner	269 (2.1%)
Missing	69 (0.5%)
**Employment**	
Employed for an employer	2,660 (21%)
Self-employed	3,401 (27%)
Retired	286 (2.2%)
Student	532 (4.2%)
Homemaker	4,221 (33%)
Unemployed and looking for a job	902 (7.1%)
None of these/other	715 (5.6%)
Missing	48 (0.4%)
**Religious service attendance**	
>1/week	2,875 (23%)
1/week	3,166 (25%)
1-3/month	2,740 (21%)
A few times a year	2,090 (16%)
Never	1,823 (14%)
Missing	71 (0.6%)
**Education**	
up to 8 years	11,422 (89%)
9-15 years	1,194 (9.4%)
16+years	145 (1.1%)
Missing	4 (<0.1%)
**Immigration**	
Born in this country	12,629 (99%)
Born in another country	110 (0.9%)
Missing	26 (0.2%)
**Religious affiliation**	

Characteristic	N = 12,765[1]
Christianity	306 (2.4%)
Islam	1,555 (12%)
Hinduism	10,362 (81%)
Buddhism	230 (1.8%)
Sikhism	127 (1.0%)
Jainism	10 (<0.1%)
Shinto	1 (<0.1%)
Primal, Animist, or Folk religion	30 (0.2%)
Some other religion	67 (0.5%)
No religion/Atheist/Agnostic	13 (0.1%)
Missing	62 (0.5%)
**Race and ethnicity**	
General	3,538 (28%)
Other backward caste	4,177 (33%)
Schedule caste	3,599 (28%)
Schedule tribe	1,185 (9.3%)
Missing	267 (2.1%)

[1]n (%)

Table S7b. Means by demographic category for India

Variable	Category	Mean	SE	95% CI	Global p-value
Age group	18-24	0.47	0.01	(0.44, 0.49)	0.692
	25-29	0.48	0.01	(0.45, 0.51)	
	30-39	0.48	0.01	(0.46, 0.50)	
	40-49	0.48	0.01	(0.45, 0.50)	
	50-59	0.50	0.02	(0.47, 0.53)	
	60-69	0.46	0.02	(0.42, 0.50)	
	70-79	0.53	0.04	(0.46, 0.60)	
	80 or older	0.47	0.08	(0.32, 0.62)	
Gender	Male	0.47	0.01	(0.45, 0.49)	0.092
	Female	0.49	0.01	(0.47, 0.50)	
Marital status	Married	0.50	0.01	(0.49, 0.51)	<0.001
	Separated	0.37	0.09	(0.19, 0.56)	
	Divorced	0.55	0.13	(0.29, 0.81)	
	Widowed	0.47	0.03	(0.41, 0.53)	
	Never	0.41	0.01	(0.38, 0.44)	
	Domestic Partner	0.27	0.04	(0.19, 0.35)	
Employment	Employed for an employer	0.46	0.01	(0.43, 0.48)	0.653
	Self-employed	0.49	0.01	(0.46, 0.51)	
	Retired	0.49	0.04	(0.40, 0.58)	
	Student	0.46	0.03	(0.41, 0.51)	
	Homemaker	0.49	0.01	(0.47, 0.51)	
	Unemployed and looking for a job	0.49	0.02	(0.45, 0.53)	
	None of these/other	0.47	0.02	(0.42, 0.51)	
Religious service attendance	>1/week	0.52	0.01	(0.49, 0.54)	<0.001
	1/week	0.50	0.01	(0.47, 0.52)	
	1-3/month	0.50	0.01	(0.48, 0.53)	
	A few times a year	0.42	0.01	(0.40, 0.45)	
	Never	0.42	0.02	(0.39, 0.45)	
Education	up to 8 years	0.49	0.01	(0.47, 0.50)	<0.001
	9-15 years	0.42	0.02	(0.40, 0.45)	

Variable	Category	Mean	SE	95% CI	Global p-value
	16+years	0.39	0.04	(0.31, 0.47)	
Immigration status	Born in this country	0.48	0.01	(0.47, 0.49)	0.028
	Born in another country	0.56	0.05	(0.45, 0.66)	
Religious affiliation	Christianity	0.58	0.04	(0.51, 0.65)	<0.001
	Islam	0.52	0.02	(0.48, 0.55)	
	Hinduism	0.48	0.01	(0.46, 0.49)	
	Buddhism	0.25	0.04	(0.17, 0.32)	
	Sikhism	0.37	0.05	(0.26, 0.48)	
	Jainism	0.28	0.13	(-0.03, 0.58)	
	Primal, Animist, or Folk religion	0.57	0.12	(0.32, 0.82)	
	Some other religion	0.58	0.07	(0.44, 0.72)	
	No religion/Atheist/Agnostic	0.17	0.13	(-0.12, 0.45)	
Race and ethnicity	General	0.48	0.01	(0.45, 0.50)	0.003
	Other backward caste	0.50	0.01	(0.48, 0.52)	
	Schedule caste	0.46	0.01	(0.44, 0.49)	
	Schedule tribe	0.45	0.02	(0.42, 0.49)	

Table S8a. Nationally representative descriptive statistics for Indonesia

Characteristic	N = 6,992[1]
**Age group**	
18-24	1,216 (17%)
25-29	849 (12%)
30-39	1,591 (23%)
40-49	1,576 (23%)
50-59	1,169 (17%)
60-69	490 (7.0%)
70-79	83 (1.2%)
80 or older	17 (0.2%)
Missing	0 (0%)
**Gender**	
Male	3,461 (50%)
Female	3,513 (50%)
Other	7 (<0.1%)
Missing	11 (0.2%)
**Marital status**	
Married	4,846 (69%)
Separated	81 (1.2%)
Divorced	196 (2.8%)
Widowed	425 (6.1%)
Never	1,381 (20%)
Domestic Partner	18 (0.3%)
Missing	45 (0.6%)
**Employment**	
Employed for an employer	1,323 (19%)
Self-employed	2,187 (31%)
Retired	78 (1.1%)
Student	272 (3.9%)
Homemaker	2,138 (31%)
Unemployed and looking for a job	529 (7.6%)
None of these/other	448 (6.4%)
Missing	18 (0.3%)
**Religious service attendance**	
>1/week	2,667 (38%)
1/week	2,529 (36%)
1-3/month	786 (11%)
A few times a year	659 (9.4%)
Never	332 (4.8%)
Missing	18 (0.3%)
**Education**	
up to 8 years	3,079 (44%)
9-15 years	3,491 (50%)
16+years	419 (6.0%)
Missing	2 (<0.1%)
**Immigration**	
Born in this country	6,958 (100%)
Born in another country	34 (0.5%)
Missing	0 (0%)
**Religious affiliation**	

Characteristic	N = 6,992[1]
Christianity	504 (7.2%)
Islam	6,406 (92%)
Hinduism	73 (1.0%)
Buddhism	3 (<0.1%)
Taoism	1 (<0.1%)
Some other religion	1 (<0.1%)
Missing	4 (<0.1%)
**Race and ethnicity**	
Bali	69 (1.0%)
Banjar/Melayu Banjar	320 (4.6%)
Batak	165 (2.4%)
Betawi	251 (3.6%)
Bugis	243 (3.5%)
Jawa	2,846 (41%)
Madura	262 (3.7%)
Makasar	91 (1.3%)
Minangkabau	273 (3.9%)
Other	1,262 (18%)
Sunda/Parahyangan	1,172 (17%)
Missing	38 (0.5%)

[1]n (%)

Table S8b. Means by demographic category for Indonesia

Variable	Category	Mean	SE	95% CI	Global p-value
Age group	18-24	0.97	0.01	(0.95, 0.98)	<0.001
	25-29	0.96	0.01	(0.95, 0.98)	
	30-39	0.95	0.01	(0.93, 0.96)	
	40-49	0.95	0.01	(0.94, 0.97)	
	50-59	0.94	0.01	(0.92, 0.96)	
	60-69	0.93	0.02	(0.89, 0.96)	
	70-79	0.89	0.06	(0.77, 1.00)	
	80 or older	1.00			
Gender	Male	0.95	0.01	(0.94, 0.96)	<0.001
	Female	0.95	0.01	(0.94, 0.96)	
	Other	1.00	0.00	(1.00, 1.00)	
Marital status	Married	0.95	0.01	(0.94, 0.95)	<0.001
	Separated	0.99	0.01	(0.98, 1.01)	
	Divorced	0.99	0.01	(0.98, 1.00)	
	Widowed	0.93	0.02	(0.90, 0.97)	
	Never	0.97	0.01	(0.96, 0.98)	
	Domestic Partner	0.83	0.16	(0.50, 1.16)	
Employment	Employed for an employer	0.95	0.01	(0.93, 0.97)	0.761
	Self-employed	0.95	0.01	(0.94, 0.97)	
	Retired	0.95	0.03	(0.89, 1.01)	
	Student	0.97	0.01	(0.94, 0.99)	
	Homemaker	0.94	0.01	(0.93, 0.96)	
	Unemployed and looking for a job	0.96	0.01	(0.94, 0.98)	
	None of these/other	0.95	0.01	(0.92, 0.98)	
Religious service attendance	>1/week	0.97	0.01	(0.96, 0.98)	<0.001
	1/week	0.95	0.01	(0.93, 0.96)	
	1-3/month	0.94	0.01	(0.92, 0.96)	
	A few times a year	0.95	0.01	(0.93, 0.97)	
	Never	0.86	0.02	(0.82, 0.91)	
Education	up to 8 years	0.93	0.01	(0.92, 0.95)	<0.001

Variable	Category	Mean	SE	95% CI	Global p-value
	9-15 years	0.96	0.00	(0.95, 0.97)	
	16+years	0.97	0.01	(0.96, 0.99)	
Immigration status	Born in this country	0.95	0.00	(0.94, 0.96)	0.071
	Born in another country	0.85	0.08	(0.68, 1.01)	
Religious affiliation	Christianity	0.82	0.03	(0.77, 0.88)	<0.001
	Islam	0.96	0.00	(0.96, 0.97)	
	Hinduism	0.72	0.06	(0.59, 0.84)	
	Buddhism	1.00	0.00	(1.00, 1.00)	
Race and ethnicity	Other	0.91	0.01	(0.88, 0.94)	<0.001
	Bali	0.72	0.07	(0.58, 0.85)	
	Banjar/Melayu Banjar	0.97	0.01	(0.95, 1.00)	
	Batak	0.90	0.03	(0.85, 0.96)	
	Betawi	0.96	0.02	(0.92, 1.00)	
	Bugis	0.96	0.02	(0.92, 0.99)	
	Jawa	0.96	0.01	(0.95, 0.98)	
	Madura	0.95	0.02	(0.91, 0.99)	
	Makasar	0.99	0.01	(0.97, 1.01)	
	Minangkabau	0.95	0.01	(0.92, 0.97)	
	Sunda/Parahyangan	0.97	0.01	(0.95, 0.98)	

Table S9a. Nationally representative descriptive statistics for Israel

Characteristic	N = 3,669[1]
**Age group**	
18-24	553 (15%)
25-29	407 (11%)
30-39	666 (18%)
40-49	616 (17%)
50-59	542 (15%)
60-69	469 (13%)
70-79	336 (9.2%)
80 or older	79 (2.2%)
Missing	0 (0%)
**Gender**	
Male	1,791 (49%)
Female	1,872 (51%)
Other	0 (<0.1%)
Missing	6 (0.2%)
**Marital status**	
Married	2,056 (56%)
Separated	48 (1.3%)
Divorced	258 (7.0%)
Widowed	212 (5.8%)
Never	834 (23%)
Domestic Partner	193 (5.3%)
Missing	69 (1.9%)
**Employment**	
Employed for an employer	1,793 (49%)
Self-employed	424 (12%)
Retired	576 (16%)
Student	388 (11%)
Homemaker	211 (5.7%)
Unemployed and looking for a job	148 (4.0%)
None of these/other	118 (3.2%)
Missing	10 (0.3%)
**Religious service attendance**	
>1/week	649 (18%)
1/week	495 (14%)
1-3/month	374 (10%)
A few times a year	1,014 (28%)
Never	1,122 (31%)
Missing	14 (0.4%)
**Education**	
up to 8 years	224 (6.1%)
9-15 years	1,517 (41%)
16+years	1,926 (52%)
Missing	2 (<0.1%)
**Immigration**	
Born in this country	2,796 (76%)
Born in another country	868 (24%)
Missing	5 (0.1%)
**Religious affiliation**	

**Characteristic**	**N = 3,669**[1]
Christianity	39 (1.1%)
Islam	656 (18%)
Judaism	2,897 (79%)
Baha'i	2 (<0.1%)
Taoism	1 (<0.1%)
Primal, Animist, or Folk religion	1 (<0.1%)
Some other religion	5 (0.1%)
No religion/Atheist/Agnostic	64 (1.7%)
Missing	4 (0.1%)
**Race and ethnicity**	
Arab	674 (18%)
Jewish	2,926 (80%)
Other	39 (1.1%)
Missing	30 (0.8%)

[1]n (%)

Table S9b. Means by demographic category for Israel

Variable	Category	Mean	SE	95% CI	Global p-value
Age group	18-24	0.63	0.03	(0.57, 0.68)	<0.001
	25-29	0.54	0.04	(0.47, 0.62)	
	30-39	0.56	0.03	(0.51, 0.61)	
	40-49	0.54	0.03	(0.48, 0.60)	
	50-59	0.56	0.03	(0.50, 0.62)	
	60-69	0.53	0.03	(0.47, 0.60)	
	70-79	0.43	0.03	(0.37, 0.50)	
	80 or older	0.39	0.06	(0.28, 0.51)	
Gender	Male	0.55	0.02	(0.52, 0.59)	0.496
	Female	0.54	0.02	(0.50, 0.58)	
Marital status	Married	0.58	0.02	(0.54, 0.63)	<0.001
	Separated	0.41	0.07	(0.28, 0.55)	
	Divorced	0.45	0.04	(0.37, 0.52)	
	Widowed	0.51	0.04	(0.43, 0.59)	
	Never	0.55	0.02	(0.50, 0.60)	
	Domestic Partner	0.34	0.05	(0.25, 0.43)	
Employment	Employed for an employer	0.53	0.02	(0.49, 0.57)	<0.001
	Self-employed	0.53	0.03	(0.47, 0.59)	
	Retired	0.48	0.03	(0.41, 0.54)	
	Student	0.67	0.04	(0.59, 0.74)	
	Homemaker	0.71	0.04	(0.63, 0.79)	
	Unemployed and looking for a job	0.51	0.06	(0.39, 0.63)	
	None of these/other	0.59	0.06	(0.46, 0.71)	
Religious service attendance	>1/week	0.94	0.01	(0.92, 0.96)	<0.001
	1/week	0.87	0.02	(0.82, 0.91)	
	1-3/month	0.75	0.02	(0.70, 0.80)	
	A few times a year	0.41	0.03	(0.36, 0.46)	
	Never	0.23	0.02	(0.20, 0.27)	
Education	up to 8 years	0.63	0.06	(0.52, 0.75)	<0.001
	9-15 years	0.61	0.02	(0.57, 0.65)	

Variable	Category	Mean	SE	95% CI	Global p-value
	16+years	0.49	0.02	(0.44, 0.53)	
Immigration status	Born in this country	0.60	0.02	(0.56, 0.63)	<0.001
	Born in another country	0.38	0.03	(0.33, 0.43)	
Religious affiliation	Christianity	0.40	0.09	(0.22, 0.59)	<0.001
	Islam	0.72	0.03	(0.66, 0.78)	
	Judaism	0.52	0.02	(0.48, 0.56)	
	Baha'i	1.00			
	Primal, Animist, or Folk religion	0.48			
	Some other religion	0.37	0.29	(-0.56, 1.30)	
	No religion/Atheist/Agnostic	0.12	0.04	(0.04, 0.20)	
Race and ethnicity	Other	0.17	0.08	(0.01, 0.33)	<0.001
	Arab	0.71	0.03	(0.64, 0.77)	
	Jewish	0.52	0.02	(0.48, 0.55)	

Table S10a. Nationally representative descriptive statistics for Japan

Characteristic	N = 20,543[1]
**Age group**	
18-24	1,589 (7.7%)
25-29	806 (3.9%)
30-39	2,851 (14%)
40-49	3,363 (16%)
50-59	3,770 (18%)
60-69	4,118 (20%)
70-79	3,554 (17%)
80 or older	493 (2.4%)
Missing	0 (0%)
**Gender**	
Male	9,847 (48%)
Female	10,602 (52%)
Other	28 (0.1%)
Missing	66 (0.3%)
**Marital status**	
Married	11,837 (58%)
Separated	190 (0.9%)
Divorced	2,126 (10%)
Widowed	1,179 (5.7%)
Never	5,004 (24%)
Domestic Partner	144 (0.7%)
Missing	64 (0.3%)
**Employment**	
Employed for an employer	10,853 (53%)
Self-employed	1,748 (8.5%)
Retired	2,535 (12%)
Student	491 (2.4%)
Homemaker	1,276 (6.2%)
Unemployed and looking for a job	622 (3.0%)
None of these/other	2,983 (15%)
Missing	36 (0.2%)
**Religious service attendance**	
>1/week	316 (1.5%)
1/week	348 (1.7%)
1-3/month	862 (4.2%)
A few times a year	3,112 (15%)
Never	15,788 (77%)
Missing	117 (0.6%)
**Education**	
up to 8 years	567 (2.8%)
9-15 years	14,893 (72%)
16+years	5,083 (25%)
Missing	0 (0%)
**Immigration**	
Born in this country	19,548 (95%)
Born in another country	158 (0.8%)
Missing	837 (4.1%)
**Religious affiliation**	

**Characteristic**	**N = 20,543**[1]
Christianity	381 (1.9%)
Islam	10 (<0.1%)
Hinduism	5 (<0.1%)
Buddhism	6,709 (33%)
Judaism	10 (<0.1%)
Sikhism	6 (<0.1%)
Baha'i	2 (<0.1%)
Jainism	11 (<0.1%)
Shinto	469 (2.3%)
Taoism	7 (<0.1%)
Confucianism	17 (<0.1%)
Primal, Animist, or Folk religion	19 (<0.1%)
Some other religion	46 (0.2%)
No religion/Atheist/Agnostic	12,497 (61%)
Missing	355 (1.7%)
**Race and ethnicity**	
Missing	20,543 (100%)

[1]n (%)

Table S10b. Means by demographic category for Japan

Variable	Category	Mean	SE	95% CI	Global p-value
Age group	18-24	0.24	0.01	(0.22, 0.27)	<0.001
	25-29	0.23	0.02	(0.19, 0.26)	
	30-39	0.22	0.01	(0.20, 0.23)	
	40-49	0.22	0.01	(0.20, 0.23)	
	50-59	0.22	0.01	(0.20, 0.24)	
	60-69	0.21	0.01	(0.19, 0.22)	
	70-79	0.15	0.01	(0.14, 0.17)	
	80 or older	0.17	0.02	(0.12, 0.21)	
Gender	Male	0.18	0.00	(0.17, 0.19)	<0.001
	Female	0.23	0.00	(0.22, 0.24)	
	Other	0.27	0.09	(0.09, 0.46)	
Marital status	Married	0.20	0.00	(0.19, 0.21)	<0.001
	Separated	0.15	0.03	(0.08, 0.21)	
	Divorced	0.23	0.01	(0.20, 0.25)	
	Widowed	0.21	0.02	(0.18, 0.24)	
	Never	0.21	0.01	(0.20, 0.22)	
	Domestic Partner	0.27	0.05	(0.18, 0.36)	
Employment	Employed for an employer	0.22	0.01	(0.21, 0.23)	<0.001
	Self-employed	0.23	0.01	(0.20, 0.25)	
	Retired	0.16	0.01	(0.14, 0.17)	
	Student	0.24	0.02	(0.20, 0.27)	
	Homemaker	0.23	0.01	(0.20, 0.25)	
	Unemployed and looking for a job	0.21	0.01	(0.18, 0.24)	
	None of these/other	0.18	0.01	(0.17, 0.20)	
Religious service attendance	>1/week	0.43	0.03	(0.37, 0.50)	<0.001
	1/week	0.55	0.03	(0.48, 0.62)	
	1-3/month	0.30	0.02	(0.26, 0.33)	
	A few times a year	0.26	0.01	(0.24, 0.28)	
	Never	0.18	0.00	(0.17, 0.19)	
Education	up to 8 years	0.22	0.02	(0.18, 0.26)	0.420

Variable	Category	Mean	SE	95% CI	Global p-value
	9-15 years	0.20	0.00	(0.20, 0.21)	
	16+years	0.21	0.01	(0.20, 0.23)	
Immigration status	Born in this country	0.21	0.00	(0.20, 0.21)	0.002
	Born in another country	0.15	0.04	(0.08, 0.21)	
Religious affiliation	Christianity	0.51	0.03	(0.45, 0.57)	<0.001
	Islam	0.38	0.21	(-0.11, 0.88)	
	Hinduism	0.44	0.26	(-0.38, 1.26)	
	Buddhism	0.25	0.01	(0.24, 0.26)	
	Judaism	0.10	0.10	(-0.14, 0.34)	
	Sikhism	0.21	0.18	(-0.31, 0.74)	
	Baha'i	0.00			
	Jainism	0.66	0.23	(0.15, 1.18)	
	Shinto	0.31	0.03	(0.26, 0.36)	
	Taoism	0.36	0.20	(-0.14, 0.86)	
	Confucianism	0.51	0.12	(0.25, 0.77)	
	Primal, Animist, or Folk religion	0.42	0.15	(0.10, 0.74)	
	Some other religion	0.48	0.09	(0.30, 0.66)	
	No religion/Atheist/Agnostic	0.17	0.00	(0.16, 0.18)	

Table S11a. Nationally representative descriptive statistics for Kenya

Characteristic	N = 11,389[1]
**Age group**	
18-24	2,868 (25%)
25-29	2,035 (18%)
30-39	2,564 (23%)
40-49	1,708 (15%)
50-59	1,072 (9.4%)
60-69	710 (6.2%)
70-79	360 (3.2%)
80 or older	67 (0.6%)
Missing	5 (<0.1%)
**Gender**	
Male	5,567 (49%)
Female	5,813 (51%)
Other	2 (<0.1%)
Missing	7 (<0.1%)
**Marital status**	
Married	6,626 (58%)
Separated	467 (4.1%)
Divorced	111 (1.0%)
Widowed	464 (4.1%)
Never	3,531 (31%)
Domestic Partner	146 (1.3%)
Missing	43 (0.4%)
**Employment**	
Employed for an employer	1,467 (13%)
Self-employed	3,630 (32%)
Retired	319 (2.8%)
Student	1,136 (10.0%)
Homemaker	1,537 (13%)
Unemployed and looking for a job	3,153 (28%)
None of these/other	138 (1.2%)
Missing	9 (<0.1%)
**Religious service attendance**	
>1/week	2,774 (24%)
1/week	6,063 (53%)
1-3/month	1,219 (11%)
A few times a year	855 (7.5%)
Never	465 (4.1%)
Missing	13 (0.1%)
**Education**	
up to 8 years	4,485 (39%)
9-15 years	6,115 (54%)
16+years	783 (6.9%)
Missing	6 (<0.1%)
**Immigration**	
Born in this country	11,270 (99%)
Born in another country	117 (1.0%)
Missing	2 (<0.1%)
**Religious affiliation**	

Characteristic	N = 11,389[1]
Christianity	10,334 (91%)
Islam	918 (8.1%)
Buddhism	1 (<0.1%)
Judaism	3 (<0.1%)
Baha'i	1 (<0.1%)
Jainism	1 (<0.1%)
Confucianism	3 (<0.1%)
Primal, Animist, or Folk religion	7 (<0.1%)
Some other religion	5 (<0.1%)
No religion/Atheist/Agnostic	108 (0.9%)
Missing	9 (<0.1%)
**Race and ethnicity**	
Embu	197 (1.7%)
Kalenjin	1,377 (12%)
Kamba	1,299 (11%)
Kenyan Somali/Somali	396 (3.5%)
Kikuyu	2,119 (19%)
Kisii	789 (6.9%)
Luhya	1,943 (17%)
Luo	1,120 (9.8%)
Maasai	237 (2.1%)
Meru	630 (5.5%)
Miji Kenda tribes	708 (6.2%)
Other	548 (4.8%)
Missing	27 (0.2%)

[1]n (%)

Table S11b. Means by demographic category for Kenya

Variable	Category	Mean	SE	95% CI	Global p-value
Age group	18-24	0.67	0.01	(0.65, 0.69)	<0.001
	25-29	0.69	0.01	(0.67, 0.72)	
	30-39	0.74	0.01	(0.72, 0.76)	
	40-49	0.75	0.01	(0.72, 0.78)	
	50-59	0.74	0.02	(0.70, 0.78)	
	60-69	0.80	0.02	(0.75, 0.84)	
	70-79	0.75	0.04	(0.67, 0.82)	
	80 or older	0.72	0.09	(0.55, 0.89)	
Gender	Male	0.71	0.01	(0.70, 0.73)	<0.001
	Female	0.73	0.01	(0.71, 0.74)	
	Other	1.00			
Marital status	Married	0.74	0.01	(0.72, 0.75)	<0.001
	Separated	0.69	0.03	(0.63, 0.74)	
	Divorced	0.69	0.06	(0.57, 0.81)	
	Widowed	0.75	0.03	(0.69, 0.81)	
	Never	0.69	0.01	(0.67, 0.71)	
	Domestic Partner	0.56	0.06	(0.44, 0.68)	
Employment	Employed for an employer	0.72	0.02	(0.69, 0.75)	<0.001
	Self-employed	0.74	0.01	(0.72, 0.76)	
	Retired	0.76	0.04	(0.69, 0.83)	
	Student	0.68	0.02	(0.65, 0.71)	
	Homemaker	0.75	0.02	(0.72, 0.78)	
	Unemployed and looking for a job	0.70	0.01	(0.67, 0.72)	
	None of these/other	0.70	0.05	(0.60, 0.80)	
Religious service attendance	>1/week	0.81	0.01	(0.79, 0.83)	<0.001
	1/week	0.73	0.01	(0.72, 0.75)	
	1-3/month	0.64	0.02	(0.61, 0.67)	
	A few times a year	0.57	0.02	(0.52, 0.61)	
	Never	0.53	0.04	(0.46, 0.60)	
Education	up to 8 years	0.70	0.01	(0.69, 0.72)	<0.001

Variable	Category	Mean	SE	95% CI	Global p-value
	9-15 years	0.73	0.01	(0.71, 0.74)	
	16+years	0.75	0.02	(0.72, 0.79)	
Immigration status	Born in this country	0.72	0.01	(0.71, 0.73)	<0.001
	Born in another country	0.80	0.04	(0.72, 0.88)	
Religious affiliation	Christianity	0.72	0.01	(0.71, 0.73)	<0.001
	Islam	0.74	0.02	(0.70, 0.77)	
	Judaism	0.83			
	Primal, Animist, or Folk religion	0.91	0.09	(0.66, 1.15)	
	Some other religion	0.92	0.09	(0.64, 1.20)	
	No religion/Atheist/Agnostic	0.52	0.06	(0.40, 0.64)	
Race and ethnicity	Other	0.72	0.02	(0.69, 0.76)	0.482
	Embu	0.74	0.05	(0.63, 0.84)	
	Kalenjin	0.73	0.02	(0.69, 0.76)	
	Kamba	0.73	0.02	(0.69, 0.77)	
	Kenyan Somali/Somali	0.73	0.03	(0.68, 0.78)	
	Kikuyu	0.73	0.01	(0.70, 0.76)	
	Kisii	0.75	0.03	(0.70, 0.80)	
	Luhya	0.70	0.02	(0.67, 0.73)	
	Luo	0.71	0.02	(0.67, 0.75)	
	Maasai	0.67	0.04	(0.59, 0.74)	
	Meru	0.68	0.02	(0.64, 0.72)	
	Miji Kenda tribes	0.74	0.03	(0.69, 0.79)	

Table S12a. Nationally representative descriptive statistics for Mexico

Characteristic	N = 5,776[1]
**Age group**	
18-24	986 (17%)
25-29	623 (11%)
30-39	1,312 (23%)
40-49	1,027 (18%)
50-59	873 (15%)
60-69	611 (11%)
70-79	277 (4.8%)
80 or older	68 (1.2%)
Missing	0 (0%)
**Gender**	
Male	2,755 (48%)
Female	2,997 (52%)
Other	3 (<0.1%)
Missing	21 (0.4%)
**Marital status**	
Married	2,089 (36%)
Separated	403 (7.0%)
Divorced	230 (4.0%)
Widowed	347 (6.0%)
Never	1,432 (25%)
Domestic Partner	1,109 (19%)
Missing	166 (2.9%)
**Employment**	
Employed for an employer	1,921 (33%)
Self-employed	1,091 (19%)
Retired	386 (6.7%)
Student	247 (4.3%)
Homemaker	1,257 (22%)
Unemployed and looking for a job	564 (9.8%)
None of these/other	169 (2.9%)
Missing	141 (2.4%)
**Religious service attendance**	
>1/week	609 (11%)
1/week	1,261 (22%)
1-3/month	676 (12%)
A few times a year	2,054 (36%)
Never	1,134 (20%)
Missing	43 (0.7%)
**Education**	
up to 8 years	1,291 (22%)
9-15 years	3,180 (55%)
16+years	1,304 (23%)
Missing	1 (<0.1%)
**Immigration**	
Born in this country	5,517 (96%)
Born in another country	108 (1.9%)
Missing	151 (2.6%)
**Religious affiliation**	

Characteristic	N = 5,776[1]
Christianity	4,844 (84%)
Islam	2 (<0.1%)
Hinduism	3 (<0.1%)
Buddhism	6 (0.1%)
Judaism	7 (0.1%)
Baha’i	1 (<0.1%)
Jainism	1 (<0.1%)
Shinto	2 (<0.1%)
Taoism	4 (<0.1%)
Confucianism	1 (<0.1%)
Primal, Animist, or Folk religion	20 (0.3%)
Some other religion	41 (0.7%)
No religion/Atheist/Agnostic	770 (13%)
Missing	75 (1.3%)
**Race and ethnicity**	
Black	108 (1.9%)
Indigenous	594 (10%)
Mestizo	2,762 (48%)
Mulatto	63 (1.1%)
Other	339 (5.9%)
White	1,116 (19%)
Missing	794 (14%)

[1]n (%)

Table S12b. Means by demographic category for Mexico

Variable	Category	Mean	SE	95% CI	Global p-value
Age group	18-24	0.58	0.02	(0.54, 0.62)	<0.001
	25-29	0.60	0.02	(0.55, 0.64)	
	30-39	0.64	0.02	(0.61, 0.67)	
	40-49	0.62	0.02	(0.58, 0.66)	
	50-59	0.62	0.02	(0.57, 0.66)	
	60-69	0.56	0.03	(0.50, 0.62)	
	70-79	0.47	0.05	(0.38, 0.56)	
	80 or older	0.67	0.09	(0.49, 0.84)	
Gender	Male	0.60	0.01	(0.58, 0.63)	1.000
	Female	0.60	0.01	(0.58, 0.63)	
	Other	0.58	0.19	(-0.53, 1.69)	
Marital status	Married	0.63	0.01	(0.60, 0.66)	0.090
	Separated	0.58	0.03	(0.52, 0.64)	
	Divorced	0.62	0.04	(0.54, 0.70)	
	Widowed	0.55	0.04	(0.47, 0.62)	
	Never	0.59	0.02	(0.55, 0.62)	
	Domestic Partner	0.60	0.02	(0.57, 0.64)	
Employment	Employed for an employer	0.63	0.01	(0.60, 0.66)	<0.001
	Self-employed	0.64	0.02	(0.60, 0.68)	
	Retired	0.56	0.04	(0.48, 0.65)	
	Student	0.52	0.04	(0.43, 0.60)	
	Homemaker	0.59	0.02	(0.56, 0.62)	
	Unemployed and looking for a job	0.54	0.03	(0.49, 0.59)	
	None of these/other	0.54	0.05	(0.43, 0.64)	
Religious service attendance	>1/week	0.76	0.02	(0.72, 0.80)	<0.001
	1/week	0.70	0.02	(0.66, 0.73)	
	1-3/month	0.65	0.02	(0.60, 0.70)	
	A few times a year	0.57	0.01	(0.54, 0.60)	
	Never	0.45	0.02	(0.41, 0.48)	
Education	up to 8 years	0.57	0.02	(0.53, 0.61)	<0.001

Variable	Category	Mean	SE	95% CI	Global p-value
	9-15 years	0.60	0.01	(0.58, 0.63)	
	16+years	0.63	0.02	(0.60, 0.67)	
Immigration status	Born in this country	0.60	0.01	(0.59, 0.62)	0.037
	Born in another country	0.52	0.06	(0.39, 0.64)	
Religious affiliation	Christianity	0.63	0.01	(0.61, 0.65)	<0.001
	Islam	0.24			
	Hinduism	0.82	0.18	(-0.26, 1.91)	
	Buddhism	0.30	0.20	(-0.28, 0.88)	
	Judaism	0.83	0.11	(0.52, 1.15)	
	Shinto	0.33			
	Taoism	0.94	0.08	(0.63, 1.24)	
	Primal, Animist, or Folk religion	0.50	0.15	(0.18, 0.82)	
	Some other religion	0.78	0.07	(0.64, 0.92)	
	No religion/Atheist/Agnostic	0.44	0.02	(0.39, 0.48)	
Race and ethnicity	Black	0.57	0.06	(0.46, 0.69)	0.250
	Indigenous	0.58	0.03	(0.53, 0.63)	
	Other	0.62	0.03	(0.56, 0.69)	
	White	0.59	0.02	(0.55, 0.63)	
	Mestizo	0.62	0.01	(0.59, 0.64)	
	Mulatto	0.49	0.08	(0.33, 0.66)	

Table S13a. Nationally representative descriptive statistics for Nigeria

Characteristic	N = 6,827[1]
**Age group**	
18-24	1,533 (22%)
25-29	1,193 (17%)
30-39	1,943 (28%)
40-49	1,059 (16%)
50-59	619 (9.1%)
60-69	296 (4.3%)
70-79	133 (2.0%)
80 or older	50 (0.7%)
Missing	0 (0%)
**Gender**	
Male	3,371 (49%)
Female	3,456 (51%)
Other	0 (<0.1%)
Missing	0 (0%)
**Marital status**	
Married	4,065 (60%)
Separated	117 (1.7%)
Divorced	71 (1.0%)
Widowed	231 (3.4%)
Never	2,289 (34%)
Domestic Partner	12 (0.2%)
Missing	42 (0.6%)
**Employment**	
Employed for an employer	699 (10%)
Self-employed	3,898 (57%)
Retired	178 (2.6%)
Student	650 (9.5%)
Homemaker	499 (7.3%)
Unemployed and looking for a job	684 (10%)
None of these/other	211 (3.1%)
Missing	8 (0.1%)
**Religious service attendance**	
>1/week	4,049 (59%)
1/week	1,895 (28%)
1-3/month	531 (7.8%)
A few times a year	254 (3.7%)
Never	77 (1.1%)
Missing	20 (0.3%)
**Education**	
up to 8 years	2,575 (38%)
9-15 years	4,120 (60%)
16+years	130 (1.9%)
Missing	2 (<0.1%)
**Immigration**	
Born in this country	6,779 (99%)
Born in another country	47 (0.7%)
Missing	1 (<0.1%)
**Religious affiliation**	

**Characteristic**	**N = 6,827**[1]
Christianity	3,476 (51%)
Islam	3,302 (48%)
Shinto	1 (<0.1%)
Confucianism	0 (<0.1%)
Primal, Animist, or Folk religion	24 (0.3%)
Some other religion	1 (<0.1%)
No religion/Atheist/Agnostic	15 (0.2%)
Missing	9 (0.1%)
**Race and ethnicity**	
Edo	116 (1.7%)
Efik	48 (0.7%)
Fulani	266 (3.9%)
Hausa	2,342 (34%)
Ibibio	180 (2.6%)
Idoma	61 (0.9%)
Igala	77 (1.1%)
Igbo (Ibo)	1,111 (16%)
Ijaw	110 (1.6%)
Kanuri	31 (0.5%)
Other	1,014 (15%)
Tiv	198 (2.9%)
Urhobo	38 (0.6%)
Yoruba	1,230 (18%)
Missing	4 (<0.1%)

[1]n (%)

Table S13b. Means by demographic category for Nigeria

Variable	Category	Mean	SE	95% CI	Global p-value
Age group	18-24	0.70	0.02	(0.67, 0.74)	<0.001
	25-29	0.70	0.02	(0.67, 0.74)	
	30-39	0.74	0.01	(0.71, 0.76)	
	40-49	0.73	0.02	(0.69, 0.77)	
	50-59	0.81	0.03	(0.75, 0.86)	
	60-69	0.78	0.05	(0.69, 0.87)	
	70-79	0.66	0.08	(0.50, 0.81)	
	80 or older	0.79	0.09	(0.61, 0.96)	
Gender	Male	0.76	0.01	(0.73, 0.78)	<0.001
	Female	0.70	0.01	(0.68, 0.72)	
Marital status	Married	0.74	0.01	(0.72, 0.77)	0.002
	Separated	0.62	0.07	(0.49, 0.75)	
	Divorced	0.61	0.08	(0.45, 0.77)	
	Widowed	0.70	0.06	(0.59, 0.81)	
	Never	0.72	0.01	(0.70, 0.74)	
	Domestic Partner	0.38	0.20	(-0.06, 0.82)	
Employment	Employed for an employer	0.71	0.02	(0.67, 0.75)	0.491
	Self-employed	0.72	0.01	(0.70, 0.74)	
	Retired	0.71	0.07	(0.57, 0.84)	
	Student	0.74	0.02	(0.70, 0.77)	
	Homemaker	0.79	0.03	(0.73, 0.85)	
	Unemployed and looking for a job	0.74	0.02	(0.69, 0.78)	
	None of these/other	0.76	0.04	(0.68, 0.84)	
Religious service attendance	>1/week	0.74	0.01	(0.72, 0.77)	0.026
	1/week	0.73	0.01	(0.70, 0.75)	
	1-3/month	0.70	0.03	(0.63, 0.76)	
	A few times a year	0.67	0.05	(0.58, 0.76)	
	Never	0.61	0.08	(0.45, 0.77)	
Education	up to 8 years	0.74	0.02	(0.71, 0.78)	0.662
	9-15 years	0.72	0.01	(0.70, 0.74)	

Variable	Category	Mean	SE	95% CI	Global p-value
	16+years	0.74	0.03	(0.69, 0.80)	
Immigration status	Born in this country	0.73	0.01	(0.71, 0.75)	0.658
	Born in another country	0.79	0.08	(0.63, 0.95)	
Religious affiliation	Christianity	0.71	0.01	(0.69, 0.73)	<0.001
	Islam	0.75	0.01	(0.72, 0.78)	
	Primal, Animist, or Folk religion	0.56	0.18	(0.19, 0.93)	
	No religion/Atheist/Agnostic	0.63	0.18	(0.23, 1.03)	
Race and ethnicity	Other	0.74	0.02	(0.71, 0.78)	<0.001
	Edo	0.69	0.06	(0.56, 0.81)	
	Efik	0.64	0.08	(0.49, 0.80)	
	Fulani	0.74	0.04	(0.67, 0.82)	
	Hausa	0.78	0.02	(0.74, 0.81)	
	Ibibio	0.66	0.07	(0.52, 0.80)	
	Idoma	0.45	0.09	(0.27, 0.63)	
	Igala	0.71	0.05	(0.61, 0.82)	
	Igbo (Ibo)	0.69	0.02	(0.66, 0.72)	
	Ijaw	0.65	0.05	(0.55, 0.76)	
	Kanuri	0.74	0.07	(0.59, 0.89)	
	Tiv	0.78	0.02	(0.75, 0.82)	
	Urhobo	0.50	0.09	(0.31, 0.69)	
	Yoruba	0.70	0.02	(0.66, 0.73)	

Table S14a. Nationally representative descriptive statistics for Philippines

Characteristic	N = 5,292[1]
**Age group**	
18-24	1,073 (20%)
25-29	695 (13%)
30-39	1,160 (22%)
40-49	972 (18%)
50-59	732 (14%)
60-69	495 (9.4%)
70-79	143 (2.7%)
80 or older	23 (0.4%)
Missing	0 (0%)
**Gender**	
Male	2,625 (50%)
Female	2,643 (50%)
Other	13 (0.2%)
Missing	11 (0.2%)
**Marital status**	
Married	2,385 (45%)
Separated	249 (4.7%)
Divorced	9 (0.2%)
Widowed	274 (5.2%)
Never	1,206 (23%)
Domestic Partner	1,152 (22%)
Missing	16 (0.3%)
**Employment**	
Employed for an employer	1,350 (26%)
Self-employed	1,379 (26%)
Retired	158 (3.0%)
Student	585 (11%)
Homemaker	1,049 (20%)
Unemployed and looking for a job	658 (12%)
None of these/other	113 (2.1%)
Missing	0 (0%)
**Religious service attendance**	
>1/week	844 (16%)
1/week	1,929 (36%)
1-3/month	1,374 (26%)
A few times a year	929 (18%)
Never	210 (4.0%)
Missing	6 (0.1%)
**Education**	
up to 8 years	1,188 (22%)
9-15 years	3,722 (70%)
16+years	381 (7.2%)
Missing	1 (<0.1%)
**Immigration**	
Born in this country	5,284 (100%)
Born in another country	8 (0.1%)
Missing	0 (0%)
**Religious affiliation**	

Characteristic	N = 5,292[1]
Christianity	4,914 (93%)
Islam	297 (5.6%)
Buddhism	4 (<0.1%)
Judaism	4 (<0.1%)
Baha'i	1 (<0.1%)
Primal, Animist, or Folk religion	5 (<0.1%)
Some other religion	35 (0.7%)
No religion/Atheist/Agnostic	23 (0.4%)
Missing	9 (0.2%)
**Race and ethnicity**	
Aeta	1 (<0.1%)
Badjao	2 (<0.1%)
Bicolano/Bikolano	300 (5.7%)
Cebuano	656 (12%)
Chinese-Filipino	3 (<0.1%)
Igorot	42 (0.8%)
Ilocano/Ilokano	429 (8.1%)
Ilonggo/Hiligaynon	428 (8.1%)
Kapampangan	107 (2.0%)
Maguindanaoan	84 (1.6%)
Mangyan	2 (<0.1%)
Maranao	39 (0.7%)
Masbateno	54 (1.0%)
Other	244 (4.6%)
Pangasinense	107 (2.0%)
Tagalog	1,691 (32%)
Tausug	94 (1.8%)
Visayan/Bisaya	739 (14%)
Waray	216 (4.1%)
Zamboangueno	51 (1.0%)
Missing	3 (<0.1%)

[1]n (%)

Table S14b. Means by demographic category for Philippines

Variable	Category	Mean	SE	95% CI	Global p-value
Age group	18-24	0.51	0.02	(0.47, 0.55)	<0.001
	25-29	0.61	0.03	(0.56, 0.66)	
	30-39	0.59	0.02	(0.56, 0.63)	
	40-49	0.51	0.02	(0.48, 0.54)	
	50-59	0.50	0.02	(0.46, 0.55)	
	60-69	0.48	0.03	(0.42, 0.54)	
	70-79	0.47	0.05	(0.38, 0.57)	
	80 or older	0.65	0.11	(0.42, 0.88)	
Gender	Male	0.52	0.01	(0.49, 0.55)	<0.001
	Female	0.55	0.01	(0.54, 0.57)	
	Other	0.81	0.10	(0.59, 1.04)	
Marital status	Married	0.55	0.01	(0.53, 0.57)	<0.001
	Separated	0.45	0.04	(0.38, 0.53)	
	Divorced	0.72	0.15	(0.38, 1.07)	
	Widowed	0.50	0.03	(0.43, 0.56)	
	Never	0.56	0.02	(0.52, 0.59)	
	Domestic Partner	0.52	0.02	(0.49, 0.56)	
Employment	Employed for an employer	0.54	0.02	(0.51, 0.58)	0.409
	Self-employed	0.54	0.02	(0.50, 0.57)	
	Retired	0.52	0.06	(0.41, 0.63)	
	Student	0.57	0.03	(0.51, 0.62)	
	Homemaker	0.54	0.01	(0.51, 0.57)	
	Unemployed and looking for a job	0.53	0.03	(0.48, 0.59)	
	None of these/other	0.41	0.05	(0.31, 0.52)	
Religious service attendance	>1/week	0.63	0.02	(0.58, 0.67)	<0.001
	1/week	0.54	0.01	(0.52, 0.57)	
	1-3/month	0.54	0.02	(0.50, 0.57)	
	A few times a year	0.49	0.02	(0.45, 0.53)	
	Never	0.33	0.04	(0.24, 0.41)	
Education	up to 8 years	0.45	0.02	(0.41, 0.49)	<0.001

Variable	Category	Mean	SE	95% CI	Global p-value
	9-15 years	0.55	0.01	(0.53, 0.57)	
	16+years	0.70	0.03	(0.64, 0.76)	
Immigration status	Born in this country	0.54	0.01	(0.52, 0.56)	0.961
	Born in another country	0.46	0.20	(-0.06, 0.97)	
Religious affiliation	Christianity	0.53	0.01	(0.52, 0.55)	<0.001
	Islam	0.62	0.04	(0.54, 0.69)	
	Buddhism	0.31	0.22	(-0.55, 1.18)	
	Judaism	0.00			
	Primal, Animist, or Folk religion	0.17	0.14	(-0.29, 0.64)	
	Some other religion	0.76	0.07	(0.62, 0.91)	
	No religion/Atheist/Agnostic	0.27	0.15	(-0.04, 0.58)	
Race and ethnicity	Other	0.58	0.04	(0.51, 0.65)	<0.001
	Aeta	0.27			
	Badjao	0.59			
	Bicolano/Bikolano	0.53	0.03	(0.46, 0.60)	
	Cebuano	0.55	0.02	(0.51, 0.59)	
	Chinese-Filipino	0.35	0.22	(-1.18, 1.88)	
	Igorot	0.76	0.03	(0.70, 0.82)	
	Ilocano/Ilokano	0.52	0.02	(0.47, 0.57)	
	Ilonggo/Hiligaynon	0.53	0.03	(0.48, 0.59)	
	Kapampangan	0.51	0.05	(0.40, 0.61)	
	Maguindanaoan	0.49	0.06	(0.37, 0.61)	
	Mangyan	1.00			
	Maranao	0.64	0.08	(0.47, 0.81)	
	Masbateno	0.52	0.08	(0.37, 0.68)	
	Pangasinense	0.51	0.08	(0.35, 0.67)	
	Tagalog	0.52	0.02	(0.49, 0.55)	
	Tausug	0.69	0.04	(0.60, 0.77)	
	Visayan/Bisaya	0.57	0.02	(0.52, 0.61)	
	Waray	0.52	0.05	(0.41, 0.62)	
	Zamboangueno	0.39	0.09	(0.21, 0.58)	

Table S15a. Nationally representative descriptive statistics for Poland

Characteristic	N = 10,389[1]
**Age group**	
18-24	955 (9.2%)
25-29	761 (7.3%)
30-39	2,159 (21%)
40-49	1,956 (19%)
50-59	1,670 (16%)
60-69	1,909 (18%)
70-79	833 (8.0%)
80 or older	145 (1.4%)
Missing	1 (<0.1%)
**Gender**	
Male	4,974 (48%)
Female	5,387 (52%)
Other	3 (<0.1%)
Missing	26 (0.2%)
**Marital status**	
Married	6,065 (58%)
Separated	111 (1.1%)
Divorced	529 (5.1%)
Widowed	990 (9.5%)
Never	1,811 (17%)
Domestic Partner	504 (4.8%)
Missing	379 (3.6%)
**Employment**	
Employed for an employer	5,837 (56%)
Self-employed	686 (6.6%)
Retired	2,434 (23%)
Student	515 (5.0%)
Homemaker	338 (3.3%)
Unemployed and looking for a job	284 (2.7%)
None of these/other	169 (1.6%)
Missing	126 (1.2%)
**Religious service attendance**	
>1/week	305 (2.9%)
1/week	3,263 (31%)
1-3/month	2,081 (20%)
A few times a year	3,064 (29%)
Never	1,597 (15%)
Missing	78 (0.8%)
**Education**	
up to 8 years	1,238 (12%)
9-15 years	6,130 (59%)
16+years	3,020 (29%)
Missing	1 (<0.1%)
**Immigration**	
Born in this country	10,258 (99%)
Born in another country	108 (1.0%)
Missing	23 (0.2%)
**Religious affiliation**	

Characteristic	N = 10,389[1]
Christianity	9,378 (90%)
Islam	2 (<0.1%)
Buddhism	2 (<0.1%)
Sikhism	1 (<0.1%)
Jainism	3 (<0.1%)
Shinto	1 (<0.1%)
Primal, Animist, or Folk religion	11 (0.1%)
No religion/Atheist/Agnostic	942 (9.1%)
Missing	50 (0.5%)
**Race and ethnicity**	
Belarussian	2 (<0.1%)
German	4 (<0.1%)
Kashubians	3 (<0.1%)
Other	4 (<0.1%)
Polish	10,309 (99%)
Silesia	14 (0.1%)
Ukrainian	38 (0.4%)
Missing	14 (0.1%)

[1]n (%)

Table S15b. Means by demographic category for Poland

Variable	Category	Mean	SE	95% CI	Global p-value
Age group	18-24	0.37	0.03	(0.31, 0.43)	<0.001
	25-29	0.47	0.02	(0.43, 0.52)	
	30-39	0.51	0.02	(0.48, 0.55)	
	40-49	0.55	0.02	(0.51, 0.59)	
	50-59	0.63	0.02	(0.59, 0.67)	
	60-69	0.70	0.02	(0.66, 0.74)	
	70-79	0.67	0.03	(0.61, 0.74)	
	80 or older	0.72	0.07	(0.57, 0.86)	
Gender	Male	0.52	0.02	(0.48, 0.55)	<0.001
	Female	0.63	0.01	(0.60, 0.65)	
	Other	0.44	0.31	(-1.69, 2.57)	
Marital status	Married	0.62	0.02	(0.59, 0.65)	<0.001
	Separated	0.53	0.09	(0.36, 0.70)	
	Divorced	0.56	0.03	(0.49, 0.63)	
	Widowed	0.67	0.03	(0.60, 0.73)	
	Never	0.43	0.02	(0.39, 0.47)	
	Domestic Partner	0.37	0.03	(0.31, 0.42)	
Employment	Employed for an employer	0.56	0.01	(0.53, 0.59)	<0.001
	Self-employed	0.52	0.03	(0.46, 0.58)	
	Retired	0.71	0.02	(0.67, 0.74)	
	Student	0.31	0.04	(0.23, 0.38)	
	Homemaker	0.55	0.06	(0.44, 0.67)	
	Unemployed and looking for a job	0.45	0.07	(0.32, 0.59)	
	None of these/other	0.48	0.07	(0.36, 0.61)	
Religious service attendance	>1/week	0.86	0.04	(0.77, 0.94)	<0.001
	1/week	0.82	0.02	(0.79, 0.85)	
	1-3/month	0.65	0.02	(0.61, 0.70)	
	A few times a year	0.44	0.02	(0.40, 0.48)	
	Never	0.17	0.02	(0.14, 0.21)	
Education	up to 8 years	0.52	0.05	(0.43, 0.61)	<0.001

Variable	Category	Mean	SE	95% CI	Global p-value
	9-15 years	0.60	0.02	(0.57, 0.63)	
	16+years	0.55	0.02	(0.52, 0.58)	
Immigration status	Born in this country	0.58	0.01	(0.55, 0.60)	<0.001
	Born in another country	0.35	0.07	(0.21, 0.49)	
Religious affiliation	Christianity	0.60	0.01	(0.57, 0.63)	<0.001
	Islam	1.00			
	Buddhism	0.42			
	Primal, Animist, or Folk religion	0.15	0.09	(-0.06, 0.35)	
	No religion/Atheist/Agnostic	0.31	0.03	(0.26, 0.37)	
Race and ethnicity	Other	0.69	0.25	(-0.24, 1.61)	<0.001
	Belarussian	0.00			
	German	0.43	0.18	(-0.55, 1.41)	
	Kashubians	0.73			
	Polish	0.57	0.01	(0.55, 0.60)	
	Silesia	0.71	0.23	(0.21, 1.20)	
	Ukrainian	0.56	0.16	(0.24, 0.88)	

Table S16a. Nationally representative descriptive statistics for South Africa

Characteristic	N = 2,651[1]
**Age group**	
18-24	461 (17%)
25-29	364 (14%)
30-39	655 (25%)
40-49	522 (20%)
50-59	309 (12%)
60-69	195 (7.4%)
70-79	120 (4.5%)
80 or older	17 (0.6%)
Missing	9 (0.3%)
**Gender**	
Male	1,288 (49%)
Female	1,356 (51%)
Other	2 (<0.1%)
Missing	4 (0.2%)
**Marital status**	
Married	539 (20%)
Separated	76 (2.9%)
Divorced	51 (1.9%)
Widowed	133 (5.0%)
Never	1,561 (59%)
Domestic Partner	264 (10.0%)
Missing	28 (1.0%)
**Employment**	
Employed for an employer	569 (21%)
Self-employed	412 (16%)
Retired	243 (9.2%)
Student	204 (7.7%)
Homemaker	137 (5.2%)
Unemployed and looking for a job	1,008 (38%)
None of these/other	74 (2.8%)
Missing	3 (0.1%)
**Religious service attendance**	
>1/week	414 (16%)
1/week	891 (34%)
1-3/month	574 (22%)
A few times a year	431 (16%)
Never	334 (13%)
Missing	7 (0.3%)
**Education**	
up to 8 years	668 (25%)
9-15 years	1,796 (68%)
16+years	183 (6.9%)
Missing	4 (0.2%)
**Immigration**	
Born in this country	2,511 (95%)
Born in another country	139 (5.2%)
Missing	1 (<0.1%)
**Religious affiliation**	

Characteristic	N = 2,651[1]
Christianity	2,163 (82%)
Islam	62 (2.3%)
Hinduism	1 (<0.1%)
Buddhism	12 (0.5%)
Jainism	2 (<0.1%)
Shinto	2 (<0.1%)
Taoism	1 (<0.1%)
Primal, Animist, or Folk religion	127 (4.8%)
Some other religion	5 (0.2%)
No religion/Atheist/Agnostic	253 (9.6%)
Missing	23 (0.9%)
**Race and ethnicity**	
Asian/Indian	6 (0.2%)
Black	2,381 (90%)
Colored	252 (9.5%)
Other	1 (<0.1%)
White	8 (0.3%)
Missing	3 (0.1%)

[1]n (%)

Table S16b. Means by demographic category for South Africa

Variable	Category	Mean	SE	95% CI	Global p-value
Age group	18-24	0.49	0.03	(0.42, 0.56)	<0.001
	25-29	0.49	0.03	(0.43, 0.55)	
	30-39	0.50	0.02	(0.45, 0.55)	
	40-49	0.50	0.03	(0.45, 0.56)	
	50-59	0.62	0.04	(0.54, 0.70)	
	60-69	0.58	0.06	(0.47, 0.70)	
	70-79	0.75	0.07	(0.62, 0.88)	
	80 or older	0.41	0.23	(-0.08, 0.91)	
Gender	Male	0.53	0.02	(0.49, 0.57)	1.000
	Female	0.52	0.02	(0.49, 0.56)	
	Other	0.58			
Marital status	Married	0.63	0.03	(0.58, 0.69)	<0.001
	Separated	0.43	0.07	(0.28, 0.57)	
	Divorced	0.47	0.10	(0.28, 0.66)	
	Widowed	0.58	0.07	(0.45, 0.72)	
	Never	0.51	0.02	(0.47, 0.54)	
	Domestic Partner	0.44	0.04	(0.36, 0.52)	
Employment	Employed for an employer	0.52	0.03	(0.46, 0.58)	0.126
	Self-employed	0.54	0.03	(0.48, 0.60)	
	Retired	0.64	0.05	(0.54, 0.75)	
	Student	0.49	0.06	(0.38, 0.60)	
	Homemaker	0.50	0.06	(0.38, 0.62)	
	Unemployed and looking for a job	0.51	0.02	(0.47, 0.55)	
	None of these/other	0.63	0.10	(0.43, 0.82)	
Religious service attendance	>1/week	0.60	0.03	(0.53, 0.67)	<0.001
	1/week	0.57	0.02	(0.52, 0.61)	
	1-3/month	0.49	0.03	(0.43, 0.56)	
	A few times a year	0.46	0.03	(0.39, 0.52)	
	Never	0.48	0.04	(0.41, 0.55)	
Education	up to 8 years	0.59	0.04	(0.52, 0.66)	0.015

Variable	Category	Mean	SE	95% CI	Global p-value
	9-15 years	0.51	0.02	(0.48, 0.54)	
	16+years	0.48	0.04	(0.40, 0.56)	
Immigration status	Born in this country	0.53	0.02	(0.50, 0.56)	0.998
	Born in another country	0.54	0.06	(0.42, 0.66)	
Religious affiliation	Christianity	0.52	0.02	(0.49, 0.56)	<0.001
	Islam	0.68	0.08	(0.52, 0.83)	
	Buddhism	0.57	0.14	(0.26, 0.89)	
	Shinto	0.00			
	Primal, Animist, or Folk religion	0.51	0.06	(0.38, 0.63)	
	Some other religion	0.30	0.20	(-0.34, 0.95)	
	No religion/Atheist/Agnostic	0.53	0.04	(0.46, 0.60)	
Race and ethnicity	Black	0.52	0.02	(0.49, 0.55)	<0.001
	White	0.75	0.20	(0.26, 1.24)	
	Asian/Indian	0.14	0.18	(-0.37, 0.66)	
	Colored	0.61	0.06	(0.49, 0.73)	

Table S17a. Nationally representative descriptive statistics for Spain

Characteristic	N = 6,290[1]
**Age group**	
18-24	594 (9.4%)
25-29	450 (7.2%)
30-39	1,111 (18%)
40-49	1,396 (22%)
50-59	1,252 (20%)
60-69	977 (16%)
70-79	467 (7.4%)
80 or older	43 (0.7%)
Missing	0 (0%)
**Gender**	
Male	3,142 (50%)
Female	3,119 (50%)
Other	6 (0.1%)
Missing	22 (0.4%)
**Marital status**	
Married	2,947 (47%)
Separated	237 (3.8%)
Divorced	518 (8.2%)
Widowed	189 (3.0%)
Never	1,742 (28%)
Domestic Partner	589 (9.4%)
Missing	67 (1.1%)
**Employment**	
Employed for an employer	2,862 (45%)
Self-employed	576 (9.2%)
Retired	1,278 (20%)
Student	448 (7.1%)
Homemaker	345 (5.5%)
Unemployed and looking for a job	646 (10%)
None of these/other	123 (2.0%)
Missing	11 (0.2%)
**Religious service attendance**	
>1/week	317 (5.0%)
1/week	662 (11%)
1-3/month	437 (6.9%)
A few times a year	1,972 (31%)
Never	2,875 (46%)
Missing	27 (0.4%)
**Education**	
up to 8 years	802 (13%)
9-15 years	4,145 (66%)
16+years	1,341 (21%)
Missing	2 (<0.1%)
**Immigration**	
Born in this country	5,479 (87%)
Born in another country	788 (13%)
Missing	23 (0.4%)
**Religious affiliation**	

**Characteristic**	**N = 6,290**[1]
Christianity	4,074 (65%)
Islam	135 (2.1%)
Hinduism	7 (0.1%)
Buddhism	36 (0.6%)
Judaism	4 (<0.1%)
Sikhism	3 (<0.1%)
Baha'i	2 (<0.1%)
Jainism	1 (<0.1%)
Taoism	5 (<0.1%)
Confucianism	3 (<0.1%)
Primal, Animist, or Folk religion	7 (0.1%)
Some other religion	27 (0.4%)
No religion/Atheist/Agnostic	1,932 (31%)
Missing	55 (0.9%)
**Race and ethnicity**	
Missing	6,290 (100%)

[1]n (%)

Table S17b. Means by demographic category for Spain

Variable	Category	Mean	SE	95% CI	Global p-value
Age group	18-24	0.44	0.03	(0.39, 0.49)	0.016
	25-29	0.43	0.03	(0.38, 0.49)	
	30-39	0.42	0.02	(0.39, 0.45)	
	40-49	0.42	0.01	(0.39, 0.45)	
	50-59	0.39	0.02	(0.35, 0.42)	
	60-69	0.35	0.03	(0.30, 0.40)	
	70-79	0.36	0.04	(0.28, 0.44)	
	80 or older	0.46	0.12	(0.22, 0.70)	
Gender	Male	0.39	0.01	(0.37, 0.42)	0.940
	Female	0.41	0.01	(0.38, 0.43)	
	Other	0.50	0.18	(0.00, 1.00)	
Marital status	Married	0.40	0.01	(0.38, 0.43)	0.421
	Separated	0.44	0.04	(0.36, 0.53)	
	Divorced	0.35	0.03	(0.29, 0.40)	
	Widowed	0.38	0.06	(0.27, 0.49)	
	Never	0.41	0.01	(0.38, 0.44)	
	Domestic Partner	0.40	0.02	(0.36, 0.45)	
Employment	Employed for an employer	0.40	0.01	(0.38, 0.42)	0.645
	Self-employed	0.41	0.02	(0.36, 0.45)	
	Retired	0.37	0.02	(0.33, 0.42)	
	Student	0.42	0.03	(0.36, 0.48)	
	Homemaker	0.47	0.04	(0.39, 0.55)	
	Unemployed and looking for a job	0.41	0.02	(0.37, 0.45)	
	None of these/other	0.38	0.05	(0.28, 0.48)	
Religious service attendance	>1/week	0.68	0.04	(0.61, 0.75)	<0.001
	1/week	0.63	0.03	(0.58, 0.68)	
	1-3/month	0.54	0.03	(0.48, 0.60)	
	A few times a year	0.45	0.02	(0.42, 0.48)	
	Never	0.26	0.01	(0.24, 0.28)	
Education	up to 8 years	0.42	0.03	(0.36, 0.48)	0.014

Variable	Category	Mean	SE	95% CI	Global p-value
	9-15 years	0.41	0.01	(0.39, 0.43)	
	16+years	0.37	0.02	(0.34, 0.40)	
Immigration status	Born in this country	0.38	0.01	(0.36, 0.40)	<0.001
	Born in another country	0.54	0.02	(0.50, 0.58)	
Religious affiliation	Christianity	0.49	0.01	(0.47, 0.51)	<0.001
	Islam	0.67	0.05	(0.57, 0.78)	
	Hinduism	0.83	0.15	(0.41, 1.24)	
	Buddhism	0.43	0.11	(0.20, 0.66)	
	Judaism	0.70	0.21	(-0.09, 1.49)	
	Sikhism	0.12	0.12	(-0.83, 1.06)	
	Baha'i	0.40			
	Taoism	0.00			
	Confucianism	0.77			
	Primal, Animist, or Folk religion	0.82	0.12	(0.47, 1.18)	
	Some other religion	0.52	0.13	(0.24, 0.80)	
	No religion/Atheist/Agnostic	0.19	0.01	(0.17, 0.21)	

Table S18a. Nationally representative descriptive statistics for Sweden

Characteristic	N = 15,068[1]
**Age group**	
18-24	1,515 (10%)
25-29	1,399 (9.3%)
30-39	2,398 (16%)
40-49	2,221 (15%)
50-59	2,493 (17%)
60-69	2,168 (14%)
70-79	2,253 (15%)
80 or older	621 (4.1%)
Missing	0 (0%)
**Gender**	
Male	7,536 (50%)
Female	7,493 (50%)
Other	27 (0.2%)
Missing	12 (<0.1%)
**Marital status**	
Married	6,408 (43%)
Separated	426 (2.8%)
Divorced	801 (5.3%)
Widowed	433 (2.9%)
Never	3,854 (26%)
Domestic Partner	3,073 (20%)
Missing	72 (0.5%)
**Employment**	
Employed for an employer	7,907 (52%)
Self-employed	1,243 (8.3%)
Retired	3,832 (25%)
Student	1,332 (8.8%)
Homemaker	75 (0.5%)
Unemployed and looking for a job	324 (2.2%)
None of these/other	337 (2.2%)
Missing	18 (0.1%)
**Religious service attendance**	
>1/week	236 (1.6%)
1/week	434 (2.9%)
1-3/month	486 (3.2%)
A few times a year	3,950 (26%)
Never	9,918 (66%)
Missing	45 (0.3%)
**Education**	
up to 8 years	252 (1.7%)
9-15 years	10,790 (72%)
16+years	4,026 (27%)
Missing	0 (0%)
**Immigration**	
Born in this country	13,922 (92%)
Born in another country	1,052 (7.0%)
Missing	94 (0.6%)
**Religious affiliation**	

**Characteristic**	**N = 15,068**[1]
Christianity	8,346 (55%)
Islam	470 (3.1%)
Hinduism	22 (0.1%)
Buddhism	110 (0.7%)
Judaism	54 (0.4%)
Sikhism	4 (<0.1%)
Baha'i	6 (<0.1%)
Shinto	0 (<0.1%)
Taoism	4 (<0.1%)
Primal, Animist, or Folk religion	83 (0.5%)
Some other religion	198 (1.3%)
No religion/Atheist/Agnostic	5,697 (38%)
Missing	74 (0.5%)
**Race and ethnicity**	
Missing	15,068 (100%)

[1]n (%)

Table S18b. Means by demographic category for Sweden

Variable	Category	Mean	SE	95% CI	Global p-value
Age group	18-24	0.38	0.01	(0.36, 0.41)	<0.001
	25-29	0.38	0.02	(0.35, 0.41)	
	30-39	0.28	0.01	(0.26, 0.31)	
	40-49	0.29	0.01	(0.27, 0.31)	
	50-59	0.30	0.01	(0.28, 0.33)	
	60-69	0.28	0.01	(0.26, 0.30)	
	70-79	0.22	0.01	(0.20, 0.25)	
	80 or older	0.17	0.02	(0.13, 0.21)	
Gender	Male	0.23	0.01	(0.21, 0.24)	<0.001
	Female	0.36	0.01	(0.35, 0.37)	
	Other	0.18	0.09	(-0.01, 0.37)	
Marital status	Married	0.28	0.01	(0.27, 0.30)	<0.001
	Separated	0.31	0.03	(0.26, 0.37)	
	Divorced	0.32	0.02	(0.28, 0.36)	
	Widowed	0.26	0.02	(0.21, 0.30)	
	Never	0.32	0.01	(0.30, 0.34)	
	Domestic Partner	0.28	0.01	(0.26, 0.30)	
Employment	Employed for an employer	0.29	0.01	(0.28, 0.30)	<0.001
	Self-employed	0.31	0.02	(0.28, 0.35)	
	Retired	0.25	0.01	(0.23, 0.26)	
	Student	0.39	0.02	(0.36, 0.42)	
	Homemaker	0.36	0.07	(0.22, 0.49)	
	Unemployed and looking for a job	0.37	0.03	(0.31, 0.43)	
	None of these/other	0.38	0.03	(0.32, 0.44)	
Religious service attendance	>1/week	0.76	0.04	(0.69, 0.84)	<0.001
	1/week	0.83	0.02	(0.78, 0.87)	
	1-3/month	0.69	0.03	(0.64, 0.74)	
	A few times a year	0.36	0.01	(0.34, 0.38)	
	Never	0.21	0.01	(0.20, 0.22)	
Education	up to 8 years	0.33	0.04	(0.25, 0.41)	<0.001

Variable	Category	Mean	SE	95% CI	Global p-value
	9-15 years	0.30	0.01	(0.29, 0.31)	
	16+years	0.27	0.01	(0.25, 0.29)	
Immigration status	Born in this country	0.28	0.00	(0.27, 0.29)	<0.001
	Born in another country	0.41	0.02	(0.37, 0.45)	
Religious affiliation	Christianity	0.36	0.01	(0.34, 0.37)	<0.001
	Islam	0.70	0.03	(0.64, 0.76)	
	Hinduism	0.77	0.14	(0.47, 1.07)	
	Buddhism	0.57	0.07	(0.42, 0.71)	
	Judaism	0.18	0.09	(0.00, 0.35)	
	Sikhism	0.45	0.35	(-0.98, 1.88)	
	Baha'i	0.44	0.30	(-0.41, 1.29)	
	Taoism	1.00			
	Primal, Animist, or Folk religion	0.45	0.08	(0.29, 0.62)	
	Some other religion	0.58	0.05	(0.47, 0.68)	
	No religion/Atheist/Agnostic	0.15	0.01	(0.14, 0.16)	

Table S19a. Nationally representative descriptive statistics for Tanzania

Characteristic	N = 9,075[1]
**Age group**	
18-24	2,284 (25%)
25-29	1,349 (15%)
30-39	2,060 (23%)
40-49	1,503 (17%)
50-59	912 (10%)
60-69	575 (6.3%)
70-79	297 (3.3%)
80 or older	93 (1.0%)
Missing	2 (<0.1%)
**Gender**	
Male	4,299 (47%)
Female	4,776 (53%)
Other	0 (0%)
Missing	0 (0%)
**Marital status**	
Married	5,577 (61%)
Separated	404 (4.5%)
Divorced	103 (1.1%)
Widowed	450 (5.0%)
Never	2,260 (25%)
Domestic Partner	275 (3.0%)
Missing	7 (<0.1%)
**Employment**	
Employed for an employer	513 (5.6%)
Self-employed	4,625 (51%)
Retired	139 (1.5%)
Student	319 (3.5%)
Homemaker	1,796 (20%)
Unemployed and looking for a job	1,491 (16%)
None of these/other	186 (2.1%)
Missing	6 (<0.1%)
**Religious service attendance**	
>1/week	2,622 (29%)
1/week	4,268 (47%)
1-3/month	1,082 (12%)
A few times a year	814 (9.0%)
Never	288 (3.2%)
Missing	1 (<0.1%)
**Education**	
up to 8 years	6,699 (74%)
9-15 years	2,252 (25%)
16+years	122 (1.3%)
Missing	2 (<0.1%)
**Immigration**	
Born in this country	9,048 (100%)
Born in another country	25 (0.3%)
Missing	1 (<0.1%)
**Religious affiliation**	

**Characteristic**	**N = 9,075**[1]
Christianity	5,647 (62%)
Islam	3,189 (35%)
Taoism	1 (<0.1%)
Primal, Animist, or Folk religion	12 (0.1%)
No religion/Atheist/Agnostic	216 (2.4%)
Missing	10 (0.1%)
**Race and ethnicity**	
African	9,060 (100%)
Arab	11 (0.1%)
Indian	3 (<0.1%)
Missing	2 (<0.1%)

[1] n (%)

Table S19b. Means by demographic category for Tanzania

Variable	Category	Mean	SE	95% CI	Global p-value
Age group	18-24	0.55	0.02	(0.52, 0.58)	<0.001
	25-29	0.59	0.02	(0.56, 0.63)	
	30-39	0.62	0.01	(0.59, 0.65)	
	40-49	0.64	0.02	(0.61, 0.68)	
	50-59	0.65	0.02	(0.61, 0.70)	
	60-69	0.65	0.03	(0.59, 0.71)	
	70-79	0.62	0.05	(0.53, 0.72)	
	80 or older	0.63	0.08	(0.48, 0.79)	
Gender	Male	0.64	0.01	(0.61, 0.66)	<0.001
	Female	0.58	0.01	(0.56, 0.61)	
Marital status	Married	0.63	0.01	(0.61, 0.65)	<0.001
	Separated	0.57	0.03	(0.51, 0.63)	
	Divorced	0.59	0.06	(0.48, 0.71)	
	Widowed	0.58	0.04	(0.51, 0.66)	
	Never	0.58	0.02	(0.55, 0.61)	
	Domestic Partner	0.50	0.04	(0.42, 0.58)	
Employment	Employed for an employer	0.67	0.03	(0.62, 0.72)	0.291
	Self-employed	0.61	0.01	(0.58, 0.63)	
	Retired	0.67	0.05	(0.57, 0.77)	
	Student	0.63	0.03	(0.57, 0.69)	
	Homemaker	0.59	0.02	(0.56, 0.63)	
	Unemployed and looking for a job	0.60	0.02	(0.57, 0.64)	
	None of these/other	0.60	0.06	(0.49, 0.71)	
Religious service attendance	>1/week	0.70	0.01	(0.68, 0.73)	<0.001
	1/week	0.60	0.01	(0.57, 0.63)	
	1-3/month	0.51	0.02	(0.46, 0.55)	
	A few times a year	0.53	0.02	(0.48, 0.57)	
	Never	0.48	0.04	(0.40, 0.56)	
Education	up to 8 years	0.60	0.01	(0.57, 0.62)	<0.001
	9-15 years	0.63	0.01	(0.61, 0.66)	

Variable	Category	Mean	SE	95% CI	Global p-value
	16+years	0.74	0.04	(0.66, 0.82)	
Immigration status	Born in this country	0.61	0.01	(0.59, 0.63)	0.951
	Born in another country	0.55	0.14	(0.27, 0.83)	
Religious affiliation	Christianity	0.59	0.01	(0.57, 0.62)	0.014
	Islam	0.64	0.02	(0.60, 0.67)	
	Primal, Animist, or Folk religion	0.44	0.18	(0.05, 0.84)	
	No religion/Atheist/Agnostic	0.54	0.06	(0.43, 0.65)	
Race and ethnicity	Indian	1.00			<0.001
	Arab	0.62	0.12	(0.34, 0.91)	
	African	0.61	0.01	(0.59, 0.63)	

Table S20a. Nationally representative descriptive statistics for Turkey

Characteristic	N = 1,473[1]
**Age group**	
18-24	222 (15%)
25-29	152 (10%)
30-39	315 (21%)
40-49	312 (21%)
50-59	225 (15%)
60-69	164 (11%)
70-79	65 (4.4%)
80 or older	18 (1.2%)
Missing	0 (0%)
**Gender**	
Male	754 (51%)
Female	719 (49%)
Other	0 (0%)
Missing	0 (0%)
**Marital status**	
Married	936 (64%)
Separated	13 (0.9%)
Divorced	64 (4.3%)
Widowed	64 (4.3%)
Never	379 (26%)
Domestic Partner	0 (0%)
Missing	17 (1.1%)
**Employment**	
Employed for an employer	413 (28%)
Self-employed	255 (17%)
Retired	205 (14%)
Student	107 (7.3%)
Homemaker	347 (24%)
Unemployed and looking for a job	87 (5.9%)
None of these/other	59 (4.0%)
Missing	0 (0%)
**Religious service attendance**	
>1/week	493 (33%)
1/week	271 (18%)
1-3/month	174 (12%)
A few times a year	255 (17%)
Never	274 (19%)
Missing	6 (0.4%)
**Education**	
up to 8 years	436 (30%)
9-15 years	711 (48%)
16+years	326 (22%)
Missing	0 (0%)
**Immigration**	
Born in this country	1,415 (96%)
Born in another country	58 (4.0%)
Missing	0 (0%)
**Religious affiliation**	

**Characteristic**	**N = 1,473**[1]
Christianity	2 (0.1%)
Islam	1,381 (94%)
Buddhism	1 (<0.1%)
Judaism	1 (<0.1%)
Sikhism	1 (<0.1%)
Primal, Animist, or Folk religion	1 (<0.1%)
Some other religion	1 (<0.1%)
No religion/Atheist/Agnostic	66 (4.5%)
Missing	19 (1.3%)
**Race and ethnicity**	
Albanian	8 (0.5%)
Arab	51 (3.5%)
Armenian	1 (<0.1%)
Azeri	9 (0.6%)
Bosnian	5 (0.3%)
Circassian	19 (1.3%)
Georgian	4 (0.3%)
Greek	1 (<0.1%)
Kurdish/Zaza	252 (17%)
Laz	25 (1.7%)
Other	58 (3.9%)
Turkish	1,030 (70%)
Uyghur	1 (<0.1%)
Missing	9 (0.6%)

[1]n (%)

Table S20b. Means by demographic category for Turkey

Variable	Category	Mean	SE	95% CI	Global p-value
Age group	18-24	0.59	0.03	(0.52, 0.65)	<0.001
	25-29	0.64	0.04	(0.56, 0.73)	
	30-39	0.68	0.03	(0.63, 0.74)	
	40-49	0.66	0.03	(0.61, 0.72)	
	50-59	0.75	0.04	(0.67, 0.82)	
	60-69	0.74	0.05	(0.63, 0.84)	
	70-79	0.53	0.11	(0.32, 0.74)	
	80 or older	0.68	0.19	(0.28, 1.08)	
Gender	Male	0.66	0.02	(0.63, 0.70)	0.984
	Female	0.67	0.02	(0.63, 0.72)	
Marital status	Married	0.70	0.02	(0.67, 0.74)	<0.001
	Separated	0.77	0.10	(0.54, 1.00)	
	Divorced	0.48	0.07	(0.33, 0.62)	
	Widowed	0.74	0.08	(0.57, 0.91)	
	Never	0.60	0.03	(0.55, 0.65)	
Employment	Employed for an employer	0.67	0.02	(0.62, 0.72)	0.014
	Self-employed	0.64	0.03	(0.58, 0.70)	
	Retired	0.70	0.05	(0.61, 0.79)	
	Student	0.58	0.04	(0.49, 0.67)	
	Homemaker	0.71	0.03	(0.64, 0.78)	
	Unemployed and looking for a job	0.57	0.06	(0.46, 0.68)	
	None of these/other	0.73	0.07	(0.59, 0.87)	
Religious service attendance	>1/week	0.87	0.02	(0.83, 0.91)	<0.001
	1/week	0.70	0.03	(0.63, 0.77)	
	1-3/month	0.65	0.05	(0.56, 0.74)	
	A few times a year	0.58	0.03	(0.51, 0.65)	
	Never	0.37	0.04	(0.30, 0.44)	
Education	up to 8 years	0.73	0.03	(0.67, 0.80)	<0.001
	9-15 years	0.67	0.02	(0.63, 0.70)	
	16+years	0.59	0.02	(0.54, 0.63)	

Variable	Category	Mean	SE	95% CI	Global p-value
Immigration status	Born in this country	0.67	0.01	(0.65, 0.70)	0.004
	Born in another country	0.52	0.08	(0.36, 0.69)	
Religious affiliation	Christianity	0.65			<0.001
	Islam	0.70	0.01	(0.67, 0.73)	
	Buddhism	0.00			
	Primal, Animist, or Folk religion	0.42			
	Some other religion	0.00			
	No religion/Atheist/Agnostic	0.11	0.03	(0.05, 0.18)	
Race and ethnicity	Other	0.57	0.09	(0.40, 0.74)	<0.001
	Arab	0.58	0.09	(0.40, 0.76)	
	Greek	0.43			
	Turkish	0.67	0.02	(0.64, 0.71)	
	Albanian	0.92	0.08	(0.71, 1.13)	
	Armenian	1.00			
	Azeri	0.42	0.21	(-0.08, 0.91)	
	Bosnian	0.24	0.17	(-0.34, 0.82)	
	Circassian	0.66	0.12	(0.40, 0.92)	
	Georgian	0.32	0.20	(-0.52, 1.16)	
	Kurdish/Zaza	0.68	0.04	(0.62, 0.75)	
	Laz	0.81	0.09	(0.63, 0.99)	

Table S21a. Nationally representative descriptive statistics for United Kingdom

Characteristic	N = 5,368[1]
**Age group**	
18-24	490 (9.1%)
25-29	391 (7.3%)
30-39	946 (18%)
40-49	827 (15%)
50-59	949 (18%)
60-69	889 (17%)
70-79	711 (13%)
80 or older	163 (3.0%)
Missing	1 (<0.1%)
**Gender**	
Male	2,557 (48%)
Female	2,789 (52%)
Other	14 (0.3%)
Missing	9 (0.2%)
**Marital status**	
Married	2,510 (47%)
Separated	114 (2.1%)
Divorced	435 (8.1%)
Widowed	294 (5.5%)
Never	1,456 (27%)
Domestic Partner	512 (9.5%)
Missing	48 (0.9%)
**Employment**	
Employed for an employer	2,798 (52%)
Self-employed	469 (8.7%)
Retired	1,262 (24%)
Student	229 (4.3%)
Homemaker	184 (3.4%)
Unemployed and looking for a job	215 (4.0%)
None of these/other	201 (3.7%)
Missing	11 (0.2%)
**Religious service attendance**	
>1/week	291 (5.4%)
1/week	499 (9.3%)
1-3/month	293 (5.5%)
A few times a year	1,165 (22%)
Never	3,110 (58%)
Missing	10 (0.2%)
**Education**	
up to 8 years	1,314 (24%)
9-15 years	2,072 (39%)
16+years	1,974 (37%)
Missing	8 (0.2%)
**Immigration**	
Born in this country	4,659 (87%)
Born in another country	682 (13%)
Missing	27 (0.5%)
**Religious affiliation**	

Characteristic	N = 5,368[1]
Christianity	2,750 (51%)
Islam	218 (4.1%)
Hinduism	61 (1.1%)
Buddhism	30 (0.6%)
Judaism	44 (0.8%)
Sikhism	29 (0.5%)
Baha'i	6 (0.1%)
Jainism	4 (<0.1%)
Taoism	4 (<0.1%)
Confucianism	2 (<0.1%)
Primal, Animist, or Folk religion	36 (0.7%)
Some other religion	61 (1.1%)
No religion/Atheist/Agnostic	2,099 (39%)
Missing	25 (0.5%)
**Race and ethnicity**	
Asian	426 (7.9%)
Black	152 (2.8%)
Other	96 (1.8%)
White	4,647 (87%)
Missing	47 (0.9%)

[1]n (%)

Table S21b. Means by demographic category for United Kingdom

Variable	Category	Mean	SE	95% CI	Global p-value
Age group	18-24	0.42	0.04	(0.35, 0.49)	<0.001
	25-29	0.48	0.04	(0.41, 0.55)	
	30-39	0.43	0.02	(0.38, 0.47)	
	40-49	0.43	0.02	(0.38, 0.47)	
	50-59	0.37	0.02	(0.32, 0.41)	
	60-69	0.33	0.02	(0.29, 0.37)	
	70-79	0.33	0.03	(0.29, 0.38)	
	80 or older	0.43	0.06	(0.32, 0.54)	
Gender	Male	0.37	0.01	(0.34, 0.39)	<0.001
	Female	0.41	0.01	(0.39, 0.44)	
	Other	0.29	0.17	(-0.09, 0.66)	
Marital status	Married	0.42	0.01	(0.39, 0.45)	<0.001
	Separated	0.29	0.05	(0.19, 0.39)	
	Divorced	0.37	0.03	(0.31, 0.44)	
	Widowed	0.43	0.04	(0.36, 0.51)	
	Never	0.37	0.02	(0.34, 0.41)	
	Domestic Partner	0.30	0.03	(0.25, 0.36)	
Employment	Employed for an employer	0.41	0.01	(0.38, 0.44)	<0.001
	Self-employed	0.38	0.03	(0.32, 0.44)	
	Retired	0.33	0.02	(0.29, 0.37)	
	Student	0.44	0.05	(0.35, 0.54)	
	Homemaker	0.48	0.05	(0.37, 0.59)	
	Unemployed and looking for a job	0.37	0.05	(0.27, 0.47)	
	None of these/other	0.40	0.05	(0.30, 0.49)	
Religious service attendance	>1/week	0.83	0.03	(0.76, 0.89)	<0.001
	1/week	0.76	0.03	(0.71, 0.82)	
	1-3/month	0.69	0.04	(0.61, 0.76)	
	A few times a year	0.45	0.02	(0.41, 0.49)	
	Never	0.24	0.01	(0.22, 0.26)	
Education	up to 8 years	0.36	0.03	(0.31, 0.41)	<0.001

Variable	Category	Mean	SE	95% CI	Global p-value
	9-15 years	0.37	0.01	(0.35, 0.40)	
	16+years	0.43	0.01	(0.41, 0.46)	
Immigration status	Born in this country	0.38	0.01	(0.36, 0.40)	<0.001
	Born in another country	0.48	0.03	(0.43, 0.53)	
Religious affiliation	Christianity	0.49	0.01	(0.46, 0.52)	<0.001
	Islam	0.82	0.04	(0.73, 0.90)	
	Hinduism	0.65	0.09	(0.48, 0.83)	
	Buddhism	0.34	0.09	(0.15, 0.53)	
	Judaism	0.30	0.09	(0.12, 0.48)	
	Sikhism	0.85	0.07	(0.70, 1.00)	
	Baha'i	0.15	0.15	(-0.30, 0.60)	
	Taoism	0.68	0.26	(-0.36, 1.72)	
	Primal, Animist, or Folk religion	0.51	0.13	(0.24, 0.78)	
	Some other religion	0.50	0.08	(0.35, 0.66)	
	No religion/Atheist/Agnostic	0.20	0.01	(0.17, 0.22)	
Race and ethnicity	Asian	0.66	0.03	(0.60, 0.73)	<0.001
	Black	0.62	0.05	(0.52, 0.73)	
	Other	0.60	0.07	(0.46, 0.74)	
	White	0.35	0.01	(0.33, 0.37)	

Table S22a. Nationally representative descriptive statistics for United States

Characteristic	N = 38,312[1]
**Age group**	
18-24	2,682 (7.0%)
25-29	3,540 (9.2%)
30-39	7,284 (19%)
40-49	5,649 (15%)
50-59	6,745 (18%)
60-69	6,832 (18%)
70-79	4,054 (11%)
80 or older	1,525 (4.0%)
Missing	0 (0%)
**Gender**	
Male	18,222 (48%)
Female	19,562 (51%)
Other	392 (1.0%)
Missing	136 (0.4%)
**Marital status**	
Married	20,360 (53%)
Separated	727 (1.9%)
Divorced	3,636 (9.5%)
Widowed	1,978 (5.2%)
Never	9,431 (25%)
Domestic Partner	1,971 (5.1%)
Missing	207 (0.5%)
**Employment**	
Employed for an employer	19,502 (51%)
Self-employed	3,445 (9.0%)
Retired	9,016 (24%)
Student	1,145 (3.0%)
Homemaker	2,049 (5.3%)
Unemployed and looking for a job	1,777 (4.6%)
None of these/other	1,292 (3.4%)
Missing	87 (0.2%)
**Religious service attendance**	
>1/week	2,633 (6.9%)
1/week	5,887 (15%)
1-3/month	2,819 (7.4%)
A few times a year	8,870 (23%)
Never	17,975 (47%)
Missing	128 (0.3%)
**Education**	
up to 8 years	210 (0.5%)
9-15 years	25,322 (66%)
16+years	12,705 (33%)
Missing	75 (0.2%)
**Immigration**	
Born in this country	34,865 (91%)
Born in another country	3,020 (7.9%)
Missing	427 (1.1%)
**Religious affiliation**	

Characteristic	N = 38,312[1]
Christianity	22,954 (60%)
Islam	205 (0.5%)
Hinduism	167 (0.4%)
Buddhism	336 (0.9%)
Judaism	638 (1.7%)
Sikhism	24 (<0.1%)
Baha'i	13 (<0.1%)
Jainism	18 (<0.1%)
Shinto	12 (<0.1%)
Taoism	93 (0.2%)
Confucianism	8 (<0.1%)
Primal, Animist, or Folk religion	240 (0.6%)
Some other religion	1,267 (3.3%)
No religion/Atheist/Agnostic	11,870 (31%)
Missing	467 (1.2%)
**Race and ethnicity**	
Asian	2,466 (6.4%)
Black	4,501 (12%)
Hispanic	6,724 (18%)
Other	997 (2.6%)
White	23,605 (62%)
Missing	20 (<0.1%)

[1] n (%)

Table S22b. Means by demographic category for United States

Variable	Category	Mean	SE	95% CI	Global p-value
Age group	18-24	0.52	0.04	(0.44, 0.59)	<0.001
	25-29	0.46	0.03	(0.41, 0.52)	
	30-39	0.49	0.02	(0.46, 0.52)	
	40-49	0.53	0.01	(0.51, 0.56)	
	50-59	0.61	0.01	(0.59, 0.63)	
	60-69	0.63	0.01	(0.61, 0.64)	
	70-79	0.59	0.01	(0.57, 0.61)	
	80 or older	0.60	0.02	(0.55, 0.64)	
Gender	Male	0.51	0.01	(0.50, 0.53)	<0.001
	Female	0.60	0.01	(0.59, 0.62)	
	Other	0.30	0.09	(0.12, 0.48)	
Marital status	Married	0.59	0.01	(0.58, 0.60)	<0.001
	Separated	0.51	0.06	(0.39, 0.64)	
	Divorced	0.57	0.01	(0.54, 0.59)	
	Widowed	0.67	0.02	(0.63, 0.70)	
	Never	0.48	0.02	(0.45, 0.51)	
	Domestic Partner	0.45	0.03	(0.39, 0.51)	
Employment	Employed for an employer	0.54	0.01	(0.53, 0.56)	<0.001
	Self-employed	0.55	0.02	(0.51, 0.60)	
	Retired	0.60	0.01	(0.58, 0.61)	
	Student	0.38	0.05	(0.27, 0.48)	
	Homemaker	0.65	0.02	(0.60, 0.69)	
	Unemployed and looking for a job	0.47	0.05	(0.38, 0.57)	
	None of these/other	0.60	0.04	(0.52, 0.68)	
Religious service attendance	>1/week	0.92	0.01	(0.89, 0.94)	<0.001
	1/week	0.87	0.01	(0.84, 0.89)	
	1-3/month	0.76	0.02	(0.72, 0.80)	
	A few times a year	0.64	0.01	(0.61, 0.66)	
	Never	0.33	0.01	(0.31, 0.35)	
Education	up to 8 years	0.60	0.16	(0.28, 0.92)	<0.001

Variable	Category	Mean	SE	95% CI	Global p-value
	9-15 years	0.58	0.01	(0.56, 0.60)	
	16+years	0.50	0.01	(0.49, 0.51)	
Immigration status	Born in this country	0.56	0.01	(0.55, 0.57)	<0.001
	Born in another country	0.49	0.03	(0.44, 0.54)	
Religious affiliation	Christianity	0.76	0.01	(0.75, 0.77)	<0.001
	Islam	0.73	0.08	(0.57, 0.89)	
	Hinduism	0.49	0.07	(0.35, 0.63)	
	Buddhism	0.53	0.06	(0.42, 0.64)	
	Judaism	0.28	0.03	(0.23, 0.34)	
	Sikhism	0.55	0.16	(0.22, 0.87)	
	Baha'i	0.91	0.07	(0.76, 1.06)	
	Jainism	0.83	0.13	(0.55, 1.11)	
	Shinto	0.69	0.17	(0.29, 1.08)	
	Taoism	0.28	0.13	(0.02, 0.54)	
	Confucianism	0.18	0.12	(-0.12, 0.48)	
	Primal, Animist, or Folk religion	0.44	0.13	(0.19, 0.69)	
	Some other religion	0.58	0.04	(0.50, 0.66)	
	No religion/Atheist/Agnostic	0.17	0.01	(0.15, 0.19)	
Race and ethnicity	Asian	0.40	0.02	(0.36, 0.45)	<0.001
	Black	0.66	0.02	(0.62, 0.70)	
	Other	0.64	0.03	(0.59, 0.70)	
	White	0.56	0.01	(0.55, 0.57)	
	Hispanic	0.53	0.02	(0.48, 0.57)	

Online Supplement

Population Weighted Meta-Analysis and Forest Plots

Population Weighted Meta-Analysis

Table S23. Population weighted meta-analysis of results demographic group means.

Variable	Category	Proportion	95% CI of Proportion	SE Analogue (CI Width/4)
Age group				
	18-24	0.56	(0.53,0.59)	0.01
	25-29	0.57	(0.54,0.59)	0.01
	30-39	0.56	(0.54,0.59)	0.01
	40-49	0.57	(0.54,0.60)	0.01
	50-59	0.59	(0.56,0.62)	0.01
	60-69	0.56	(0.53,0.59)	0.01
	70-79	0.56	(0.53,0.58)	0.01
	80 or older	0.73	(0.69,0.76)	0.02
Gender				
	Male	0.56	(0.53,0.59)	0.01
	Female	0.58	(0.55,0.60)	0.01
	Other	0.80	(0.74,0.85)	0.03
Marital status				
	Married	0.58	(0.55,0.61)	0.01
	Separated	0.55	(0.51,0.58)	0.02
	Divorced	0.62	(0.59,0.65)	0.01
	Widowed	0.57	(0.54,0.60)	0.01
	Domestic partner	0.41	(0.38,0.44)	0.02
	Single, never married	0.54	(0.51,0.57)	0.01
Employment status				
	Employed for an employer	0.56	(0.53,0.59)	0.01
	Self-employed	0.58	(0.55,0.60)	0.01
	Retired	0.57	(0.54,0.60)	0.01
	Student	0.54	(0.51,0.57)	0.01
	Homemaker	0.59	(0.56,0.61)	0.01
	Unemployed and looking for a job	0.56	(0.53,0.59)	0.01
	None of these/other	0.57	(0.54,0.60)	0.01
Education				

Variable	Category	Proportion	95% CI of Proportion	SE Analogue (CI Width/4)
	Up to 8 years	0.57	(0.54,0.60)	0.01
	9-15 years	0.55	(0.53,0.58)	0.01
	16+ years	0.55	(0.52,0.58)	0.01
Religious service attendance				
	>1/week	0.71	(0.69,0.74)	0.01
	1/week	0.67	(0.65,0.70)	0.01
	1-3/month	0.62	(0.59,0.65)	0.01
	A few times a year	0.55	(0.52,0.58)	0.01
	Never	0.44	(0.41,0.47)	0.01
Immigration status				
	Born in this country	0.57	(0.54,0.60)	0.01
	Born in another country	0.57	(0.54,0.59)	0.01
Overall		0.55	(0.54,0.57)	

Table S24. Ordered means of belief in life after death with standard deviations, dichotomizing as Yes/Unsure vs. No.

Country	Mean	95% CI	Standard Deviation
Indonesia	0.98	(0.97, 0.98)	0.14
Philippines	0.92	(0.91, 0.93)	0.28
Kenya	0.87	(0.86, 0.88)	0.34
Mexico	0.85	(0.84, 0.86)	0.36
Turkey	0.85	(0.83, 0.87)	0.36
Argentina	0.84	(0.83, 0.86)	0.36
United States	0.83	(0.82, 0.83)	0.38
Nigeria	0.79	(0.77, 0.80)	0.41
Poland	0.79	(0.76, 0.81)	0.41
Brazil	0.78	(0.77, 0.79)	0.41
Egypt	0.78	(0.76, 0.79)	0.42
Israel	0.78	(0.75, 0.81)	0.41
Tanzania	0.78	(0.77, 0.80)	0.41
Hong Kong	0.75	(0.73, 0.77)	0.44
Spain	0.72	(0.71, 0.74)	0.45
United Kingdom	0.72	(0.70, 0.73)	0.45
South Africa	0.71	(0.69, 0.73)	0.45
Australia	0.68	(0.66, 0.70)	0.47
Japan	0.67	(0.66, 0.68)	0.47
Germany	0.65	(0.63, 0.66)	0.48
Sweden	0.63	(0.62, 0.64)	0.48
India	0.57	(0.56, 0.59)	0.49

Table S25. Ordered proportions of people who said "unsure" to the belief in life after death with standard deviations

Country	Mean	95% CI	Standard Deviation
Japan	0.47	(0.46, 0.47)	0.50
Philippines	0.38	(0.36, 0.39)	0.48
Sweden	0.34	(0.33, 0.35)	0.47
United Kingdom	0.33	(0.31, 0.35)	0.47
Australia	0.32	(0.30, 0.34)	0.47
Spain	0.32	(0.31, 0.34)	0.47
Germany	0.30	(0.29, 0.31)	0.46
Hong Kong	0.29	(0.27, 0.32)	0.46
United States	0.27	(0.26, 0.28)	0.44
Argentina	0.26	(0.25, 0.28)	0.44
Mexico	0.25	(0.23, 0.26)	0.43
Israel	0.23	(0.21, 0.26)	0.42
Poland	0.21	(0.19, 0.23)	0.41
South Africa	0.18	(0.16, 0.20)	0.39
Tanzania	0.18	(0.16, 0.19)	0.38
Turkey	0.18	(0.15, 0.20)	0.38
Brazil	0.17	(0.17, 0.18)	0.38
Kenya	0.15	(0.14, 0.16)	0.36
Egypt	0.13	(0.12, 0.15)	0.34
India	0.10	(0.09, 0.10)	0.29
Nigeria	0.06	(0.05, 0.07)	0.24
Indonesia	0.03	(0.02, 0.04)	0.17

1. Forest Plots

Figure S1. Forest plot for `Age group`-`18-24`

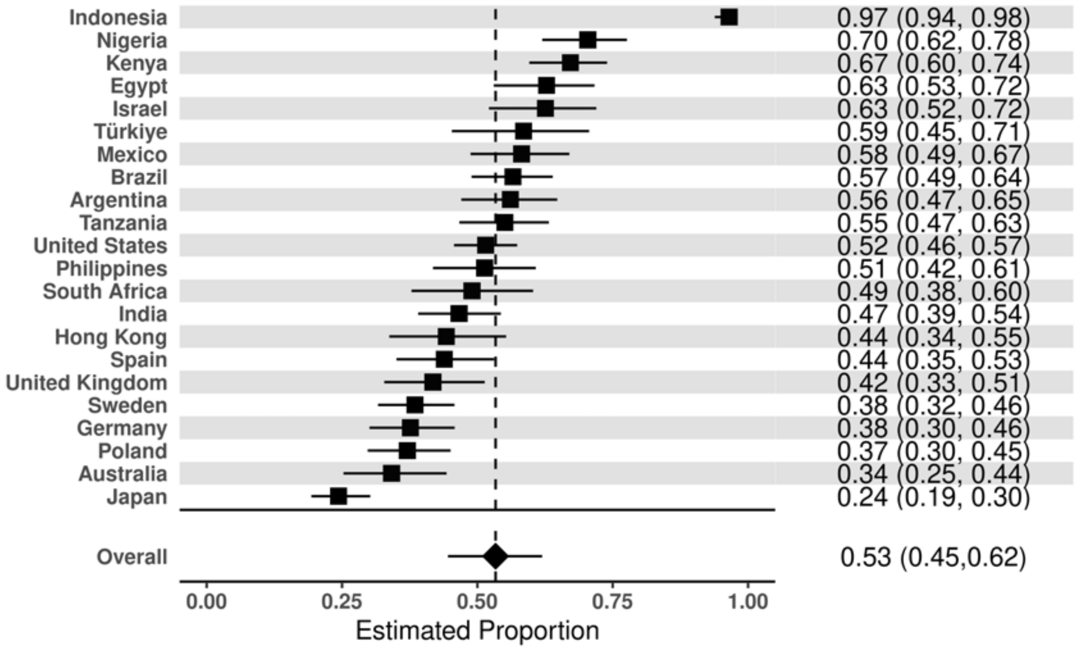

Probability-scale: τ=0.206;
Logit-scale: τ=0.829; Q-profile 95% CI [0.589, 1.127]; I^2=96.66;
Plot is based on back transformed bounds after using approximate logit SE that aren't guarenteed to match the robust SE of a proportion.

Figure S2. Forest plot for `Age group`-`25-29`

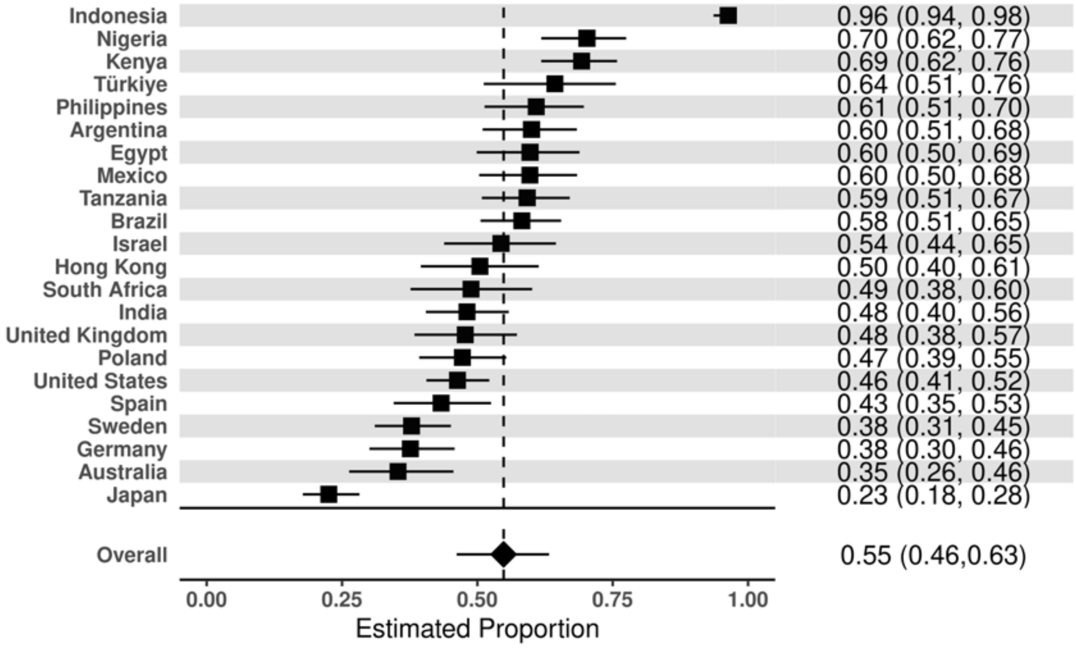

Probability-scale: τ=0.202;
Logit-scale: τ=0.816; Q-profile 95% CI [0.581, 1.110]; I^2=96.55;
Plot is based on back transformed bounds after using approximate logit SE that aren't guarenteed to match the robust SE of a proportion.

Figure S3. Forest plot for `Age group`-`30-39`

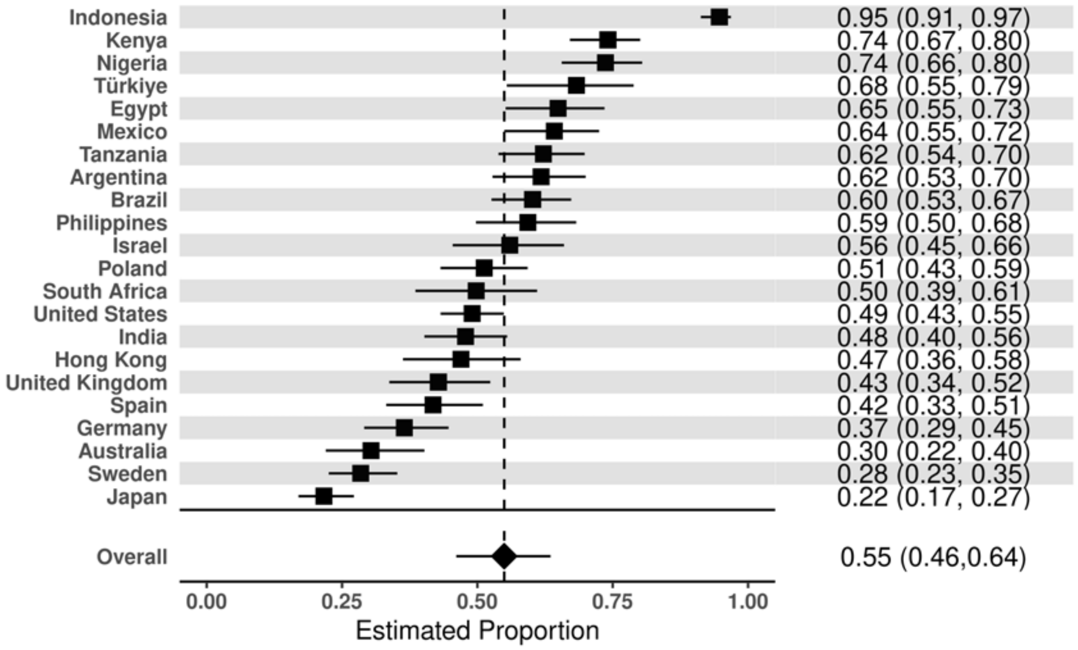

Probability-scale: τ=0.207;
Logit-scale: τ=0.835; Q-profile 95% CI [0.605, 1.140]; I^2=96.66;
Plot is based on back transformed bounds after using approximate logit SE that aren't guarenteed to match the robust SE of a proportion.

Figure S4. Forest plot for `Age group`-`40-49`

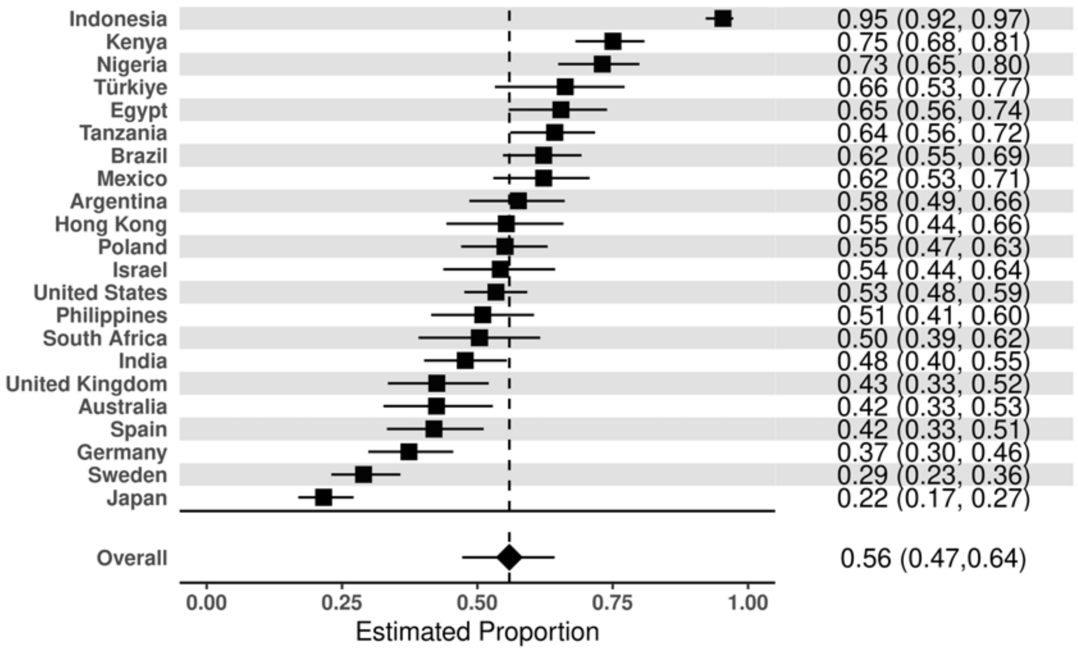

Probability-scale: τ=0.202;
Logit-scale: τ=0.821; Q-profile 95% CI [0.592, 1.120]; I^2=96.56;
Plot is based on back transformed bounds after using approximate logit SE that aren't guarenteed to match the robust SE of a proportion.

Figure S5. Forest plot for `Age group`-`50-59`

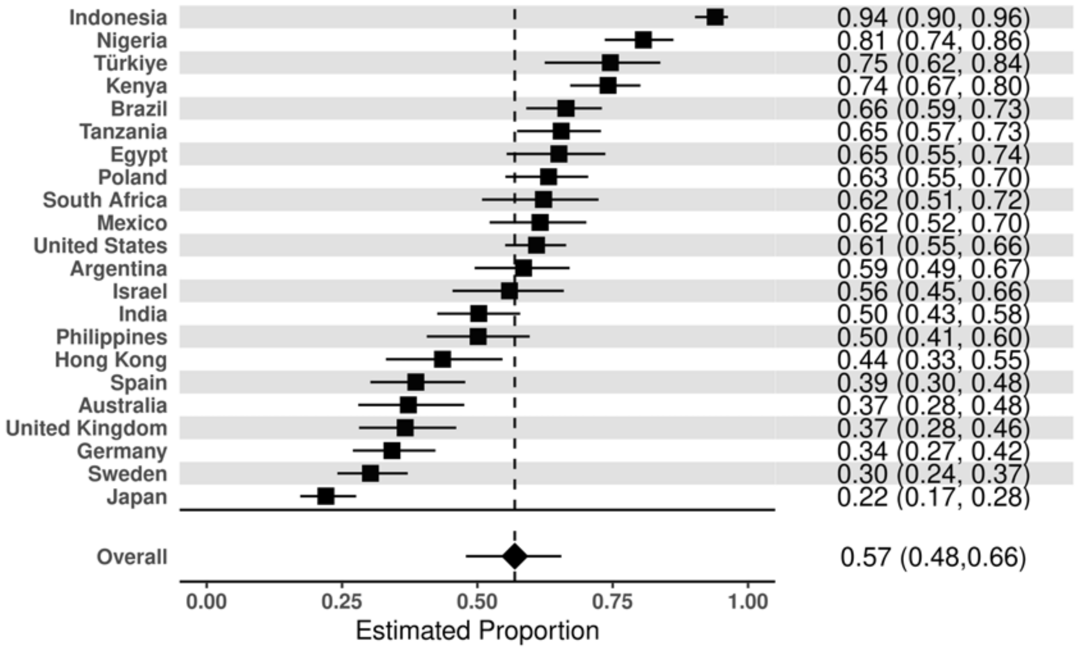

Probability-scale: τ=0.209;
Logit-scale: τ=0.853; Q-profile 95% CI [0.621, 1.165]; I^2=96.77;
Plot is based on back transformed bounds after using approximate logit SE that aren't guarenteed to match the robust SE of a proportion.

Figure S6. Forest plot for `Age group`-`60-69`

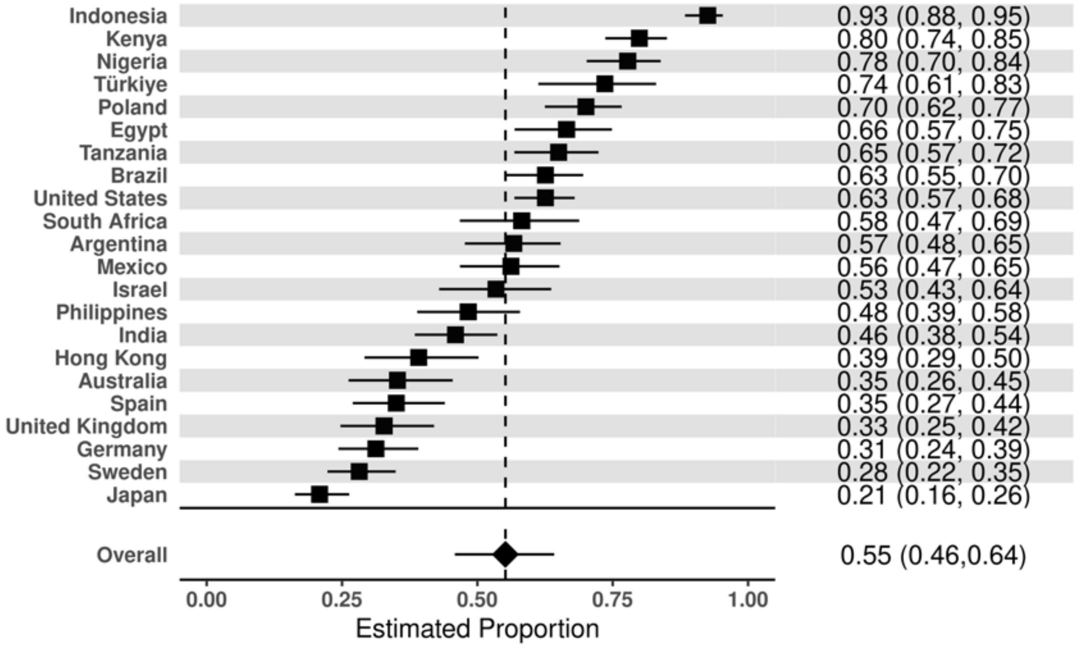

Probability-scale: τ=0.218;
Logit-scale: τ=0.883; Q-profile 95% CI [0.646, 1.206]; I^2=96.95;
Plot is based on back transformed bounds after using approximate logit SE that aren't guarenteed to match the robust SE of a proportion.

Figure S7. Forest plot for `Age group`-`70-79`

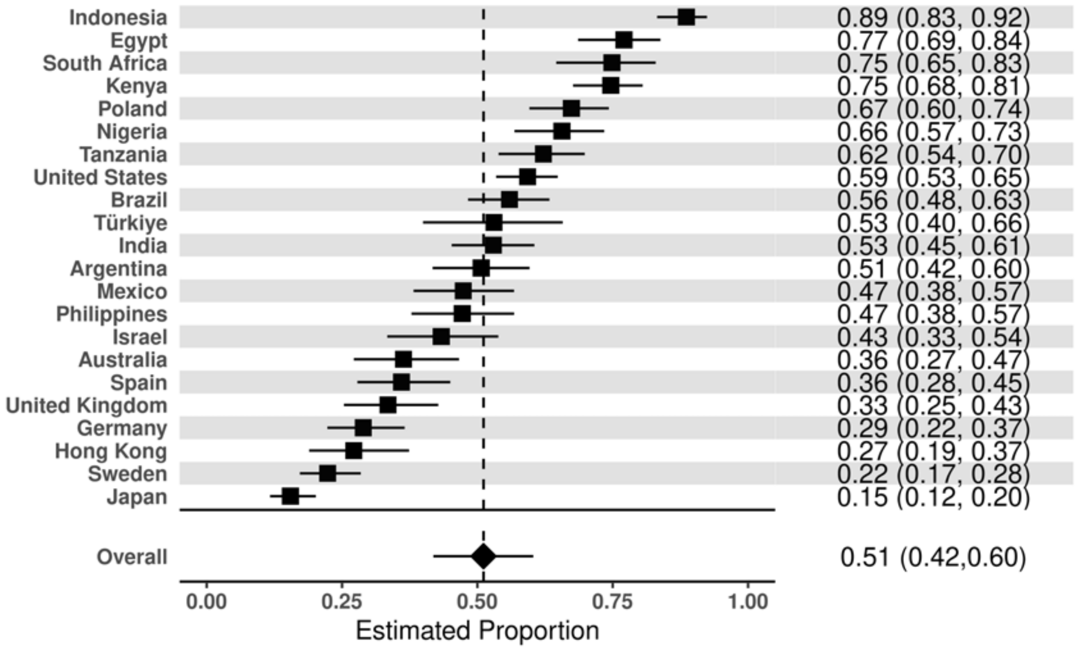

Probability-scale: τ=0.220;
Logit-scale: τ=0.879; Q-profile 95% CI [0.645, 1.202]; I^2=96.93;
Plot is based on back transformed bounds after using approximate logit SE that aren't guarenteed to match the robust SE of a proportion.

Figure S8. Forest plot for `Age group`-`80 or older`

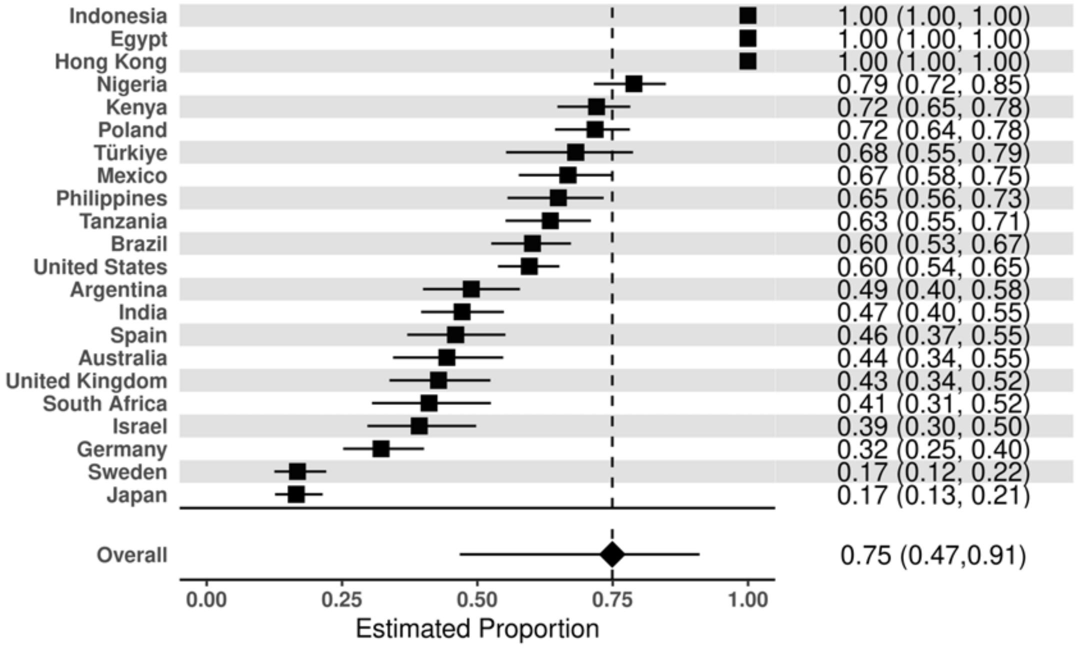

Probability-scale: τ=0.546;
Logit-scale: τ=2.906; Q-profile 95% CI [1.966, 3.888]; I^2=99.68;
Plot is based on back transformed bounds after using approximate logit SE that aren't guarenteed to match the robust SE of a proportion.

Figure S9. Forest plot for `Gender`-`Male`

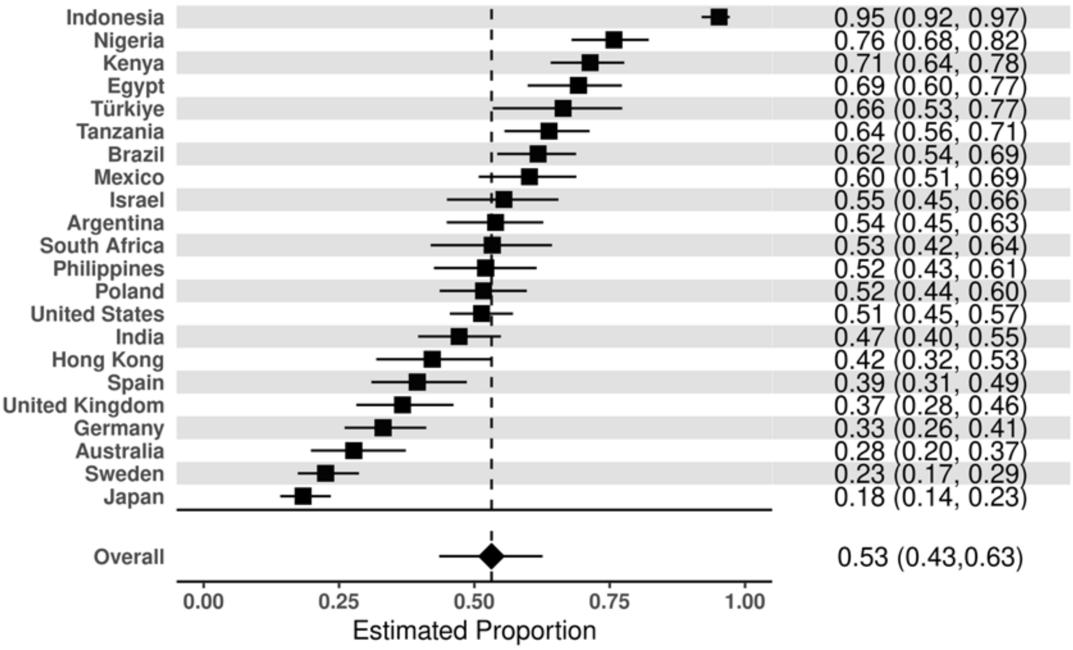

Probability-scale: τ=0.228;
Logit-scale: τ=0.915; Q-profile 95% CI [0.665, 1.247]; I^2=97.17;
Plot is based on back transformed bounds after using approximate logit SE that aren't guarenteed to match the robust SE of a proportion.

Figure S10. Forest plot for `Gender`-`Female`

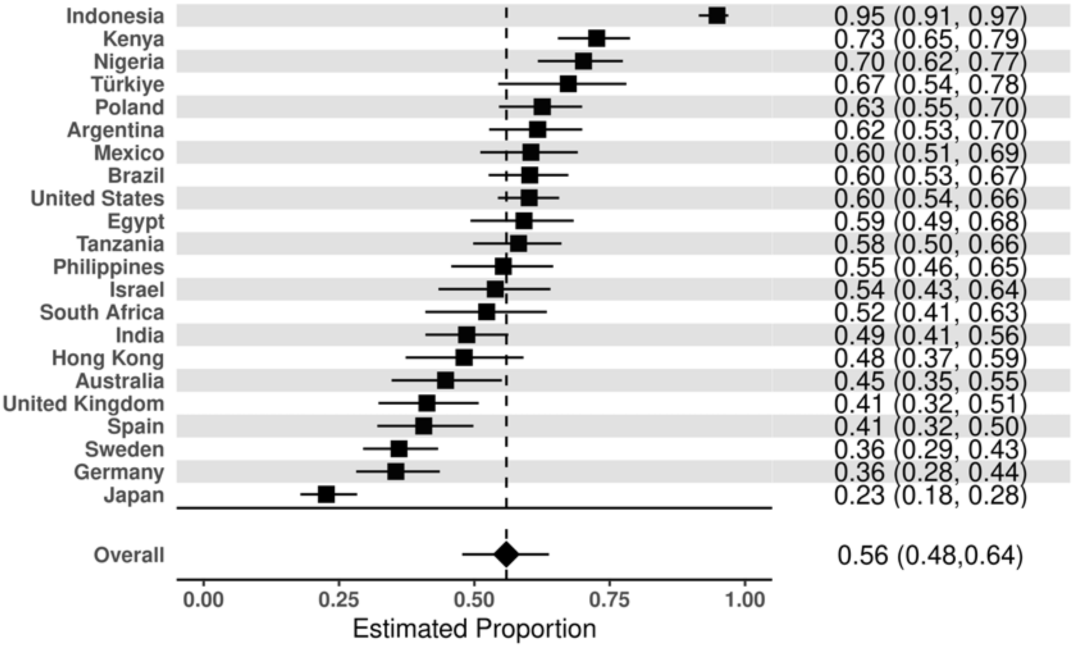

Probability-scale: τ=0.189;
Logit-scale: τ=0.768; Q-profile 95% CI [0.551, 1.048]; I^2=96.11;
Plot is based on back transformed bounds after using approximate logit SE that aren't guarenteed to match the robust SE of a proportion.

Figure S11. Forest plot for `Gender`-`Other`

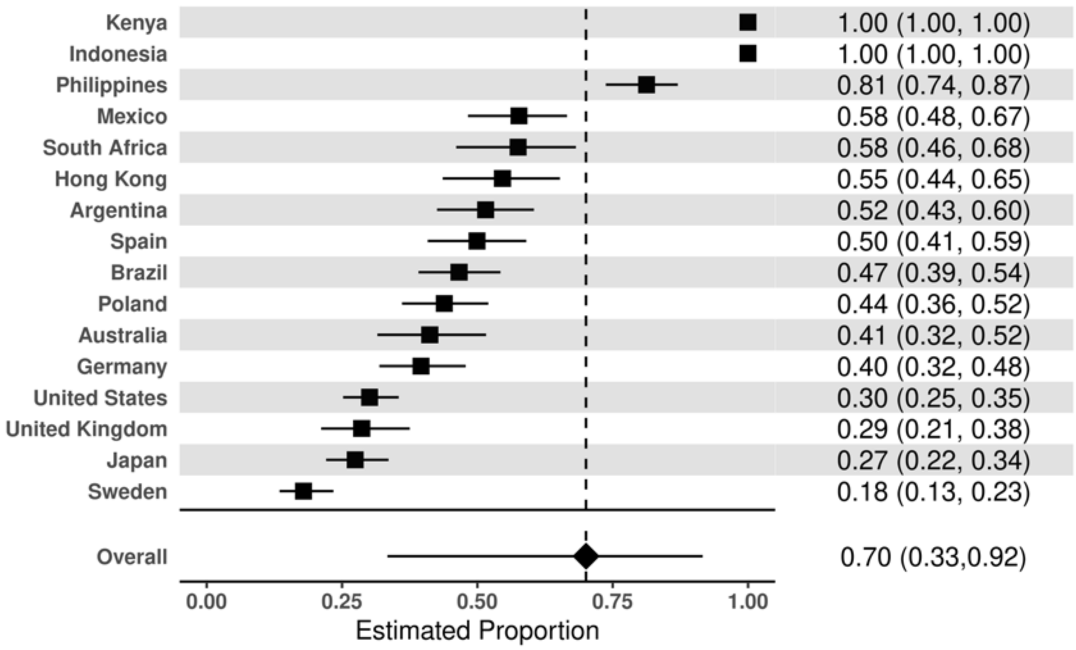

Probability-scale: τ=0.655;
Logit-scale: τ=3.124; Q-profile 95% CI [2.006, 4.432]; I^2=99.73;
Plot is based on back transformed bounds after using approximate logit SE that aren't guarenteed to match the robust SE of a proportion.
Excluded countries: India, Egypt, Israel, Nigeria, Tanzania, Turkiye

Figure S12. Forest plot for `Marital status`-`Married`

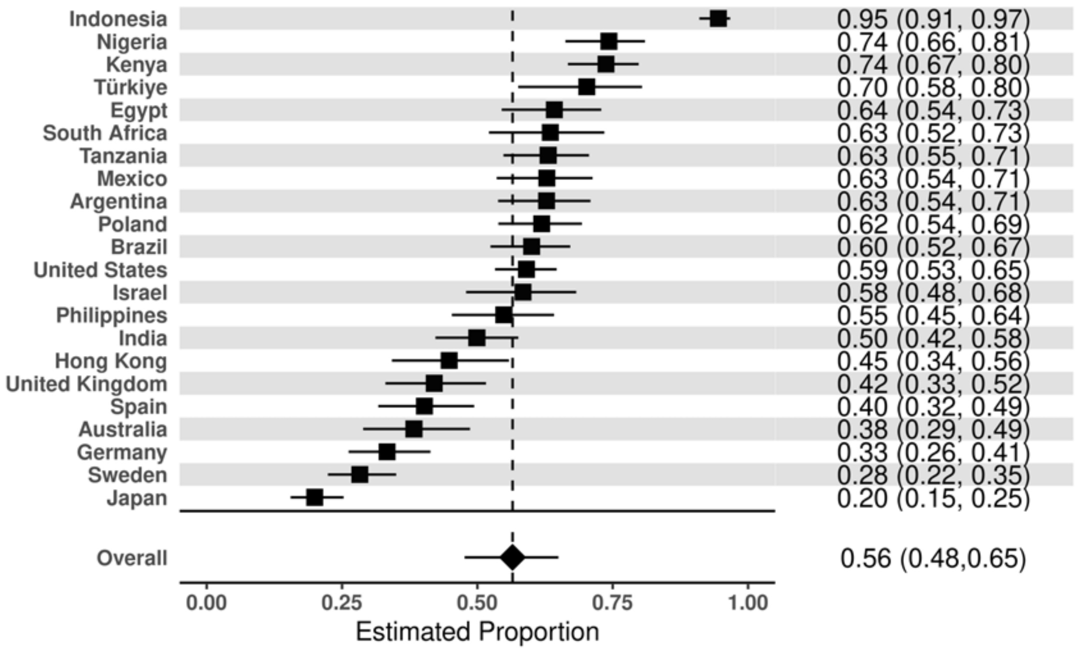

Probability-scale: τ=0.206;
Logit-scale: τ=0.836; Q-profile 95% CI [0.607, 1.142]; I^2=96.65;
Plot is based on back transformed bounds after using approximate logit SE that aren't guarenteed to match the robust SE of a proportion.

Figure S13. Forest plot for `Marital status`-`Separated`

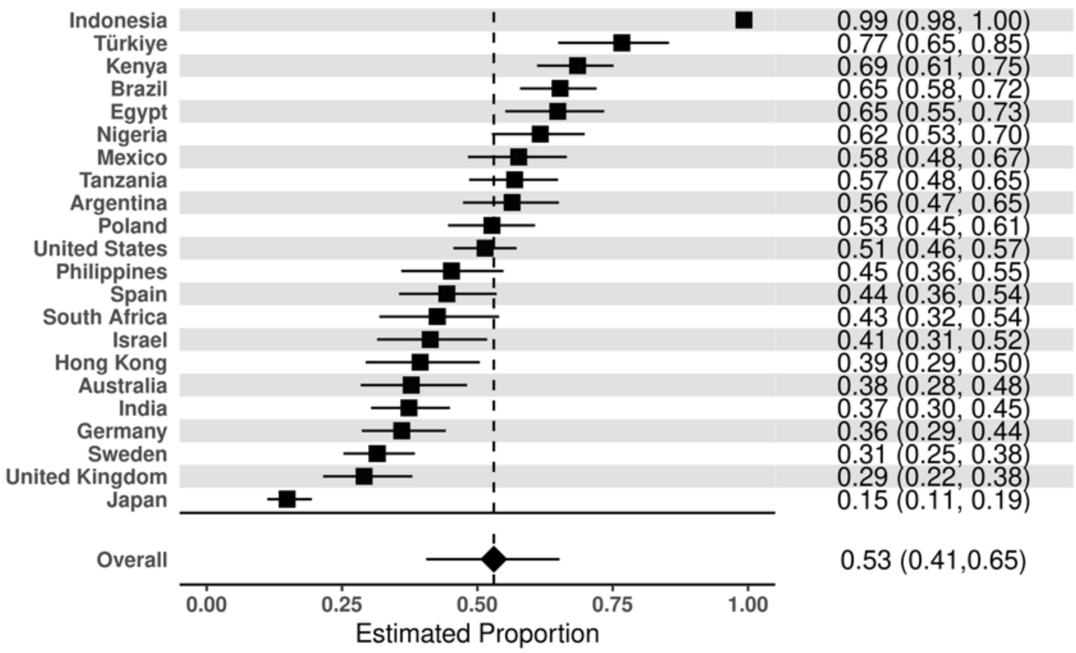

Probability-scale: τ=0.298;
Logit-scale: τ=1.195; Q-profile 95% CI [0.840, 1.612]; I^2=98.31;
Plot is based on back transformed bounds after using approximate logit SE that aren't guarenteed to match the robust SE of a proportion.

Figure S14. Forest plot for `Marital status`-`Divorced`

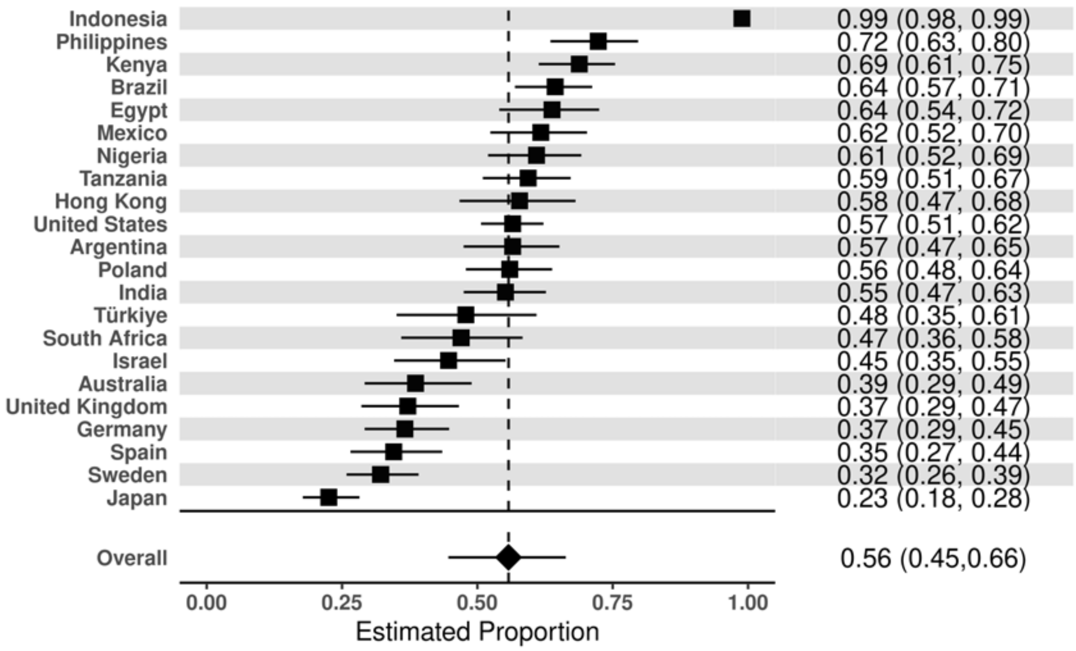

Probability-scale: τ=0.260;
Logit-scale: τ=1.055; Q-profile 95% CI [0.739, 1.424]; I^2=97.87;
Plot is based on back transformed bounds after using approximate logit SE that aren't guarenteed to match the robust SE of a proportion.

Figure S15. Forest plot for `Marital status`-`Widowed`

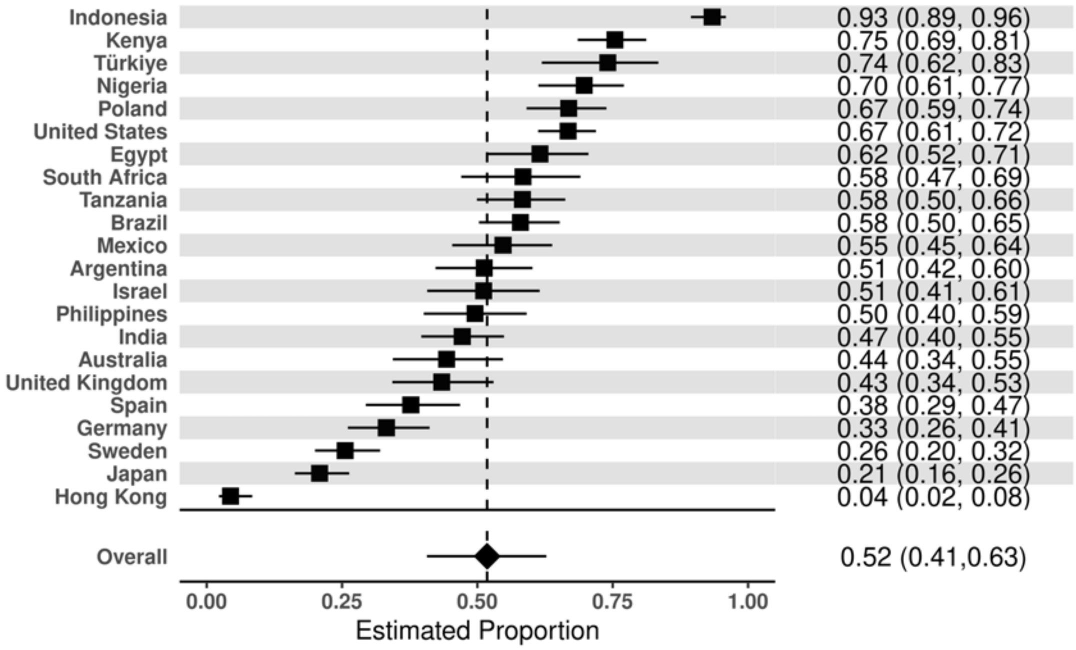

Probability-scale: τ=0.265;
Logit-scale: τ=1.061; Q-profile 95% CI [0.765, 1.440]; I^2=97.86;
Plot is based on back transformed bounds after using approximate logit SE that
aren't guarenteed to match the robust SE of a proportion.

Figure S16. Forest plot for `Marital status`-`Single, never married`

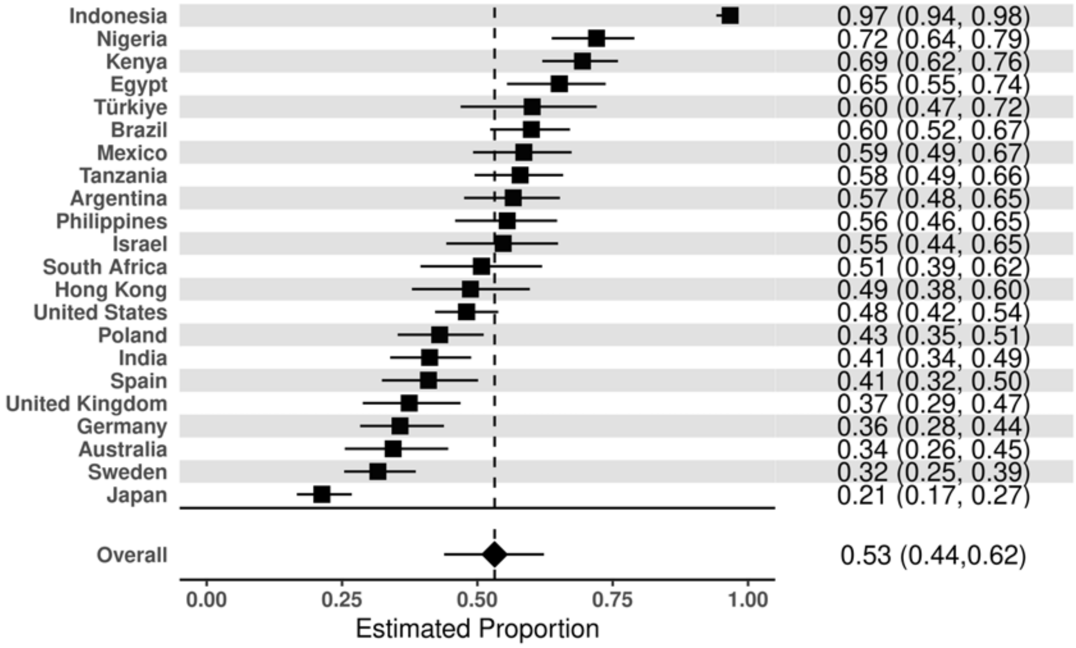

Probability-scale: τ=0.219;
Logit-scale: τ=0.881; Q-profile 95% CI [0.631, 1.198]; I^2=97.00;
Plot is based on back transformed bounds after using approximate logit SE that
aren't guarenteed to match the robust SE of a proportion.

Figure S17. Forest plot for `Marital status`-`Domestic partner`

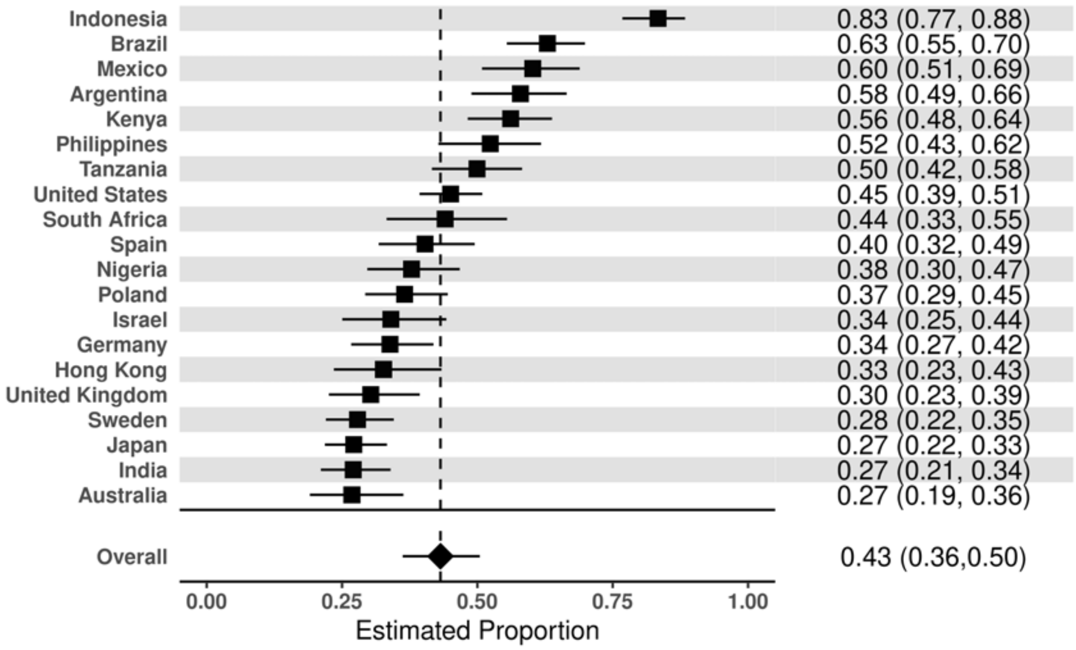

Probability-scale: τ=0.159;
Logit-scale: τ=0.648; Q-profile 95% CI [0.462, 0.905]; I^2=94.85;
Plot is based on back transformed bounds after using approximate logit SE that aren't guarenteed to match the robust SE of a proportion.
Excluded countries: Egypt, Turkiye

Figure S18. Forest plot for `Employment status`-`Employed for an employer`

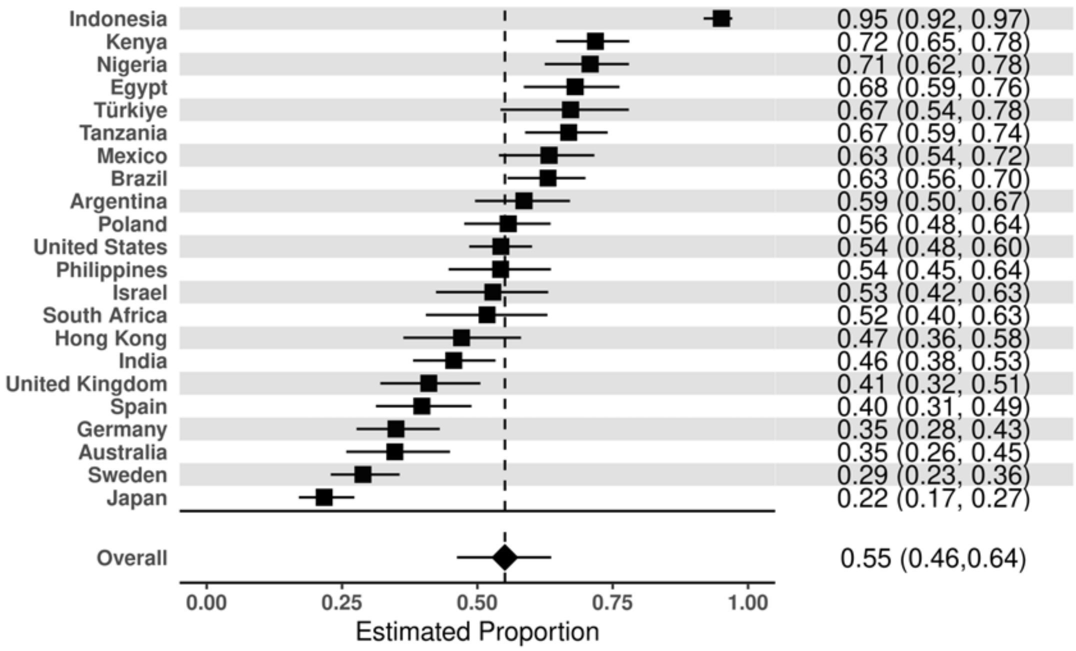

Probability-scale: τ=0.207;
Logit-scale: τ=0.835; Q-profile 95% CI [0.604, 1.139]; I^2=96.66;
Plot is based on back transformed bounds after using approximate logit SE that aren't guarenteed to match the robust SE of a proportion.

Figure S19. Forest plot for `Employment status`-`Self-employed`

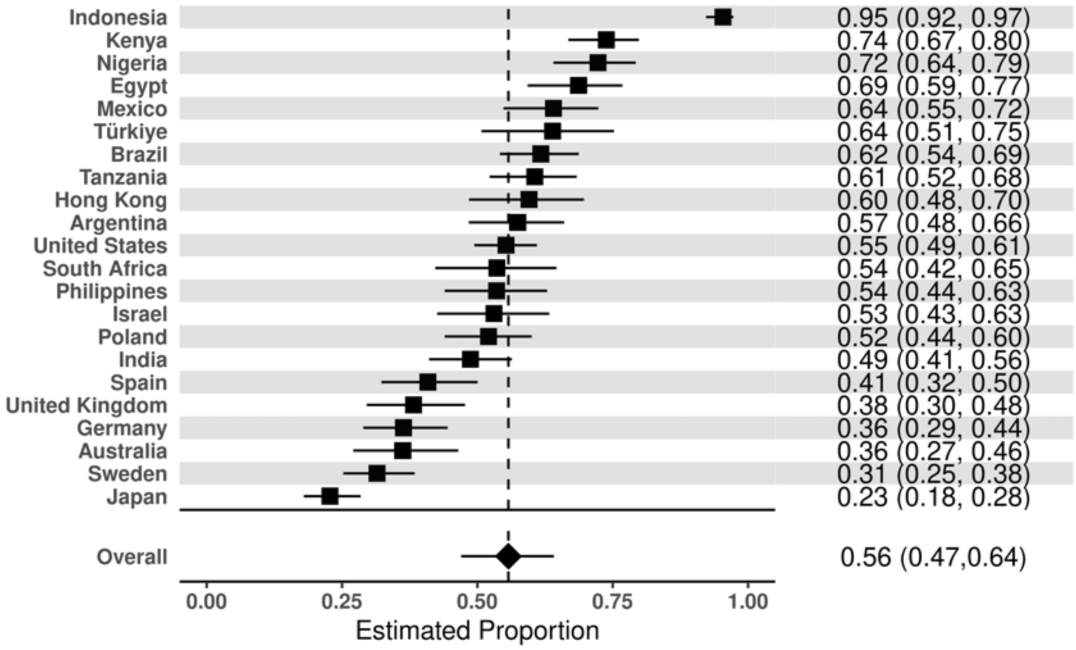

Probability-scale: τ=0.203;
Logit-scale: τ=0.823; Q-profile 95% CI [0.593, 1.123]; I^2=96.58;
Plot is based on back transformed bounds after using approximate logit SE that aren't guarenteed to match the robust SE of a proportion.

Figure S20. Forest plot for `Employment status`-`Retired`

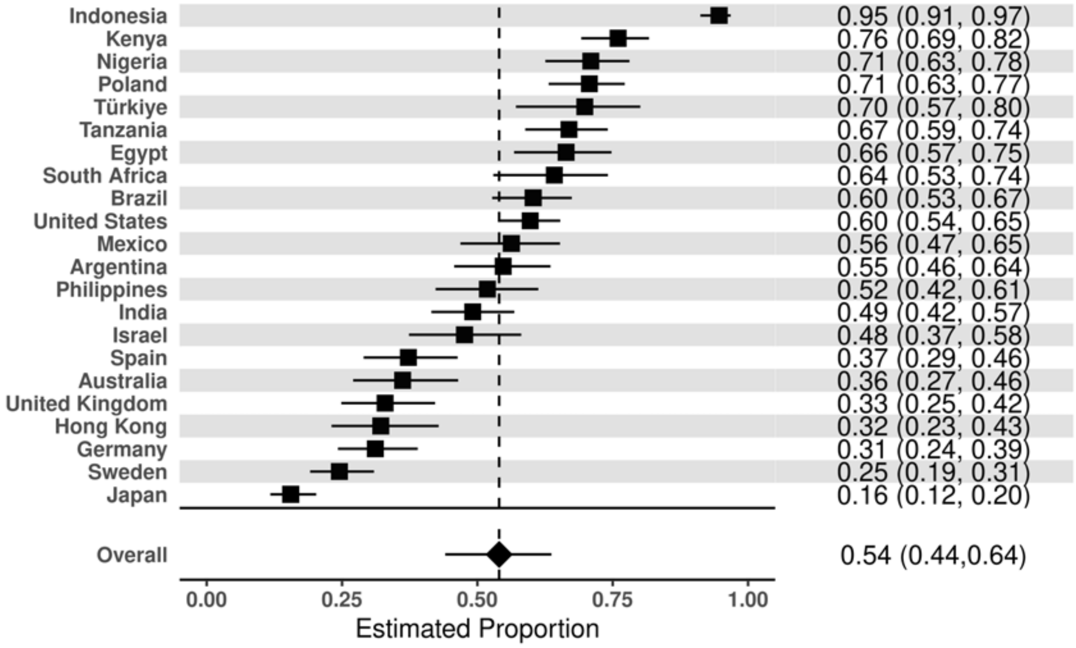

Probability-scale: τ=0.234;
Logit-scale: τ=0.943; Q-profile 95% CI [0.689, 1.286]; I^2=97.30;
Plot is based on back transformed bounds after using approximate logit SE that aren't guarenteed to match the robust SE of a proportion.

Figure S21. Forest plot for `Employment status`-`Student`

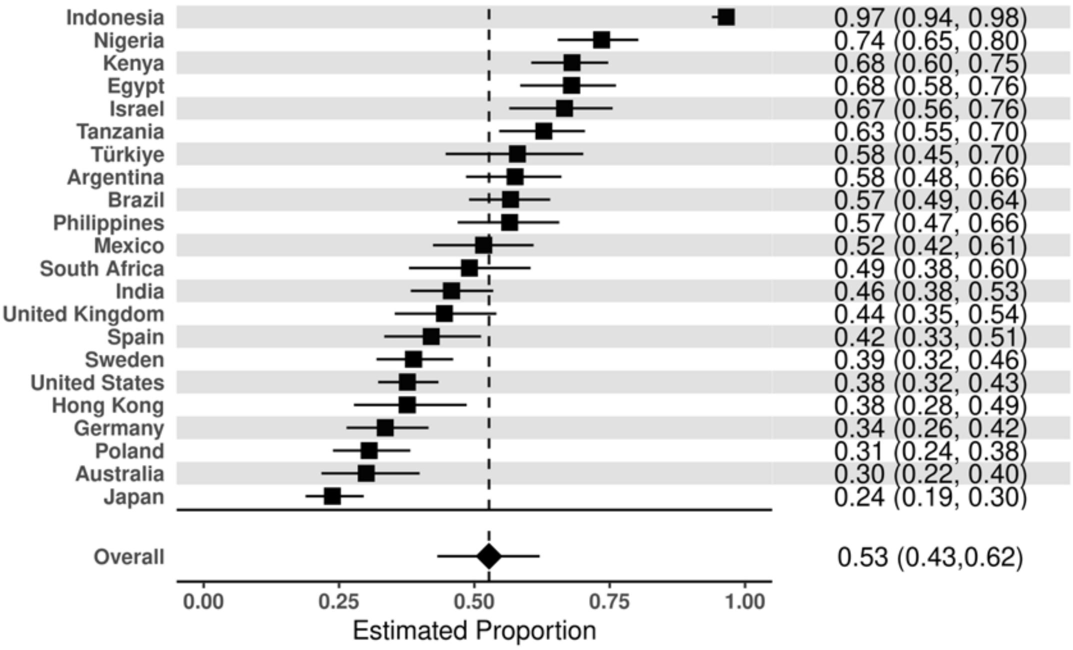

Probability-scale: τ=0.225;
Logit-scale: τ=0.903; Q-profile 95% CI [0.649, 1.229]; I^2=97.13;
Plot is based on back transformed bounds after using approximate logit SE that aren't guarenteed to match the robust SE of a proportion.

Figure S22. Forest plot for `Employment status`-`Homemaker`

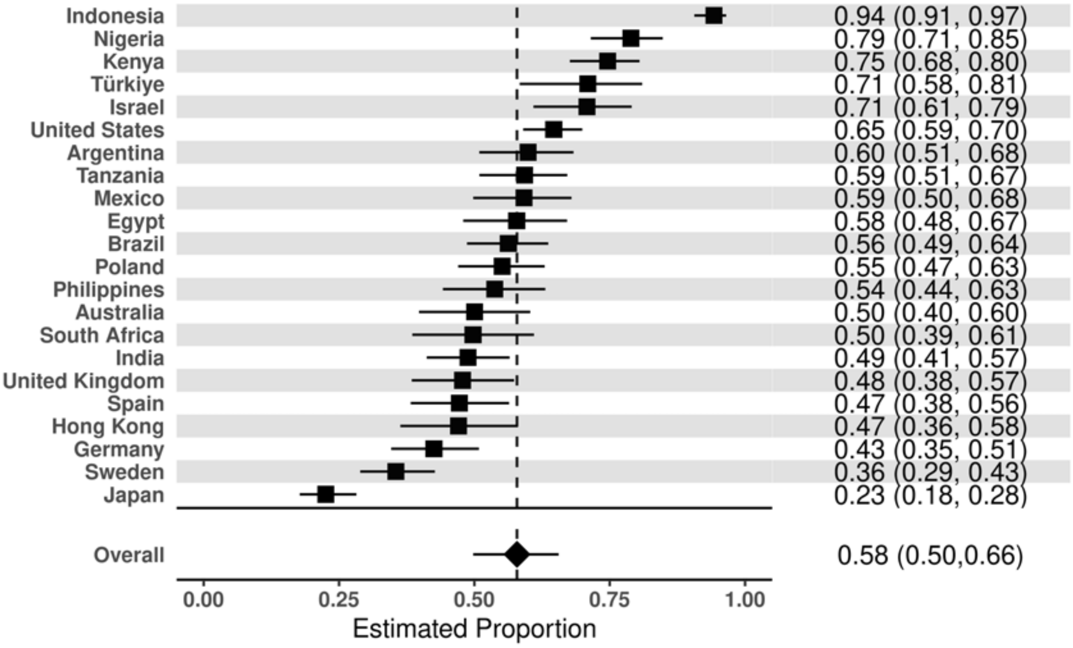

Probability-scale: τ=0.186;
Logit-scale: τ=0.764; Q-profile 95% CI [0.551, 1.044]; I^2=96.05;
Plot is based on back transformed bounds after using approximate logit SE that aren't guarenteed to match the robust SE of a proportion.

Figure S23. Forest plot for `Employment status`-`Unemployed and looking for a job`

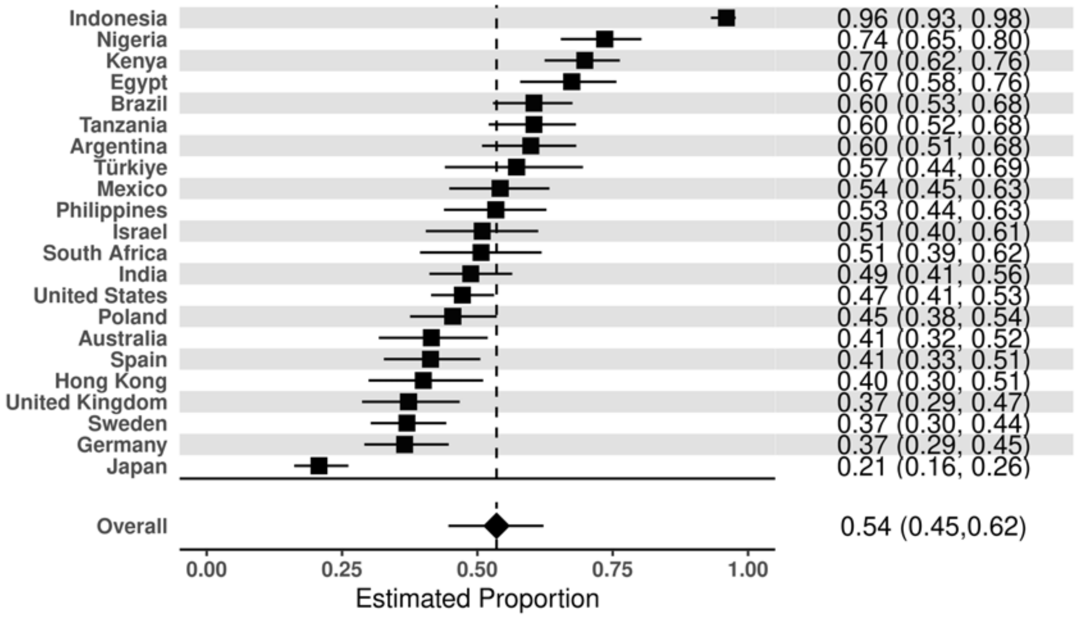

Probability-scale: τ=0.208;
Logit-scale: τ=0.837; Q-profile 95% CI [0.600, 1.140]; I^2=96.70;
Plot is based on back transformed bounds after using approximate logit SE that aren't guarenteed to match the robust SE of a proportion.

Figure S24. Forest plot for `Employment status`-`None of these/other`

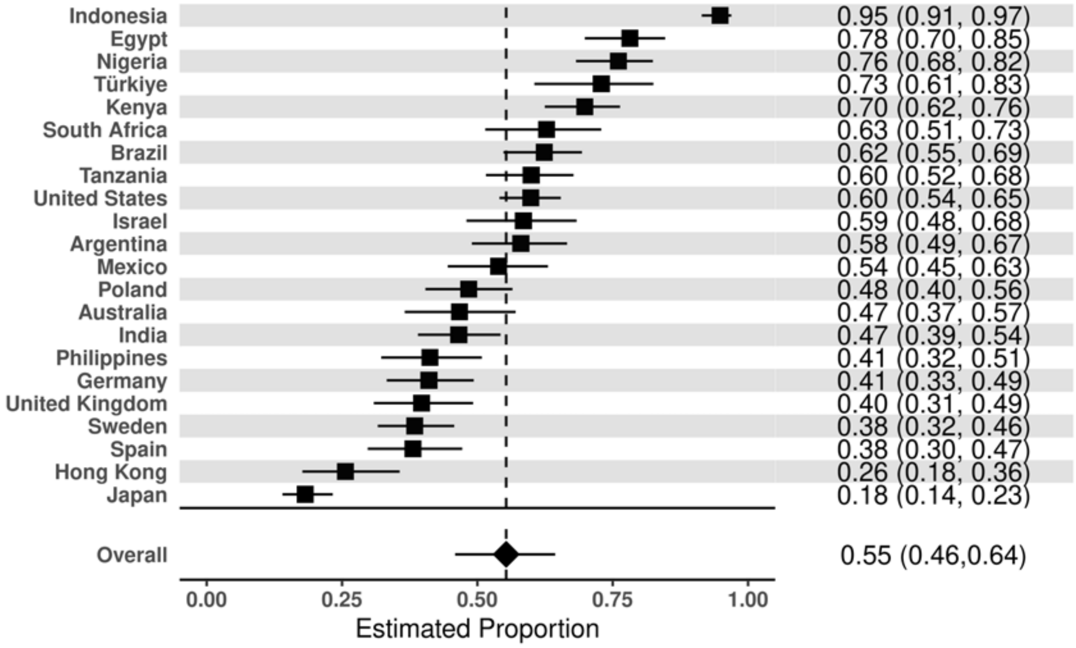

Probability-scale: τ=0.220;
Logit-scale: τ=0.890; Q-profile 95% CI [0.646, 1.214]; I^2=97.03;
Plot is based on back transformed bounds after using approximate logit SE that aren't guarenteed to match the robust SE of a proportion.

Figure S25. Forest plot for `Religious service attendance`-`>1/week`

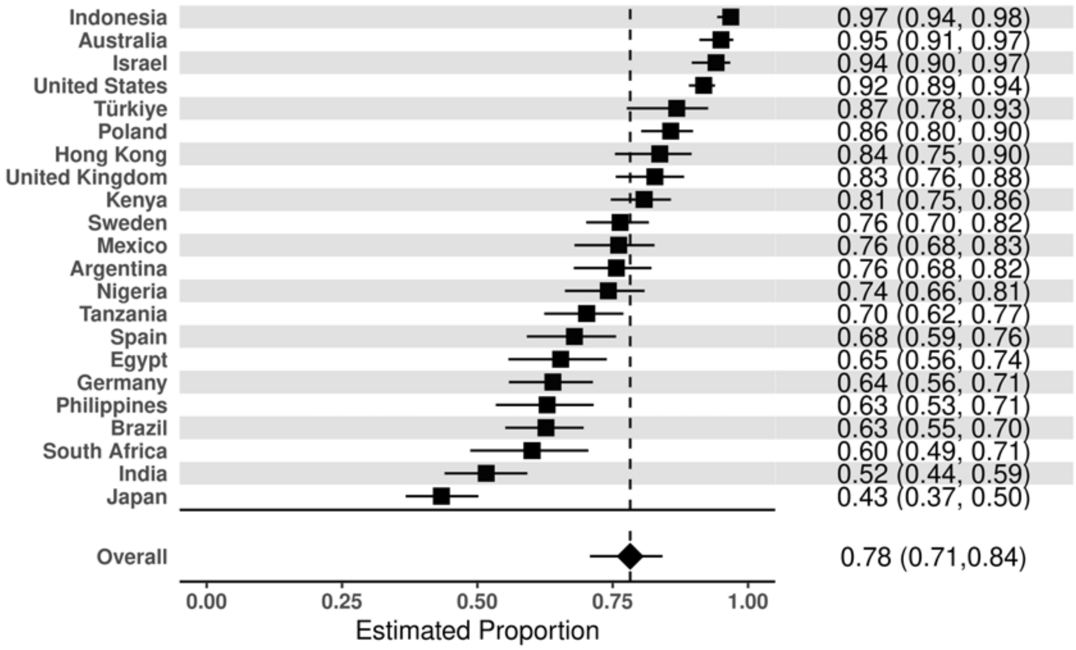

Probability-scale: τ=0.157;
Logit-scale: τ=0.924; Q-profile 95% CI [0.670, 1.262]; I^2=96.83;
Plot is based on back transformed bounds after using approximate logit SE that aren't guarenteed to match the robust SE of a proportion.

Figure S26. Forest plot for `Religious service attendance`-`1/week`

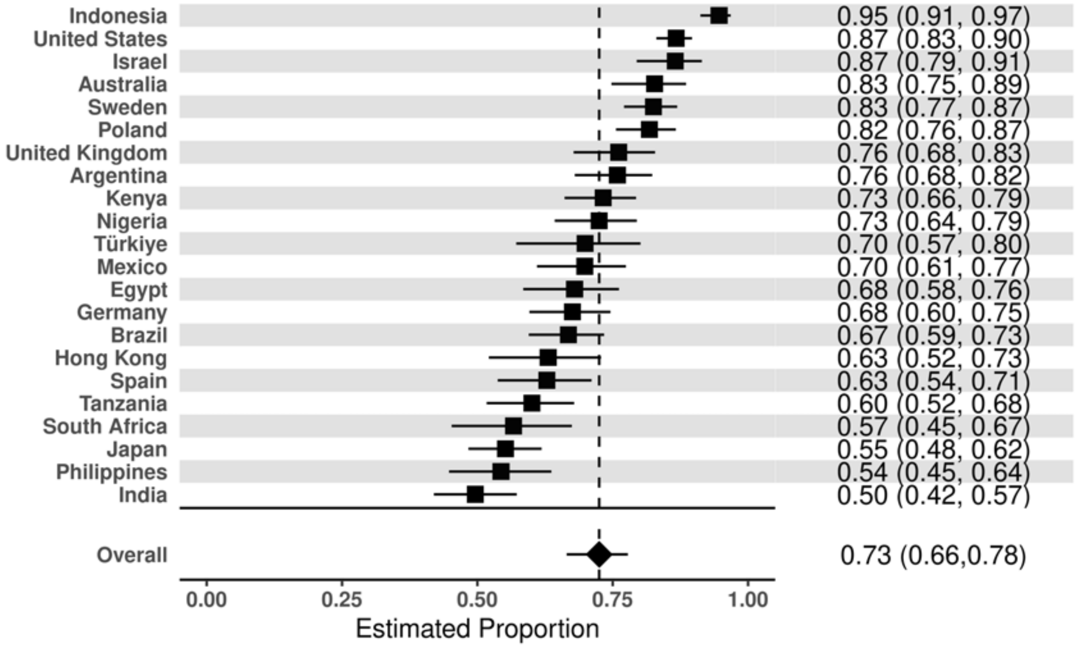

Probability-scale: τ=0.131;
Logit-scale: τ=0.658; Q-profile 95% CI [0.471, 0.904]; I^2=94.34;
Plot is based on back transformed bounds after using approximate logit SE that aren't guarenteed to match the robust SE of a proportion.

Figure S27. Forest plot for `Religious service attendance`-`1-3/month`

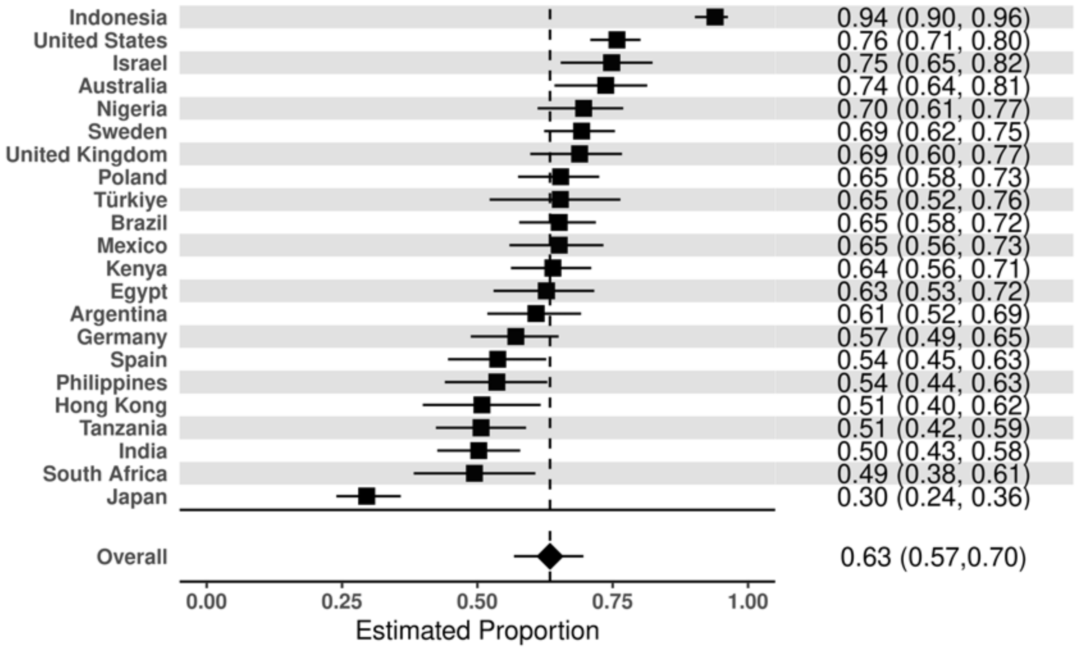

Probability-scale: τ=0.149;
Logit-scale: τ=0.644; Q-profile 95% CI [0.459, 0.883]; I^2=94.48;
Plot is based on back transformed bounds after using approximate logit SE that aren't guarenteed to match the robust SE of a proportion.

Figure S28. Forest plot for `Religious service attendance`-`A few times a year`

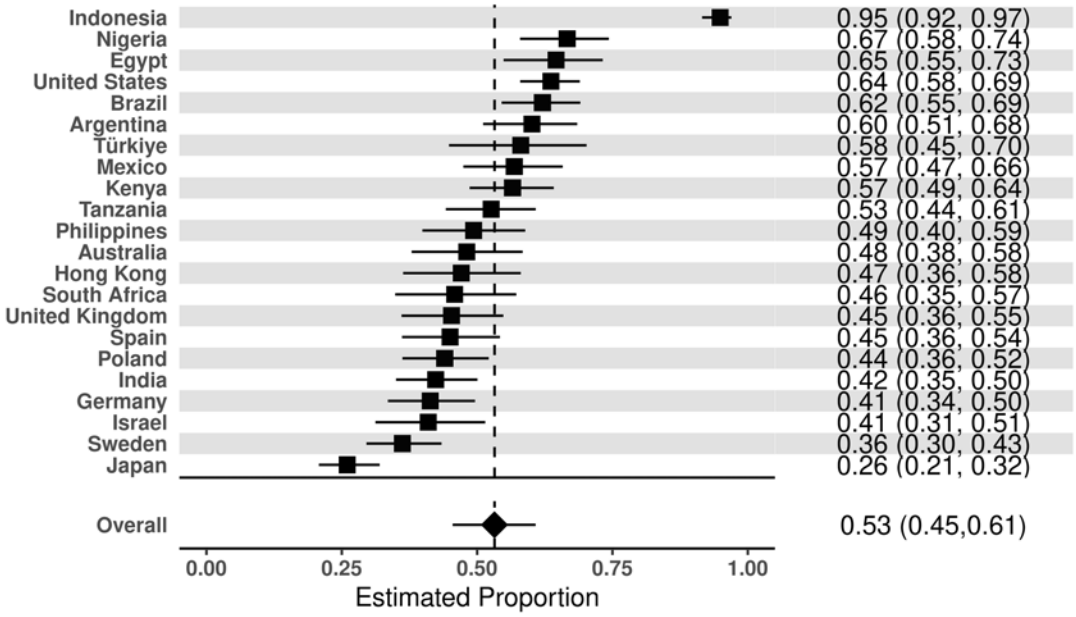

Probability-scale: τ=0.180;
Logit-scale: τ=0.725; Q-profile 95% CI [0.514, 0.988]; I^2=95.70;
Plot is based on back transformed bounds after using approximate logit SE that aren't guarenteed to match the robust SE of a proportion.

Figure S29. Forest plot for `Religious service attendance`-`Never`

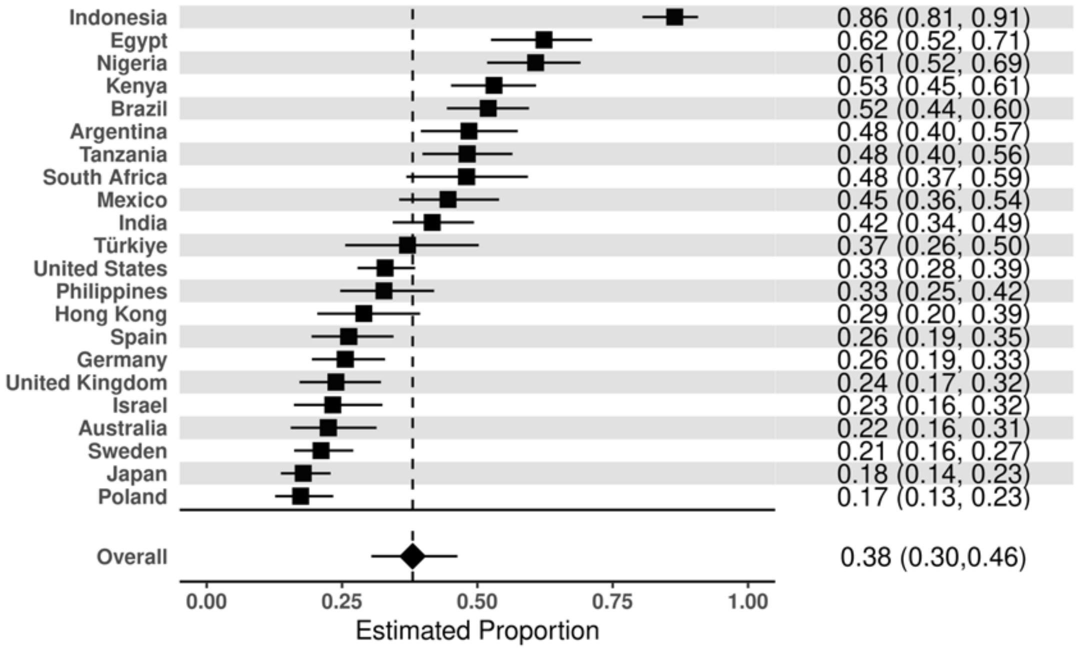

Probability-scale: τ=0.188;
Logit-scale: τ=0.797; Q-profile 95% CI [0.582, 1.091]; I^2=96.24;
Plot is based on back transformed bounds after using approximate logit SE that aren't guarenteed to match the robust SE of a proportion.

Figure S30. Forest plot for `Education`-`Up to 8 years`

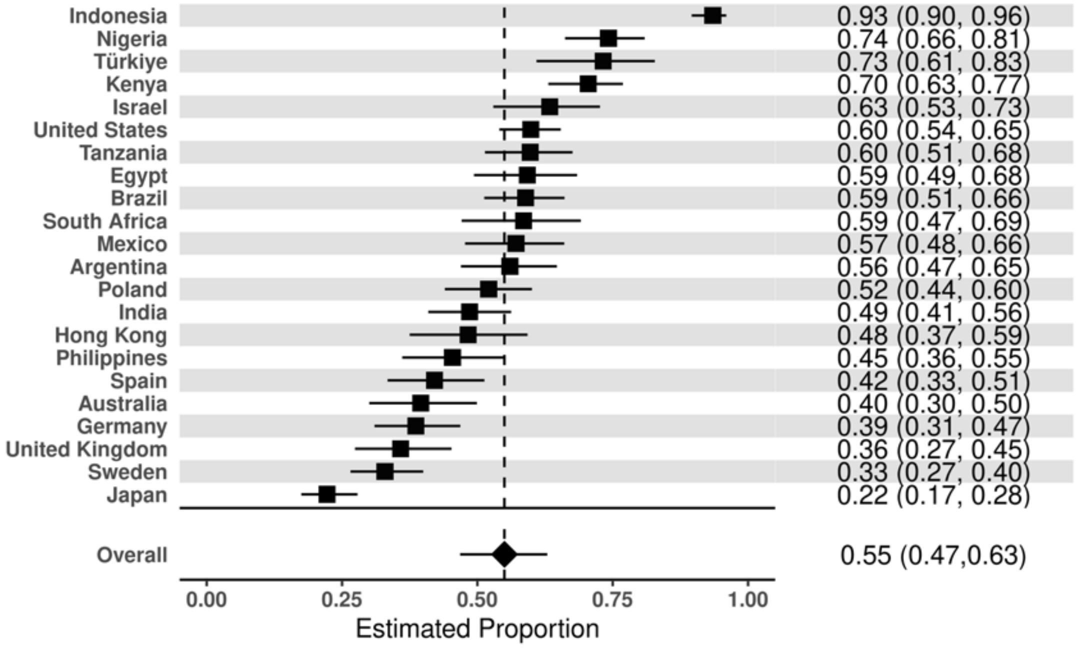

Probability-scale: τ=0.190;
Logit-scale: τ=0.768; Q-profile 95% CI [0.554, 1.050]; I^2=96.11;
Plot is based on back transformed bounds after using approximate logit SE that aren't guarenteed to match the robust SE of a proportion.

Figure S31. Forest plot for `Education`-`9-15 years`

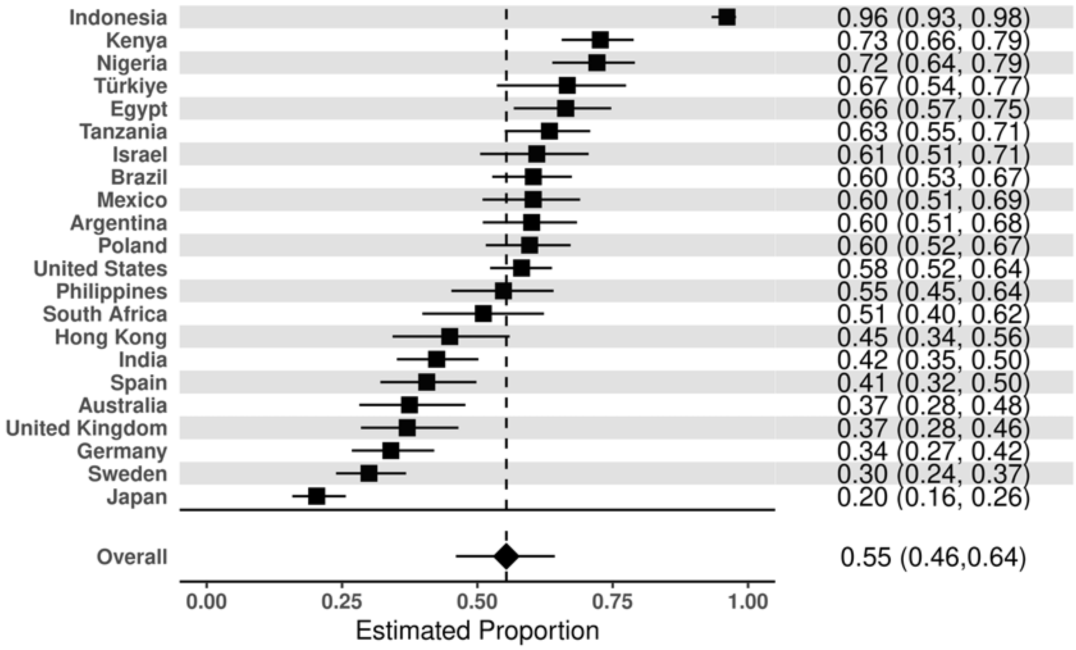

Probability-scale: τ=0.217;
Logit-scale: τ=0.879; Q-profile 95% CI [0.635, 1.198]; I^2=96.97;
Plot is based on back transformed bounds after using approximate logit SE that aren't guarenteed to match the robust SE of a proportion.

Figure S32. Forest plot for `Education`-`16+ years`

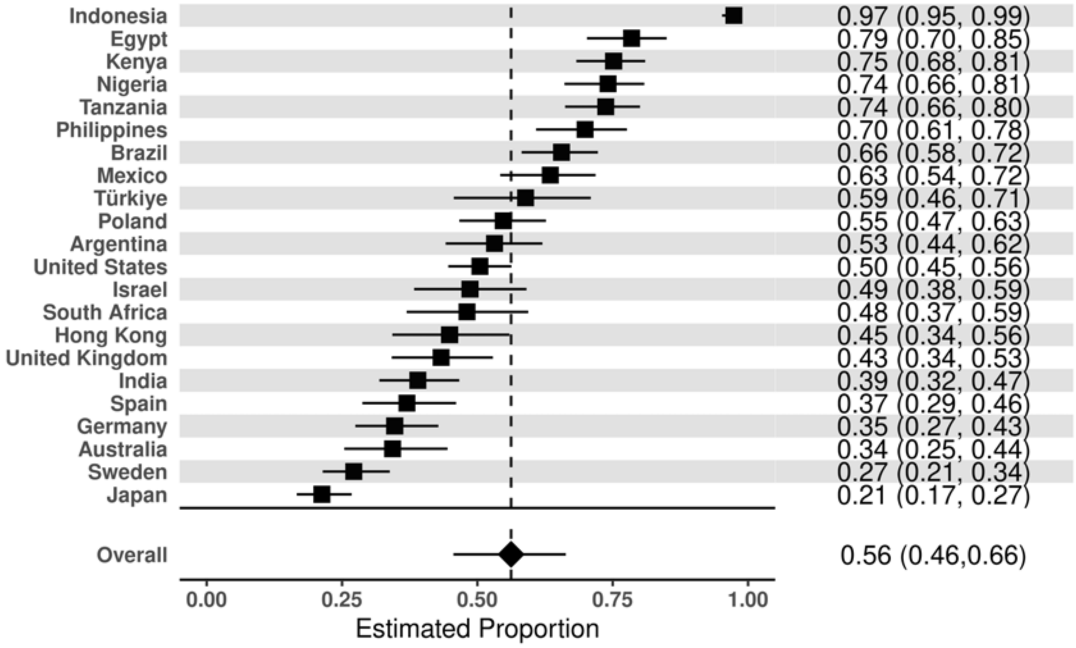

Probability-scale: τ=0.249;
Logit-scale: τ=1.010; Q-profile 95% CI [0.731, 1.373]; I^2=97.64;
Plot is based on back transformed bounds after using approximate logit SE that aren't guarenteed to match the robust SE of a proportion.

Figure S33. Forest plot for `Immigration status`-`Born in this country`

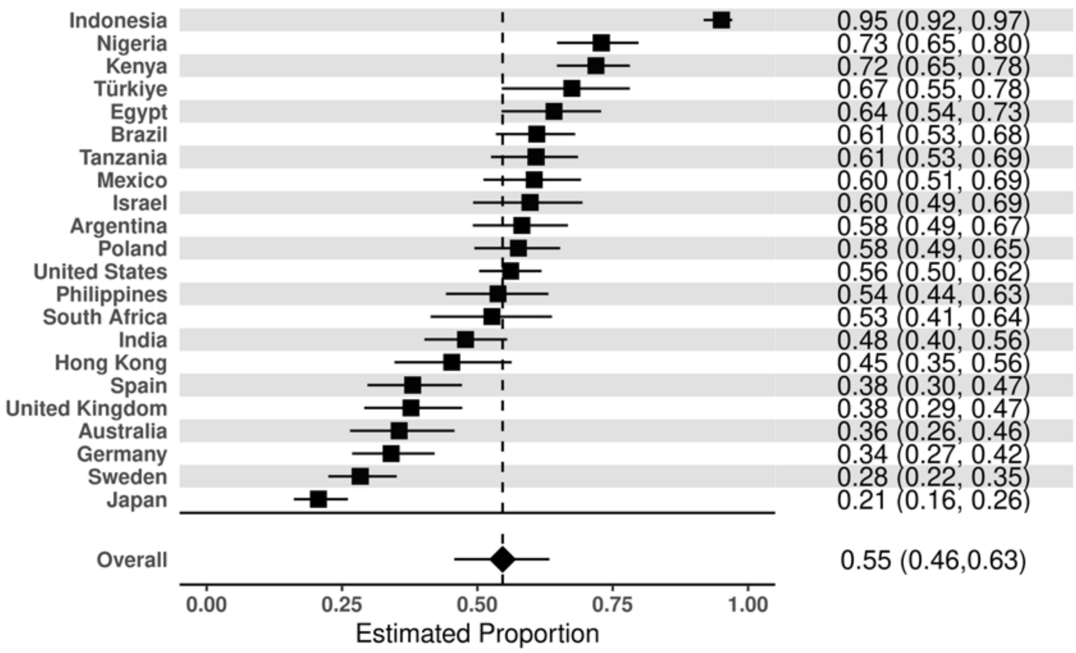

Probability-scale: τ=0.209;
Logit-scale: τ=0.842; Q-profile 95% CI [0.609, 1.148]; I^2=96.71;
Plot is based on back transformed bounds after using approximate logit SE that aren't guarenteed to match the robust SE of a proportion.

Figure S34. Forest plot for `Immigration status`-`Born in another country`

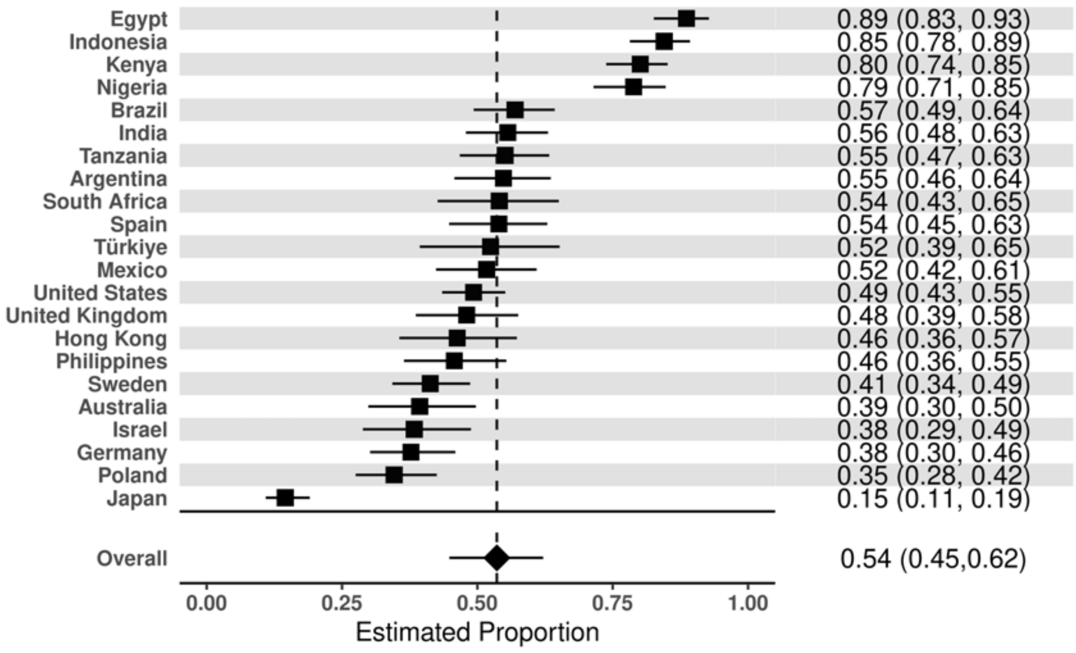

Probability-scale: τ=0.205;
Logit-scale: τ=0.826; Q-profile 95% CI [0.604, 1.130]; I^2=96.58;
Plot is based on back transformed bounds after using approximate logit SE that aren't guarenteed to match the robust SE of a proportion.

Figure S35. Forest plot for `Age group`-`(Ref: 18-24) 25-29`

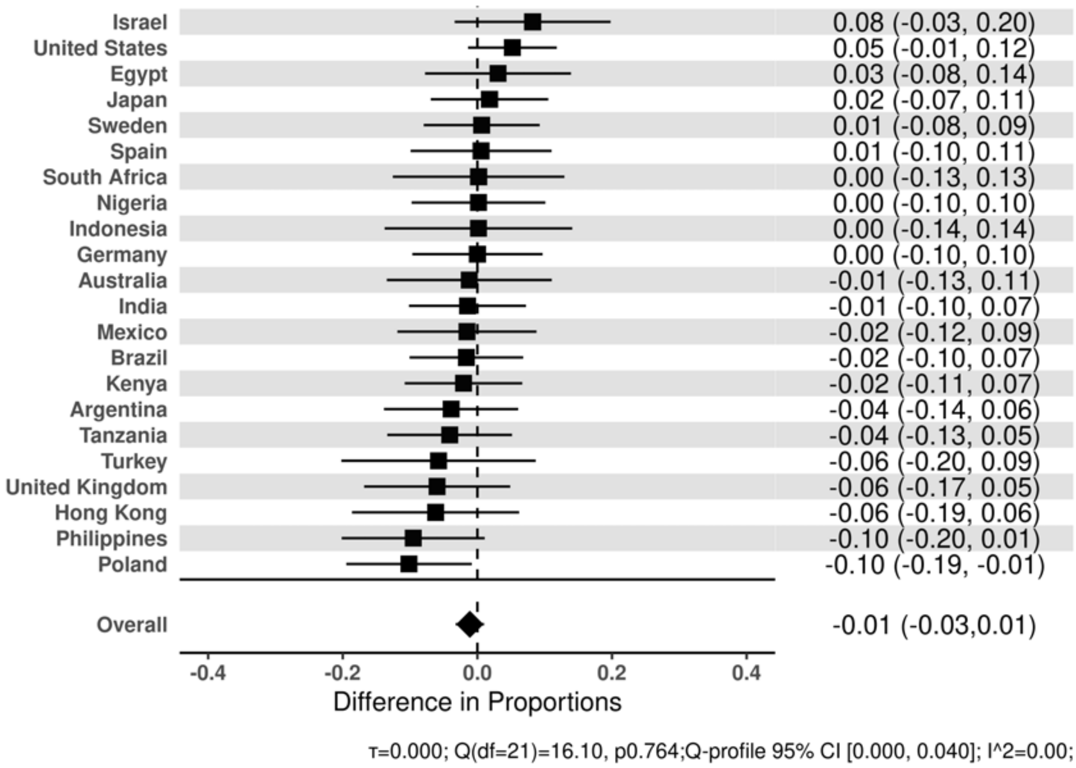

Figure S36. Forest plot for `Age group`-`(Ref: 18-24) 30-39`

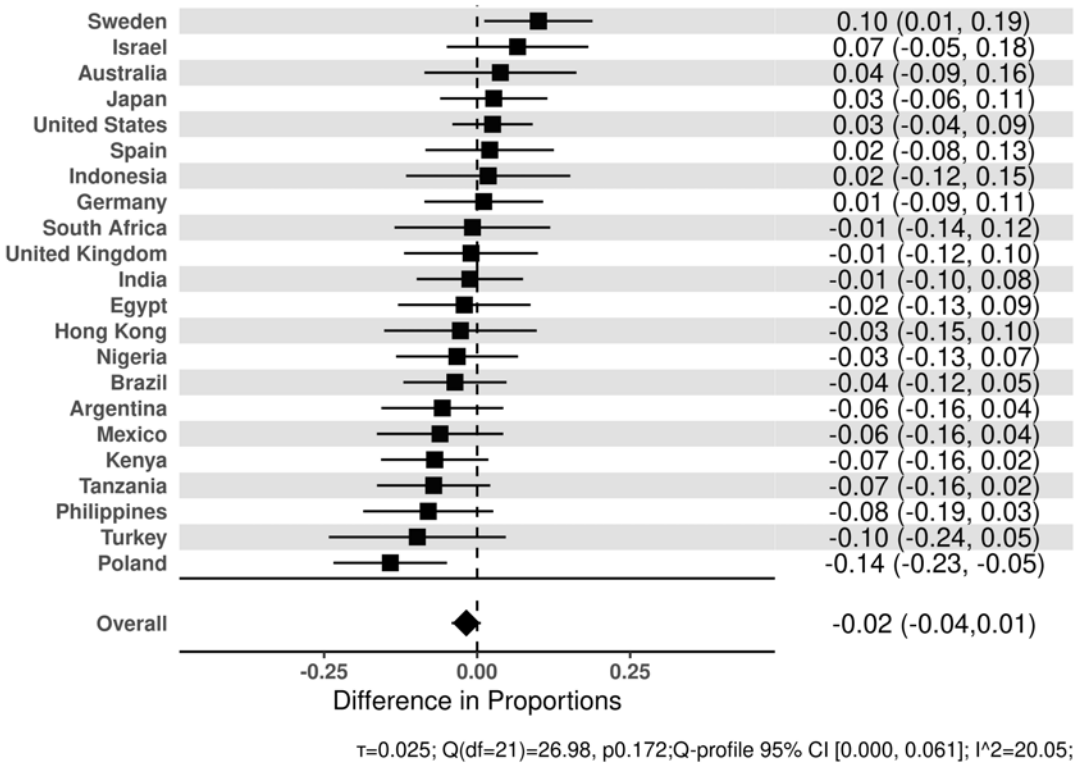

Figure S37. Forest plot for `Age group`-`(Ref: 18-24) 40-49`

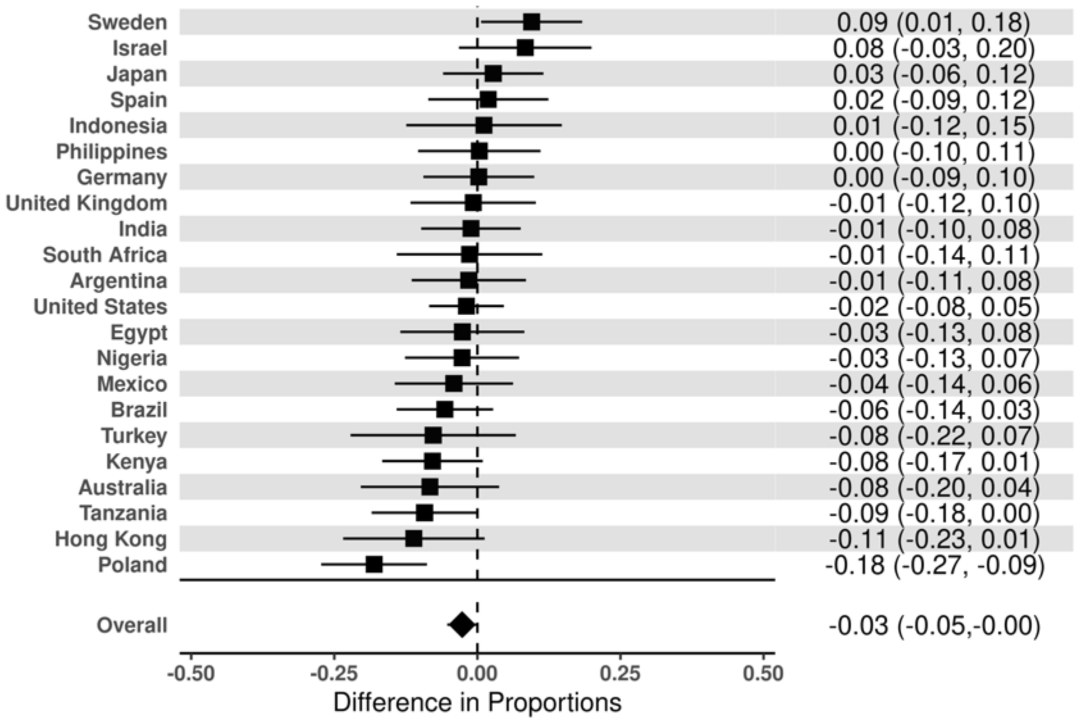

τ=0.035; Q(df=21)=31.86, p0.060;Q-profile 95% CI [0.000, 0.069]; I^2=32.62;

Figure S38. Forest plot for `Age group`-`(Ref: 18-24) 50-59`

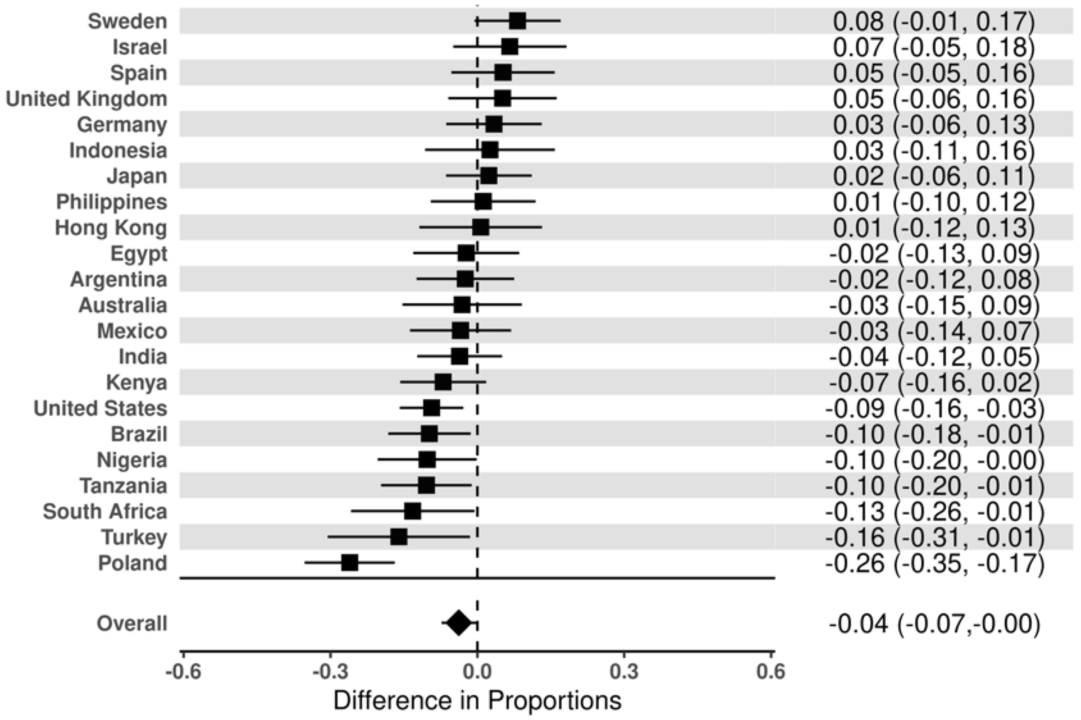

τ=0.066; Q(df=21)=58.01, p<.001;Q-profile 95% CI [0.038, 0.102]; I^2=62.99;

Figure S39. Forest plot for `Age group`-`(Ref: 18-24) 60-69`

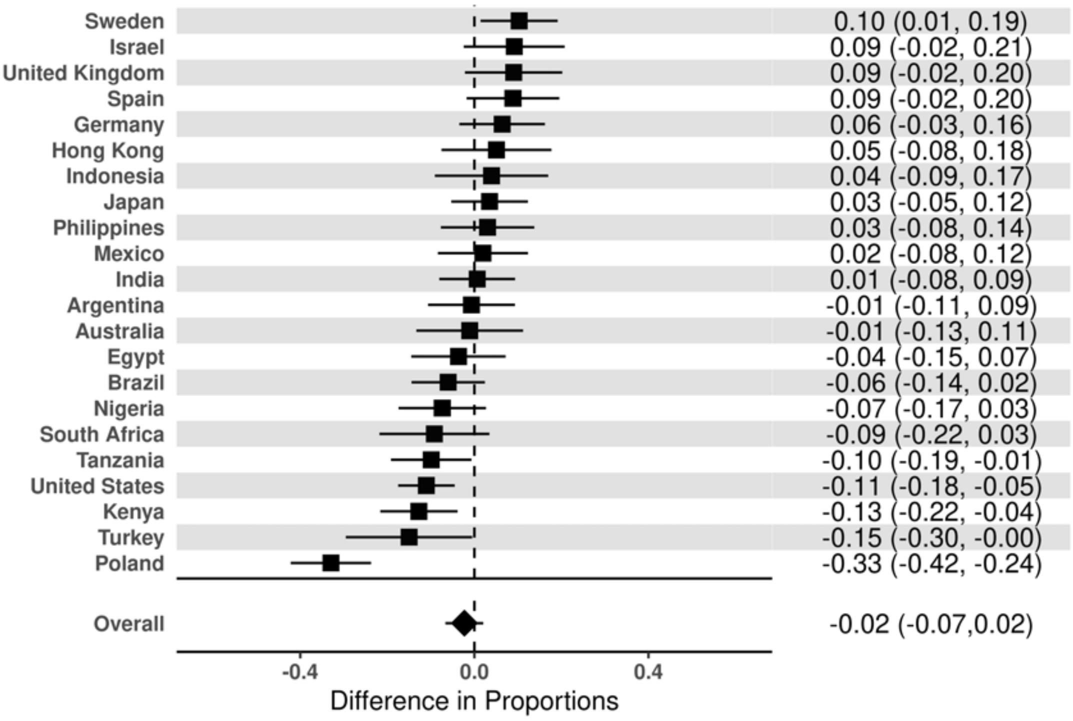

τ=0.090; Q(df=21)=91.34, p<.001;Q-profile 95% CI [0.059, 0.132]; I^2=75.70;

Figure S40. Forest plot for `Age group`-`(Ref: 18-24) 70-79`

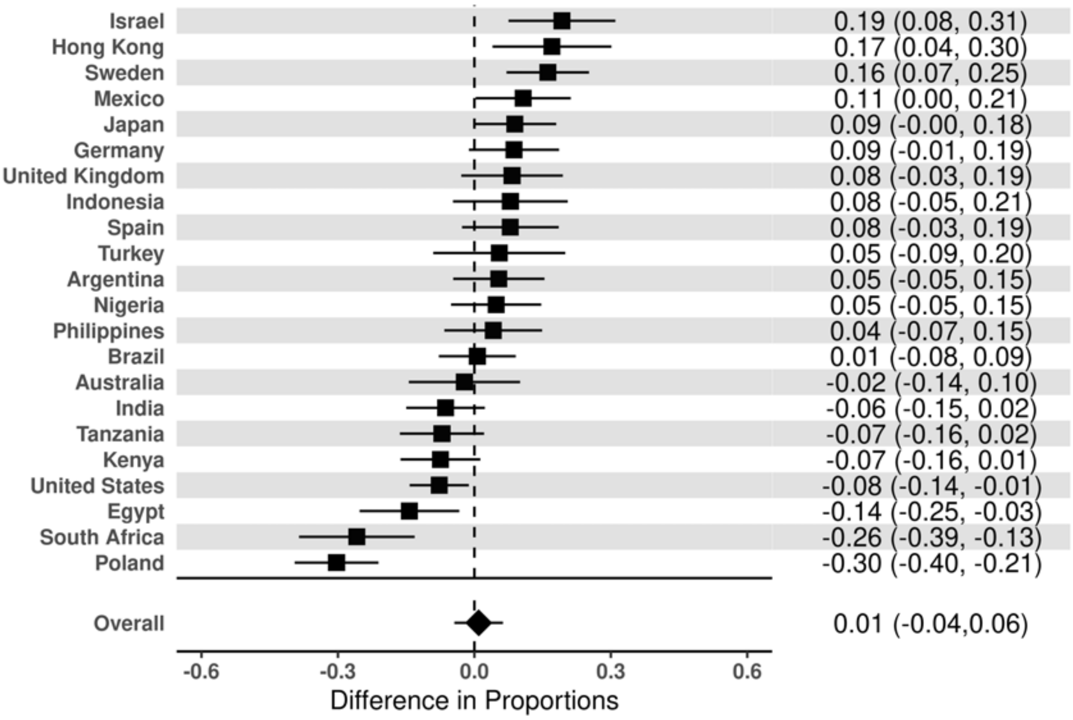

τ=0.116; Q(df=21)=125.23, p<.001;Q-profile 95% CI [0.079, 0.165]; I^2=83.83;

Figure S41. Forest plot for `Age group`-`(Ref: 18-24) 80 or older`

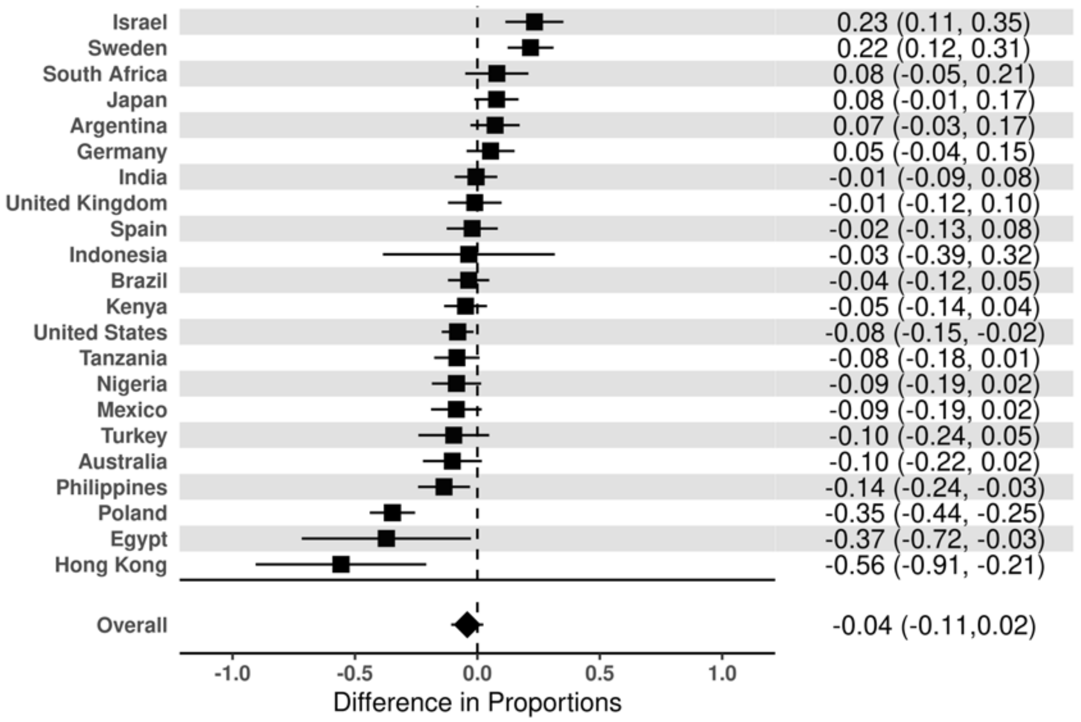

τ=0.141; Q(df=21)=131.01, p<.001;Q-profile 95% CI [0.083, 0.193]; I^2=87.57;

Figure S42. Forest plot for `Age group`-`(Ref: 25-29) 30-39`

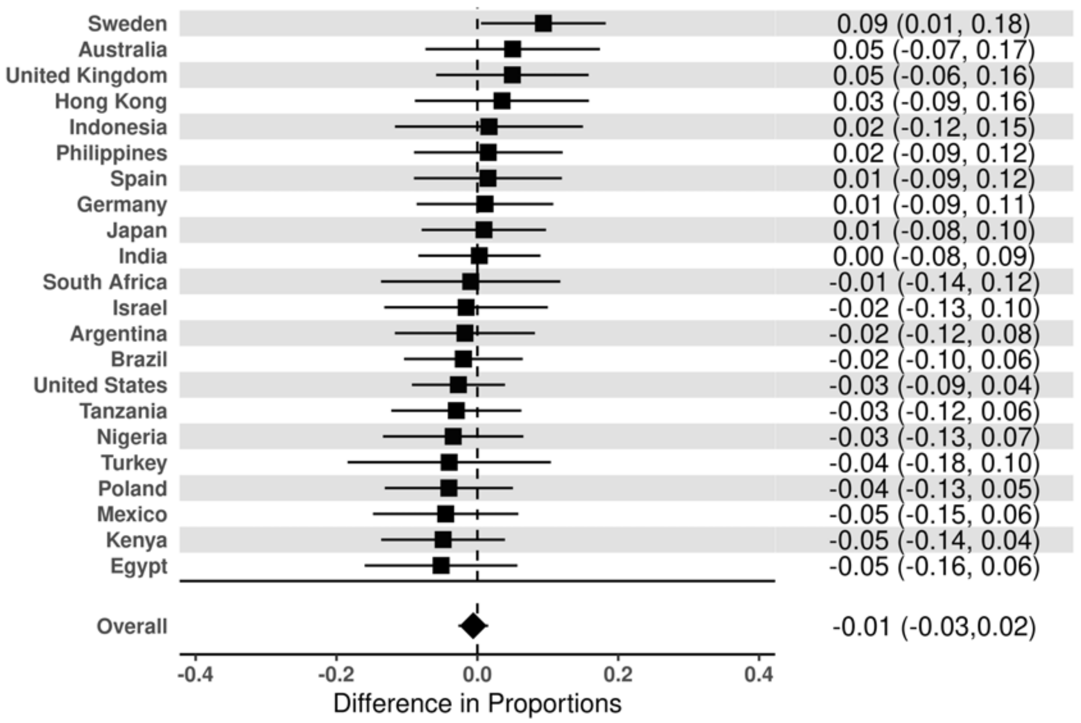

τ=0.000; Q(df=21)=11.83, p0.944;Q-profile 95% CI [0.000, 0.032]; I^2=0.00;

Figure S43. Forest plot for `Age group`-`(Ref: 25-29) 40-49`

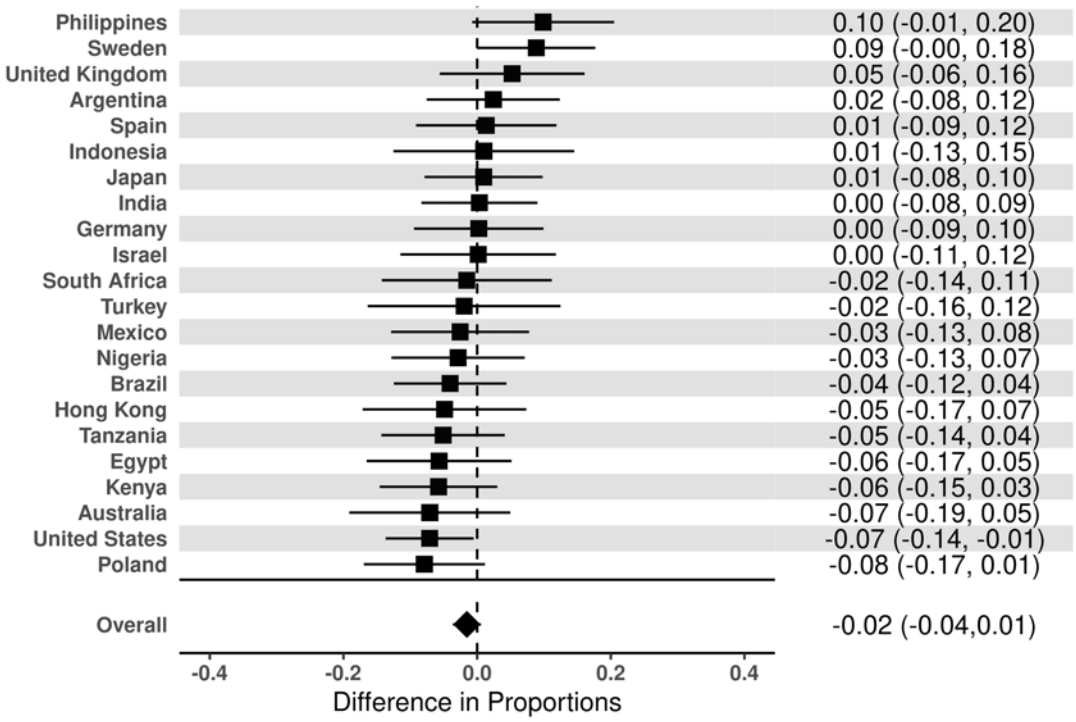

τ=0.000; Q(df=21)=21.19, p0.447;Q-profile 95% CI [0.000, 0.051]; I^2=0.00;

Figure S44. Forest plot for `Age group`-`(Ref: 25-29) 50-59`

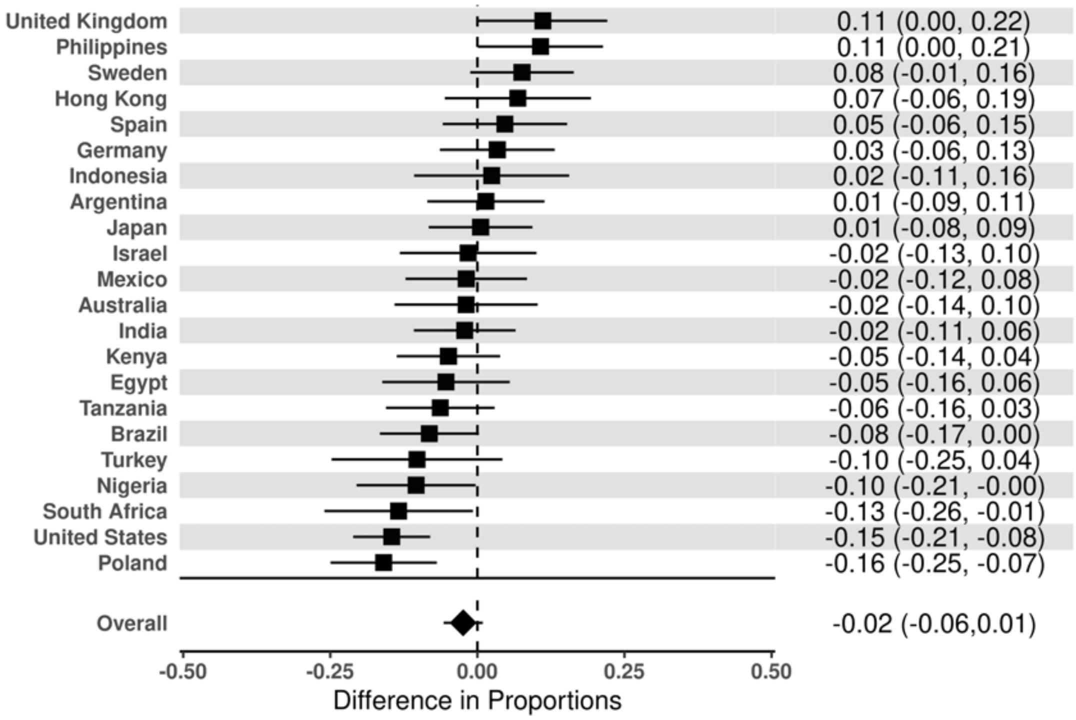

τ=0.060; Q(df=21)=54.43, p<.001;Q-profile 95% CI [0.034, 0.096]; I^2=58.44;

Figure S45. Forest plot for `Age group`-`(Ref: 25-29) 60-69`

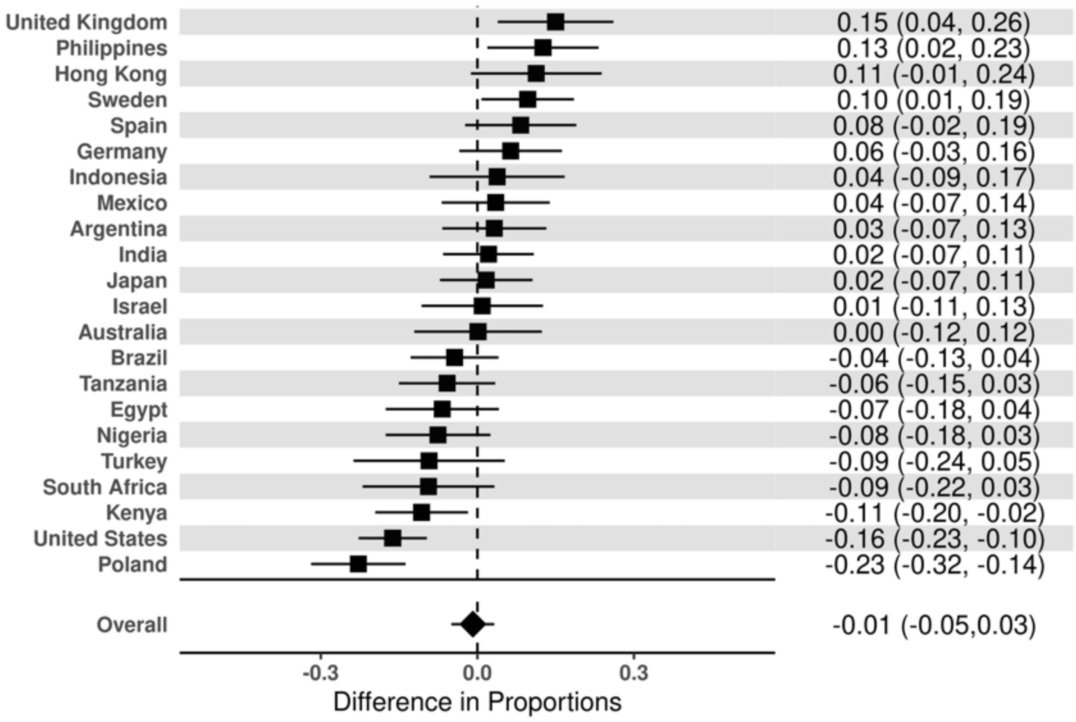

τ=0.083; Q(df=21)=85.62, p<.001;Q-profile 95% CI [0.054, 0.124]; I^2=72.77;

Figure S46. Forest plot for `Age group`-`(Ref: 25-29) 70-79`

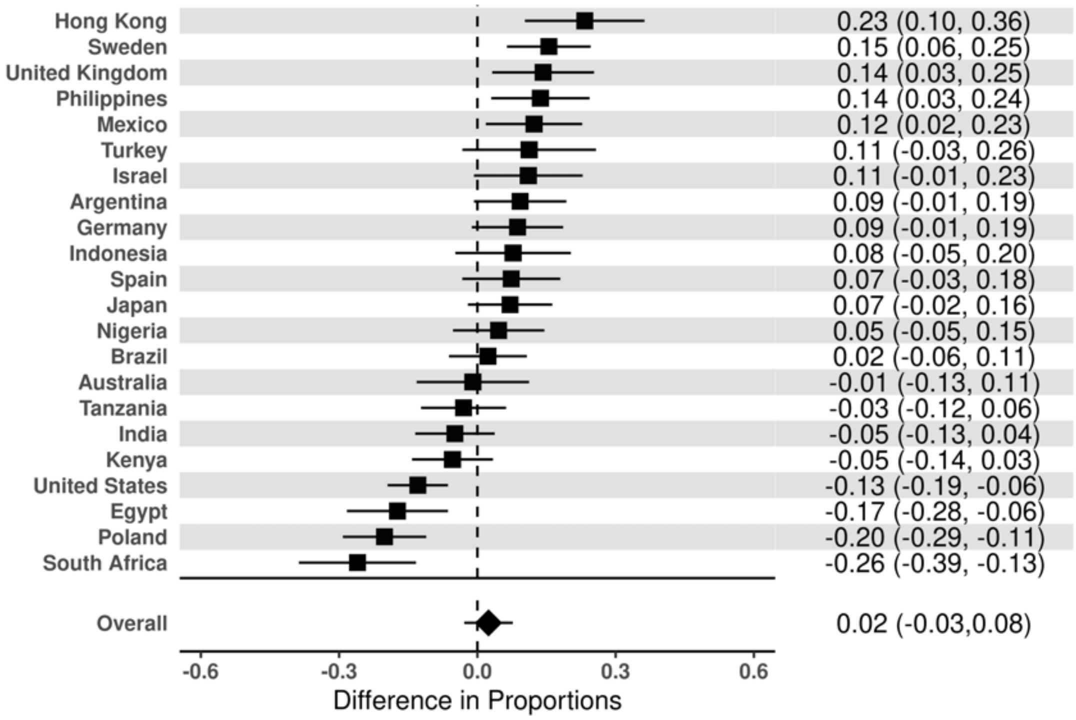

τ=0.114; Q(df=21)=122.17, p<.001;Q-profile 95% CI [0.076, 0.162]; I^2=83.38;

Figure S47. Forest plot for `Age group`-`(Ref: 25-29) 80 or older`

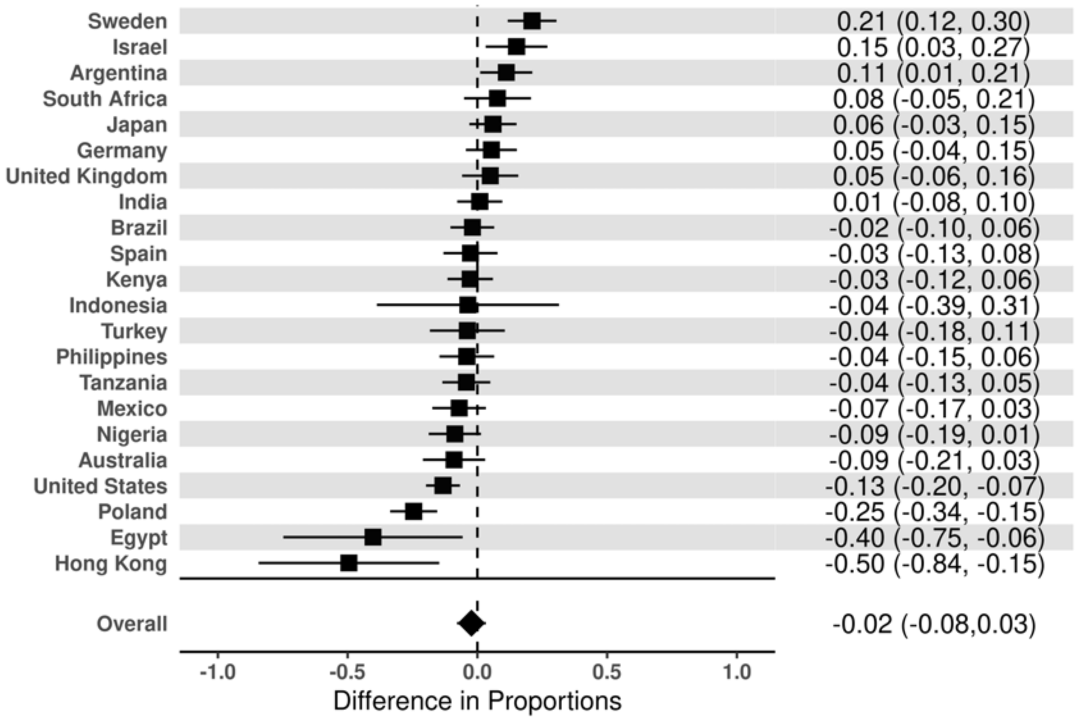

τ=0.116; Q(df=21)=98.50, p<.001;Q-profile 95% CI [0.061, 0.158]; I^2=82.62;

Figure S48. Forest plot for `Age group`-`(Ref: 30-39) 40-49`

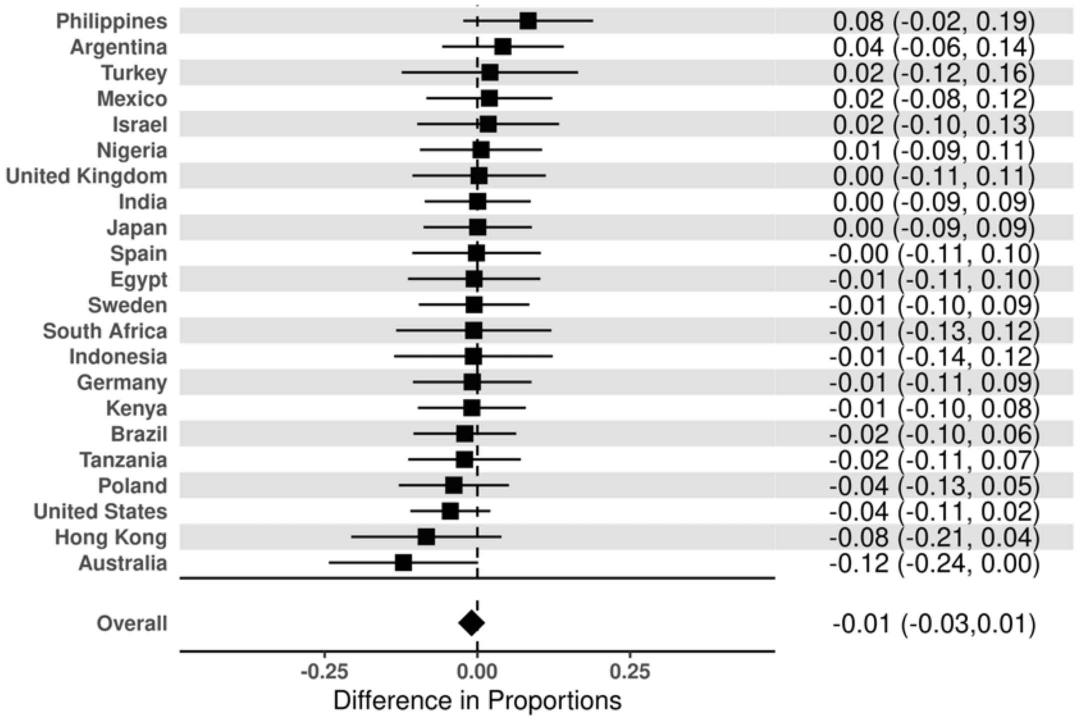

τ=0.000; Q(df=21)=11.11, p0.961;Q-profile 95% CI [0.000, 0.030]; I^2=0.00;

Figure S49. Forest plot for `Age group`-`(Ref: 30-39) 50-59`

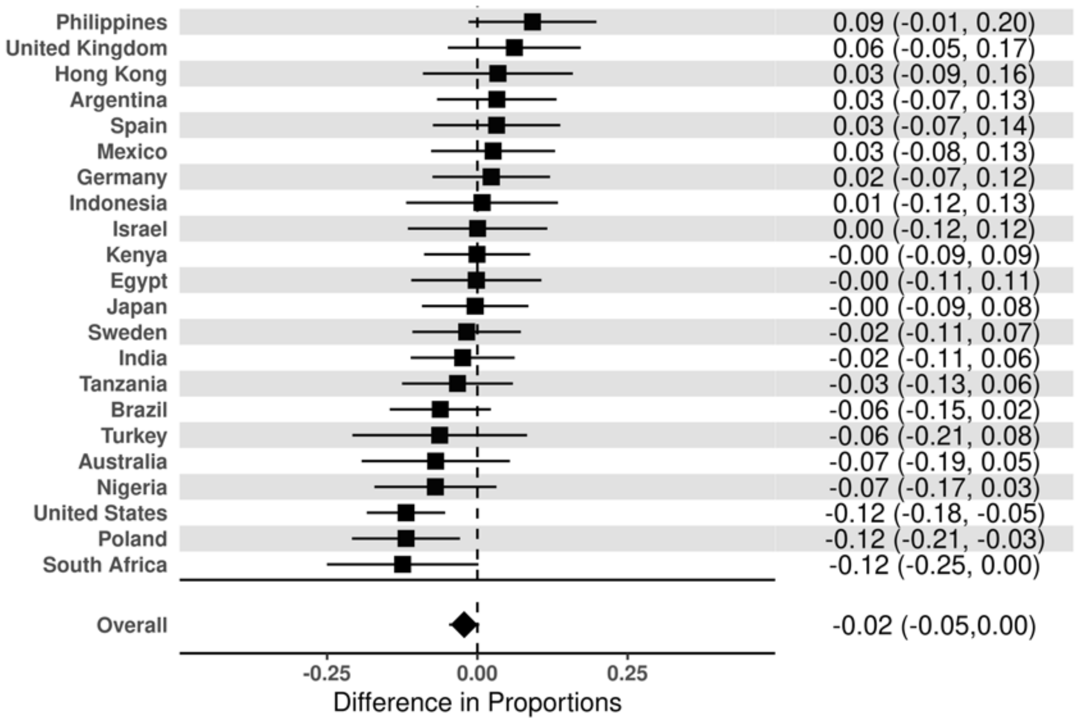

τ=0.031; Q(df=21)=30.04, p0.091;Q-profile 95% CI [0.000, 0.065]; I^2=26.73;

Figure S50. Forest plot for `Age group`-`(Ref: 30-39) 60-69`

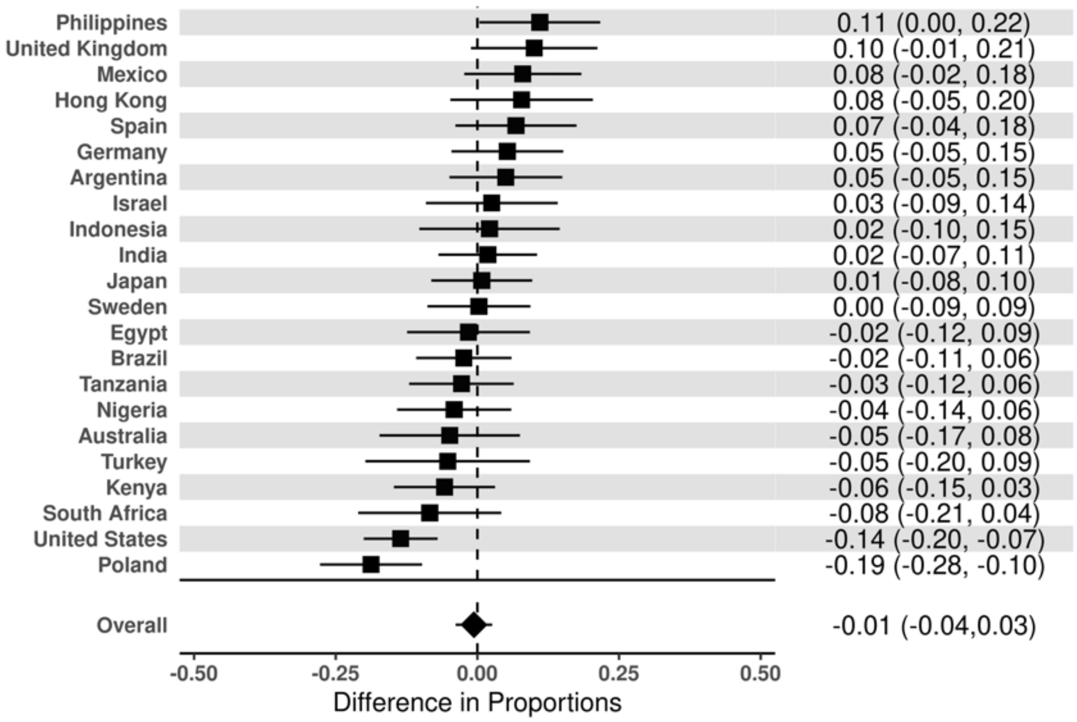

τ=0.057; Q(df=21)=52.49, p<.001;Q-profile 95% CI [0.032, 0.093]; I^2=55.40;

Figure S51. Forest plot for `Age group`-`(Ref: 30-39) 70-79`

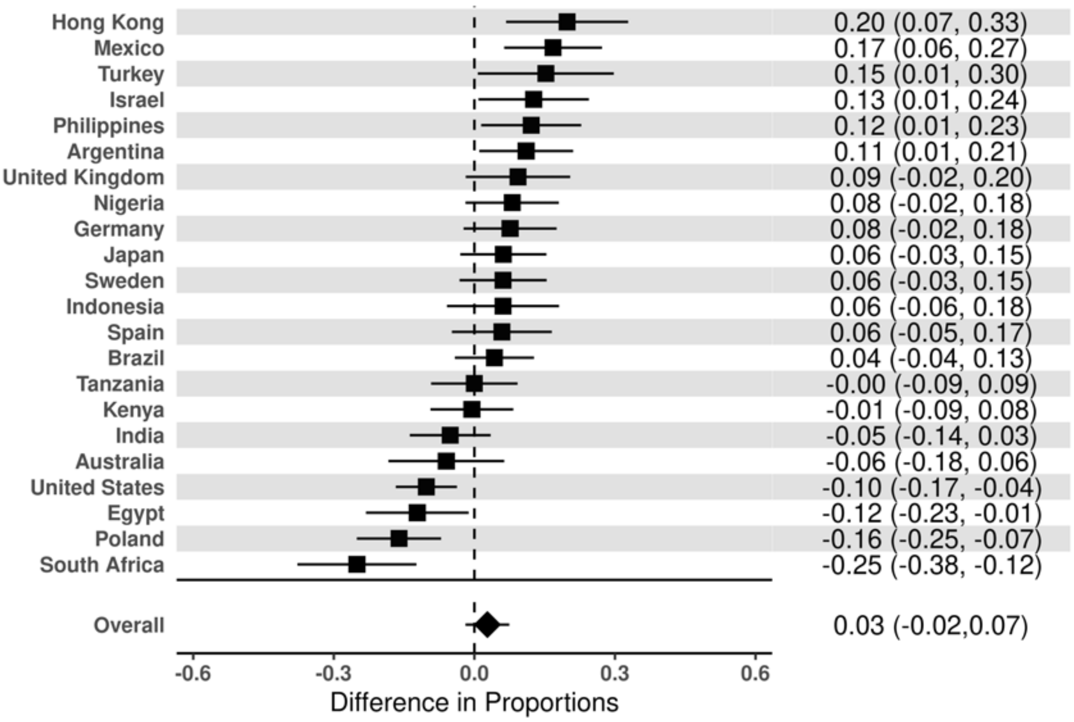

τ=0.098; Q(df=21)=92.57, p<.001;Q-profile 95% CI [0.061, 0.141]; I^2=78.80;

Figure S52. Forest plot for `Age group`-`(Ref: 30-39) 80 or older`

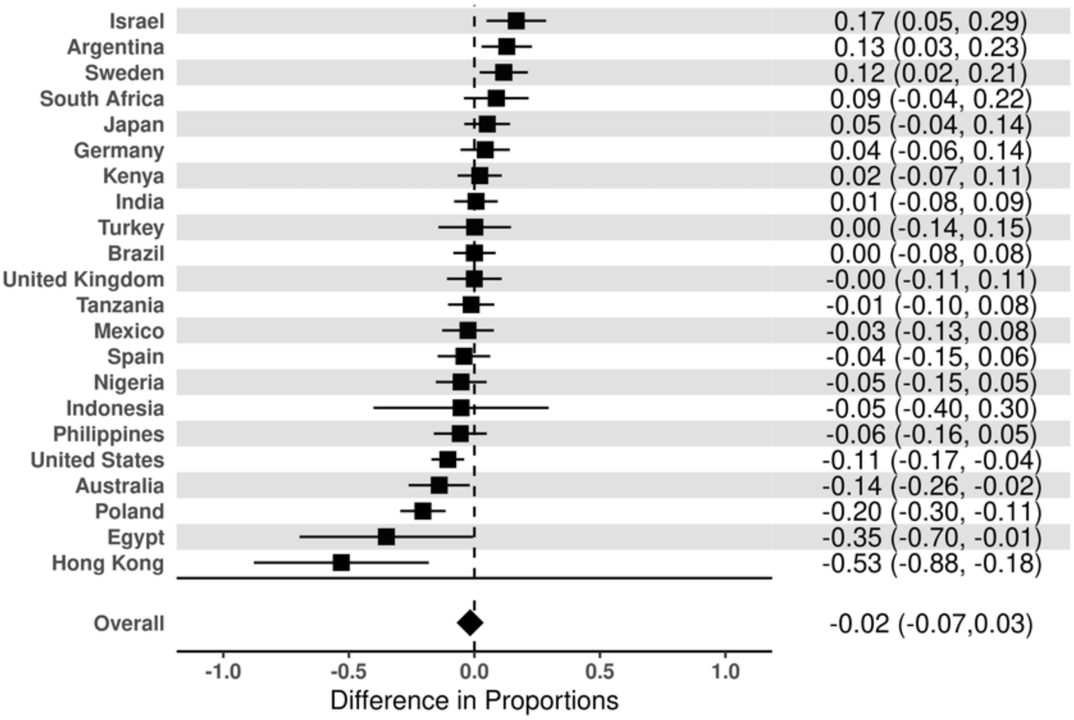

τ=0.101; Q(df=21)=73.10, p<.001;Q-profile 95% CI [0.043, 0.137]; I^2=78.32;

Figure S53. Forest plot for `Age group`-`(Ref: 40-49) 50-59`

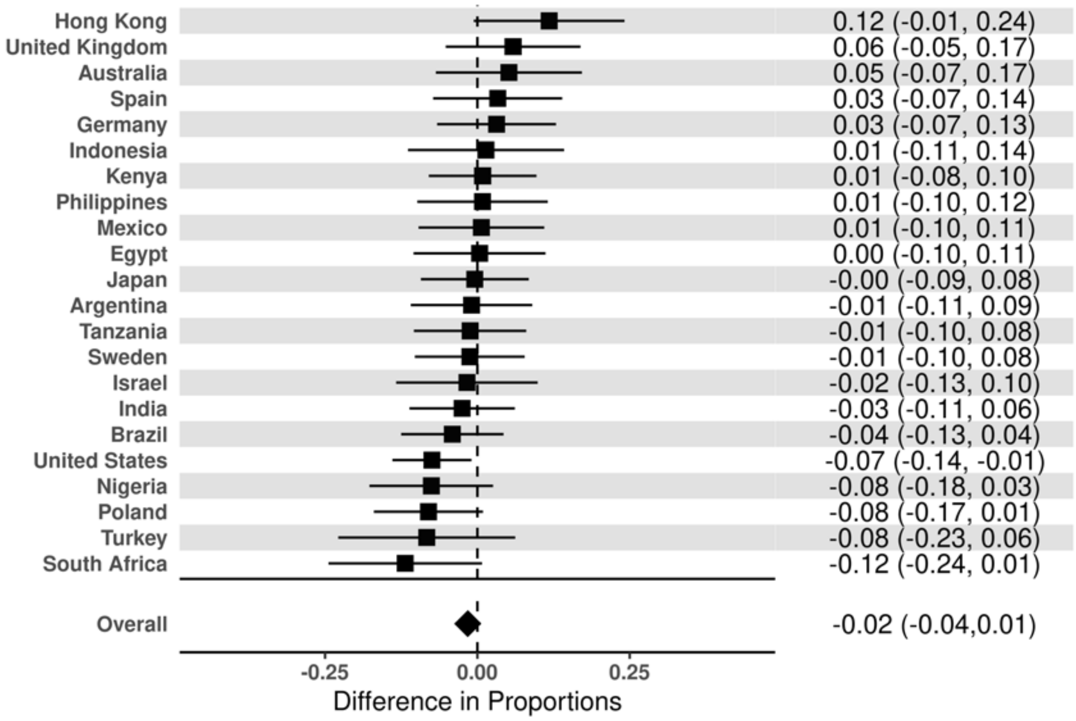

τ=0.000; Q(df=21)=20.52, p0.489;Q-profile 95% CI [0.000, 0.045]; I^2=0.00;

Figure S54. Forest plot for `Age group`-`(Ref: 40-49) 60-69`

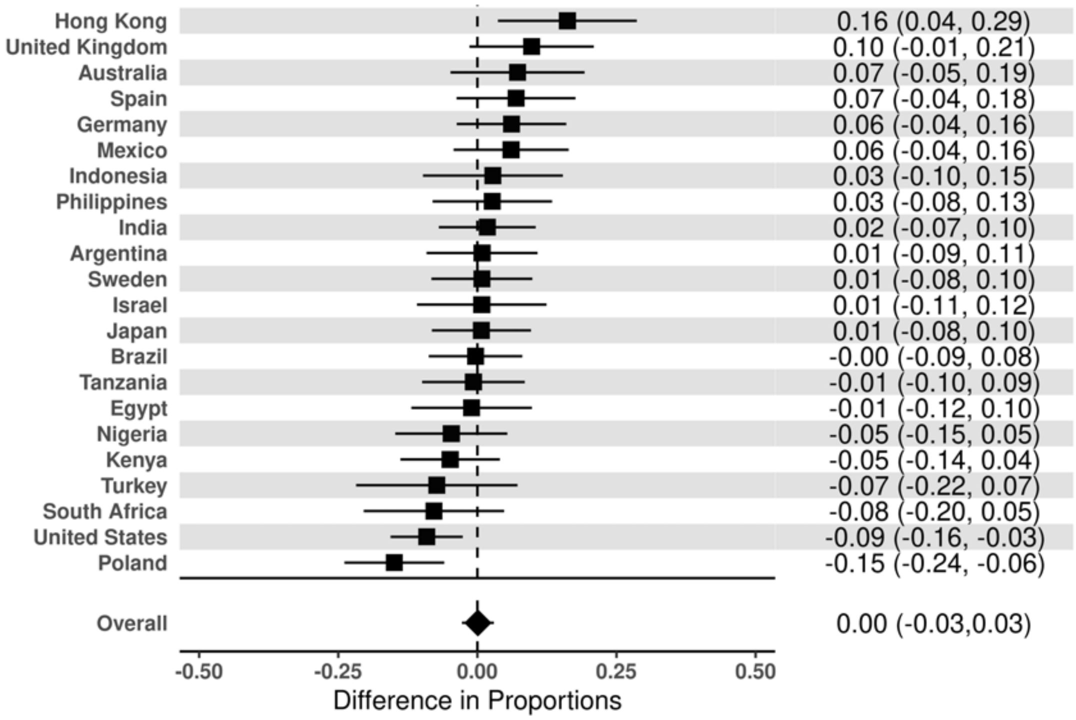

τ=0.045; Q(df=21)=38.41, p0.012;Q-profile 95% CI [0.011, 0.077]; I^2=43.72;

Figure S55. Forest plot for `Age group`-`(Ref: 40-49) 70-79`

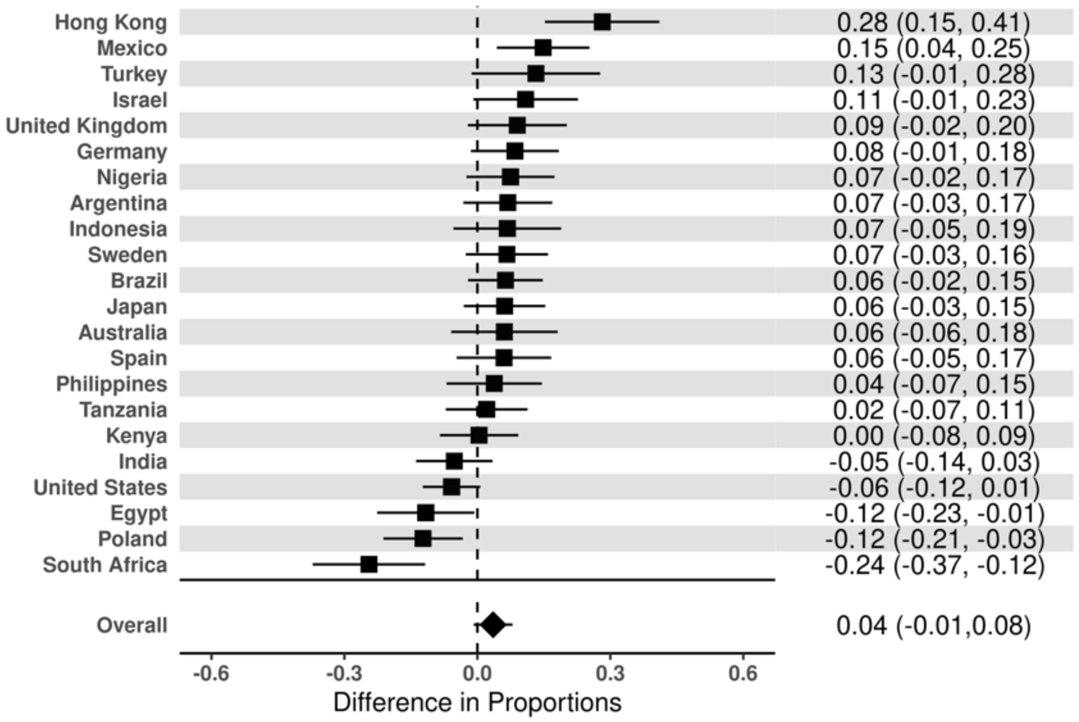

τ=0.090; Q(df=21)=76.20, p<.001;Q-profile 95% CI [0.052, 0.130]; I^2=75.94;

Figure S56. Forest plot for `Age group`-`(Ref: 40-49) 80 or older`

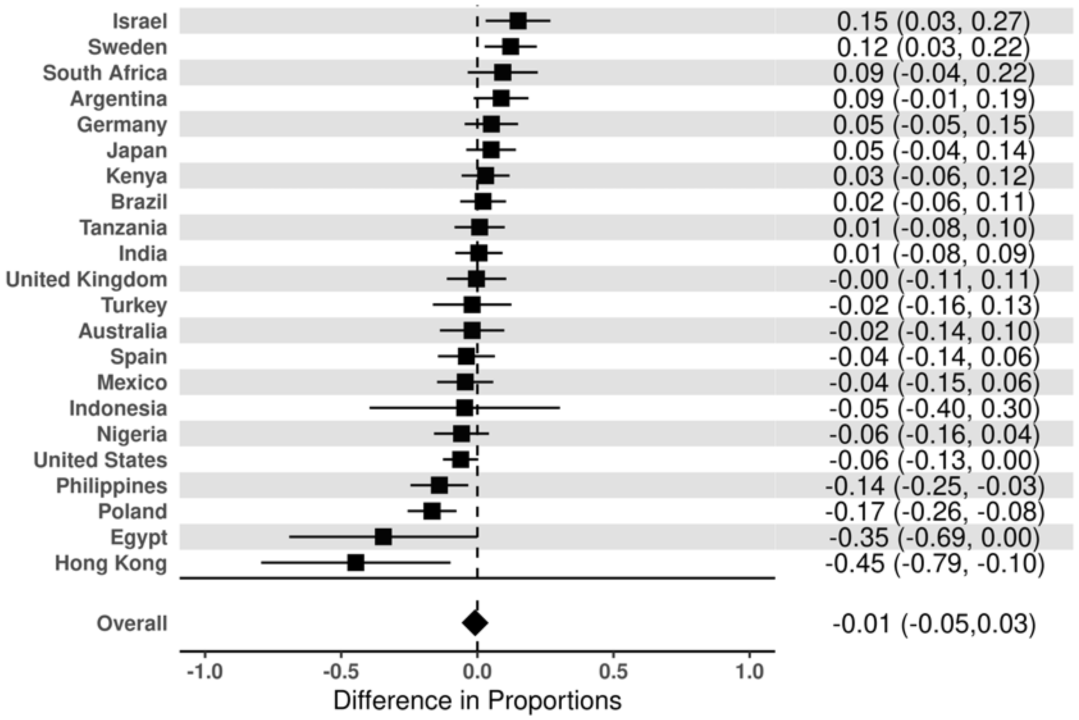

τ=0.081; Q(df=21)=56.15, p<.001;Q-profile 95% CI [0.029, 0.113]; I^2=69.97;

Figure S57. Forest plot for `Age group`-`(Ref: 50-59) 60-69`

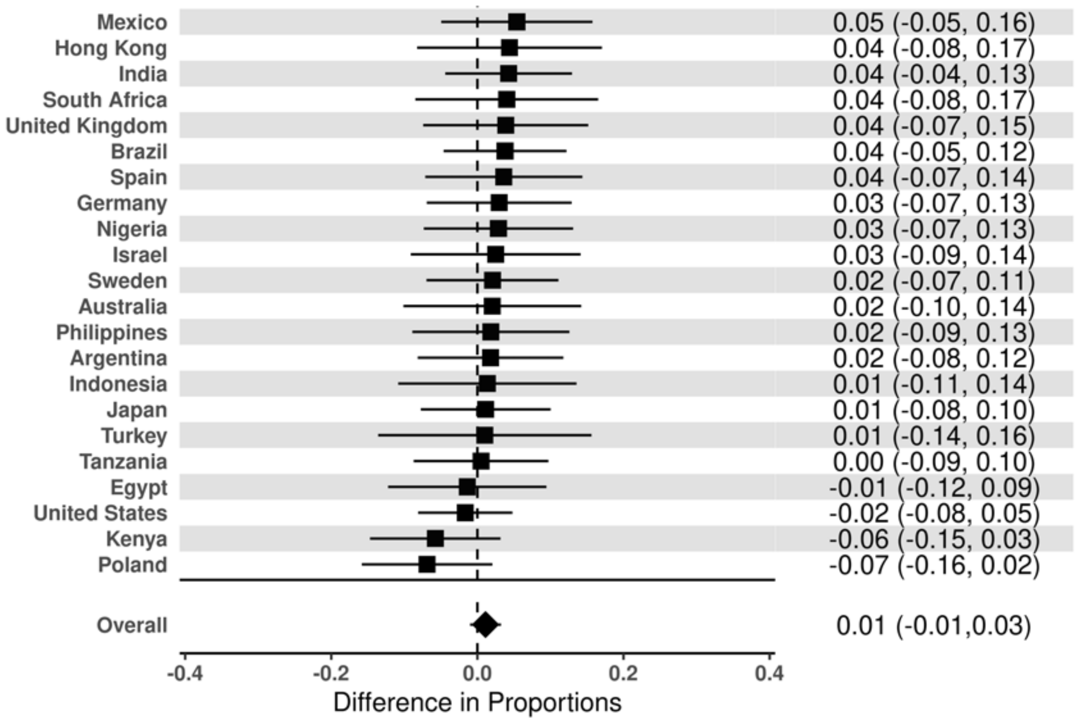

τ=0.000; Q(df=21)=9.16, p0.988;Q-profile 95% CI [0.000, 0.030]; I^2=0.00;

Figure S58. Forest plot for `Age group`-`(Ref: 50-59) 70-79`

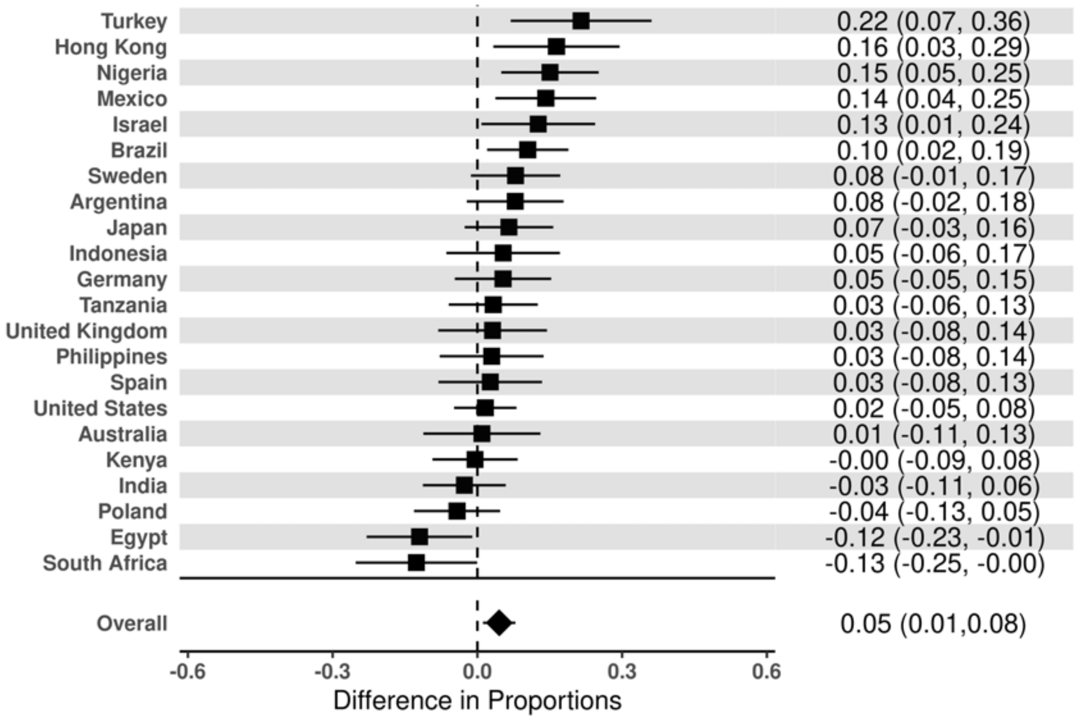

τ=0.061; Q(df=21)=45.54, p0.001;Q-profile 95% CI [0.017, 0.093]; I^2=58.75;

Figure S59. Forest plot for `Age group`-`(Ref: 50-59) 80 or older`

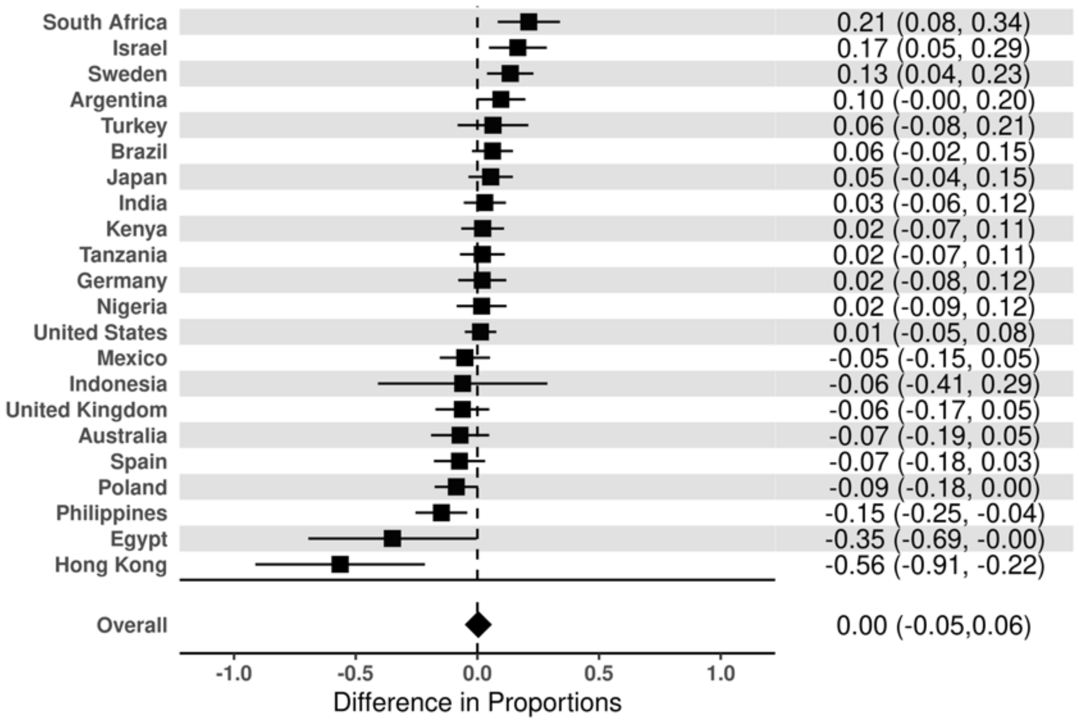

τ=0.108; Q(df=21)=63.61, p<.001;Q-profile 95% CI [0.028, 0.142]; I^2=80.35;

Figure S60. Forest plot for `Age group`-`(Ref: 60-69) 70-79`

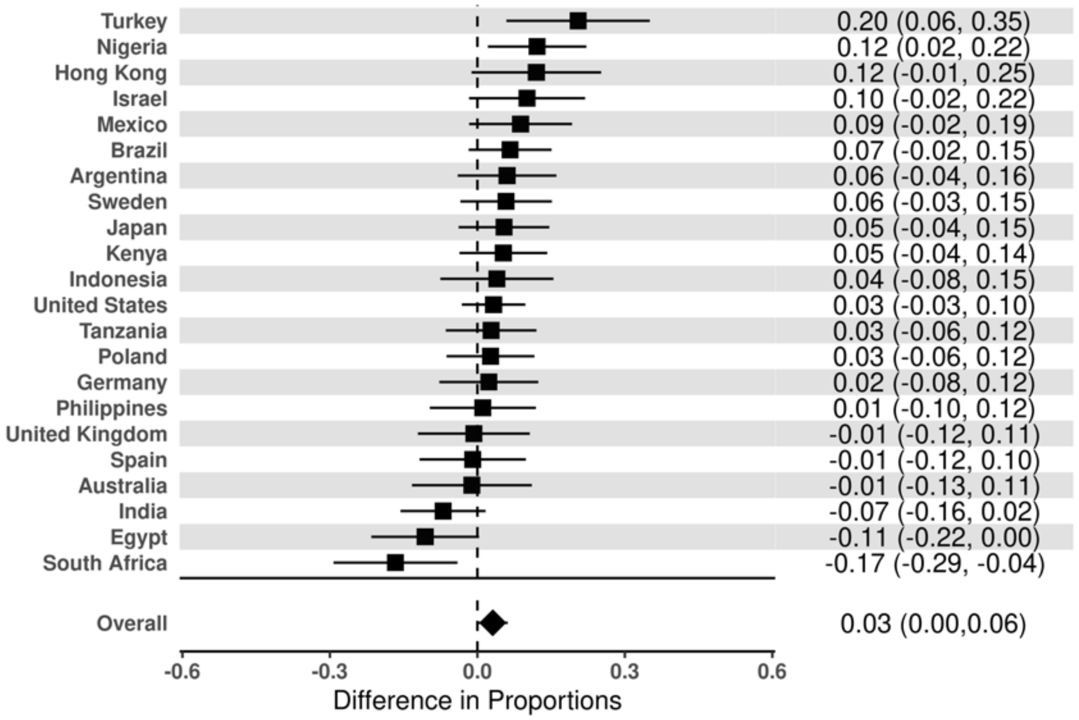

τ=0.050; Q(df=21)=36.96, p0.017;Q-profile 95% CI [0.000, 0.080]; I^2=49.32;

Figure S61. Forest plot for `Age group`-`(Ref: 60-69) 80 or older`

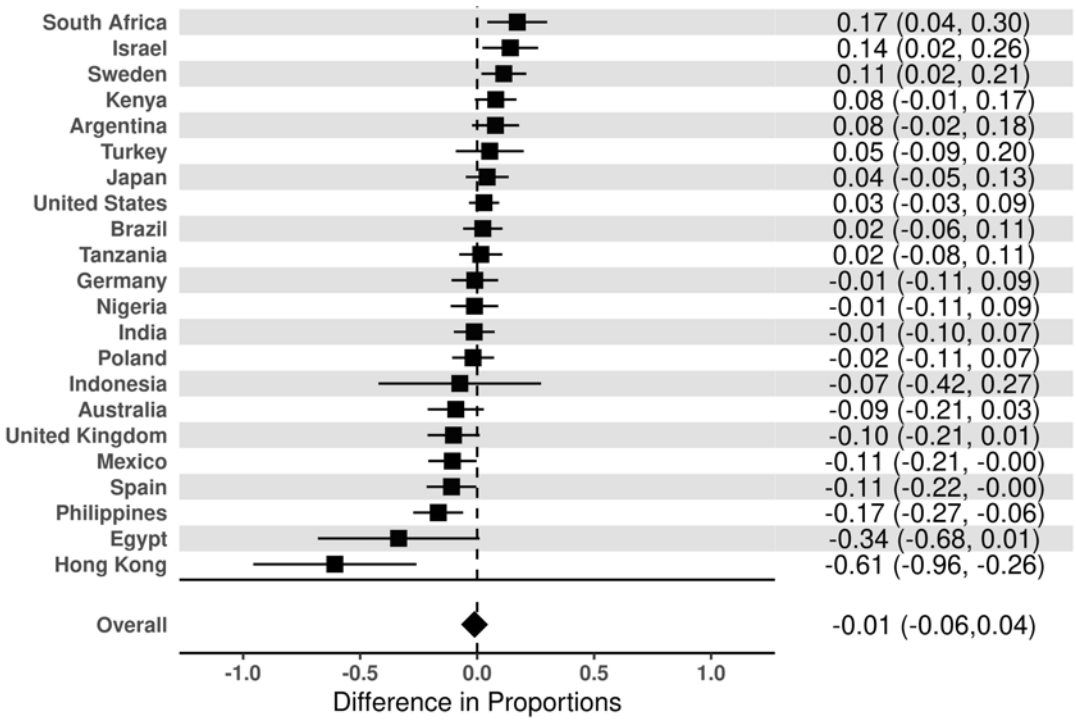

τ=0.109; Q(df=21)=64.21, p<.001;Q-profile 95% CI [0.030, 0.144]; I^2=80.64;

Figure S62. Forest plot for `Age group`-`(Ref: 70-79) 80 or older`

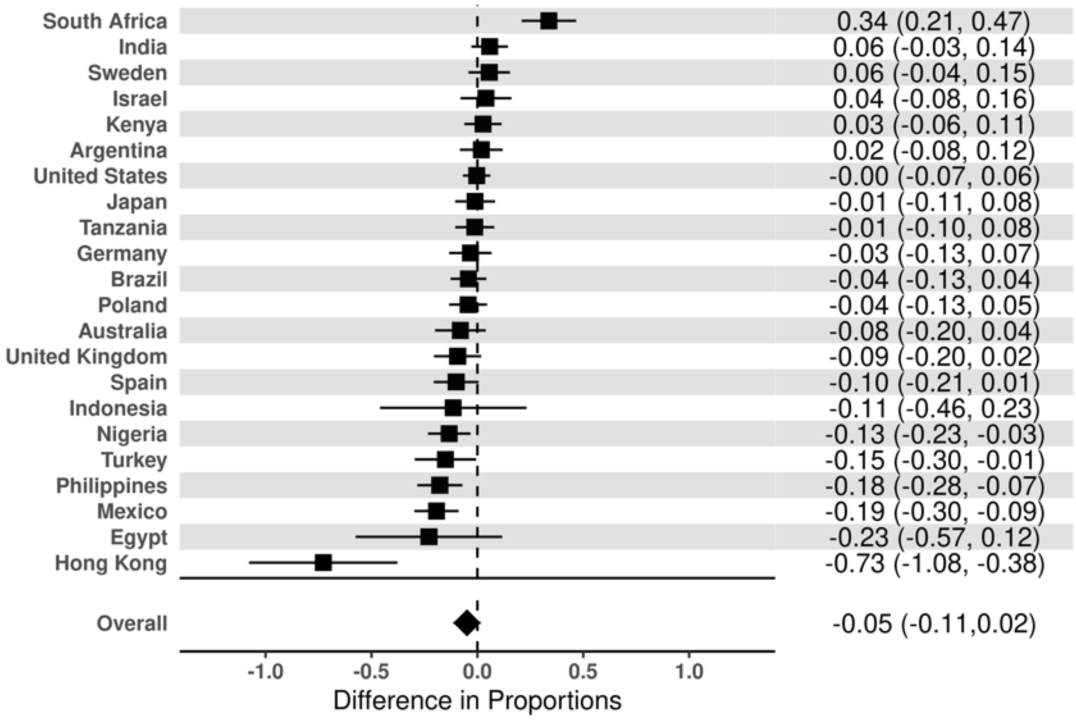

τ=0.138; Q(df=21)=86.97, p<.001;Q-profile 95% CI [0.058, 0.183]; I^2=86.88;

Figure S63. Forest plot for `Gender`-`(Ref: Male) Female`

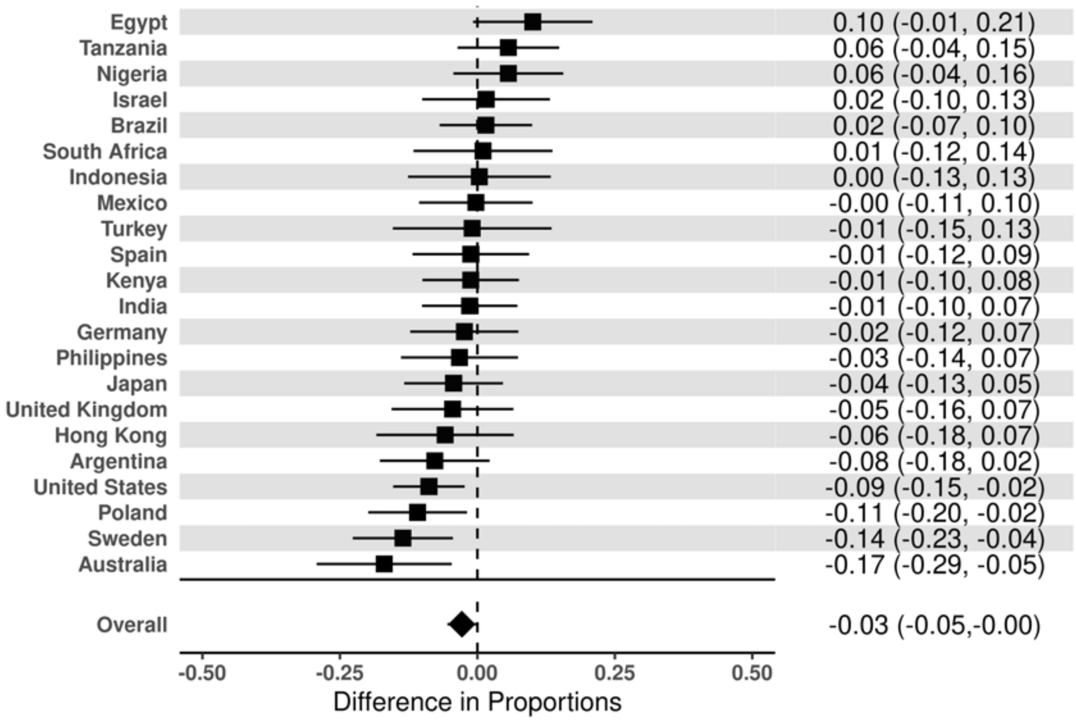

τ=0.036; Q(df=21)=32.28, p0.055;Q-profile 95% CI [0.000, 0.069]; I^2=33.31;

Figure S64. Forest plot for `Gender`-`(Ref: Male) Other`

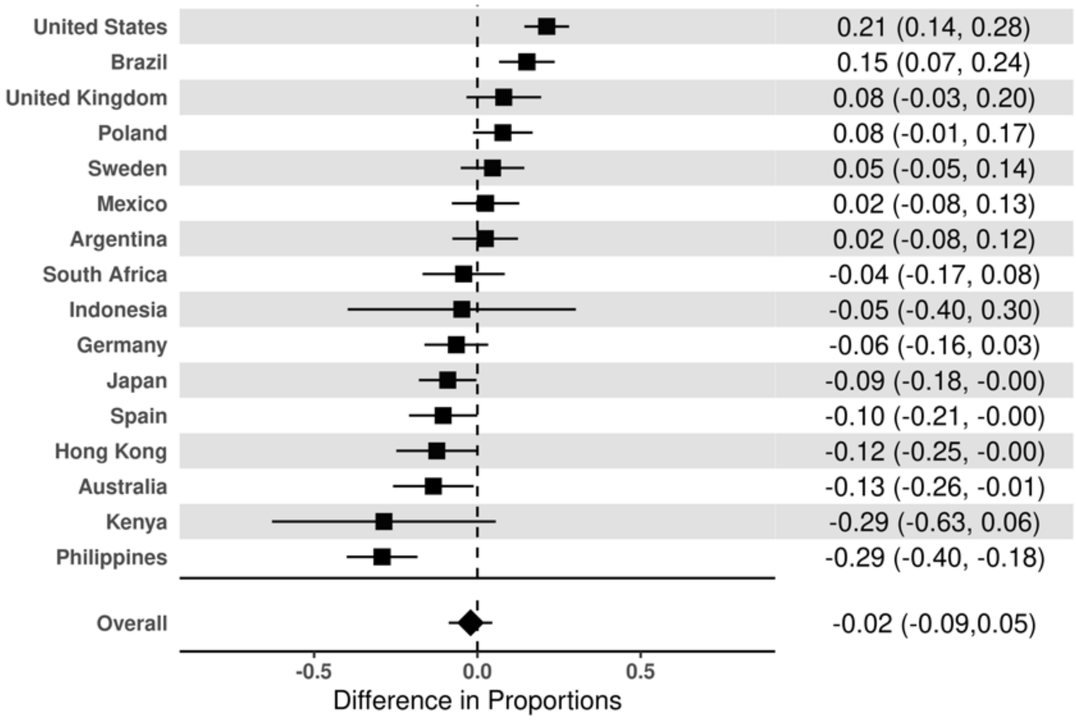

τ=0.120; Q(df=15)=103.54, p<.001;Q-profile 95% CI [0.078, 0.186]; I^2=83.17;
Excluded countries: India, Egypt, Israel, Nigeria, Tanzania, Turkiye

Figure S65. Forest plot for `Gender`-`(Ref: Female) Other`

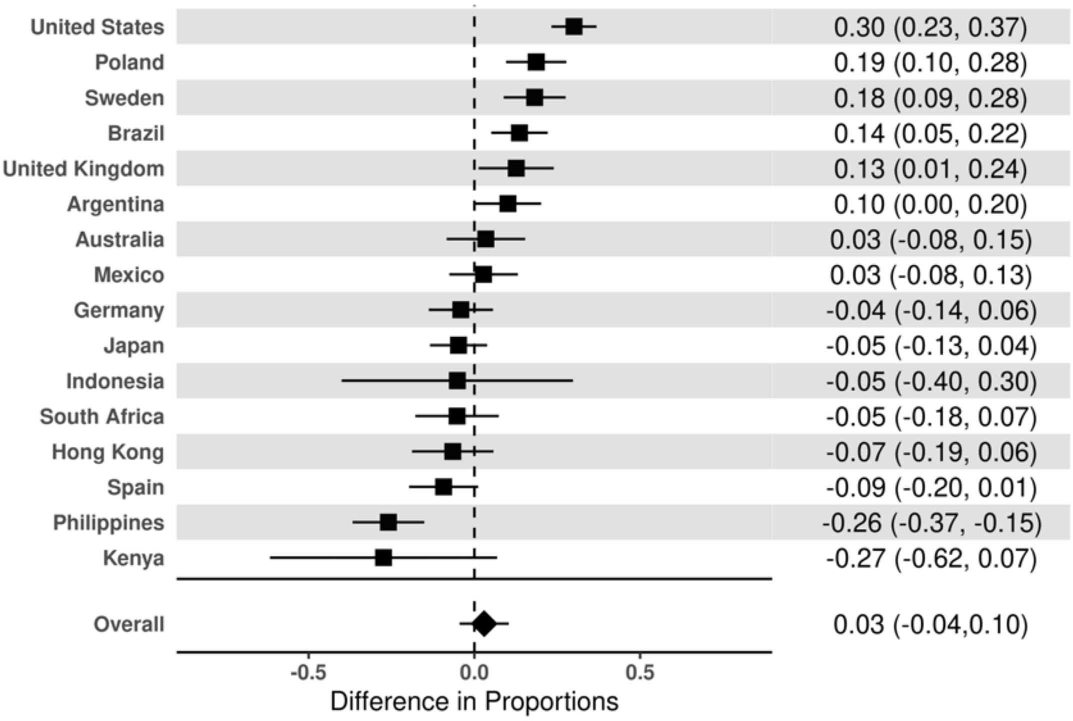

τ=0.136; Q(df=15)=130.33, p<.001;Q-profile 95% CI [0.089, 0.208]; I^2=86.79;
Excluded countries: India, Egypt, Israel, Nigeria, Tanzania, Turkiye

Figure S66. Forest plot for `Marital status`-`(Ref: Married) Separated`

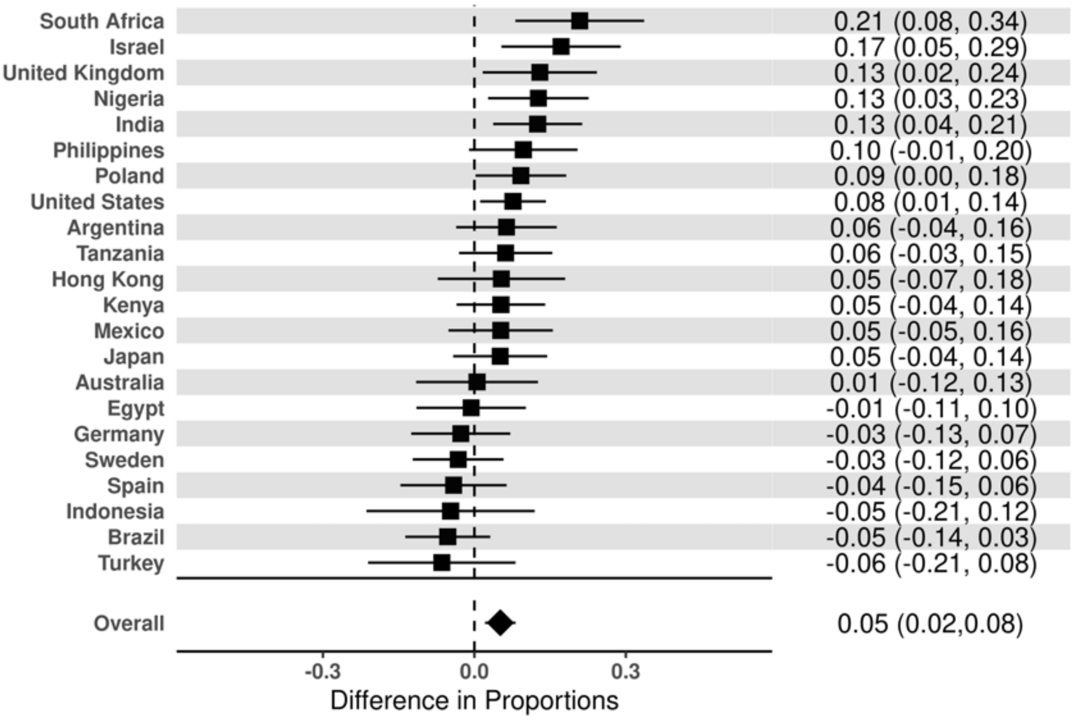

τ=0.050; Q(df=21)=38.79, p0.010;Q-profile 95% CI [0.000, 0.081]; I^2=48.44;

Figure S67. Forest plot for `Marital status`-`(Ref: Married) Divorced`

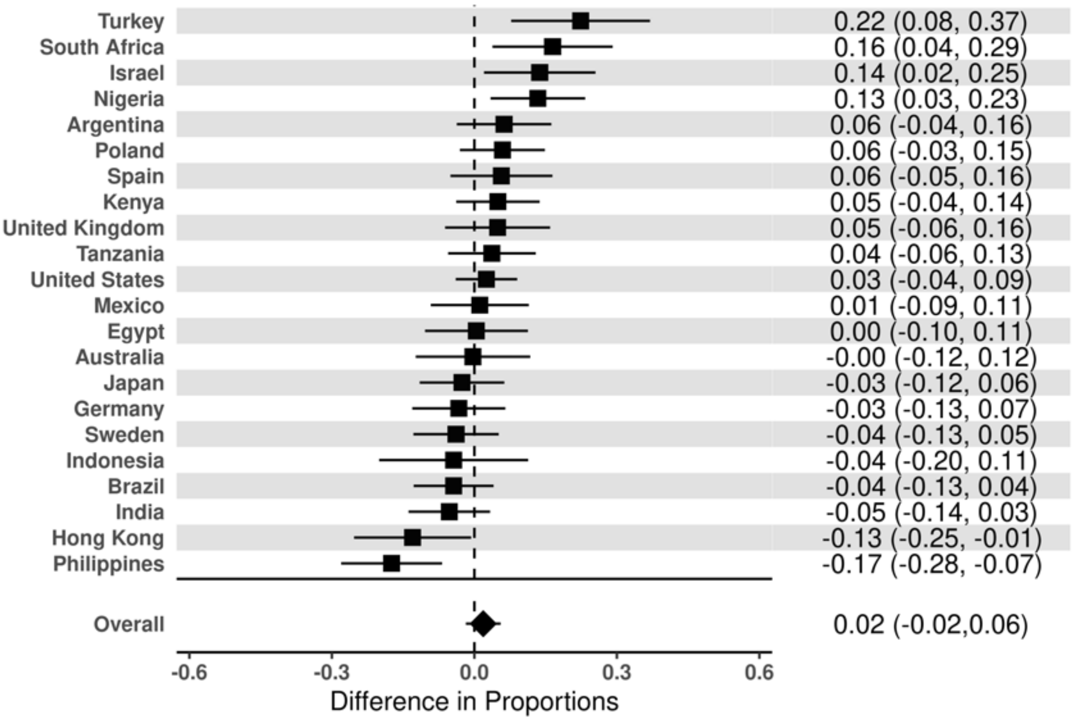

τ=0.070; Q(df=21)=52.06, p<.001;Q-profile 95% CI [0.028, 0.104]; I^2=65.29;

Figure S68. Forest plot for `Marital status`-`(Ref: Married) Widowed`

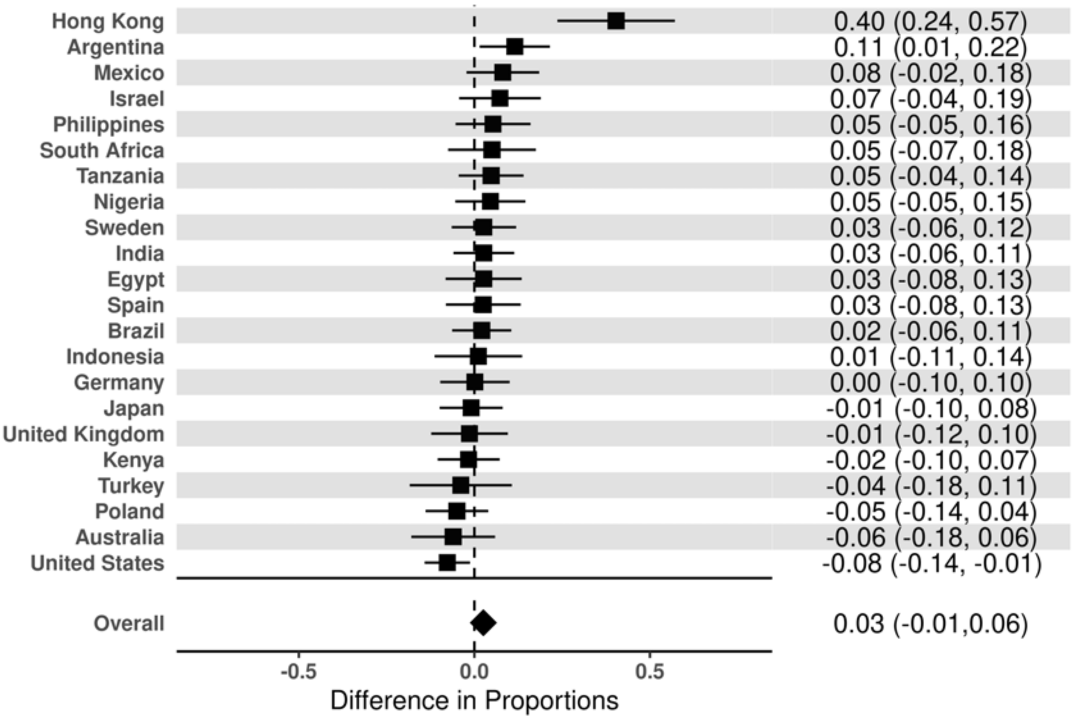

τ=0.061; Q(df=21)=41.94, p0.004;Q-profile 95% CI [0.000, 0.087]; I^2=58.64;

Figure S69. Forest plot for `Marital status`-`(Ref: Married) Single, never married`

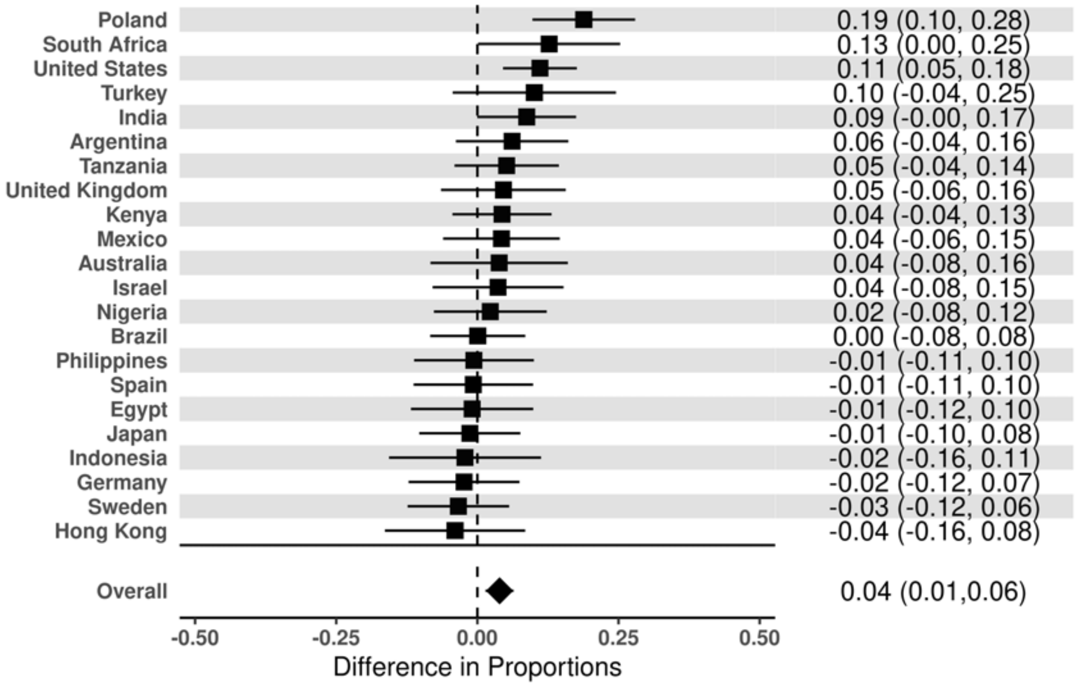

τ=0.030; Q(df=21)=29.82, p0.096;Q-profile 95% CI [0.000, 0.065]; I^2=25.58;

Figure S70. Forest plot for `Marital status`-`(Ref: Married) Domestic partner`

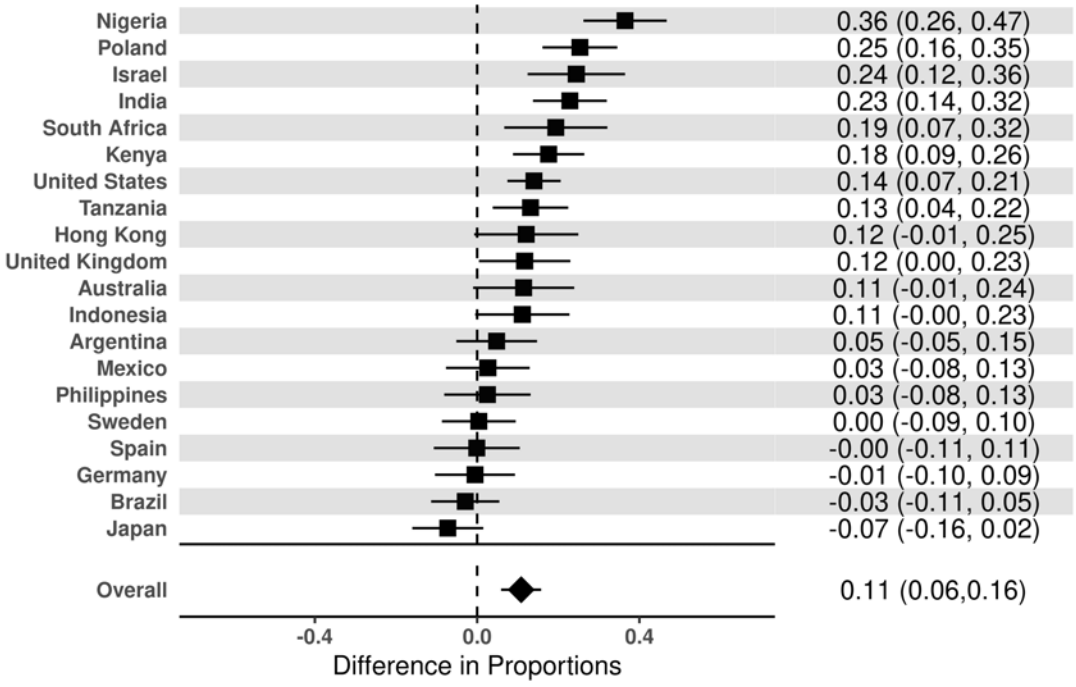

τ=0.100; Q(df=19)=97.35, p<.001;Q-profile 95% CI [0.066, 0.147]; I^2=79.92;
Excluded countries: Egypt, Turkiye

Figure S71. Forest plot for `Marital status`-`(Ref: Separated) Divorced`

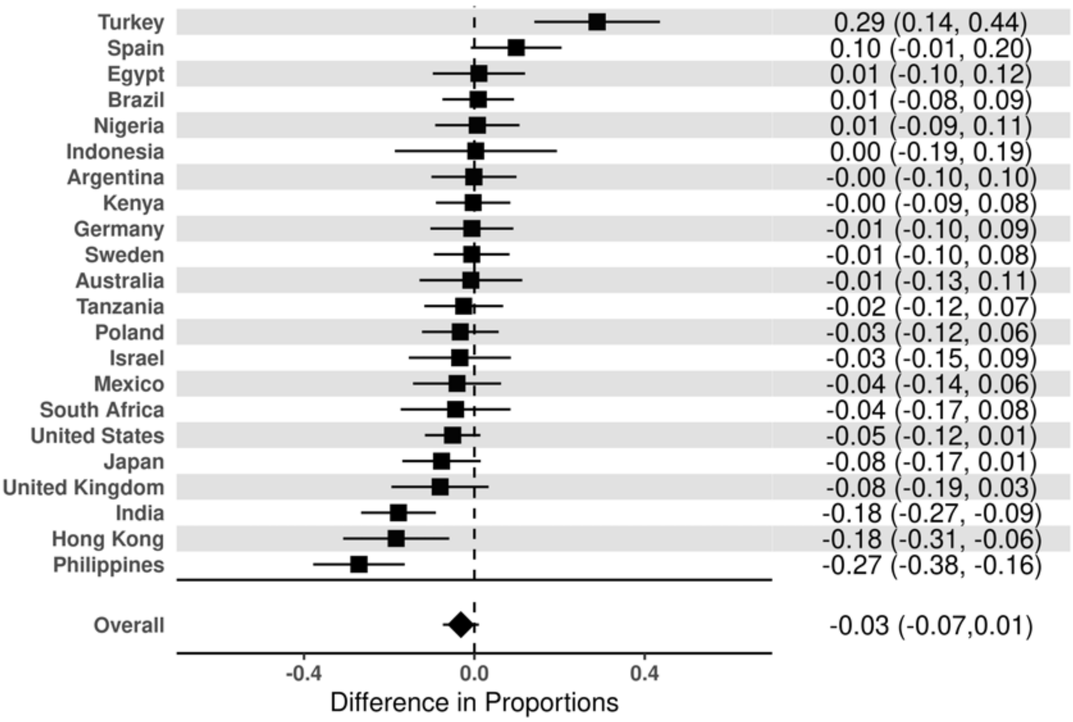

τ=0.085; Q(df=21)=64.94, p<.001;Q-profile 95% CI [0.043, 0.123]; I^2=73.39;

Figure S72. Forest plot for `Marital status`-`(Ref: Separated) Widowed`

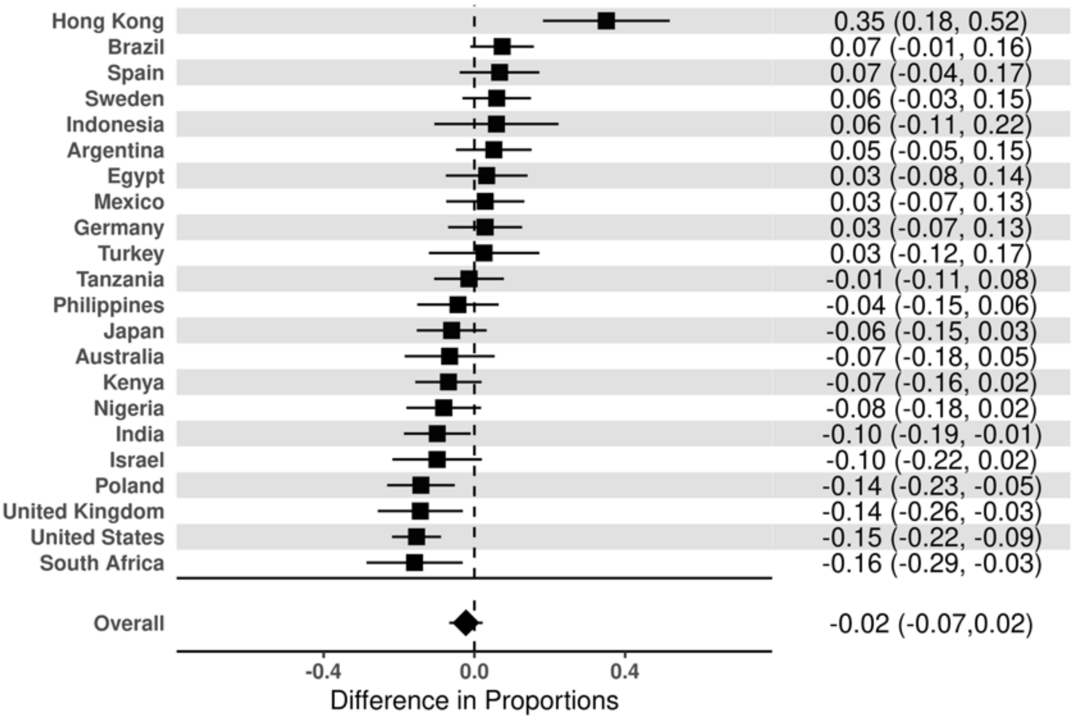

τ=0.091; Q(df=21)=74.15, p<.001;Q-profile 95% CI [0.047, 0.128]; I^2=75.50;

Figure S73. Forest plot for `Marital status`-`(Ref: Separated) Single, never married`

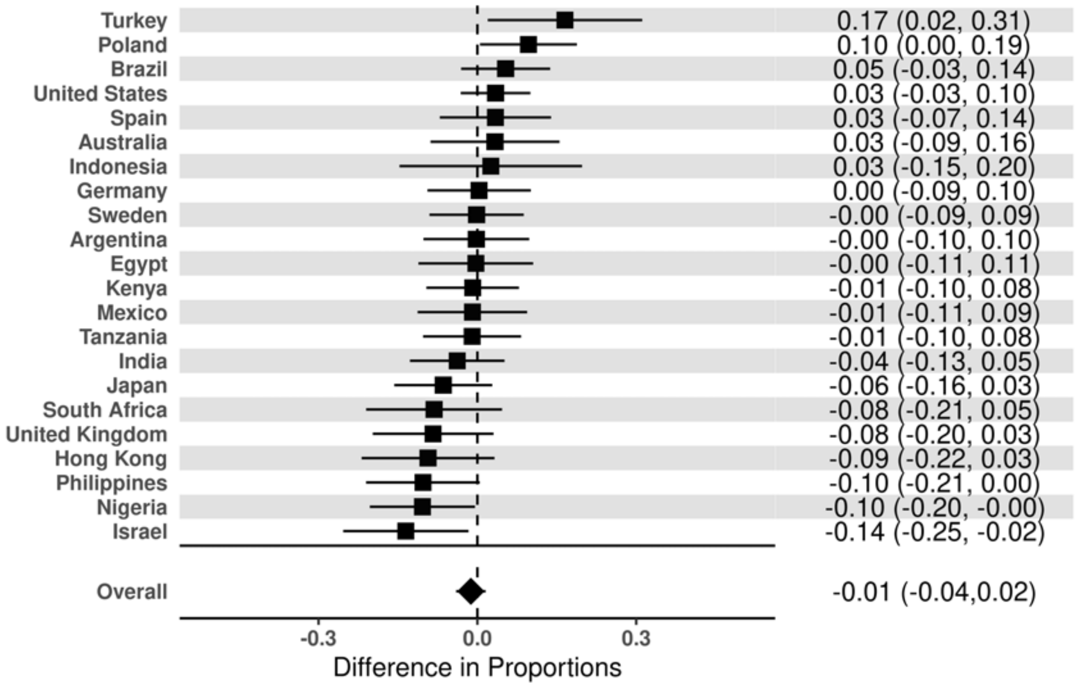

τ=0.041; Q(df=21)=32.94, p0.047;Q-profile 95% CI [0.000, 0.071]; I^2=39.11;

Figure S74. Forest plot for `Marital status`-`(Ref: Separated) Domestic partner`

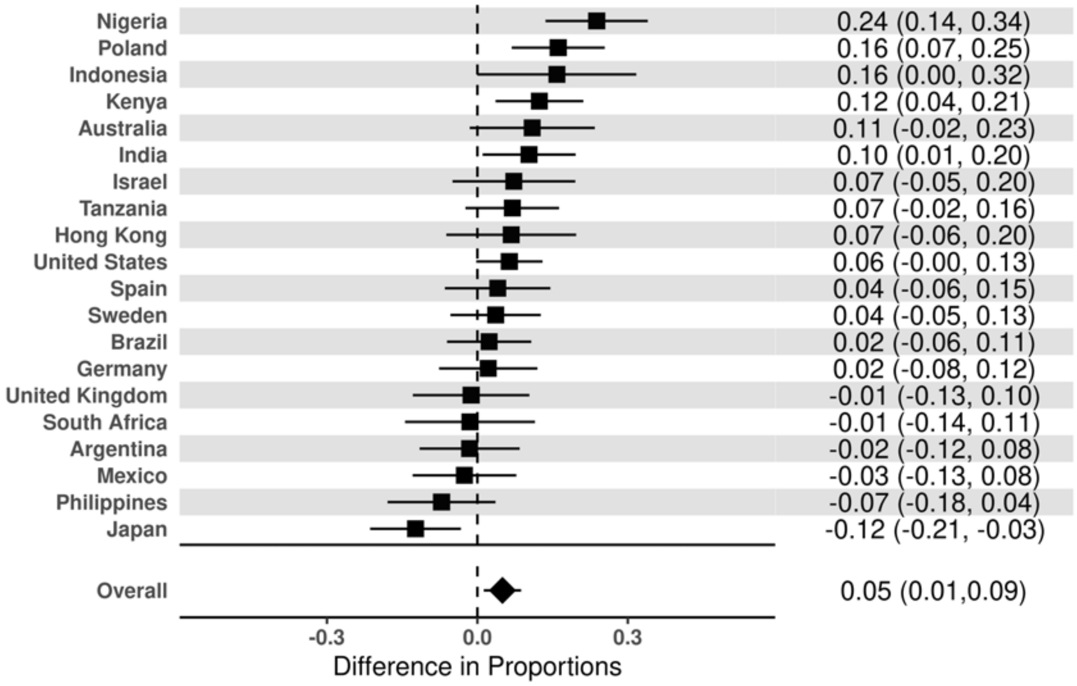

τ=0.067; Q(df=19)=51.38, p<.001;Q-profile 95% CI [0.035, 0.105]; I^2=63.18;
Excluded countries: Egypt, Turkiye

Figure S75. Forest plot for `Marital status`-`(Ref: Divorced) Widowed`

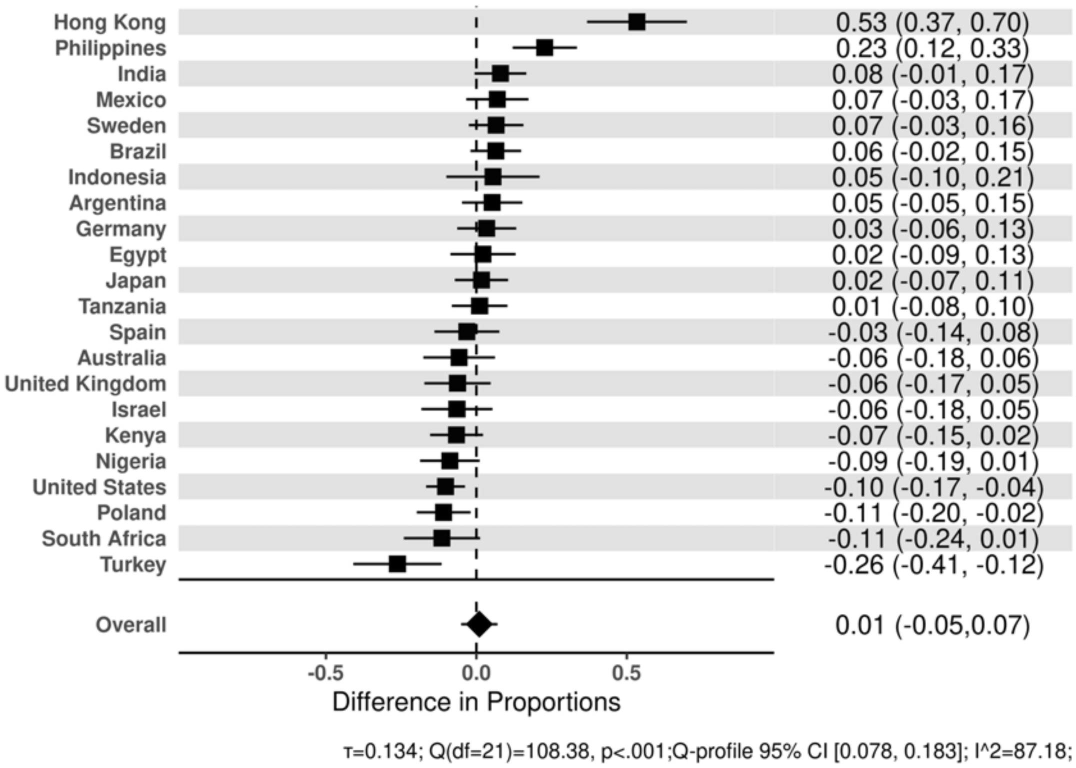

Figure S76. Forest plot for `Marital status`-`(Ref: Divorced) Single, never married`

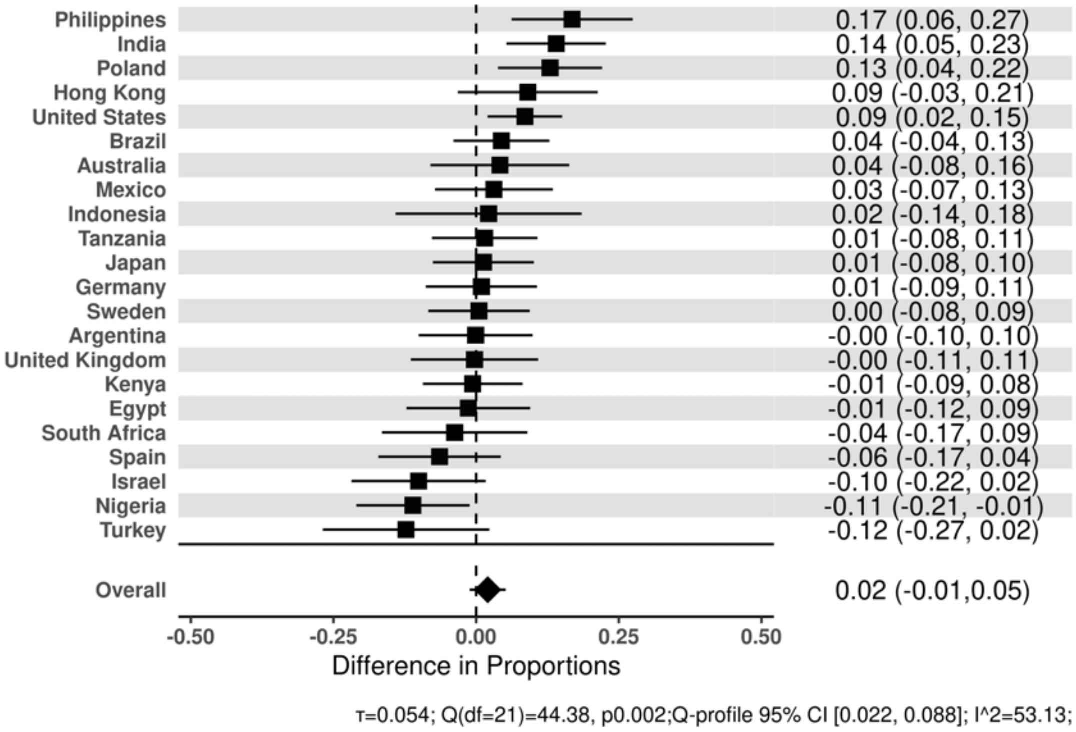

Figure S77. Forest plot for `Marital status`-`(Ref: Divorced) Domestic partner`

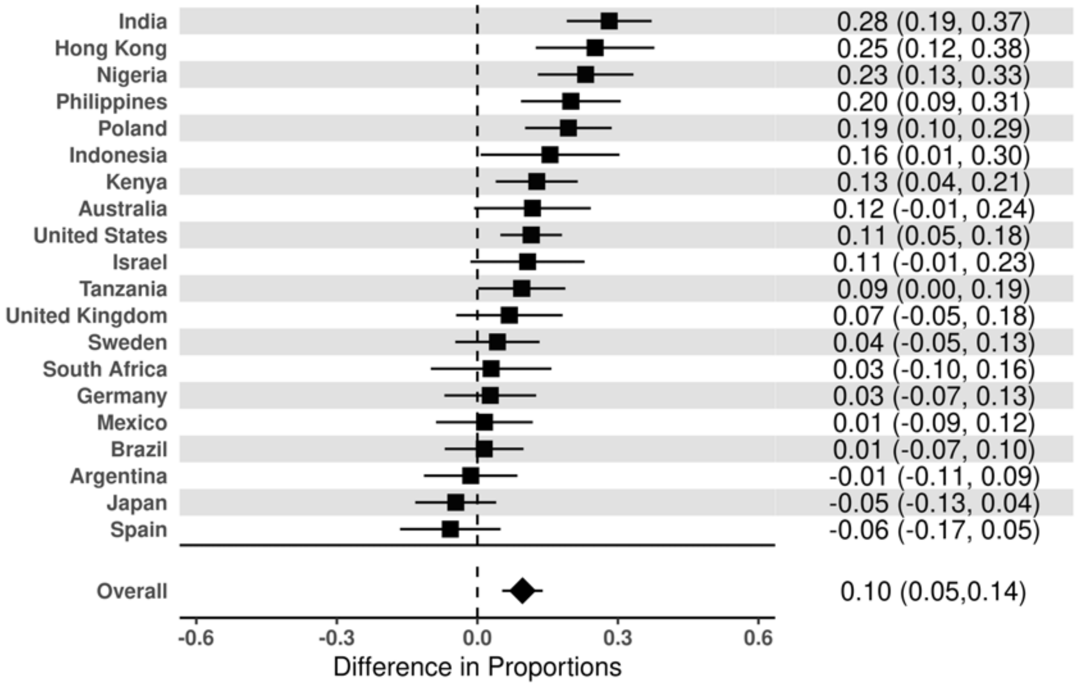

τ=0.084; Q(df=19)=71.58, p<.001;Q-profile 95% CI [0.051, 0.126]; I^2=73.31;
Excluded countries: Egypt, Turkiye

Figure S78. Forest plot for `Marital status`-`(Ref: Widowed) Single, never married`

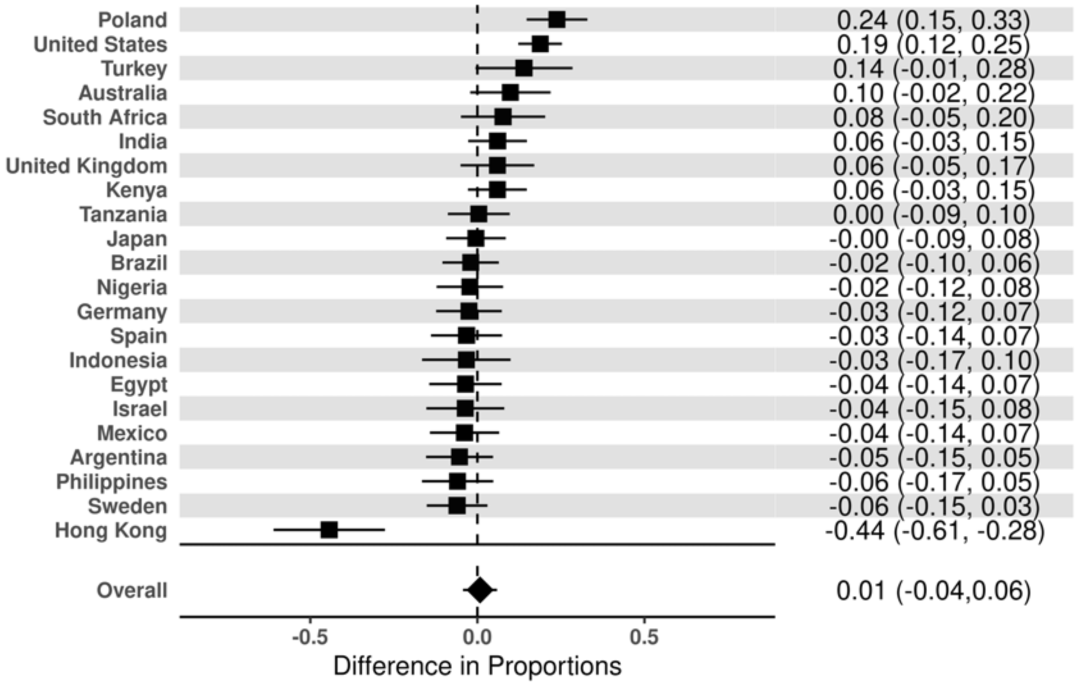

τ=0.109; Q(df=21)=98.75, p<.001;Q-profile 95% CI [0.063, 0.152]; I^2=81.99;

Figure S79. Forest plot for `Marital status`-`(Ref: Widowed) Domestic partner`

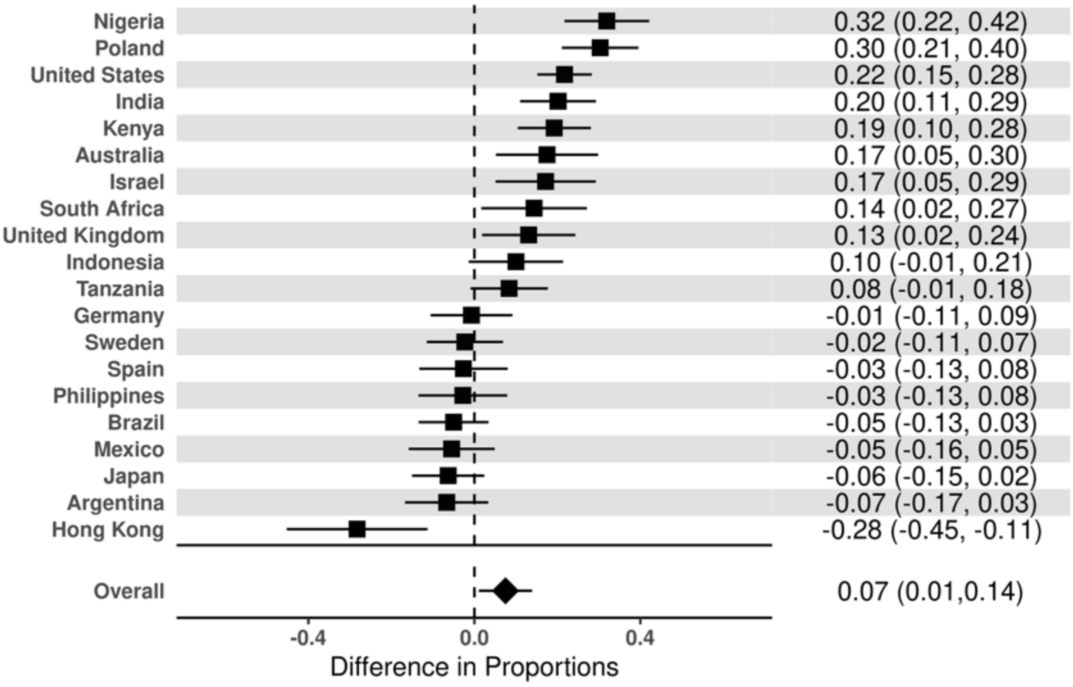

τ=0.137; Q(df=19)=146.74, p<.001;Q-profile 95% CI [0.091, 0.194]; I^2=88.00;
Excluded countries: Egypt, Turkiye

Figure S80. Forest plot for `Marital status`-`(Ref: Single, never married) Domestic partner`

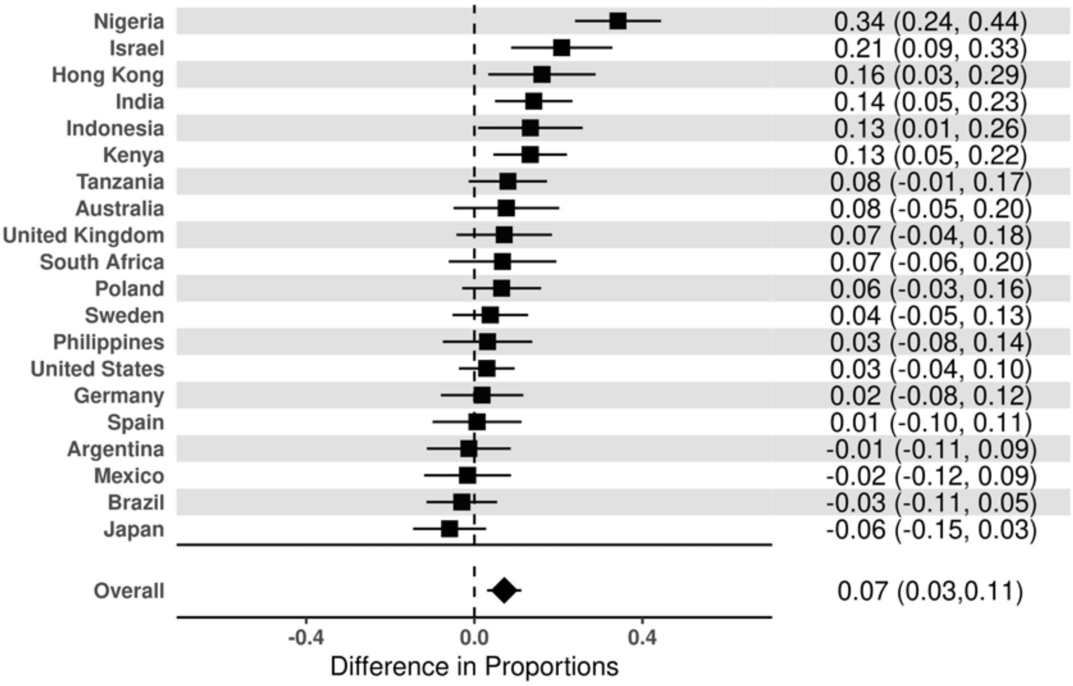

τ=0.077; Q(df=19)=63.22, p<.001;Q-profile 95% CI [0.045, 0.118]; I^2=70.28;
Excluded countries: Egypt, Turkiye

Figure S81. Forest plot for `Employment status`-`(Ref: Employed for an employer) Self-employed`

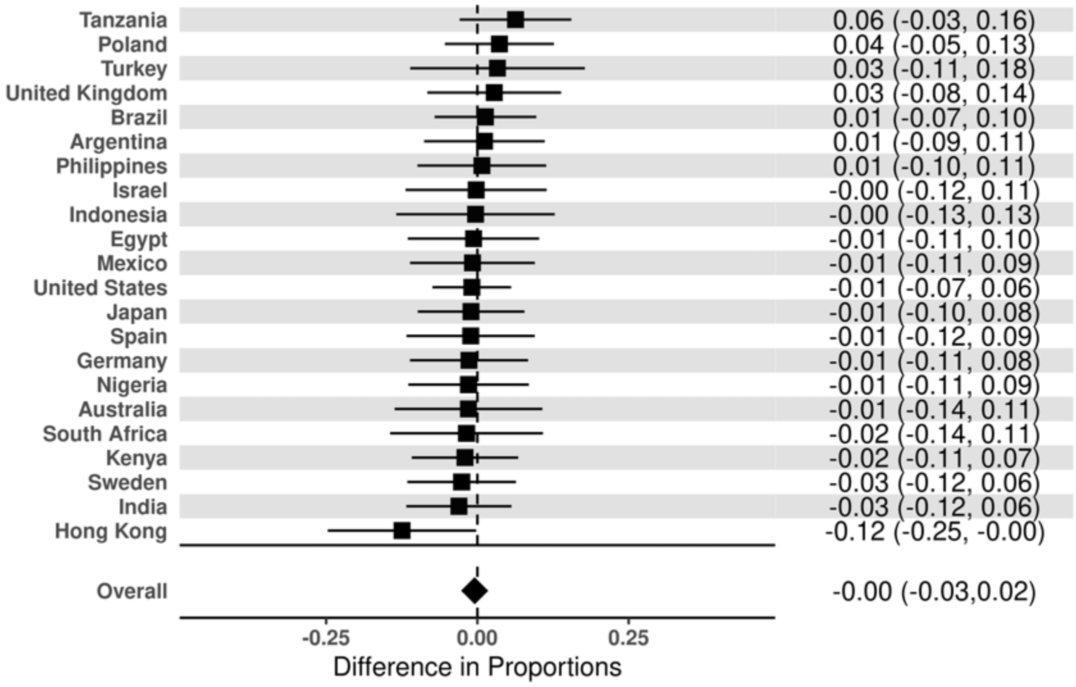

τ=0.000; Q(df=21)=8.34, p0.994;Q-profile 95% CI [0.000, 0.028]; I^2=0.00;

Figure S82. Forest plot for `Employment status`-`(Ref: Employed for an employer) Retired`

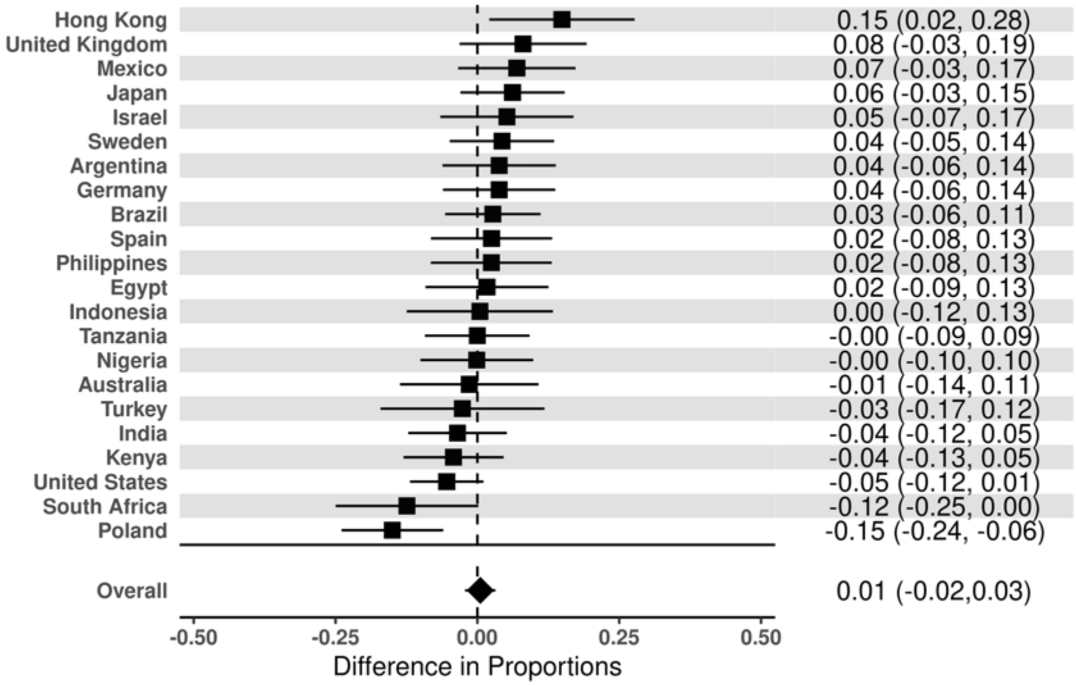

τ=0.038; Q(df=21)=33.13, p0.045;Q-profile 95% CI [0.000, 0.071]; I^2=35.41;

Figure S83. Forest plot for `Employment status`-`(Ref: Employed for an employer) Student`

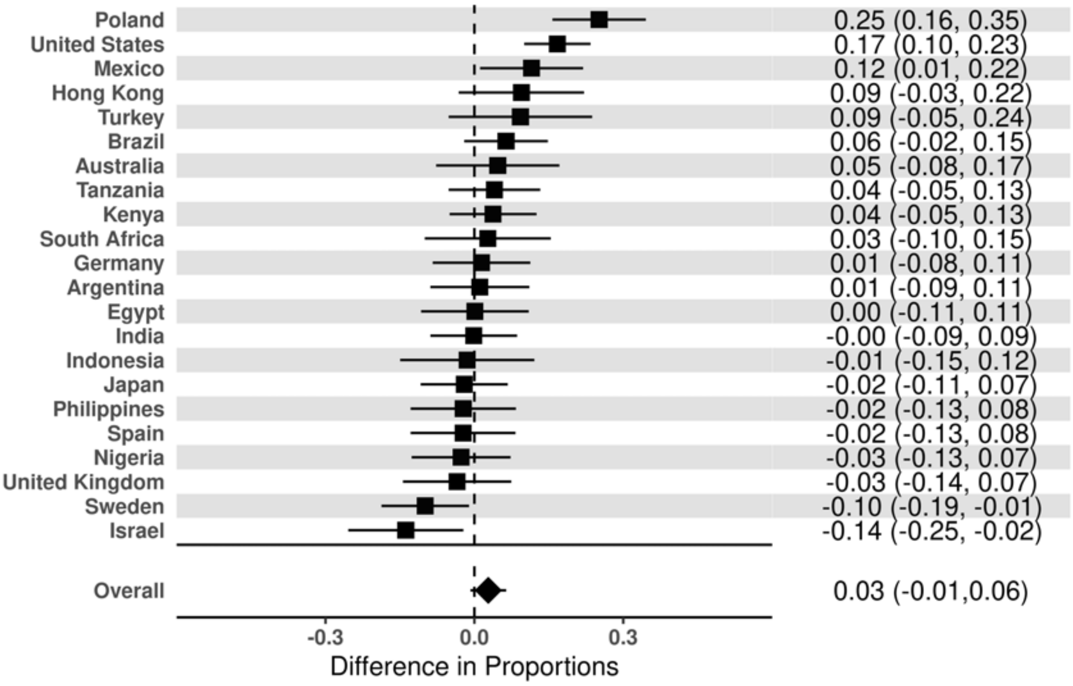

τ=0.068; Q(df=21)=65.88, p<.001;Q-profile 95% CI [0.043, 0.106]; I^2=64.27;

Figure S84. Forest plot for `Employment status`-`(Ref: Employed for an employer) Homemaker`

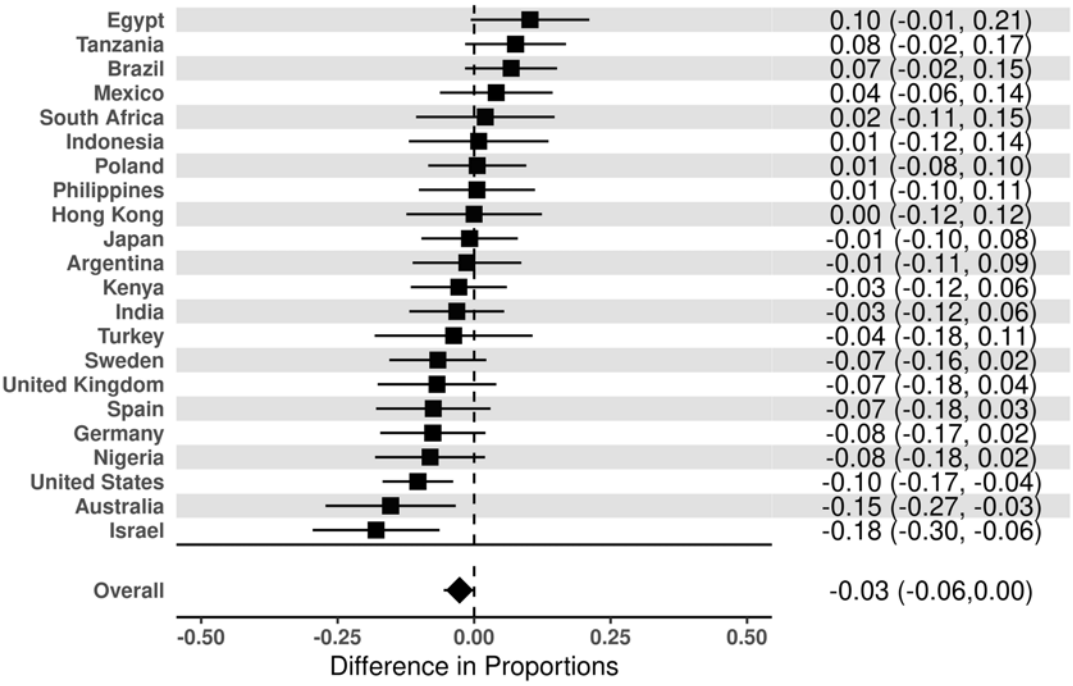

τ=0.046; Q(df=21)=39.22, p0.009;Q-profile 95% CI [0.012, 0.079]; I^2=45.70;

Figure S85. Forest plot for `Employment status`-`(Ref: Employed for an employer) Unemployed and looking for a job`

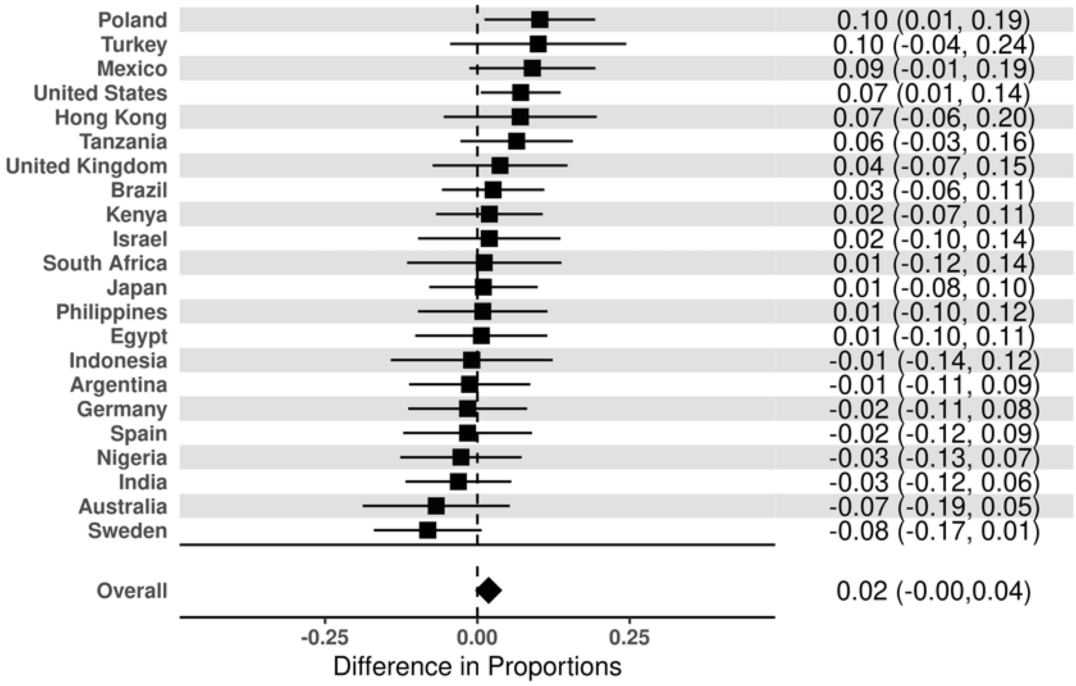

τ=0.000; Q(df=21)=21.02, p0.458;Q-profile 95% CI [0.000, 0.050]; I^2=0.00;

Figure S86. Forest plot for `Employment status`-`(Ref: Employed for an employer) None of these/other`

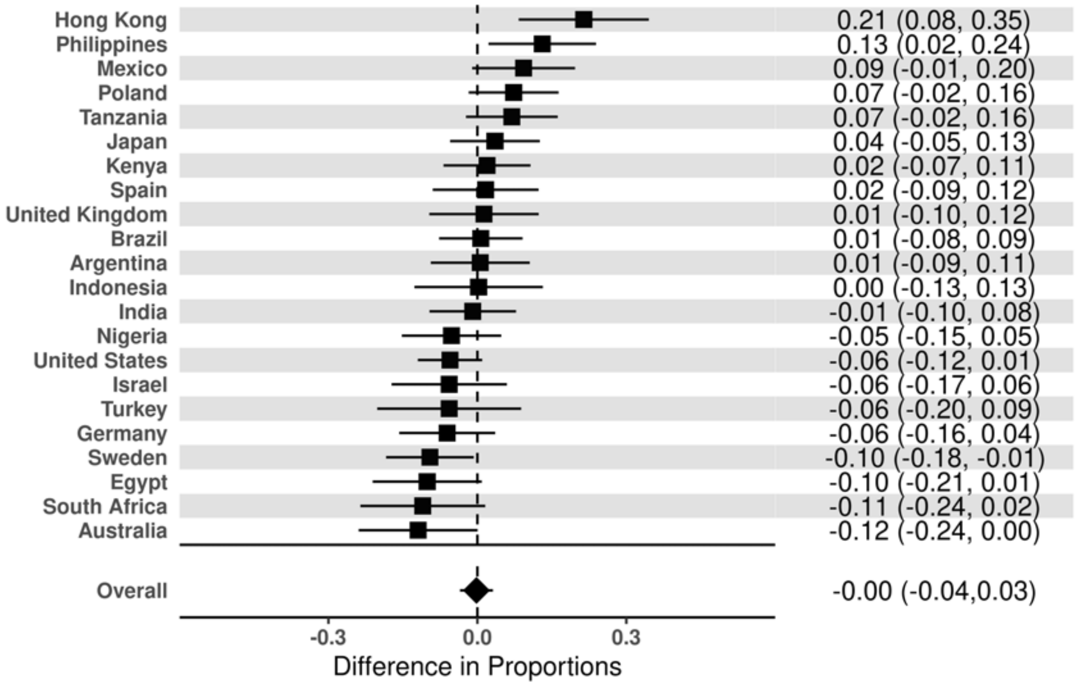

τ=0.060; Q(df=21)=45.88, p0.001;Q-profile 95% CI [0.019, 0.092]; I^2=58.13;

Figure S87. Forest plot for `Employment status`-`(Ref: Self-employed) Retired`

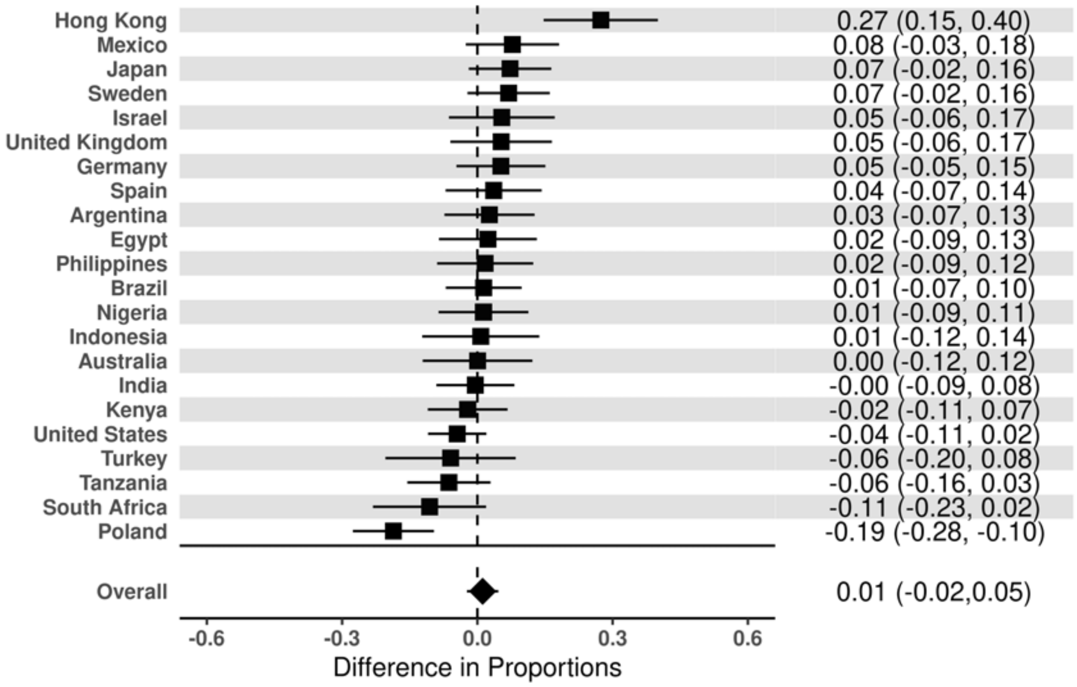

τ=0.065; Q(df=21)=52.04, p<.001;Q-profile 95% CI [0.030, 0.099]; I^2=61.77;

Figure S88. Forest plot for `Employment status`-`(Ref: Self-employed) Student`

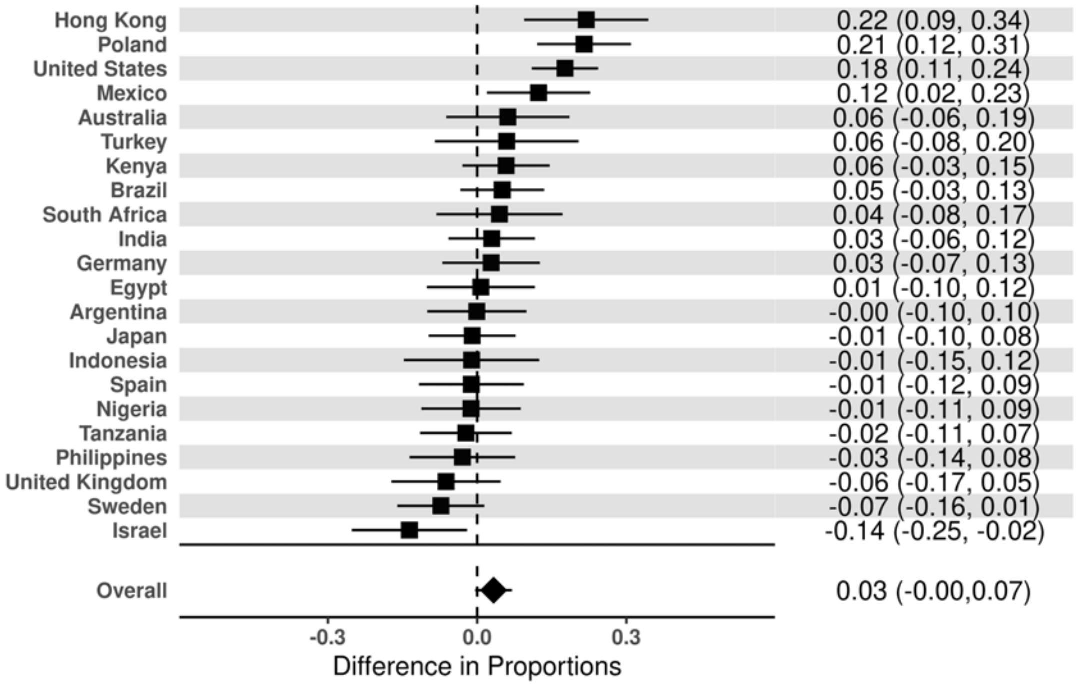

τ=0.071; Q(df=21)=67.00, p<.001;Q-profile 95% CI [0.043, 0.109]; I^2=66.35;

Figure S89. Forest plot for `Employment status`-`(Ref: Self-employed) Homemaker`

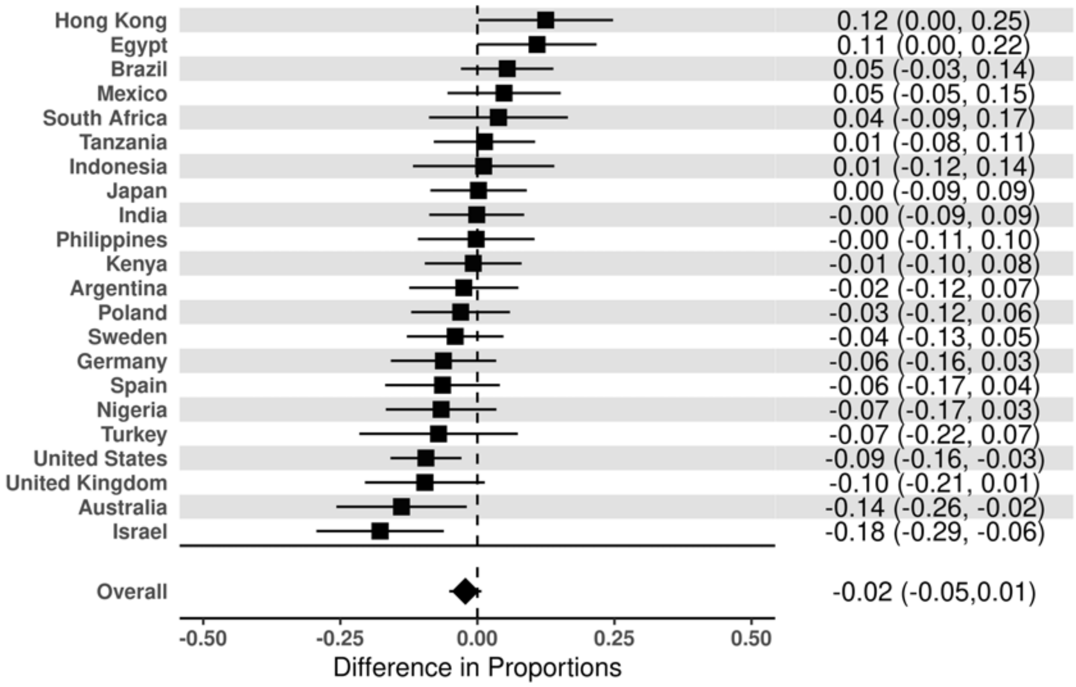

τ=0.048; Q(df=21)=38.09, p0.013;Q-profile 95% CI [0.000, 0.078]; I^2=47.15;

Figure S90. Forest plot for `Employment status`-`(Ref: Self-employed) Unemployed and looking for a job`

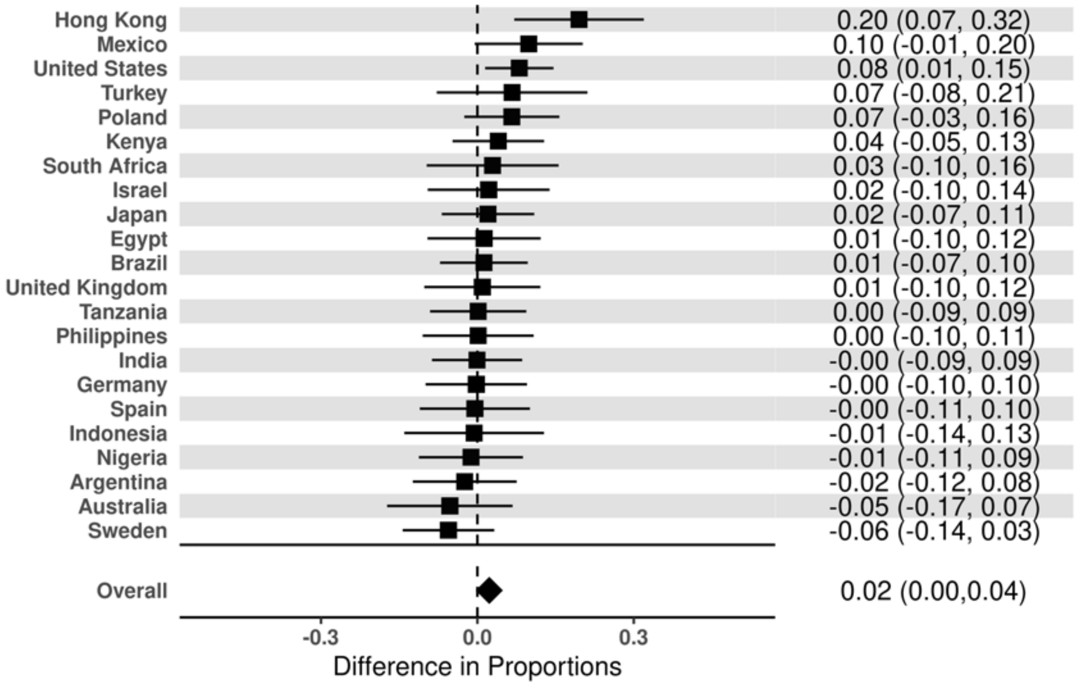

τ=0.000; Q(df=21)=21.17, p0.448;Q-profile 95% CI [0.000, 0.047]; I^2=0.00;

Figure S91. Forest plot for `Employment status`-`(Ref: Self-employed) None of these/other`

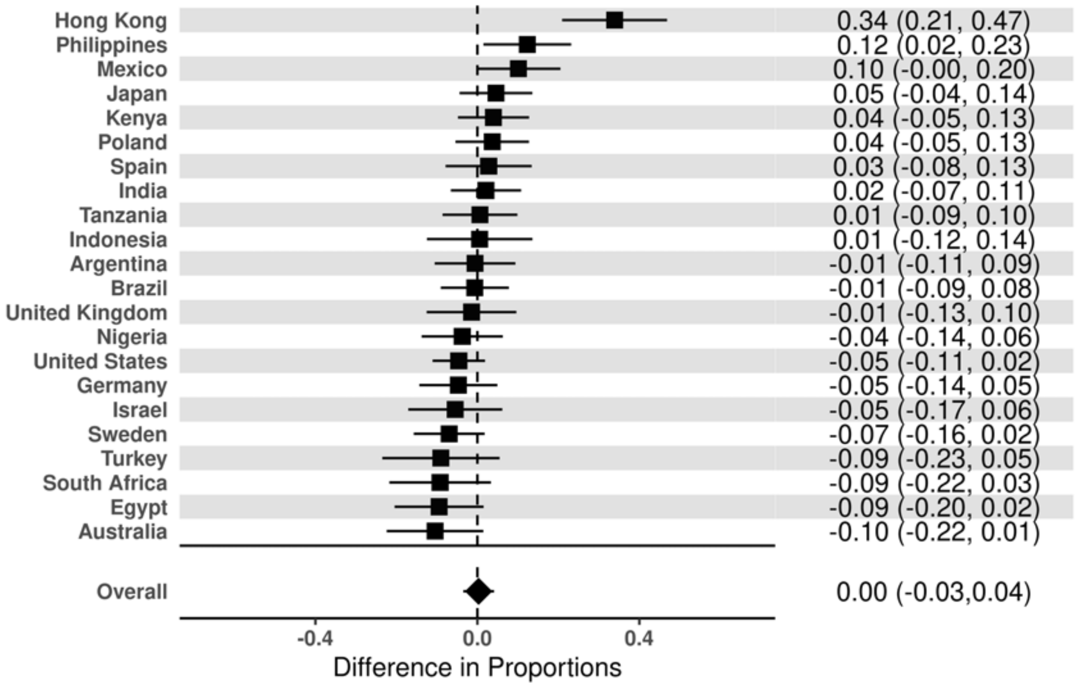

τ=0.074; Q(df=21)=53.93, p<.001;Q-profile 95% CI [0.028, 0.108]; I^2=68.19;

Figure S92. Forest plot for `Employment status`-`(Ref: Retired) Student`

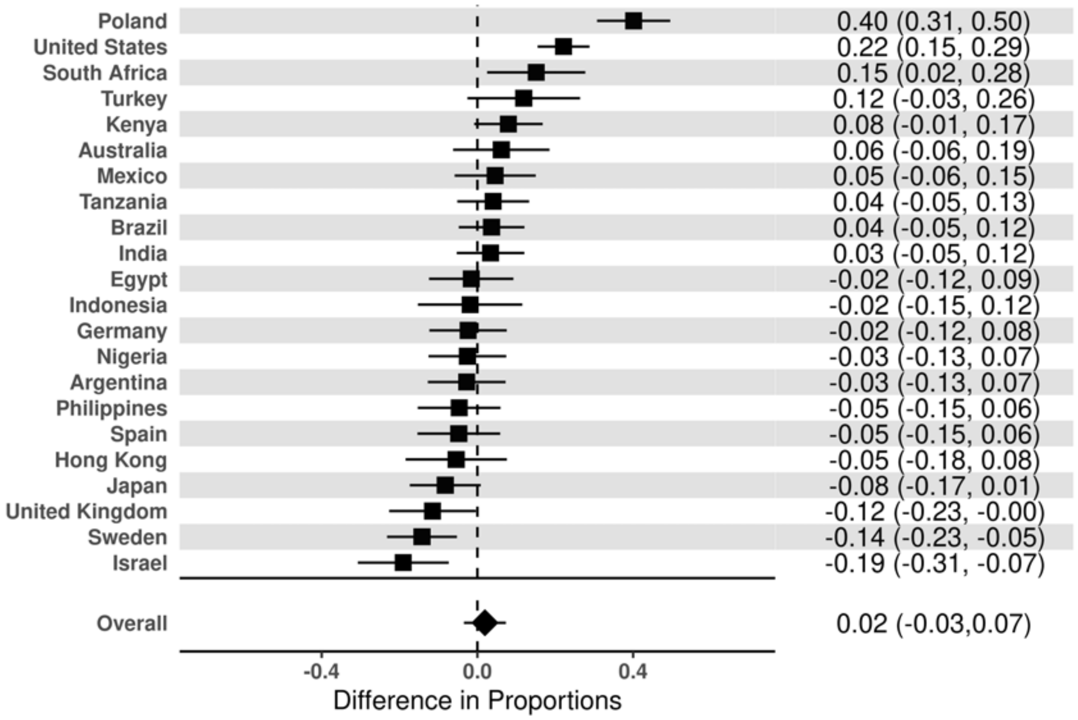

τ=0.117; Q(df=21)=148.97, p<.001;Q-profile 95% CI [0.082, 0.168]; I^2=84.02;

Figure S93. Forest plot for `Employment status`-`(Ref: Retired) Homemaker`

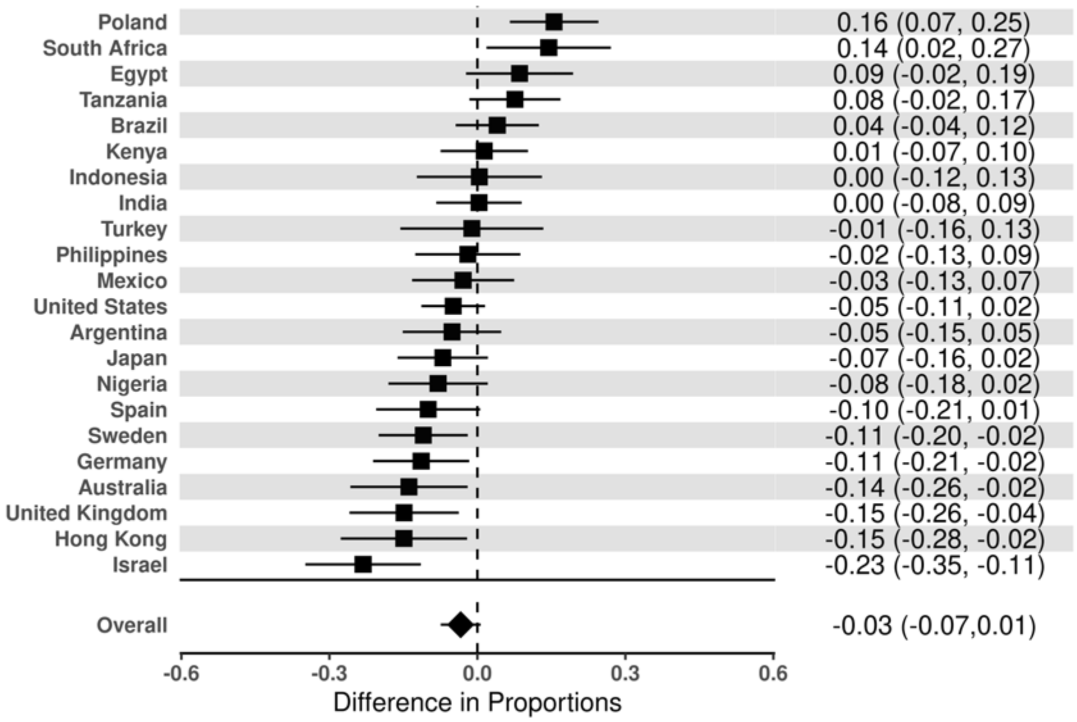

τ=0.081; Q(df=21)=69.84, p<.001;Q-profile 95% CI [0.047, 0.120]; I^2=71.89;

Figure S94. Forest plot for `Employment status`-`(Ref: Retired) Unemployed and looking for a job`

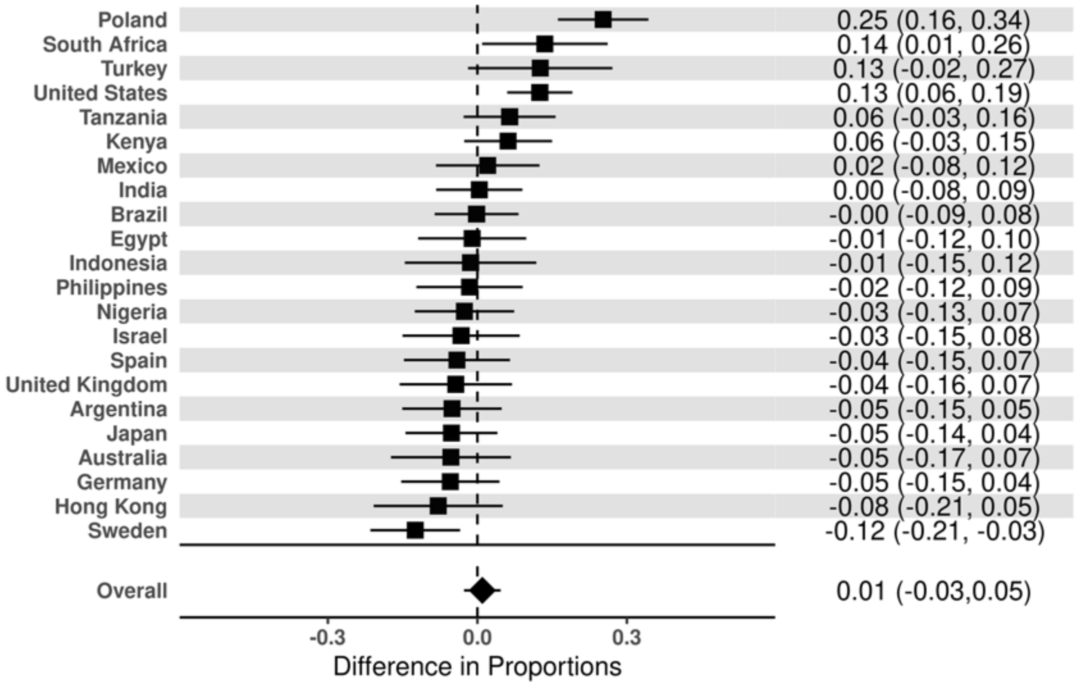

τ=0.070; Q(df=21)=67.53, p<.001;Q-profile 95% CI [0.044, 0.109]; I^2=65.57;

Figure S95. Forest plot for `Employment status`-`(Ref: Retired) None of these/other`

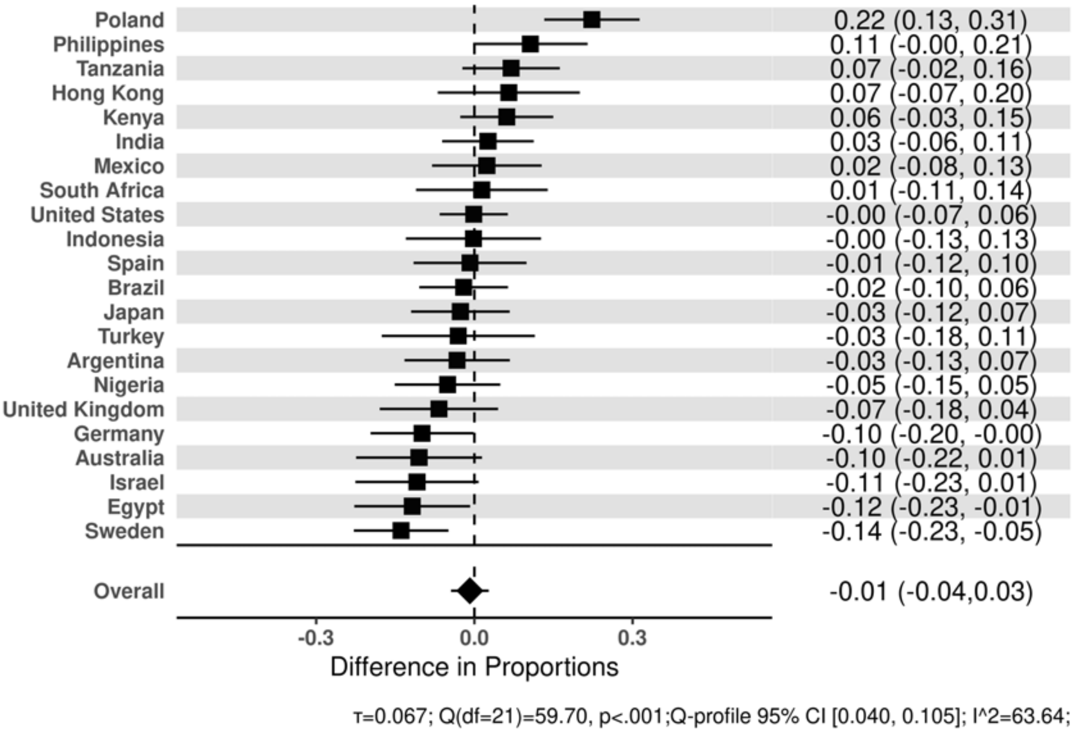

Figure S96. Forest plot for `Employment status`-`(Ref: Student) Homemaker`

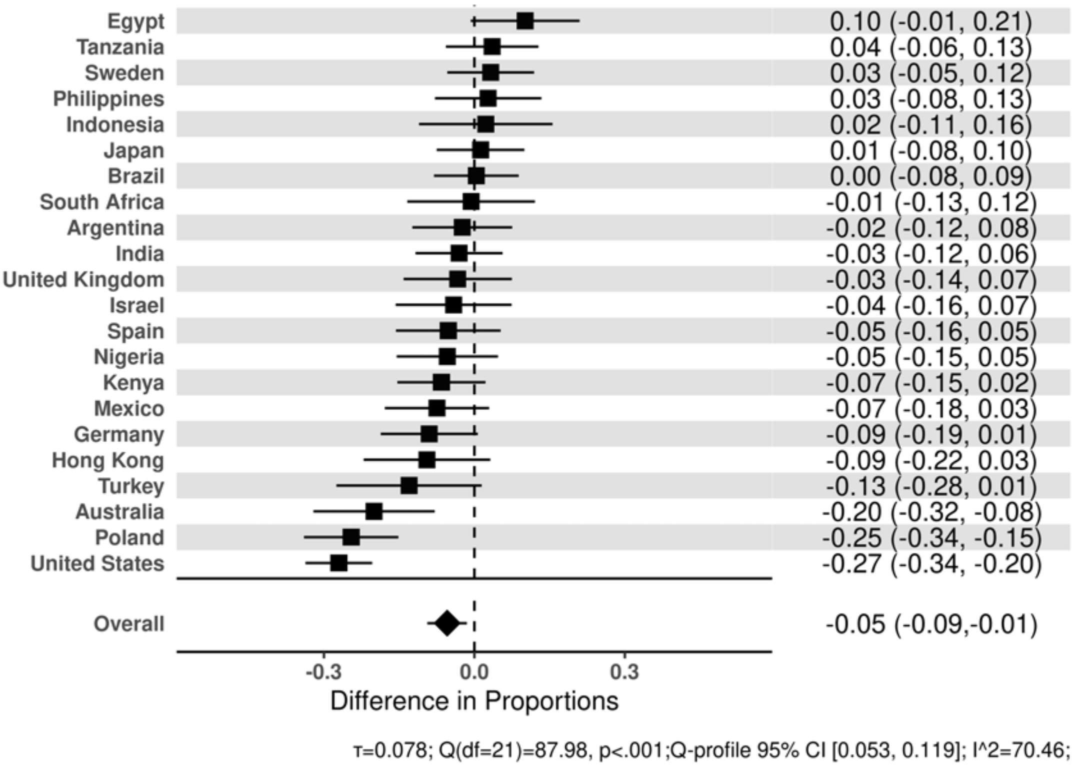

Figure S97. Forest plot for `Employment status`-`(Ref: Student) Unemployed and looking for a job`

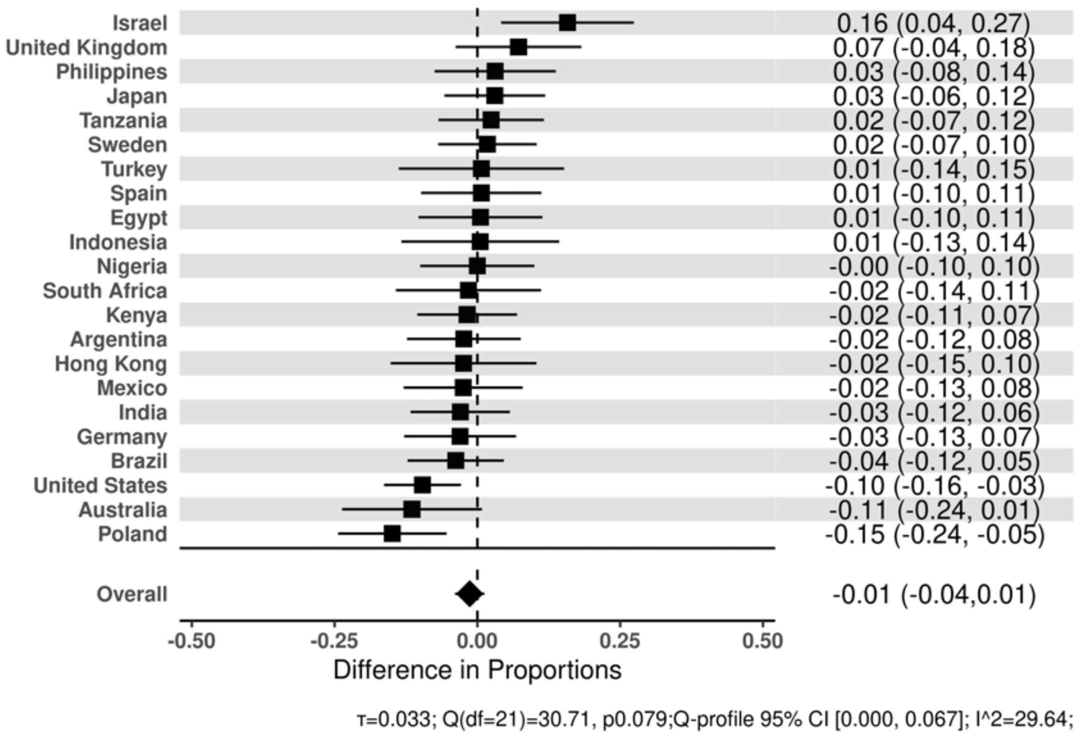

Figure S98. Forest plot for `Employment status`-`(Ref: Student) None of these/other`

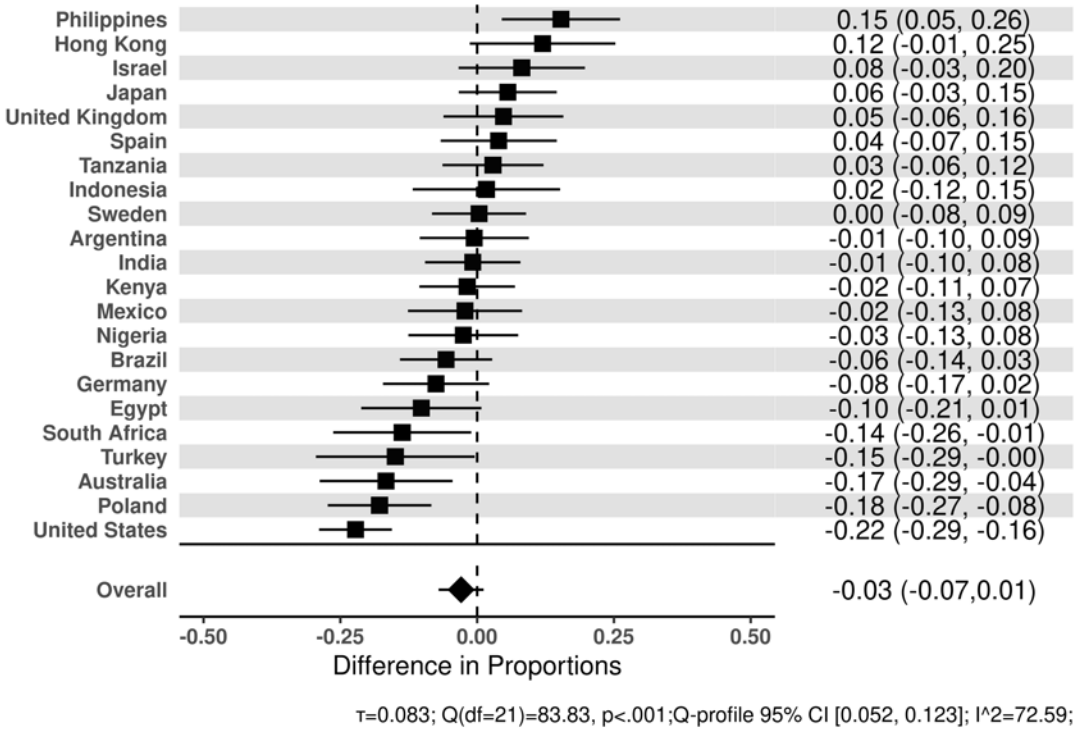

Figure S99. Forest plot for `Employment status`-`(Ref: Homemaker) Unemployed and looking for a job`

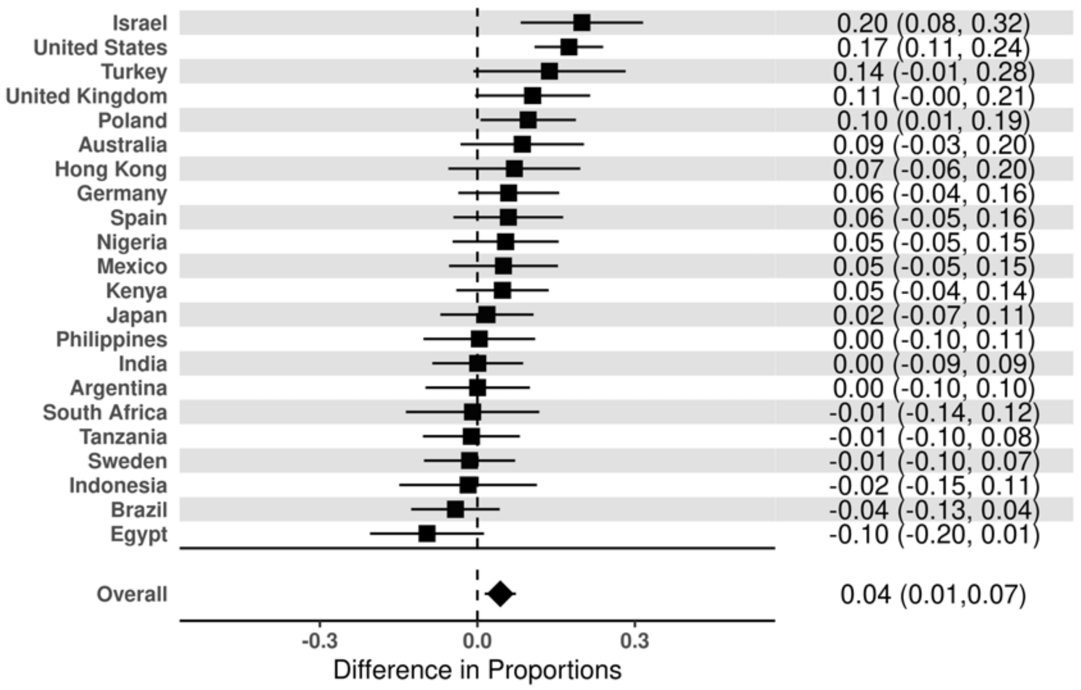

τ=0.049; Q(df=21)=44.61, p0.002;Q-profile 95% CI [0.024, 0.083]; I^2=48.13;

Figure S100. Forest plot for `Employment status`-`(Ref: Homemaker) None of these/other`

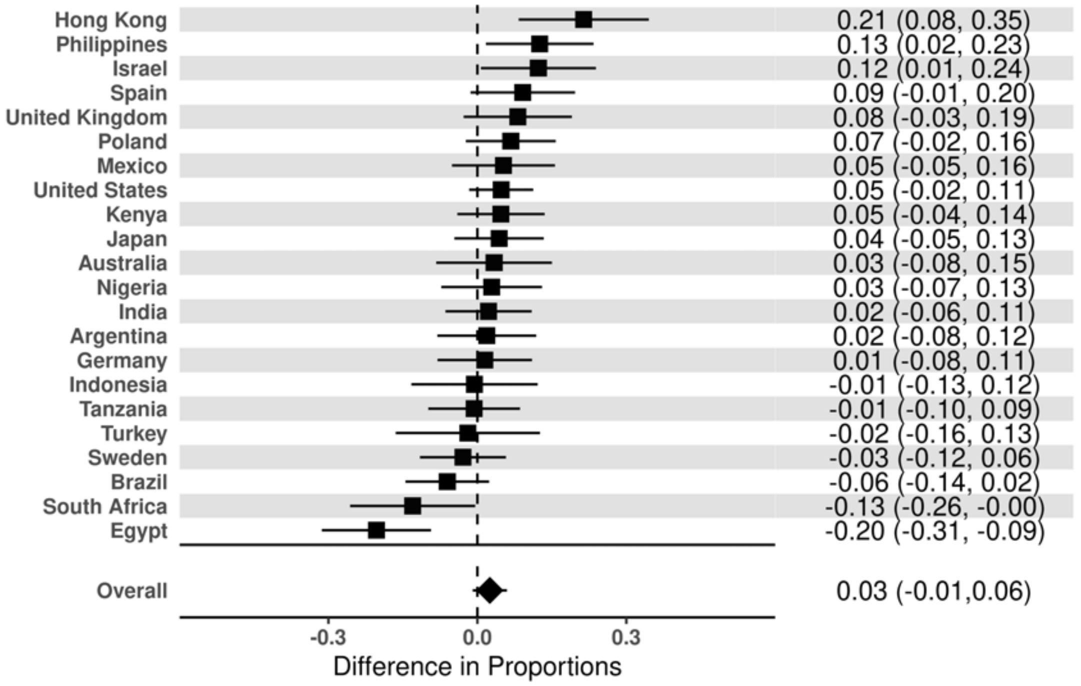

τ=0.064; Q(df=21)=47.61, p<.001;Q-profile 95% CI [0.019, 0.096]; I^2=61.60;

Figure S101. Forest plot for `Employment status`-`(Ref: Unemployed and looking for a job) None of these/other`

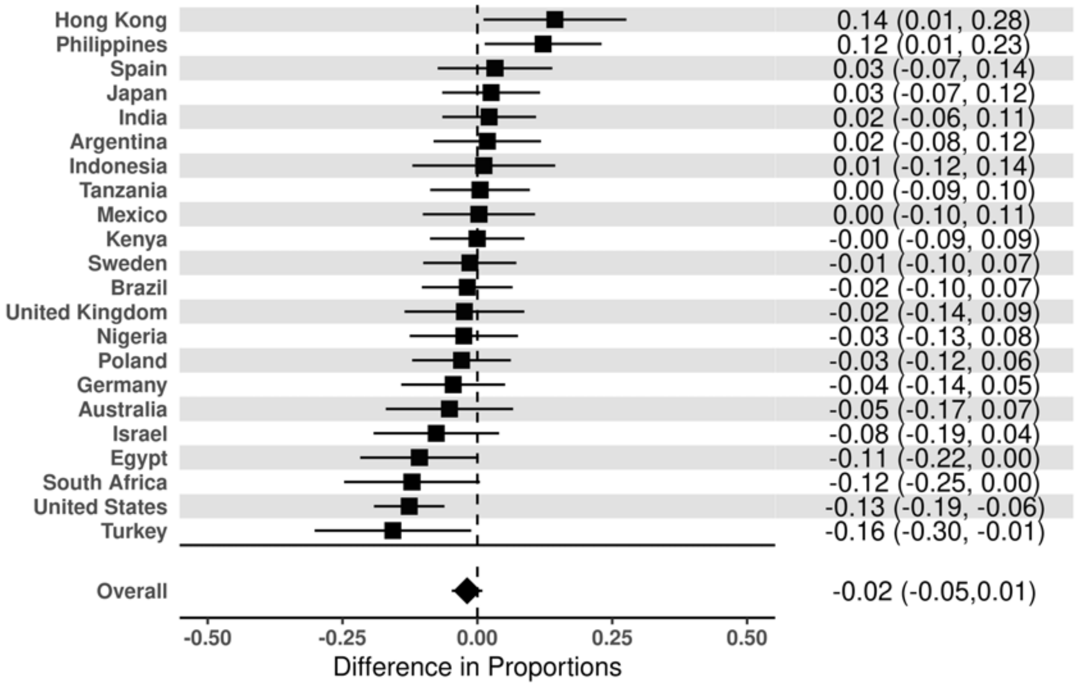

τ=0.044; Q(df=21)=36.68, p0.018;Q-profile 95% CI [0.000, 0.074]; I^2=42.67;

Figure S102. Forest plot for `Religious service attendance`-`(Ref: >1/week) 1/week`

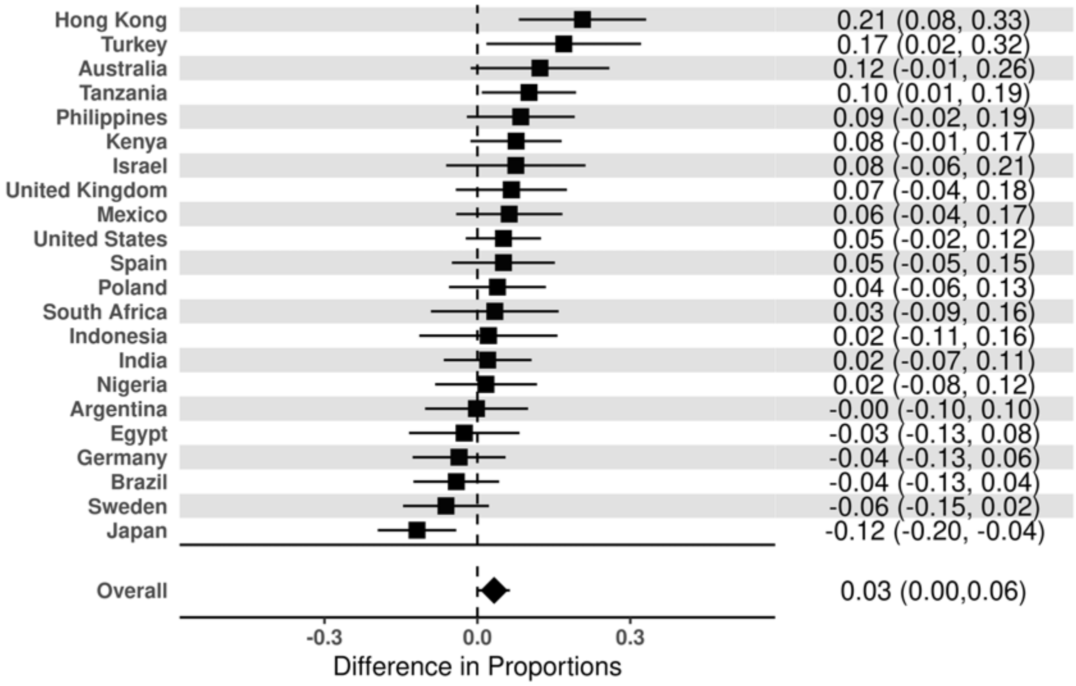

τ=0.051; Q(df=21)=43.72, p0.003;Q-profile 95% CI [0.022, 0.085]; I^2=50.50;

Figure S103. Forest plot for `Religious service attendance`-`(Ref: >1/week) 1-3/month`

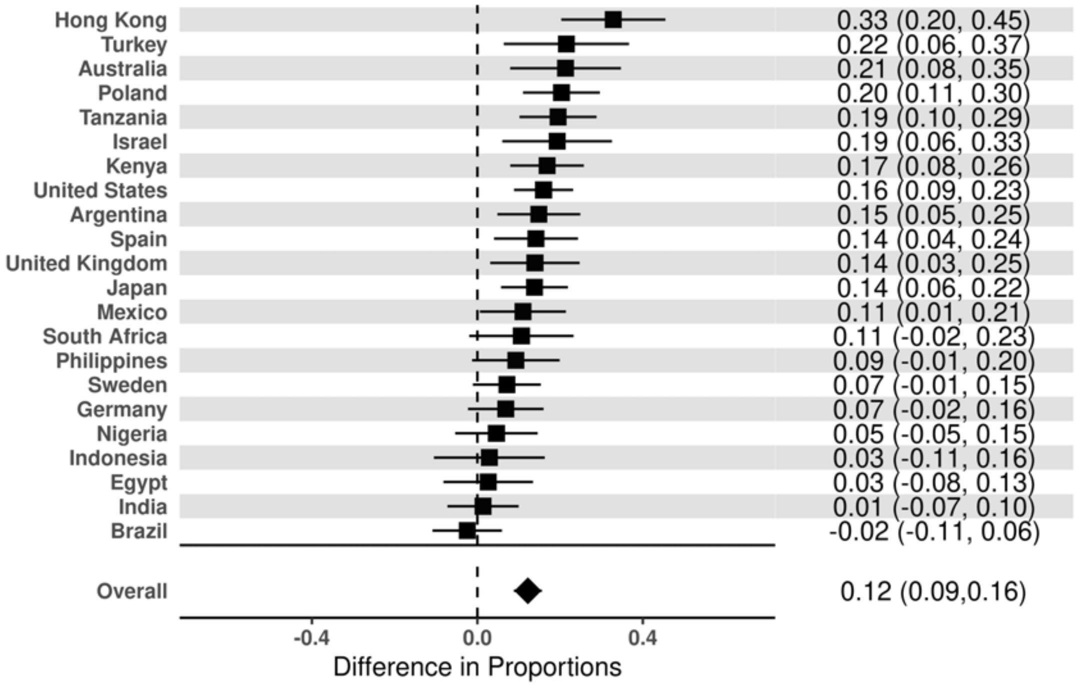

τ=0.062; Q(df=21)=50.64, p<.001;Q-profile 95% CI [0.028, 0.096]; I^2=59.56;

Figure S104. Forest plot for `Religious service attendance`-`(Ref: >1/week) A few times a year`

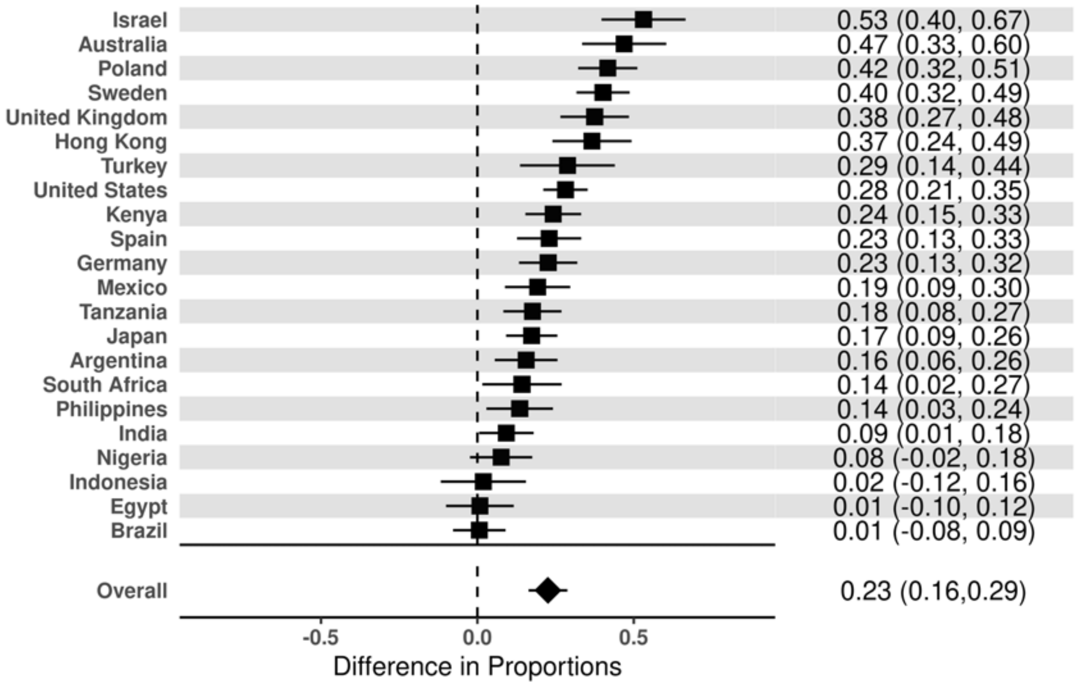

τ=0.138; Q(df=21)=156.91, p<.001;Q-profile 95% CI [0.095, 0.193]; I^2=88.00;

Figure S105. Forest plot for `Religious service attendance`-`(Ref: >1/week) Never`

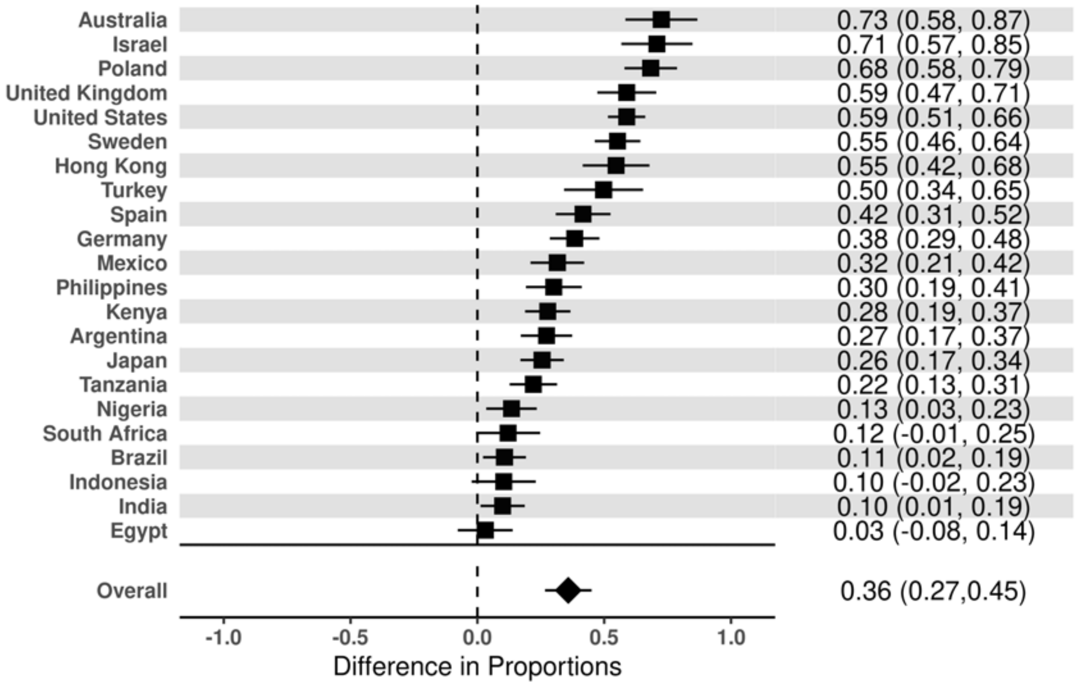

τ=0.213; Q(df=21)=341.54, p<.001;Q-profile 95% CI [0.153, 0.293]; I^2=94.27;

Figure S106. Forest plot for `Religious service attendance`-`(Ref: 1/week) 1-3/month`

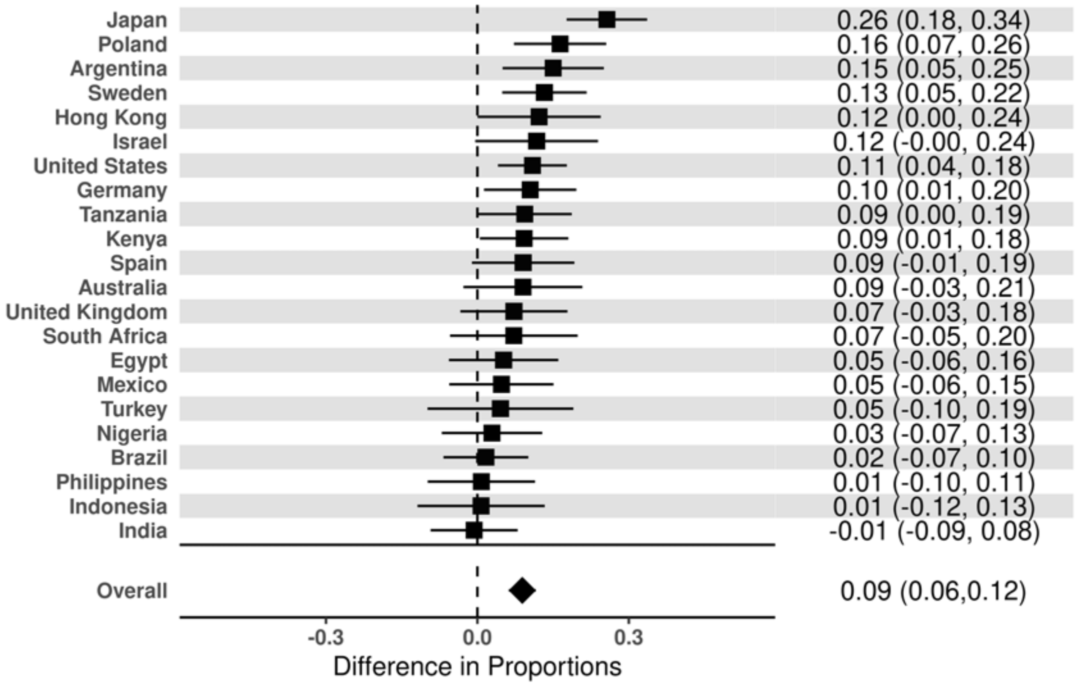

τ=0.039; Q(df=21)=37.26, p0.016;Q-profile 95% CI [0.014, 0.075]; I^2=38.09;

Figure S107. Forest plot for `Religious service attendance`-`(Ref: 1/week) A few times a year`

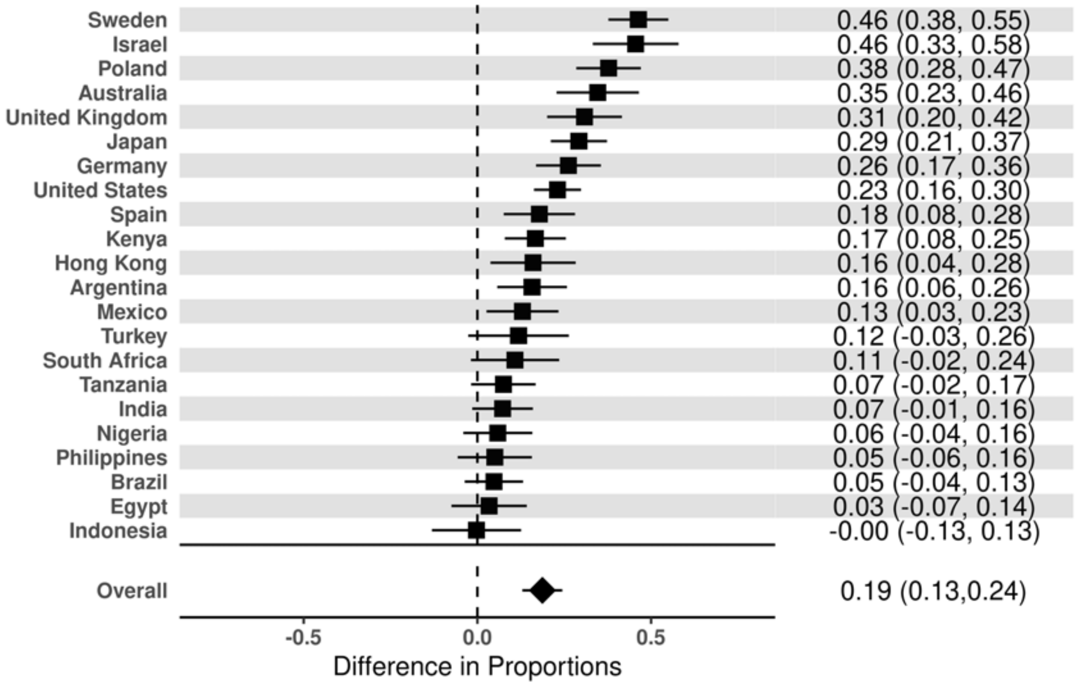

τ=0.127; Q(df=21)=151.66, p<.001;Q-profile 95% CI [0.088, 0.179]; I^2=86.46;

Figure S108. Forest plot for `Religious service attendance`-`(Ref: 1/week) Never`

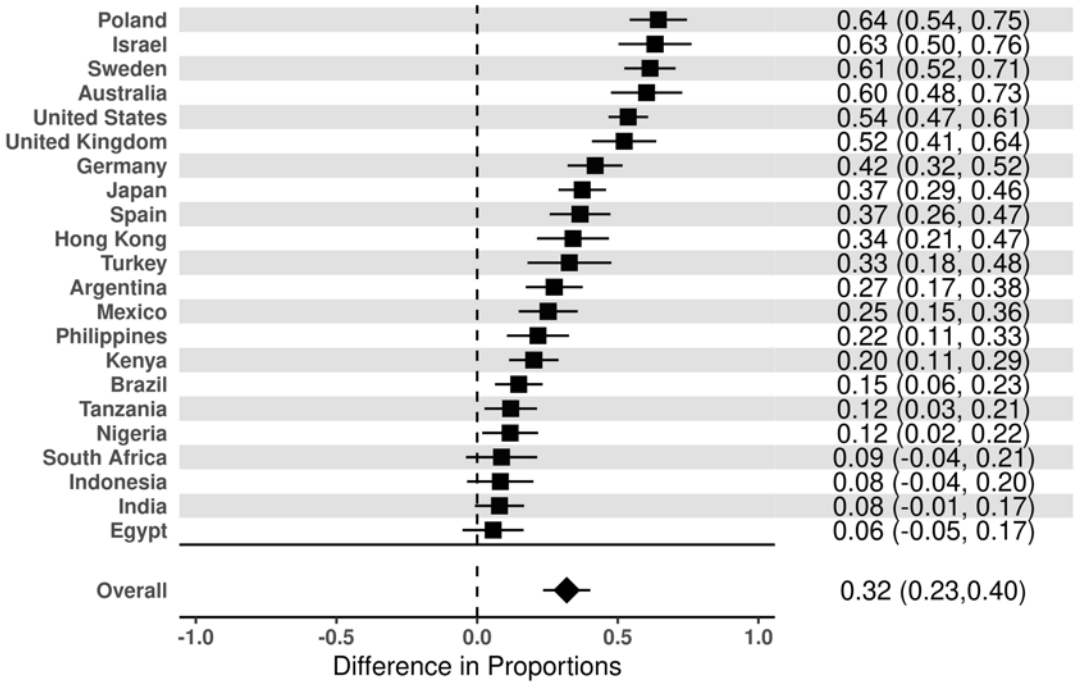

τ=0.194; Q(df=21)=317.85, p<.001;Q-profile 95% CI [0.140, 0.268]; I^2=93.38;

Figure S109. Forest plot for `Religious service attendance`-`(Ref: 1-3/month) A few times a year`

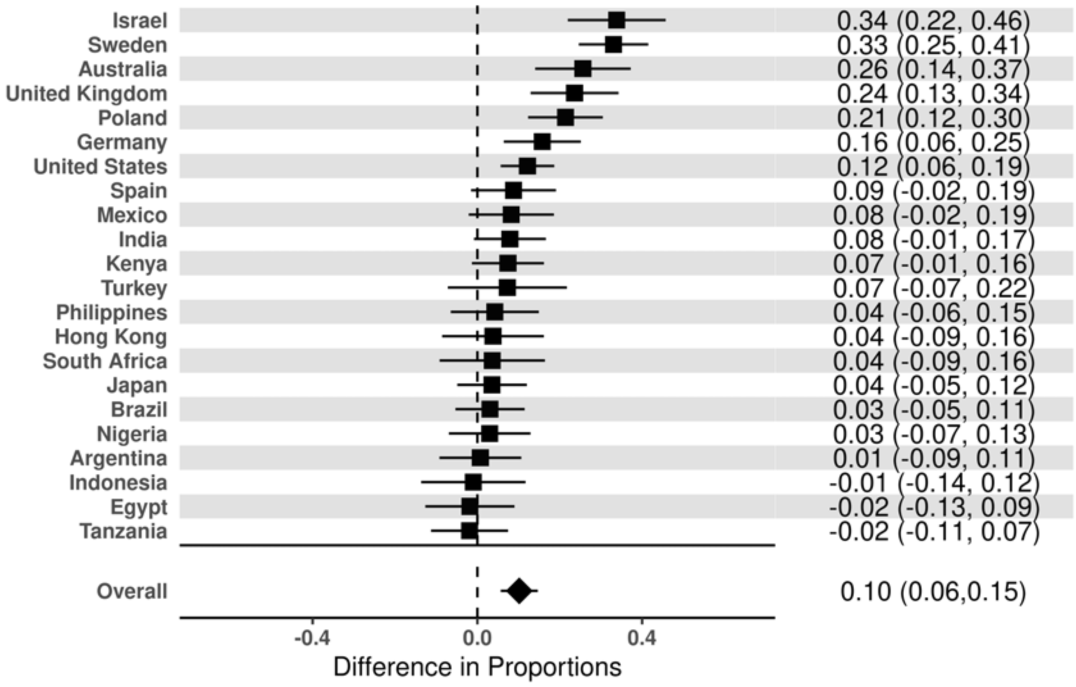

τ=0.094; Q(df=21)=92.89, p<.001;Q-profile 95% CI [0.061, 0.137]; I^2=78.04;

Figure S110. Forest plot for `Religious service attendance`-`(Ref: 1-3/month) Never`

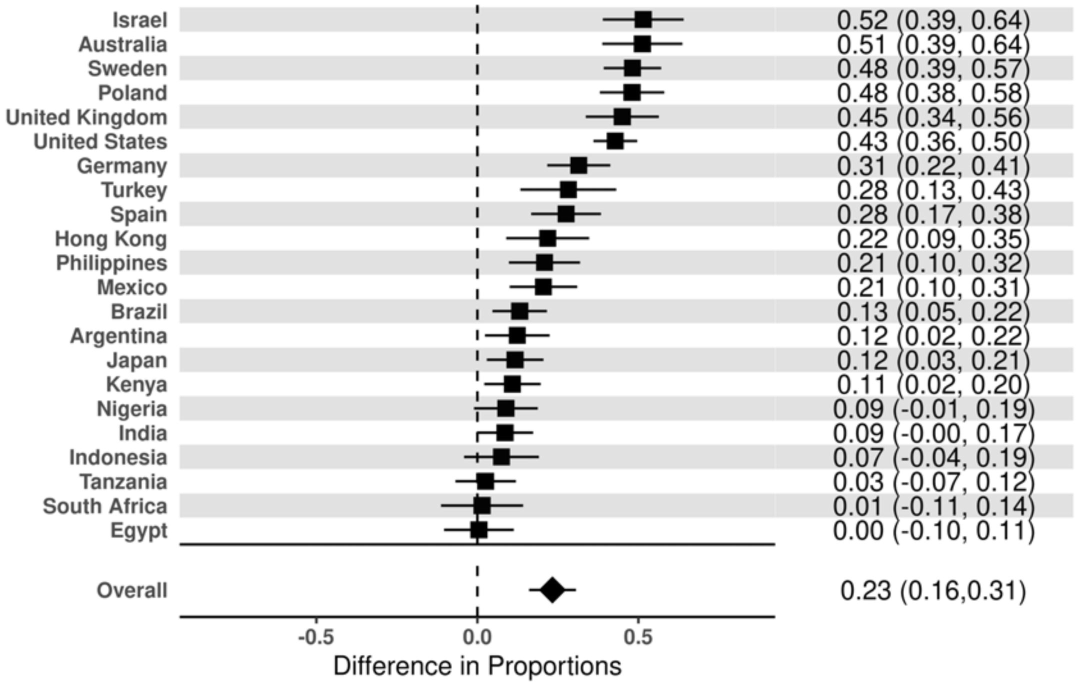

τ=0.166; Q(df=21)=239.75, p<.001;Q-profile 95% CI [0.118, 0.230]; I^2=91.22;

Figure S111. Forest plot for `Religious service attendance`-`(Ref: A few times a year) Never`

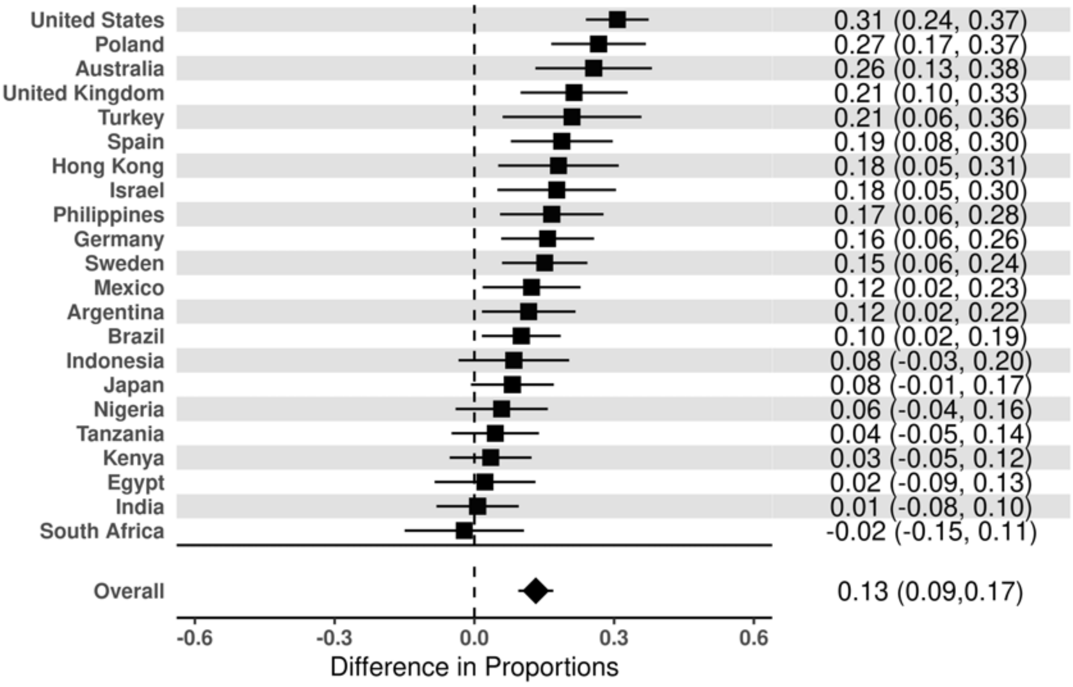

τ=0.072; Q(df=21)=71.87, p<.001;Q-profile 95% CI [0.046, 0.111]; I^2=66.08;

Figure S112. Forest plot for `Education`-`(Ref: Up to 8 years) 9-15 years`

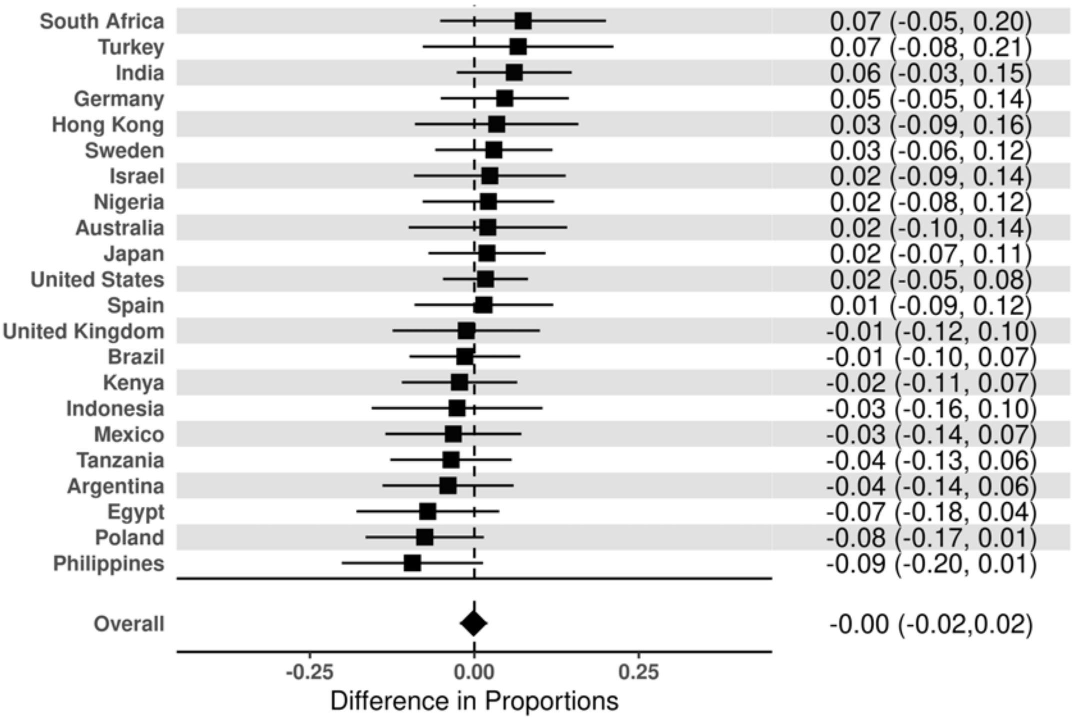

τ=0.000; Q(df=21)=15.93, p0.773;Q-profile 95% CI [0.000, 0.037]; I^2=0.00;

Figure S113. Forest plot for `Education`-`(Ref: Up to 8 years) 16+ years`

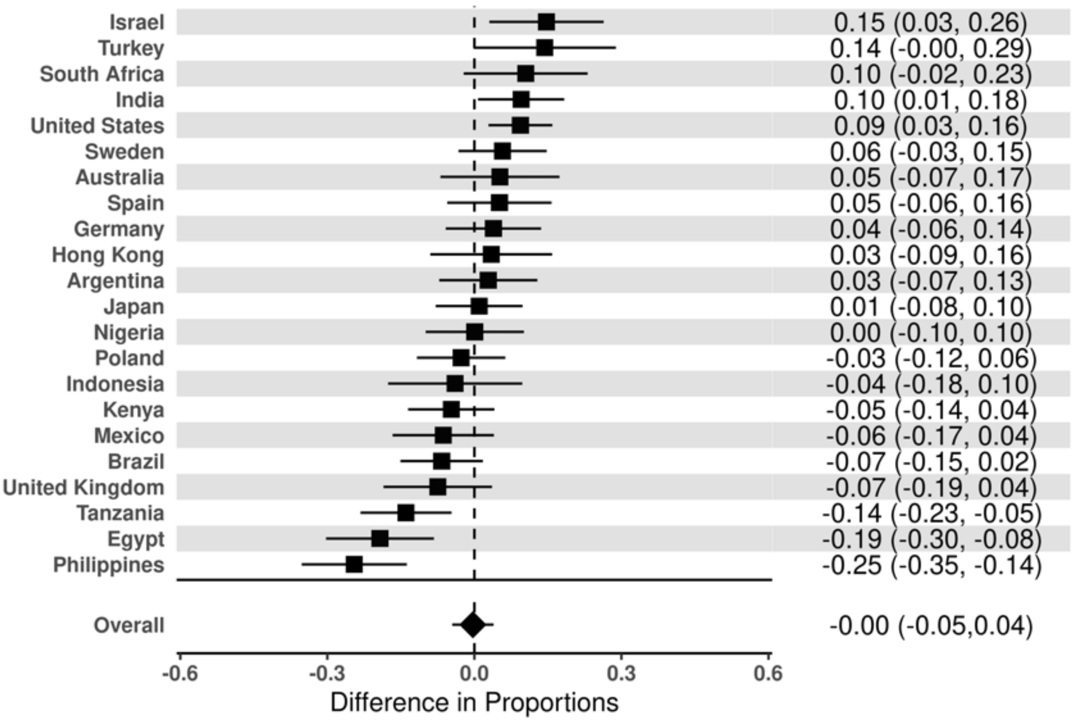

τ=0.086; Q(df=21)=77.33, p<.001;Q-profile 95% CI [0.052, 0.125]; I^2=73.99;

Figure S114. Forest plot for `Education`-`(Ref: 9-15 years) 16+ years`

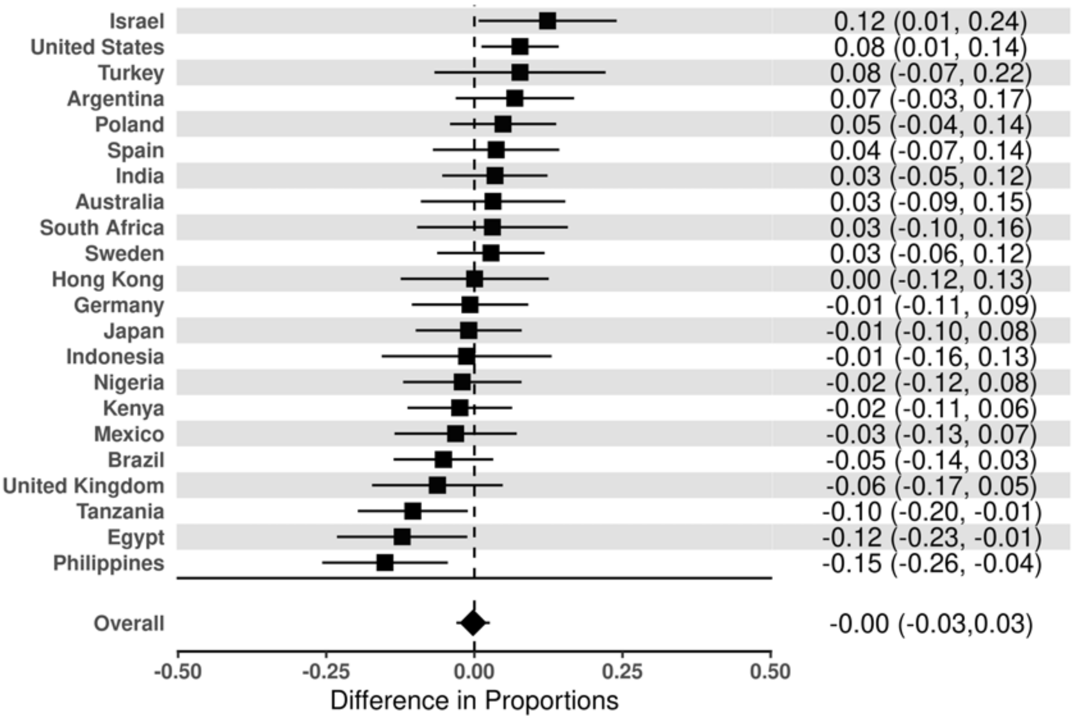

τ=0.043; Q(df=21)=36.54, p0.019;Q-profile 95% CI [0.000, 0.076]; I^2=41.16;

Figure S115. Forest plot for `Immigration status`-`(Ref: Born in this country) Born in another country`

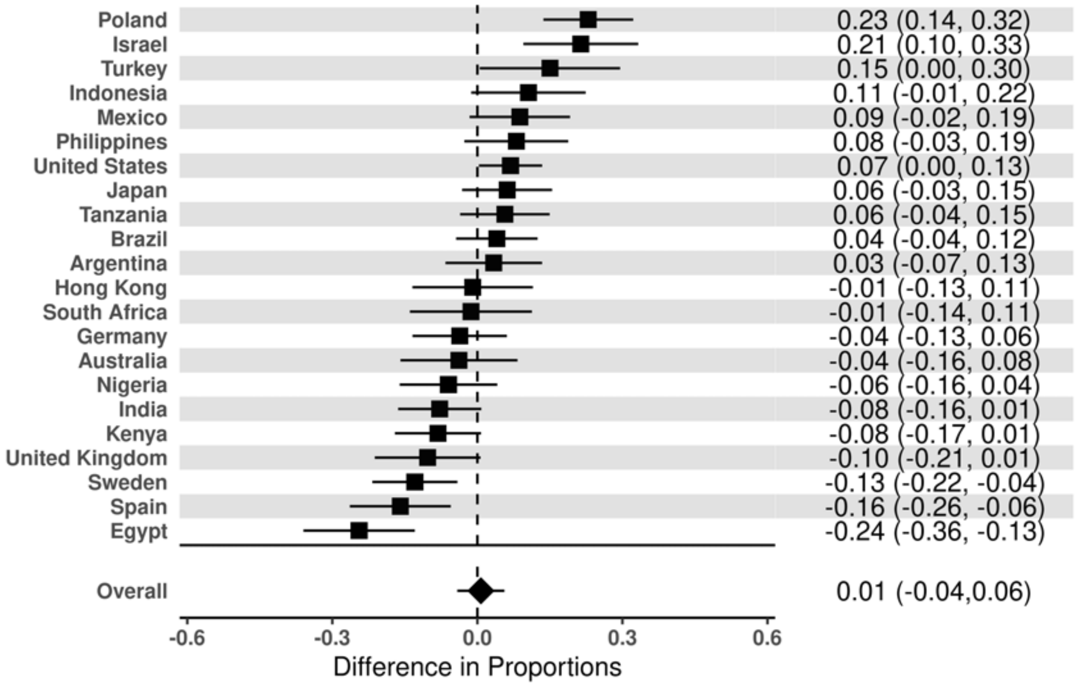

τ=0.104; Q(df=21)=102.78, p<.001;Q-profile 95% CI [0.068, 0.149]; I^2=80.74;
